# Use of P450 Enzymes for Late-Stage Functionalization in Drug Discovery

**DOI:** 10.1021/acs.jmedchem.5c01467

**Published:** 2025-10-12

**Authors:** Vincent Poon, Christopher Bailey, Sandra Carvalho, Stephen Patterson, James W. B. Fyfe, Olga Semenova, Stephen K. Wrigley, Emily Hopkins, Ravi Manohar, Christopher Drake, Tetsuo Kokubun, John Boyle, Lisbet Kvaerno, Kristin Lees, Karl F. Hoffmann, Josephine Forde-Thomas, Susan Wyllie, Jonathan Steele, Ian H. Gilbert, Gary J. Tarver

**Affiliations:** † Drug Discovery Unit, Wellcome Centre for Anti-Infectives Research, School of Life Sciences, 3042 University of Dundee Dundee DD1 5EH, U.K.; ‡ Mode of Action Group, Wellcome Centre for Anti-Infectives Research, School of Life Sciences, University of Dundee, Dundee DD1 5EH, U.K.; § 295794 Hypha Discovery Limited, 154B Brook Drive, Milton Park, Abingdon OX14 4SD, U.K.; ∥ Department of Life Sciences, 1026 Aberystwyth University, Penglais, Aberystwyth, Ceredigion SY23 3FL, U.K.

## Abstract

Herein, we demonstrate the use of a commercially available enzymatic kit to achieve late-stage hydroxylation of biologically relevant compounds by using the PolyCYPs screening kit. A selection of promising biotransformations were scaled up, products isolated, and structures elucidated. Isolated compounds were screened against a range of pathogens, namely, *Schistosoma mansoni*, *Leishmania donovani*, *Trypanosoma cruzi*, and *Trypanosoma brucei* to obtain biological data. This approach has allowed data generation more efficiently than the chemical synthesis of the same molecules. Importantly, it has been demonstrated that production of hits of interest can also be scaled up to enable further study. We also demonstrate the biosynthetic synthesis of a lead compound in fewer steps than using standard synthetic chemistry, offering faster access to compounds for screening or further transformation. This approach has the potential to save time and resources in a drug discovery program, by reducing the necessity to synthesize late-stage intermediates and develop new chemistry.

## Graphical abstract

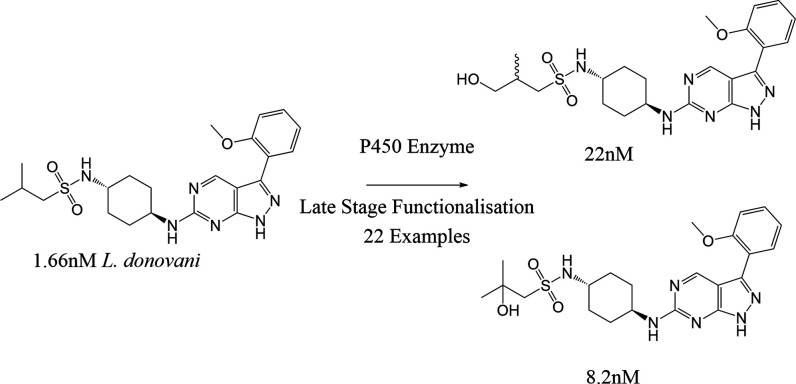

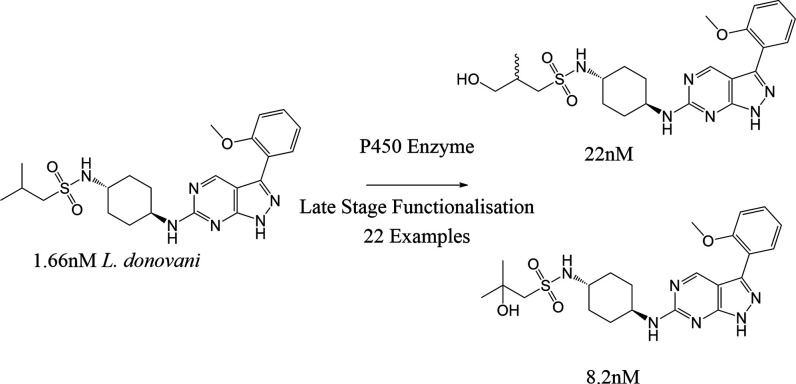

## Introduction

Medicinal chemistry campaigns often focus on the ability of synthetic chemists to produce large numbers of structural analogues of lead compounds in an efficient and timely manner. These pressures often bias the compounds synthesized toward relatively simple modifications using a limited set of robust synthetic reactions, rather than specific compounds required to answer key questions, which may be more synthetically challenging or require long synthetic schemes, going “back” many steps.^1^ This can often mean returning to the very beginning of a synthetic path in order to add a simple substituent or handle to a lead molecule on a synthetically inaccessible location, leading to a slowdown in compound optimization.

In addition to medicinal chemistry requirements, drug target deconvolution studies could benefit from specific functionalization of a lead molecule. Chemical pulldown is an unbiased technique used to identify protein(s) that directly bind to bioactive molecules. Vectors on a compound of interest must be identified where it is possible to attach a linker and a chemical handle to facilitate target pulldown from cells or cell lysates without compromising target engagement. This requires specific functionalization of lead molecules. It is also useful to functionalize lead molecules, typically with hydroxyl groups, to provide mass spectrometry standards for detection of metabolites.

We were interested to see if we could utilize biotransformations to achieve these modifications in a much more efficient and “green” manner. These are often mild and selective ways to modify a scaffold or molecule. However, despite the advantages and their increasing use in the production of pharmaceuticals, biotransformations are often still seen as a niche and inaccessible method for synthesis, with a substrate scope too limited for early stage drug discovery.^2–7^

The challenge of improving enzyme substrate scope has largely been addressed through advancements in both enzyme discovery and directed evolution, providing tools to develop enzymes that can better utilize a substrate to make a desired target product, as well as developing new-to-nature reactivity for an enzyme.^8–11^ However, despite its advantages, the timeframes involved in directed evolution and enzyme discovery often preclude its use in drug discovery where the focus is the rapid generation of compound analogues from a plethora of chemotypes. Such approaches have the additional hurdle of requiring the chemist to have advanced skills in molecular biology as well as access to appropriate equipment. An alternative to carrying out a time-intensive directed evolution campaign for each compound of interest is to use commercially available enzyme screening kits. These kits typically contain either tens or hundreds of variants of a particular class of enzyme that either naturally occur or are derived through mutagenesis. These kits provide rapid access to enzymes with the desired reactivity, with no advanced biological knowledge or skills needed and only limited specialized equipment. Further, different mutations can give rise to different substrate specificities, which is important in biotransformation of a novel synthetic substrate.

Hydroxylations are a particularly useful transformation in medicinal chemistry allowing for modulation of a compound’s physical properties such as solubility and Log *P*, modulating activity and off target effects, as well as providing a functional handle for further derivatization.^12^ These modifications also provide a handle for linker attachment in mode of action studies,^13^ potentially saving many synthetic steps on what can sometimes be a precious lead compound. There are a wide range of oxidative enzymes in nature including cytochrome P450s, flavin monooxygenases, alpha-ketoglutarate-dependent hydroxylases, aldehyde oxidases, and peroxygenases. Some enzymes within these classes are available in commercial kits to screen against compounds of interest, with many different substrate specificities available. These enzymes are attractive tools for the introduction of hydroxyl groups on a compound of interest that can selectively catalyze reactions at sp^2^ or sp^3^ centers even with other reactive groups present on the molecule of interest. They are also of interest for metabolite identification and the preparation of analytical standards thereof. There are several commercially available formats encompassing mammalian and bacterial-derived cytochrome P450 enzymes expressed in recombinant *Escherichia coli*, yeast, and insect expression systems, some of which are available in preassembled screening kits suitable for nonbiologists’ use, such as Codexis MicroCYPs and the kit used herein, Hypha Discovery’s PolyCYPs.^14^ Such kits have been used for metabolite identification and metabolite scale-up reactions of drug molecules in-house.^15^

In this paper, we demonstrate how a commercially available enzyme screening panel comprising microbial cytochrome P450s complemented with human flavin monooxygenases and aldehyde oxidase can provide a simple and expeditious route to the late-stage functionalization of lead compounds from several medicinal chemistry projects investigating the treatment of multiple neglected tropical diseases. We demonstrate this with leads from several current and historical drug discovery programs, on a variety of different chemotypes. We show how initial hits through the enzyme panel can be used to investigate the structure–activity relationships (SARs) of the lead compounds and how such hits can be scaled up to gram scale for further development. The kit used in this approach is a convenient method to access compounds of interest and generate start points for further enzyme engineering to achieve selective functionalization as required. Some of the cytochrome P450s in the panel are engineered enzymes generated by site-directed mutagenesis to broaden the substrate selectivity.

## Results and Discussion

### PolyCYPs Panel Screening

PolyCYPs enzymes are cytochrome P450 enzymes mined from talented biotransforming strains in Hypha’s microbial collection and expressed with ferredoxin and ferredoxin reductase redox partners in *E. coli*. The PolyCYPs metabolite kits have been developed into a lyophilized screening kit and include human flavin-containing monooxygenase (FMO) and human aldehyde oxidase (AO). These enzymes were selected for inclusion in the kit based on extensive biocatalytic activity against a diverse panel of substrates and are capable of oxidizing groups in a wide range of substrate compounds.

Twelve structurally diverse lead compounds were identified from in-house antiparasitic drug discovery programs to screen against a PolyCYPs panel consisting of twenty-three PolyCYPs enzymes, five FMO enzymes, and one AO enzyme. Each of the chosen compounds had previously been shown to have good activity in parasite assays.

As per the instructions for PolyCYPs kit use, compounds were screened at a concentration of 0.1 mg/mL, a concentration typically used for preliminary screening with PolyCYPs to reduce risk of enzyme inhibition effects, as well as being a suitable concentration for direct use for initial scale-up of reactions toward milligram amounts of product for structural identification and preliminary activity testing purposes. Reactions were prepared by mixing resuspended lyophilized PolyCYP extracts with test compound stock solutions (e.g., 0.4% v/v DMSO) and formulants where needed (5% or 10% v/v addition of e.g., 40% w/v hydroxypropyl-beta-cyclodextrin). Reactions were initiated by the addition of a premixed cofactor regeneration system containing nicotinamide adenine dinucleotide phosphate, glucose-6-phosphate, and glucose-6-phosphate dehydrogenase at 10% v/v.

While monitoring the reactions by ultraperformance liquid chromatography–mass spectrometry (UPLC–MS), we observed that all 12 compounds screened showed conversion to at least one new product across the panel of enzymes tested. Closer inspection of the data, however, indicated that higher yielding reactions for compounds **1–4** were, for example, hydrolyses yielding +18 Da products rather than the preferred oxidations yielding +16 Da products from cytochrome P450s. Compounds **5–12** were therefore selected for scale-up reactions to produce mg quantities of products for structural characterization (see below and the Supporting Information). Ten of the PolyCYPs enzymes were judged to have performed well against the 12 compounds, while three were considered to have performed poorly. The best-performing cytochrome P450s included PolyCYP194 (see below), PolyCYP6 and its mutants PolyCYPs 166, 477, and 478, which were effective in achieving aromatic hydroxylations, and PolyCYP168 and its mutants PolyCYPs 479, 484, 486, and 488, which were effective in achieving aromatic and aliphatic hydroxylations as well as, in one case, O-demethylation. The FMO and AO enzymes were less successful, although some FMO +16 Da products were not selected for scale-up, as they were likely to be N-oxide products and therefore of low interest.

From these results we noted a particularly apparent promiscuity of PolyCYP194 which catalyzed reactions for five separate starting materials (three structurally distinct classes; **5–7**, **10**, and **11**) giving a total of nine different compounds. PolyCYP194 had previously not proved promiscuous in screening against pharmaceutical targets and had previously only been effective against some peptidic compounds, but this was the first time the PolyCYPs enzymes had been tested against a set of antiparasitic leads. In the present project, PolyCYP194 generally catalyzed aliphatic hydroxylations and may be particularly suited to oxidizing some of the building blocks used to generate these leads. Such promiscuity could be a useful tool for generating many different analogues of a compound of interest to either scope out SARs or to determine the optimal placement of a linker for pull-down experiments for target determination.

### Milligram Scale Reactions

Once the initial hits were identified, we next sought to scale up the reactions of eight selected compounds (**5–12**) to obtain enough material to characterize the products by nuclear magnetic resonance (NMR) and for downstream biological testing. Although hits were obtained for compounds **1–4**, their projected yields were not deemed suitable for scale-up investigations. For this initial scale-up and structure elucidation step, we required a minimum of 0.5 mg of each product. To achieve this, we scaled up each of the enzymatic reaction volumes to between 30 and 250 mL, at a 0.1 mg/mL substrate concentration using either lyophilized scale-up vials or fresh enzyme extract from *E. coli*-derived pellet processing. From these reactions, we obtained each of the twenty-three previously observed products in between 0.66 mg–11.16 mg yield after purification. Reaction details of specified scaled-up reactions are provided in Table 1. Structural elucidation by NMR of the isolated products showed that a range of transformations had occurred mediated by enzyme catalysis, including hydroxylation (**5b**), carboxylation (hydroxylation followed by oxidation) (**5a**), oxidative ring opening (**9**), and demethylation (**12**) (Figure 1). These findings showcase some of the diverse chemistries that oxidative enzymes are known to catalyze.^16^

**Table 1:** Scale-Up of Tractable Enzymatic Conversions

compound	PolyCYP isoform	scale-up approach	reaction volume (ml)	parent dose (mg)	product	amount produced (mg)	isolated yield (%)
**5**	194	lyophilized vials	100	10.00	**5a**	1.95	19.5
					**5b**	0.94	9.4
	488	lyophilized vials	256	25.75	**5c**	1.41	5.5
					**5d**	0.68	2.6
					**5e**	1.45	5.6
**6**	152	lyophilized vials	259	26.00	**6a**	0.66	2.5
					**6b**	2.68	10.3
	194	lyophilized vials	31	3.00	**6c**	2.50	83.3
	483	lyophilized vials	71	7.00	**6d**	2.36	33.7
					**6e**	0.59	8.4
**7**	194	lyophilized vials	106	10.75	**7a**	2.74	25.5
					**7b**	2.57	23.9
**8**	166	lyophilized vials	100	10.00	**8a**	0.73	7.3
**9**	166	lyophilized vials	259	26.00	**9a**	2.91	11.2
					**9b**	1.15	4.4
					**9c**	11.16	42.9
**10**	194	lyophilized vials	122	12.25	**10a**	0.97	7.9
					**10b**	1.41	11.5
**11**	194	lyophilized vials	106	10.75	**11a**	1.20	11.2
					**11b**	0.98	9.1
**12**	168	fresh enzyme from stock pellet	201	17.35	**12a**	2.95	17.0
					**12b**	1.04	6.0
					**12c**	0.74	4.3

**Figure 1:**
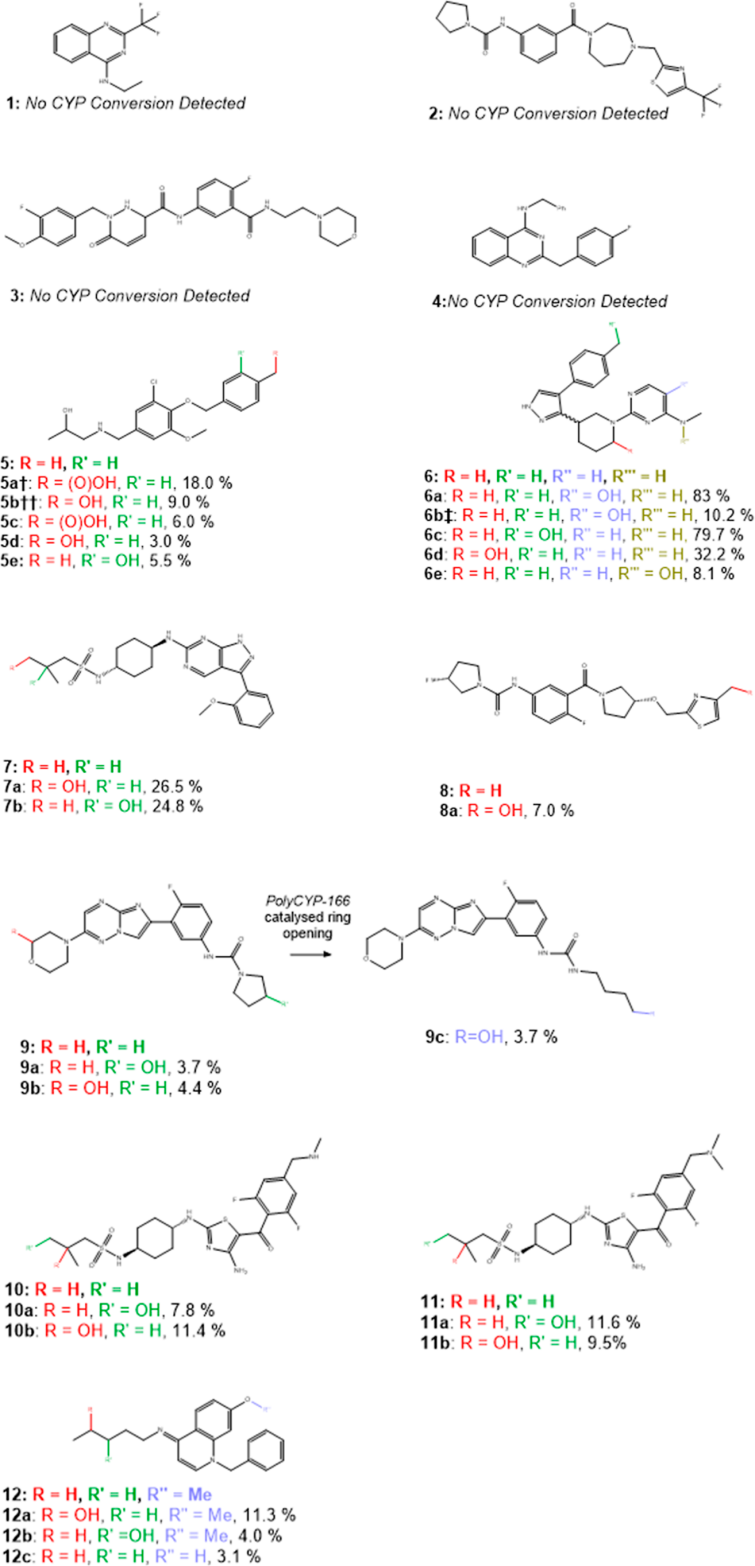
Structures of parent compounds and products from PolyCYP reactions indicating isolated yields. Structures determined by NMR.

Hydroxylation of the starting material was the most common type of reaction that we observed. These reactions occurred at a variety of positions including aromatic, aliphatic, and benzylic positions as well as one instance of *N*-hydroxylation (**6e**). Some of the positions, such as the products of the hydroxylation of **5** were in positions that may be accessible to standard chemical oxidations. However, other examples such as the products of the reactions with **10**, **11**, and **12** are in positions that would be extremely challenging to hydroxylate via standard chemical methods and would likely require a total resynthesis of the compound incorporating the hydroxy group at a much earlier stage. This is demonstrated through the chemical synthesis of **6c**, where even hydroxylation of a benzylic position could not be achieved via late-stage functionalization in our hands (see below).

Another particularly intriguing transformation we observed was the oxidative ring opening of pyrrolidine in **9**. We reasoned that the first step in this reaction was likely to be the α-hydroxylation of the pyrrolidine followed by N–C bond cleavage. However, when this transformation has been described in the literature previously the product is the resulting aldehyde rather than the corresponding alcohol. We reasoned that as the PolyCYPs used for the screening are present within an *E. coli* crude lysate an endogenous ketoreductase (KRED) may be responsible for the reduction of any aldehyde product of the PolyCYP reaction to the corresponding alcohol (Figure 2).

**Figure 2:**
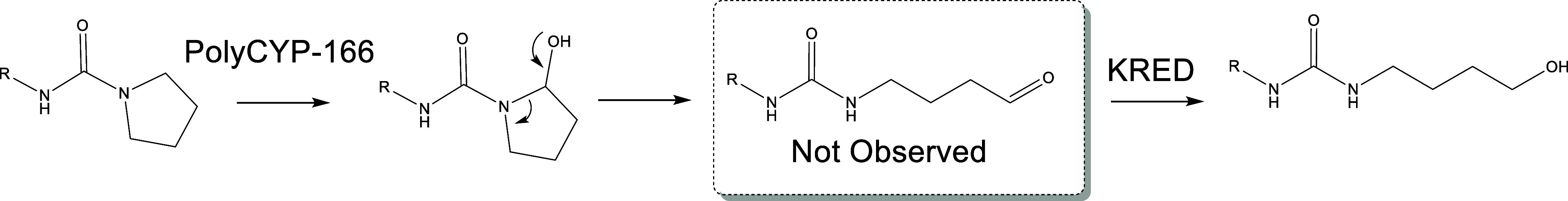
Postulated mechanism for the formation of the primary alcohol on **9C** via α-hydroxylation of the pyrrolidine, ring opening, and subsequent reduction by a putative endogenous KRED.

### Biological Screening

A total of twenty-three compounds were successfully isolated in sufficient quantity and purity for screening in biological assays. Each of the compounds was screened against the same biological assay(s) their parent compounds were screened against; full details of parasites and assays can be found in Table 2. In Table 3, assay results for screened compounds are shown.

**Table 2:** Parasites Compounds Screened against, Showing Assay Details and Disease Caused by Each Parasite

Pathogen	Disease	Assay notes
*Schistosoma mansoni*	Schistosomiasis	Schistosomula
*Leishmania donovani*	Visceral Leishmaniasis	Promastigotes
*Trypanosoma cruzi*	Chagas Disease	In vero amastigotes
*Trypanosoma brucei*	African Sleeping Sickness	Bloodstream form

**Table 3:**
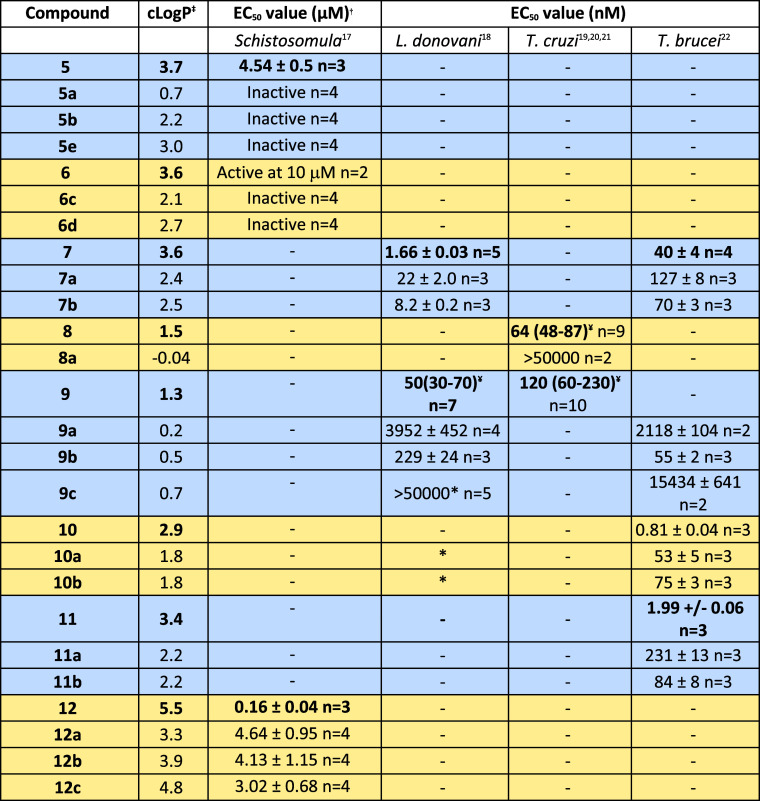
Biological Activity of Parent Compounds and Products of P450 Reactions[Table-fn t3fn6] ^17–22^

* Indicates biphasic curve in the assay.

- Indicates not measured in the assay.

† EC_50_ values quoted for schistosomula motility.

‡ *c* Log *P* calculated using ChemDraw Professional 22.0.0.22.

¥ 95% confidence intervals.

a Parent compounds shown in bold.

For the compounds derived from **7**, a slight drop in activity could be seen for the primary alcohol **7a** when tested against *L. donovani* and *T. brucei*. The tertiary alcohol **7b** showed activity similar to that of **7**. What this demonstrates is that simple changes offer modulation of physicochemical properties and offer the potential for a handle for further derivatization, with a simple one-step biotransformation.

For the compounds derived from both **10** and **11,** the primary and tertiary alcohol derivatives, **10a**, **10b**, **11a**, and **11b** maintained reasonable activity with all compounds displaying an EC_50_ below 250 nM in the *T. brucei* assay. However, for **10a** and **10b**, in the *L. donovani* assay, a biphasic curve was noted. Biphasic curves are characteristic where there are several different mechanisms of action. This shows that even simple modifications to a molecule can cause changes in molecular targets. Hence, it is important to track the mode of action during optimization of a compound.

For five of the metabolites, activity from 229 nM to 15 μM was observed against *L. donovani* and *T. brucei*. Compound **9c** gave a biphasic assay curve.

#### Chemical Synthesis of **6c**

The chemical synthesis of **6c** was investigated as a comparison to the enzymatic synthesis. Initially, attempts were made to directly hydroxylate **6** using KMnO_4_ or IBX. Unfortunately, both of these led to degradation of the starting material. A further attempt was made to brominate at the benzylic position to allow subsequent conversion to the primary alcohol and afford the same conversion that had been demonstrated enzymatically. In this case, bromination occurred to give a regio-isomeric product.

After these unsuccessful attempts to chemically synthesize **6c**, we resorted to modifying the multistep route previously used to synthesize **6**. By substituting the *p*-tolylboronic acid in step 2 for 4-(hydroxymethyl)­phenylboronic acid we were able to successfully synthesize **6c** in an overall yield of 17% (Figure 3). This is comparable to that for **6** but when compared to the enzymatic route requires a multistep synthesis to access the hydroxylated compound rather than a single late-stage enzymatic transformation. Thus, the enzymatic route is the preferred route to obtain material to screen.

**Figure 3:**
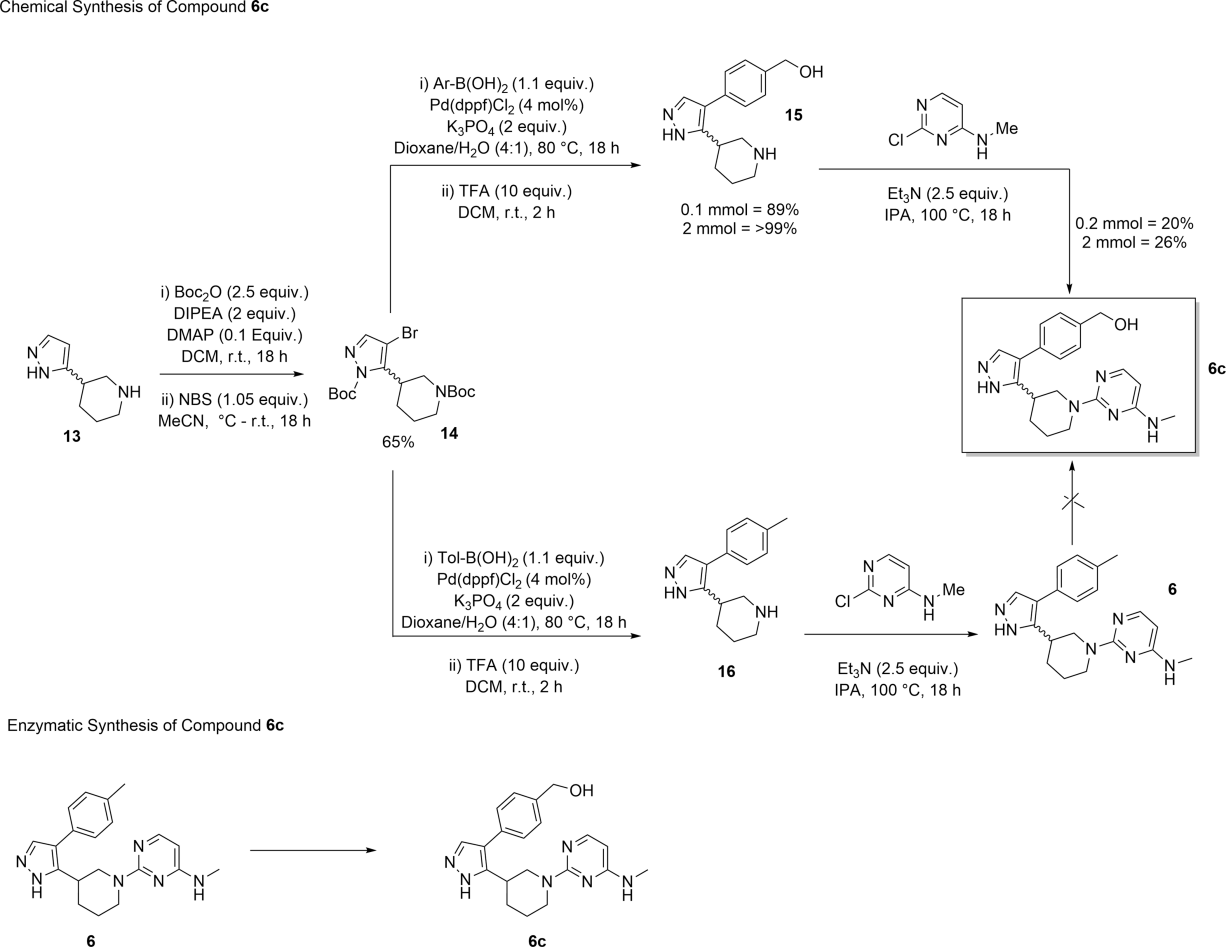
Synthesis of **6c** by chemical and enzymatic hydroxylation.

#### Biotransformation Scale-Up of Selected Compounds

One of the difficulties often associated with biotransformations in synthetic chemistry laboratories is how to scale reactions up to beyond the milligram scale. This difficulty is largely down to two key factors: enzyme availability and reaction concentration. Both these challenges can be addressed through close collaboration between medicinal chemistry laboratories and enzyme screening kit companies. While, particularly for academic laboratories, the cost of buying sufficient enzyme for scale-up reactions may seem high, the cost of extensive chemical synthesis in terms of time also needs to be considered. The second challenge to scaling up enzyme reactions is the relatively dilute substrate concentrations needed in the reactions compared to those of standard chemical methods. These dilute reaction conditions result in reaction volumes much larger than those usually encountered in standard chemistry laboratories. However, as enzymatic reactions are carried out in aqueous buffers, they can often be carried out in shake flasks within incubators, the same as those used in the production of the enzyme initially.

To test the feasibility of scaling up these reactions, we set out to scale-up the enzymatic biotransformation of five products identified (**6c**, **7a**, **7b**, **11a**, **11b**) using three different starting materials (**6**, **7, 11**) in the original screen to 50 mg–1 g scale. It was hoped that this would demonstrate that hits of interest can be further scaled-up to quantities that may be needed for any downstream studies.

To expedite this work, we chose to use a whole cell approach. This approach has the advantage of being quicker to produce the required enzymes in larger volumes and to reduce the use of costly cofactors as they are generated in vivo. For all three scale-up reactions, the same P450 enzyme, PolyCYP194, was used.

To facilitate this work, we used the already prepared *Streptomyces lividans* strain engineered to express the PolyCYP194 gene alongside its redox partners in a polycistronic operon under the control of the constitutive promotor ermE* to produce strain *S. lividans* HD005. This strain^23^ was then tested for its viability as a whole-cell biocatalyst by carrying out dose-confirmation experiments with starter materials (**6**, **7**, and **11**), measuring for production of **6a**, **7a**, **7b**, **11a**, and **11b**. Concentrations ranging from 100–500 mg/L were screened, as well as different times postinoculation for the dosing of the starter material. Time points were taken for analysis at 24, 48, and 72 h postdosage.

Maximum conversion was observed when **6** was dosed at 24 h postinoculation at a concentration of 500 mg/L and harvesting carried out 72 h postdosing. These conditions were subsequently used to scale the reaction up to a total of 3 L of culture, dosing 1.5 g of starting material. Pleasingly from this reaction we achieved an isolatable yield of 59%, corresponding to almost 900 mg of the product.

Maximum conversion from dose-confirmation experiments with **7**, measuring for production of **7a** and **7b**, was dosed at 48 h postinoculation at a concentration of 150 mg/L and harvesting carried out 24 h postdosing. These conditions were used to scale the reaction up to a total of 4.7 L of culture, dosing 705 mg of starting material, and we achieved an isolatable yield of 34.6% and 27.5% for **7a** and **7b**, respectively, corresponding to 244 mg and 194 mg.

Maximum conversion from dose-confirmation experiments with **11**, measuring for production of **11a** and **11b**, was dosed at 72 h postinoculation at a concentration of 500 mg/L and harvesting carried out 24 h postdosing. These conditions were used to scale the reaction up to a total 0.45 L of culture, dosing 220 mg of starting material and 81 and 49 mg of **11a** and **11b,** respectively, were successfully scaled-up.

Across all scale-up reactions, we were successful in generating a total 1.45 g of products (Figure 4 and Table 4).

**Figure 4:**
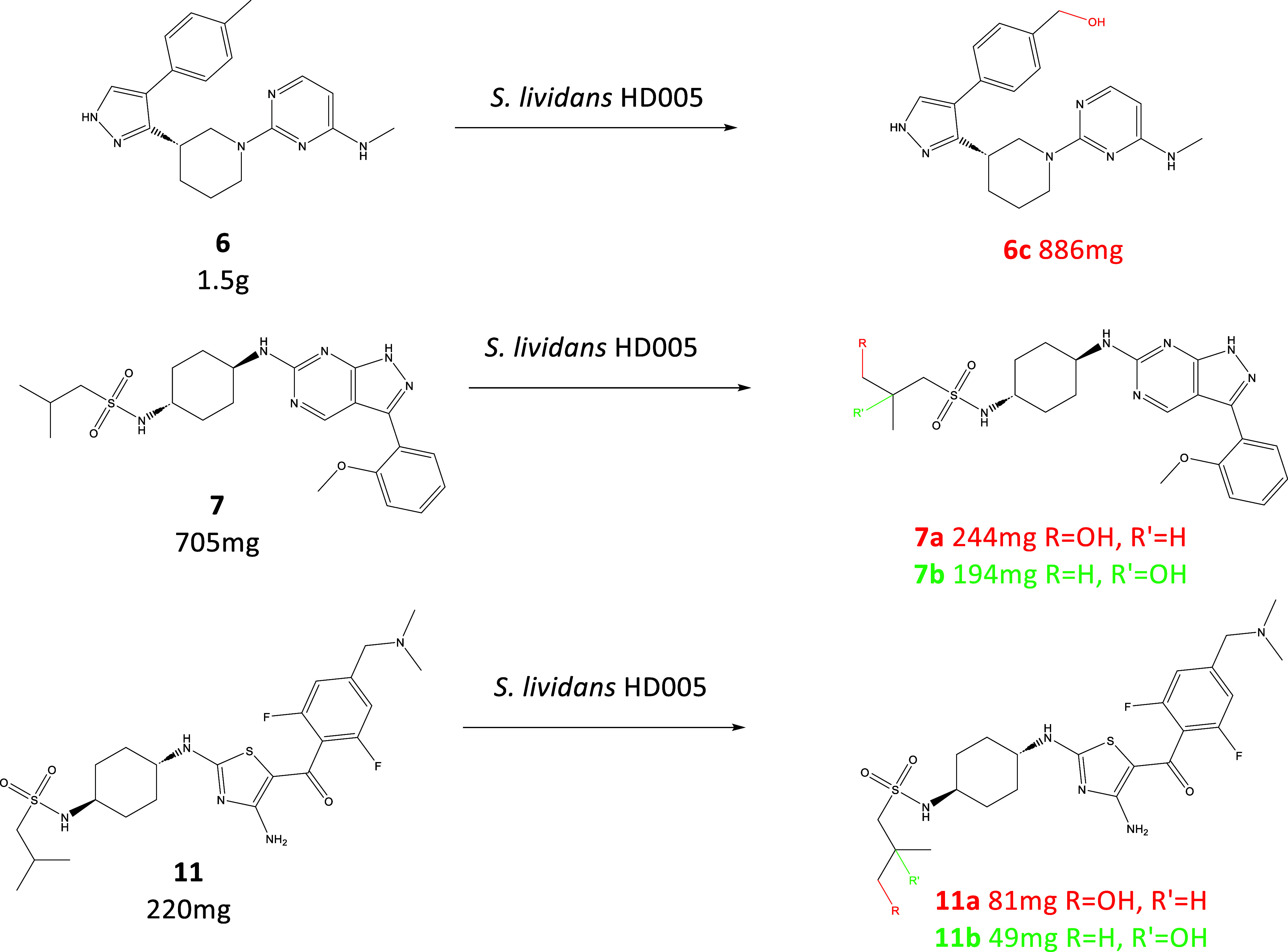
Scale-up of **6c**, **7a**, **7b**, **11a**, and **11b**.

**Table 4:** Scale-Up of Selected Compounds for Further Studies

parent compound	PolyCYPs enzyme	S. lividans strain	reaction volume (ml)	Product	amount produced (mg)
**6**	194	HD005	3000	**6c**	885.58
**7**	194	HD005	4700	**7a**	243.88
				**7b**	194.12
**11**	194	HD005	450	**11a**	80.82
				**11b**	48.64

## Conclusions

A series of 12 known bioactive compounds have been screened against a PolyCYPs panel containing 23 microbial cytochrome P450 enzymes, 5 human flavin-containing monooxygenase enzymes, and one human aldehyde oxidase enzyme. Hits were obtained against all 12 compounds screened but hits from 4 compounds (**1–4**) were deprioritized as their projected yields were judged unsuitable for further investigation in a screening project of this type. Twenty-three products were selected and successfully scaled up from 8 starting materials (**5–12**) to produce milligram amount quantities for structure elucidation and testing for biological activities. Five of these products (**6c**, **7a**, **7b**, **11a,** and **11b**) were further scaled-up using whole cell biotransformations by a recombinant *S. lividans* strain of a single PolyCYPs enzyme (PolyCYP194) in several-liter volumes to produce between 49 and 885 mg of target product. This method of scale-up is cost-effective when larger quantities of a desired product are needed. It is noteworthy that use of the PolyCYPs panel had previously been focused on compounds active in other therapeutic areas and had not previously been tested against antiparasitic compounds, and that PolyCYP194 had not shown any significant promiscuity. The present study has shown that this enzyme is effective for aliphatic hydroxylations, particularly for some of the building blocks used for the compounds screened herein. It was complemented by other enzymes in the panel producing different products.

Several different reactions were observed as the outcome of these reactions, with the most prevalent reaction being hydroxylation. Other observed reactions included further oxidation of the new methyl hydroxyls to carboxyl groups, oxidative ring openings, and demethylations. In many instances, these reactions occurred on positions that would be challenging to perform late-stage functionalization on by chemical synthesis and would likely require a ground-up resynthesis of the compound to obtain the same product. We demonstrated this through the chemical synthesis of one of the products of the enzymatic reactions, **6c**.

Each of the isolated compounds was tested for biological activity in the assay in which its parent compound was originally found to have biological activity. Ten of the isolated compounds showed significant biological activity, demonstrating the utility of this approach to generate biologically active compounds. It is not unexpected that the levels of bioactivity are less potent than those of the parent molecules, but these biotransformations serve to allow further elaboration of a lead and to add handles to compounds for mode of action studies. They also allow other properties to be modulated, for example, increased solubility, for which a trade off with activity would be acceptable within limits.

This demonstrates that biotransformations can be successfully used in drug discovery programs, potentially shortening synthetic chemistry efforts. We have also demonstrated that it is possible to screen and scale up the biotransformation reaction in-house using commercially available products such as the PolyCYPs enzyme technology.

## Experimental Section

All compounds are >95% pure except the following compounds. Compounds **5b** and **5e** were minor components isolated with purities of 70% and 76%, respectively. They were screened crude and showed no biological activity in the schistosomula assay. Compounds **6b**, **6d**, and **6e** were isolated with purities of 17%, 43%, and 16%, respectively. The only compound screened was **6d** which was screened crude and showed no activity in the schistosomula assay. Compound **9b** was isolated at 87% purity but is a hemiacetal so in equilibrium with the open-chain form. Compound **10b** was isolated at 94% purity and was accepted for the assay.

UPLC-UV-MS analyses were conducted using an H-Class Acquity UPLC system consisting of a photodiode array detector (scanning 201 to 499 nm at 20 Hz at a resolution of 1.2 nm) and a QDa single quadrupole mass detector (Waters Corporation, Milford, MA), scanning from 101 to 1250 *m*/*z* in alternate positive and negative modes at 2 Hz.

For acid conditions, 1 μL injections of the enzyme reaction extracts were analyzed on a Waters BEH Shield RP18 column (1.7 μm, 2.1 mm i.d. × 50 mm length) at 45 °C eluted with a water–acetonitrile (MeCN) gradient in the presence of 0.1% formic acid, increasing linearly from 2 to 98% MeCN over a period of 2.4 min, holding at that concentration for a further 0.4 min and then returning to the starting conditions over 0.05 min and re-equilibrating for 0.15 min, all at a flow rate of 1.0 mL/min.

For base conditions, 1 μL injections of the enzyme reaction extracts were analyzed on a Waters BEH Shield RP18 column (1.7 μm, 2.1 mm i.d. × 50 mm length) at 45 °C eluted with water–MeCN gradient in the presence of 10 mM ammonium bicarbonate using the same gradient as described above.

High-resolution LCMS (HRMS) analysis was conducted using an Orbitrap Exploris 120 system with an H-ESI probe. The instrument was set to measure between 80 and 1000 *m*/*z* in positive ionization mode. A 2 μL injection of a 250 μM solution of the compound was analyzed on a Thermo Scientific Hypersil GOLD 50 × 2.1 mm ID column with a particle size of 1.9 μm, eluting with a water–MeCN gradient of 0.1% formic acid, increasing linearly from 2 to 98% MeCN over 3.0 min, holding at that concentration for a further 1.0 min and then returning to the starting conditions over 0.05 min, all at a flow rate of 0.5 mL/min.

For schistosomiasis assays, all procedures performed on mice adhered to the United Kingdom Home Office Animals (Scientific Procedures) Act of 1986 (project licenses: PP2955700 and P3B8C46FD) as well as the European Union Animals Directive 2010/63/EU and were approved by Aberystwyth University’s Animal Welfare and Ethical Review Body.

## Supplementary Material





## Notes

Molecular formula string data for compounds with associated biological activity (CSV)
Details of enzymatic reactions, biological assays, chemical synthesis, and analytical data (PDF)

## Abbreviations

AO: aldehyde oxidase
DIPEA: diisopropylethylamine
FMO: flavin-containing monooxygenase
H-ESI: heated-electrospray ionization
IPA: isopropyl alcohol
LCMS: liquid chromatography mass spectrometry
Et_3_N: triethylamine
UPLC: ultra performance liquid chromatography
