# Supplementary material for: Use of P450 Enzymes for Late-Stage Functionalization in Drug Discovery
Source: J Med Chem. 2025 Oct 12;68(20):21479–88. doi: 10.1021/acs.jmedchem.5c01467 (PMC12557363; doi:10.1021/acs.jmedchem.5c01467)
Supplement: Supplementary file 2 [file jm5c01467_si_002.pdf]

Supporting Information

Use of P450 Enzymes for Late-Stage Functionalisation in Drug Discovery

Vincent Poon [c], Christopher Bailey [a], Sandra Carvalho [b], Stephen Patterson [b], James W. B. Fyfe [a], Olga Semenova [a], Stephen K. Wrigley [c], Emily Hopkins [c], Ravi Manohar [c], Christopher Drake [c], Tetsuo Kokubun [c], John Boyle [c], Lisbet Kvaerno [c], Kristin Lees [d], Karl F. Hoffmann [d], Josephine Forde-Thomas [d], Susan Wyllie [b], Jonathan Steele[c], Ian H. Gilbert [a*], Gary J. Tarver [a*]

[a] Drug Discovery Unit, Wellcome Centre for Anti-Infectives Research, School of Life Sciences, University of Dundee, DD1 5EH, UK.

[b] Mode of Action group, Wellcome Centre for Anti-Infectives Research, School of Life Sciences, University of Dundee, DD1 5EH, UK.

[C]Hypha Discovery Limited, 154B Brook Drive, Milton Park, Abingdon, OX14 4SD, UK.

[d] Department of Life Sciences, Aberystwyth University, Penglais, Aberystwyth, Ceredigion, SY23 3FL, UK.

Corresponding authors: g.j.tarver@dundee.ac.uk; i.h.gilbert@dundee.ac.uk

Contents

1 Contents S2

2 General S4

2.1 Reagents S4

2.2 Instruments S4

3 Enzymatic Reactions S5

3.1 Panel Screening S5

3.2 Scaled-up Biotransformation Reactions S5

3.3 Generic purification of scaled-up reactions S17

3.4 Whole cell biotransformation scaled-up reaction of compound **6** S26

3.5 Purification of compound **6c** S27

3.6 Whole cell biotransformation scaled-up reaction of compound **7** S28

3.7 Purification of compound **7a** and **7b** S29

3.8 Whole cell biotransformation scaled-up reaction of compound **11** S30

3.9 Purification of compounds **11a** and **11b** S31

3.10 Biological Assays S33

4.1 Schistosomula S33

4.2 Ld pro S33

4.3 Tc-Invero S34

4.4 Tb bsf S34

4.5 Assay Results S34

5 Chemical Synthesis S38

5.1 Synthesis of Compound **6** S39

5.2 Synthesis of Compound **6c** S41

6.1 tert-butyl 3-(1-(tert-butoxycarbonyl)-1H-pyrazol-3-yl)piperidine-1-carboxylate S38

6.2 tert-butyl 3-(4-bromo-1-(tert-butoxycarbonyl)-1H-pyrazol-3-yl)piperidine-1-carboxylate S39

6.3 3-(4-(p-tolyl)-1H-pyrazol-3-yl)piperidine S39
6.4 N-methyl-2-(3-(4-(p-tolyl)-1H-pyrazol-3-yl)piperidin-1-yl)pyrimidin-4-amine S46
6.5 (4-(5-(1-(4-(methylamino)pyrimidin-2-yl)piperidin-3-yl)-1H-pyrazol-4-yl)phenyl)methanol S47
6.6 Analytical Data S48
6.6 Compound **5a** S48
6.7 Compound **5b** S57
6.8 Compound **5e** S65
6.9 Compound **6b** S75
6.10 Compound **6c** S82
6.11 Compound **6d** S91
6.12 Compound **6e** S96
6.13 Compound **7a** S100
6.14 Compound **7b** S101
6.15 Compound **8a** S118
6.16 Compound **9a** S123
6.17 Compound **9b** S132
6.18 Compound **9c** S141
6.19 Compound **10a** S150
6.20 Compound **10b** S158
6.21 Compound **11a** S167
6.22 Compound **11b** S175
6.23 Compound **12a** S184
6.24 Compound **12b** S192
6.25 Compound **12c** S200
7 References S200

General

Reagents

Reagents were used as received unless otherwise stated.

Instruments

Nuclear Magnetic Resonance

NMR analysis was performed using either a Bruker AVANCE III HD 500 MHz spectrometer running under TopSpin v.3.6.4 and equipped with a QCI-F cryo-probe at a sample compartment temperature of 25ºC or, for compounds isolated in the lowest yields, spectra were acquired at the University of Bristol on a Bruker AVANCE III HD 700 NMR spectrometer equipped with a 1.7 mm microcryoprobe. The samples for structure elucidation were all generated on a small scale and DMSO-*d6* was generally a pragmatic choice of NMR solvent but some of the resulting spectra were quite broad and not all the carbon signals could be detected directly, in which case these were detected indirectly through the HSQC and HMBC heteronuclear shift correlation spectra.

Ultra-Performance Liquid Chromatography – Mass Spectrometry

UPLC-UV-MS analyses were conducted using an H-Class Acquity UPLC system, consisting of a photodiode array detector (scanning 201 to 499 nm at 20 Hz at a resolution of 1.2 nm) and a QDa single quadrupole mass detector (Waters Corporation, Milford, MA), scanning from 101 to 1250 m/z in alternate positive and negative modes at 2 Hz.

For acid conditions, 1 µl injections of the enzyme reaction extracts were analysed on a Waters BEH Shield RP18 column (1.7 µm, 2.1 mm i.d. x 50 mm length) at 45°C eluted with a water-acetonitrile (MeCN) gradient in the presence of 0.1% formic acid, increasing linearly from 2 to 98% MeCN over a period of 2.4 mins, holding at that concentration for a further 0.4 mins and then returning to the starting conditions over 0.05 mins and re-equilibrating for 0.15 mins, all at a flow rate of 1.0 mL/min.

For base conditions, 1 µl injections of the enzyme reaction extracts were analysed on a Waters BEH Shield RP18 column (1.7 µm, 2.1 mm i.d. x 50 mm length) at 45°C eluted with water-MeCN gradient in the presence of 10 mM ammonium bicarbonate using the same gradient as described above.

High-Resolution LCMS (HRMS) analysis was conducted using an Orbitrap Exploris 120 system with a H-ESI probe. The instrument was set to measure between 80 – 1000 m/z in positive ionization mode. A 2 µl injection of a 250 µM solution of the compound to be analysed were analysed on a Thermo Scientific Hypersil GOLD 50 x 2.1 mm ID column with a particle size of 1.9 µm, eluting with a water-MeCN gradient of 0.1% formic acid, increasing linearly from 2

to 98% MeCN over 3.0 mins, holding at that concentration for a further 1.0 min and then returning to the starting conditions over 0.05 min, all at a flow rate of 0.5 mL/ min.

The HPLC-grade solvents (acetonitrile, formic acid) were from Fisher Scientific (Leicester, UK), and the water (resistivity 18.2 MΩ) was prepared in-house using a Milli-Q Synergy UV system (Millipore, Molsheim, France).

Enzymatic Reactions

1. Panel Screening

Parent compounds were screened against a panel of recombinant enzymes consisting of twenty-three microbial PolyCYPs® cytochrome P450 enzymes, five human FMO (Flavin-containing monooxygenase) enzymes and one human recombinant AO (aldehyde oxidase) enzyme, as follows.

The reactions were performed in V-well 96-well polypropylene microtitre plates at 100 µL reaction volumes. Each reaction comprised in order of addition, 0.4 µL test compound at 25 mg/mL stock solution in DMSO, 89.6 µL recombinant enzymes (from 500 µL stock prepared in cold H_2O) and 10 µL cofactor reagent stock solution to initiate reactions (50 mM glucose-6-phosphate [G6P], 10 mM nicotinamide adenine dinucleotide phosphate [NADP+], 10 UN/mL glucose-6-phosphate dehydrogenase [G6PDH], 5 mM $MgCl_2$ and 100 mM potassium phosphate (KPi) buffer at pH 8 dissolved in cold H_2O) to give final concentrations of 0.1 mg/mL test compound, 5 mM G6P, 1 mM NADP+ and 1 UN/mL G6PDH; where needed to aid test compound solubility, 5 or 10 µL 40% 2-hydroxypropyl-β-cyclodextrin (HP-*β*-CD) was added and the volume of enzyme solution respectively adjusted to 84.6 or 79.6 µL accordingly. All reactions were shaken at 200 rpm on a Kuhner (AG Switzerland) 5 cm orbital shaker at 27°C for 18 hours and stopped by the addition of an equal volume of MeCN. Some reactions showing over-oxidation from the initial screening were rescreened as above except with sampling at shorter reaction times of 2 and 4 hours.

2. Scaled-up Biotransformation Reactions for milligram production

Two different methods were used for the scale-up reactions with these compounds: using PolyCYPs® enzyme prepared by a proprietary process from recombinant *E. coli* cells (final buffer concentration of 100 mM potassium phosphate pH 8 and 5 mM $MgCl_2$) or using pooled material from pre-lyophilised enzyme materials after being resuspended using cold H_2O. All reactions comprised of, in order of addition, parent compound (from a 25 mg/mL DMSO stock) at 0.1 mg/mL reaction concentration, PolyCYP® enzyme material, and cofactor stock solution (final reaction concentrations of 5 mM G6P, 1 mM NADP+, and 1 UN/mL G6PDH, prepared in 100 mM potassium phosphate pH 8 and 5 mM $MgCl_2$). Where needed, formulant (40% w/v HP-*β*-CD, prepared by dissolving HP-*β*-CD in 100 mM potassium phosphate and 5 mM $MgCl_2$ buffer at pH 8), was added to give final reaction concentrations of either 2% or 4% v/v HP-*β*-CD, accordingly reducing the volume of PolyCYP® enzyme

added. All reactions were mixed, then evenly distributed into 250 mL Erlenmeyer flasks as ca. 50 mL aliquots and incubated overnight at 27°C and 200 rpm (5 cm diameter orbit; Kuhner AG Switzerland). The reactions were checked by UPLC-MS, and stopped after either 4- or 17.5-18.5-hours reaction time by pooling and storage at -80°C. Chromatograms of selected scaled-up biotransformation reactions are shown in Figures S1-S11. Details of reaction conditions for scaled-up biotransformation reactions are shown in Table S1.

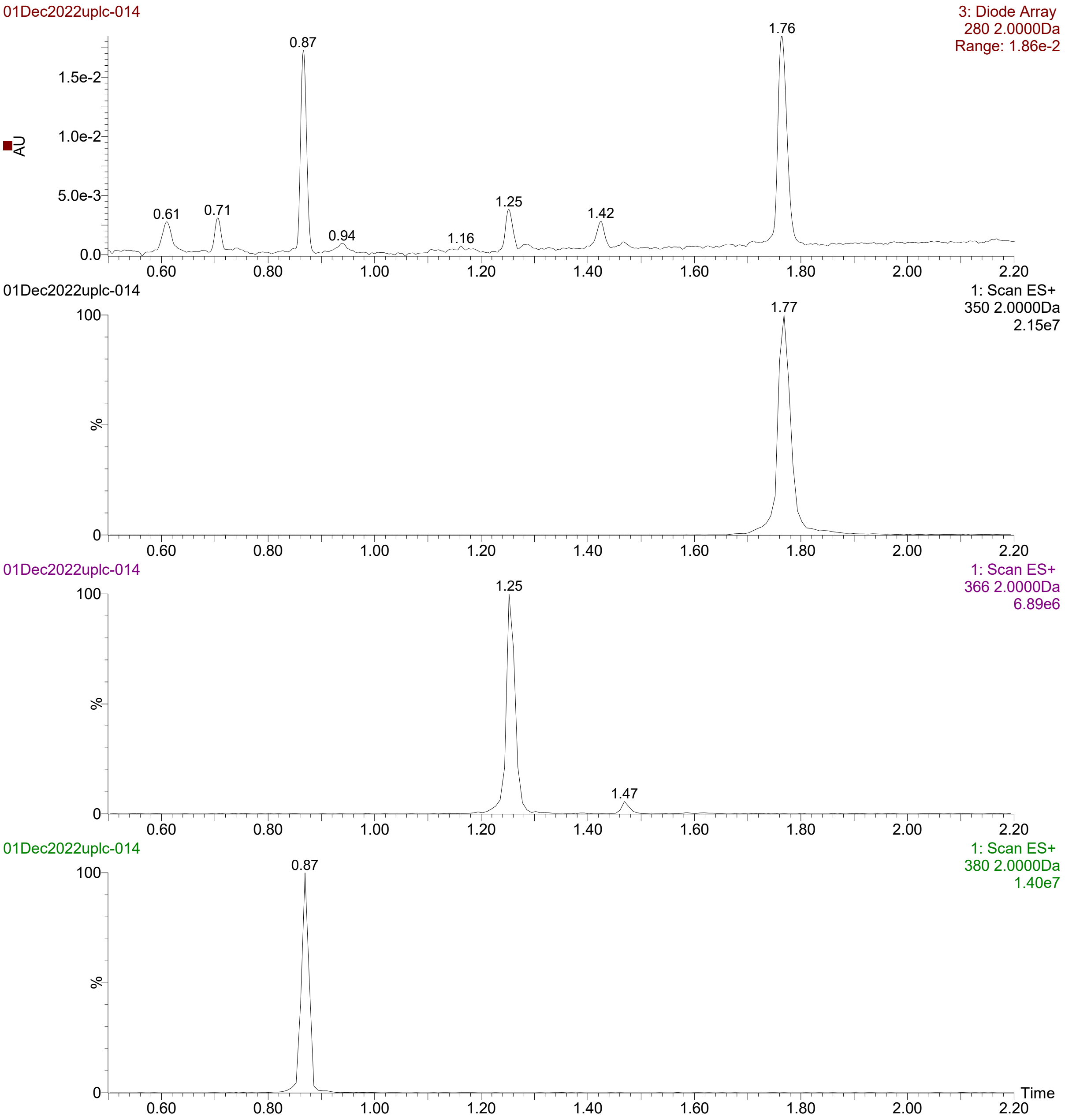

Figure S1 - UPLC-MS chromatograms of post-reaction extracts using PolyCYP194 cytochrome P450 and redox partners on **5.** Parent compound **5** (EIC: 350 m/z; 2nd trace) elutes at 1.77 minutes. Target +30Da metabolite **5a** (EIC: 380 m/z; 4th trace) elutes at 0.87 mins with a

conversion of 34.7%. Target +16Da metabolite **5b** (EIC: 366 m/z; 3rd trace) elutes at 1.25 mins with a conversion of 8.6%. In addition, another +16 Da product (EIC: 366 m/z; 3rd trace) was also detected at 1.47 mins, but was not purified due to low conversion.

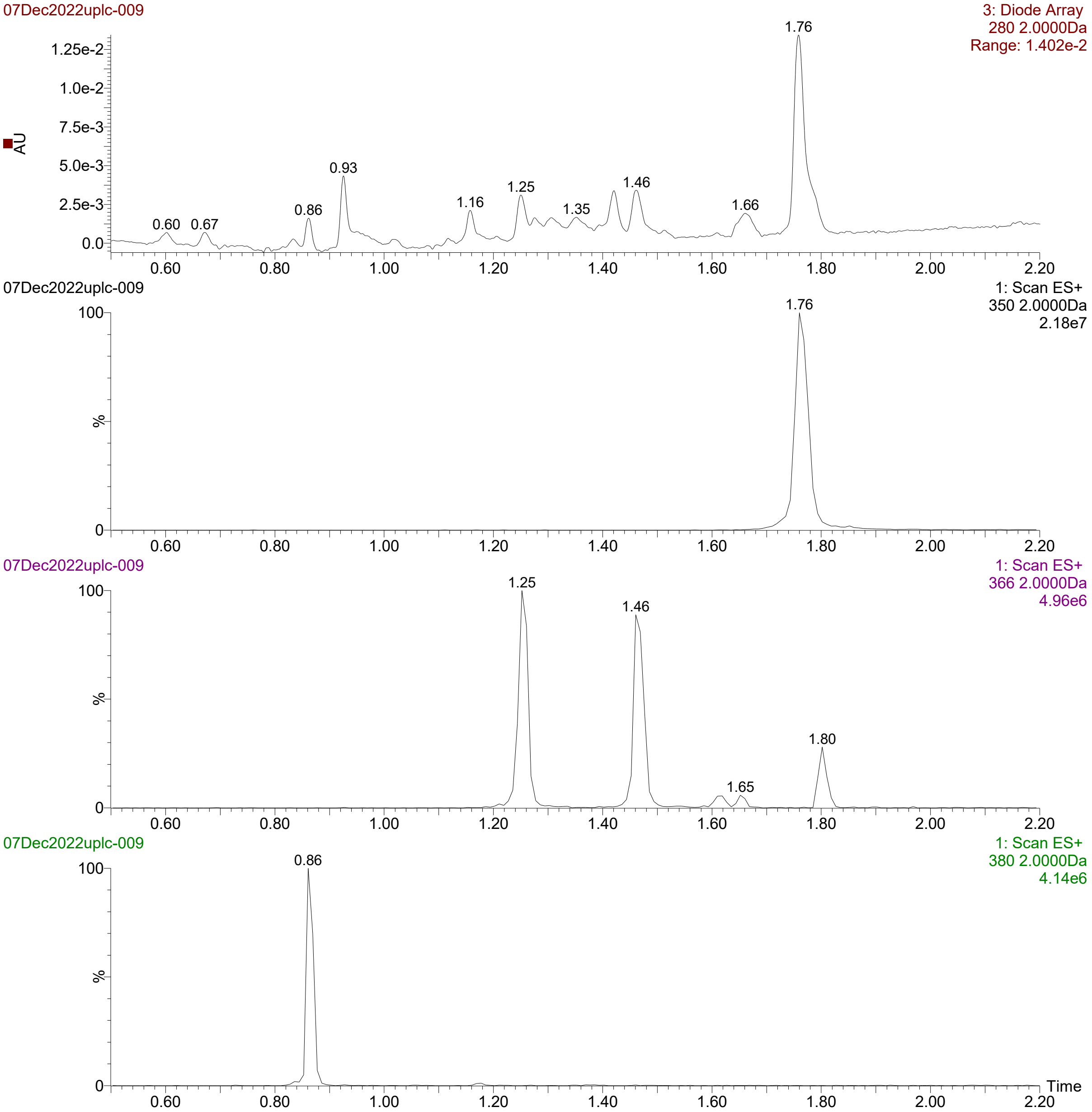

Figure S2 - UPLC-MS chromatograms of post-reaction extracts using PolyCYP488 cytochrome P450 and redox partners on **5.** Parent compound **5** (EIC: 350 m/z; 2nd trace) elutes at 1.76 minutes. Target +30Da metabolite **5c** (EIC: 380 m/z; 4th trace) elutes at 0.86 mins with a conversion of 5.3%. Target +16Da hydroxylated metabolites **5d** and **5e** (EIC: 366 m/z; 3rd trace) elute at 1.25 and 1.46 mins with conversions of 11.3% and 16.4%, respectively.

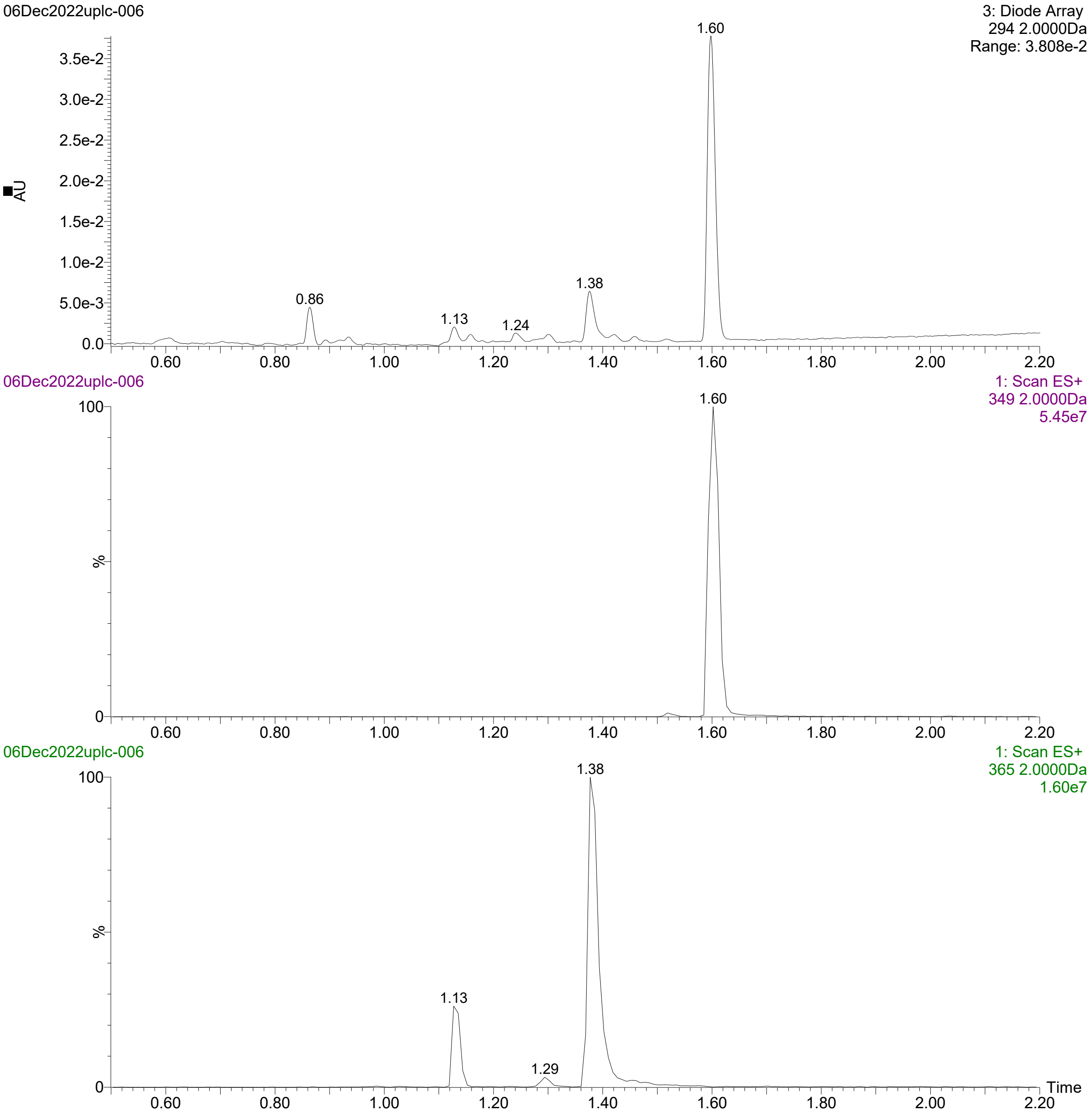

Figure S3 - UPLC-MS chromatograms of post-reaction extracts using PolyCYP152 cytochrome P450 and redox partners on **6.** Parent compound **6** (EIC: 349 m/z; 2nd trace) elutes at 1.60 minutes. Target +16Da hydroxylated metabolites **6a** and **6b** (EIC: 365 m/z; 3rd trace) elute at 1.13 and 1.38 mins with conversions of 4.5% and 14.4%, respectively. In addition, another +16 Da (EIC: 366 m/z; 3rd trace) was also detected at 1.29 mins, but was not purified due to the low conversion.

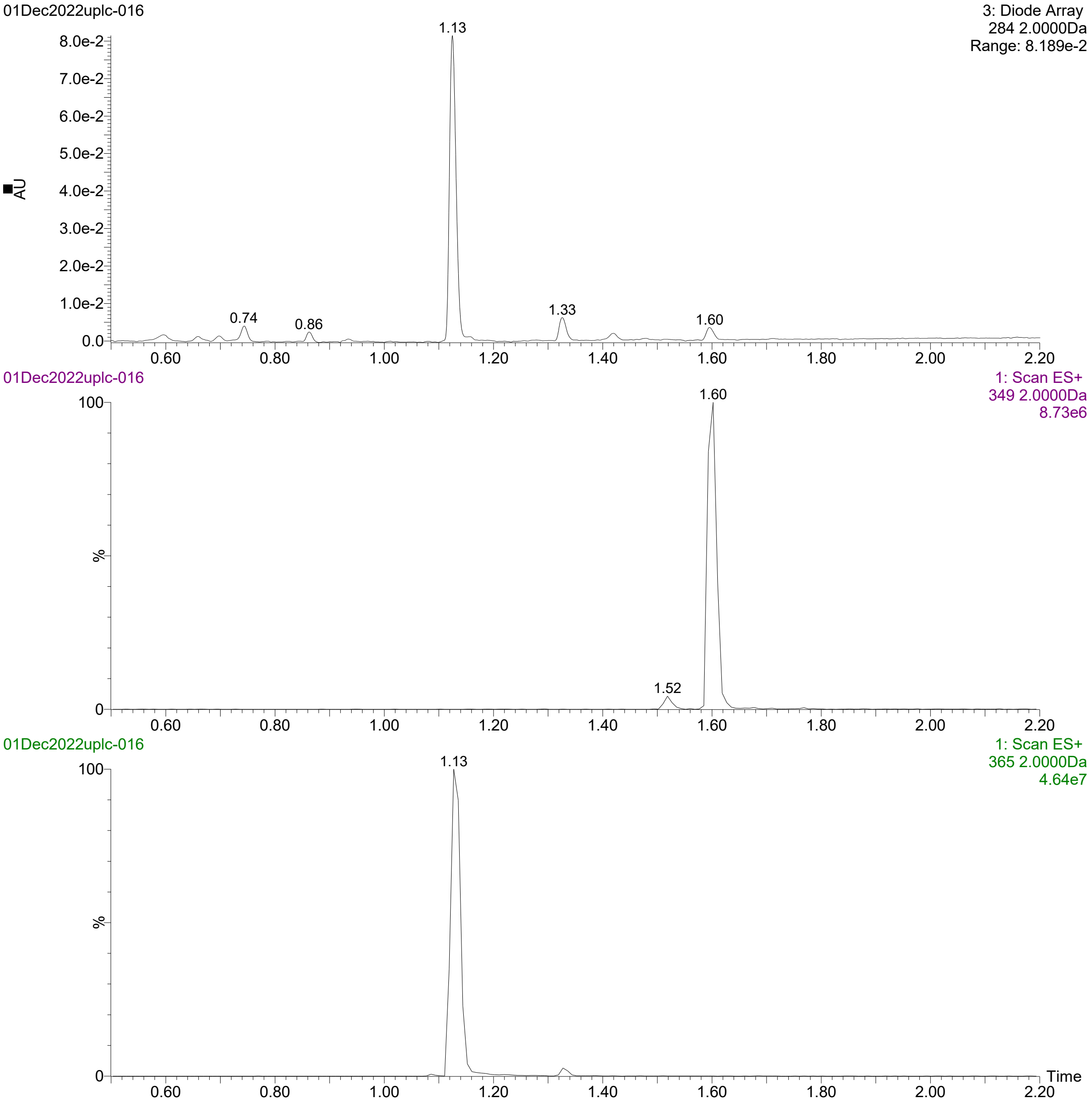

Figure S4 - UPLC-MS chromatograms of post-reaction extracts using PolyCYP194 cytochrome P450 and redox partners on **6.** Parent compound **6** (EIC: 349 m/z; 2nd trace) elutes at 1.60 minutes. Target +16Da hydroxylated metabolite **6c** (EIC: 365 m/z; 2nd trace) elutes at 1.13 mins with a conversion of 95.3%.

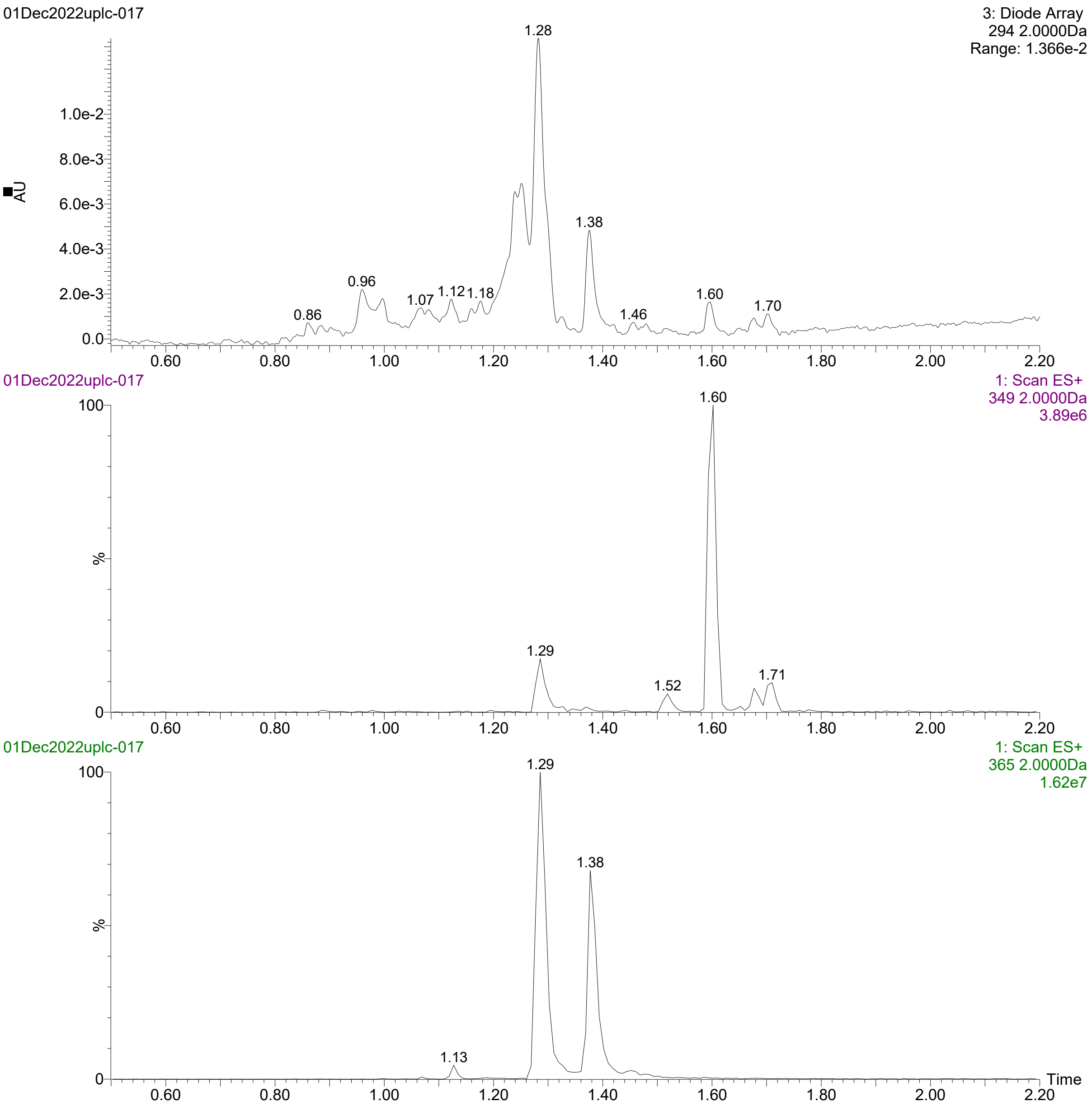

Figure S5 - UPLC-MS chromatograms of post-reaction extracts using PolyCYP483 cytochrome P450 and redox partners on **6.** Parent compound **6** (EIC: 349 m/z; 2nd trace) elutes at 1.60 minutes. Target +16Da hydroxylated metabolites **6d** and **6e** (EIC: 365 m/z; 3rd trace) elute at 1.29 and 1.38 mins with conversions of 72.6% and 18.5%, respectively. In addition, another +16 Da (EIC: 365 m/z; 3rd trace) was also detected at 1.13 mins, but was not purified due to the low conversion.

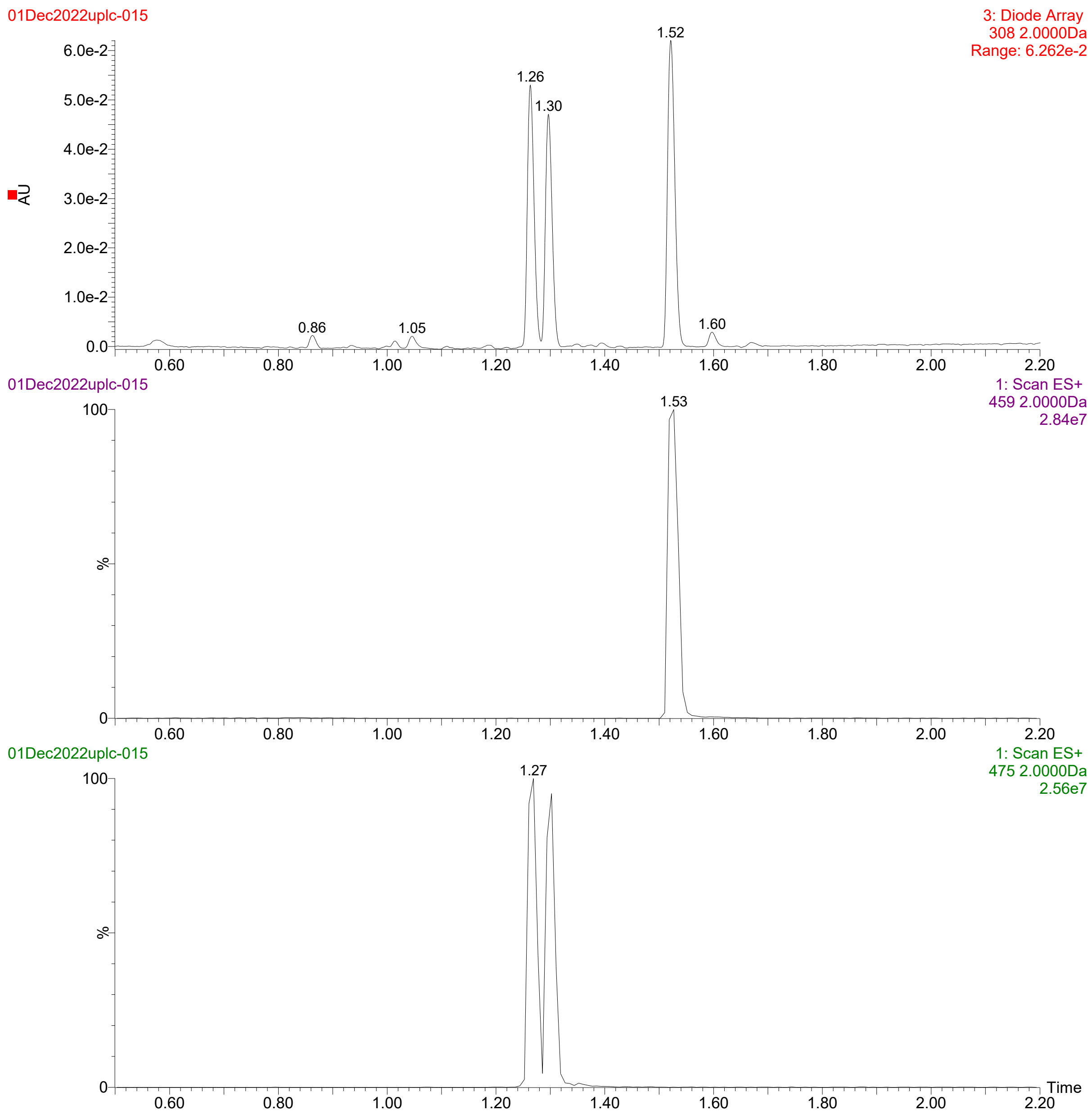

Figure S6 - UPLC-MS chromatograms of post-reaction extracts using PolyCYP194 cytochrome P450 and redox partners on **7.** Parent compound **7** (EIC: 459 m/z; 2nd trace) elutes at 1.53 minutes. Target +16Da hydroxylated metabolites **7a** and **7b** (EIC: 475 m/z; 3rd trace) elute at 1.27 and 1.30 mins with conversions of 31.9% and 28.2%, respectively.

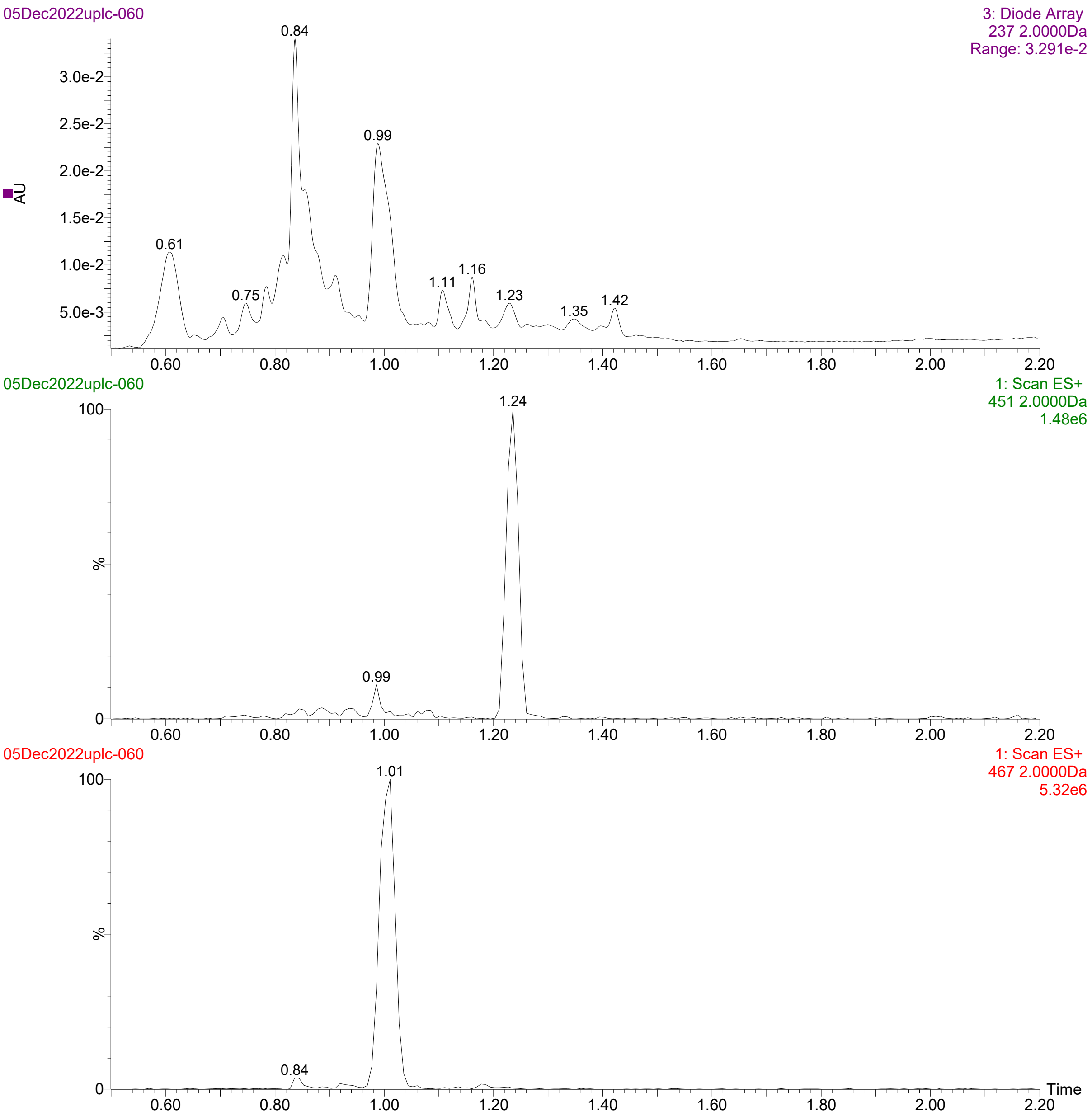

Figure S7 - UPLC-MS chromatograms of post-reaction extracts using PolyCYP166 cytochrome P450 and redox partners on **8.** Parent compound **8** (EIC: 451 m/z; 2nd trace) elutes at 1.24 minutes. Target +16Da hydroxylated metabolite **8a** (EIC: 467 m/z; 3rd trace) elutes at 1.01 minutes with a conversion of 92.2%.

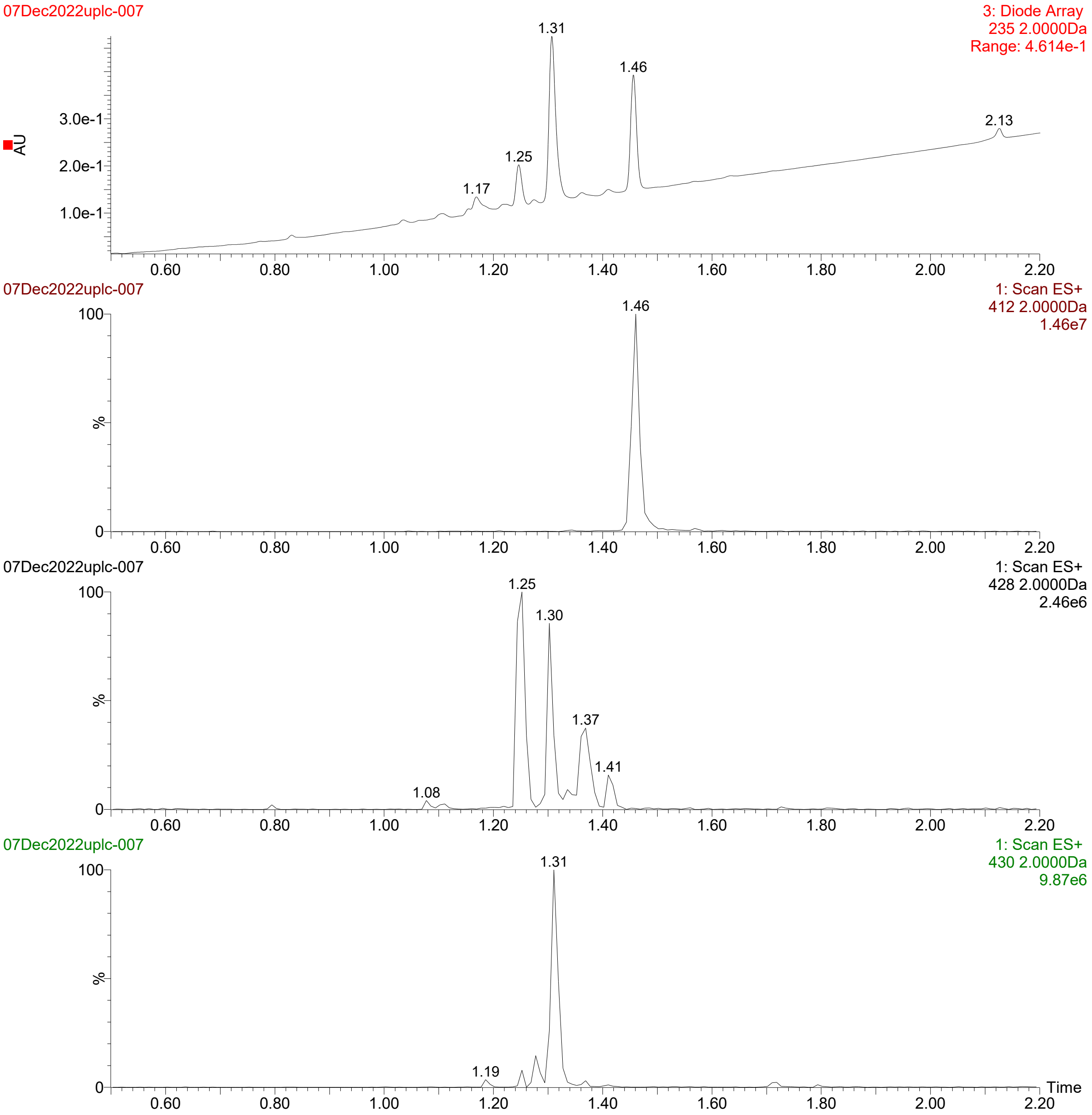

Figure S8 - UPLC-MS chromatograms of post-reaction extracts using PolyCYP166 cytochrome P450 and redox partners on **9.** Parent compound **9** (EIC: 412 m/z; 2nd trace) elutes at 1.46 minutes. Target +16Da hydroxylated metabolites **9a** and **9b** (EIC: 428 m/z; 3rd trace) co-elute at 1.25 mins with a combined conversion of 11.7%. Target +18Da metabolite **9c** (EIC: 430 m/z; 4th trace) elutes at 1.31 mins with a conversion of 53.1%. In addition, two other +16 Da products (EIC: 428 m/z; 3rd trace) were also detected at 1.37 and 1.41 mins, but were not purified due to the low conversions.

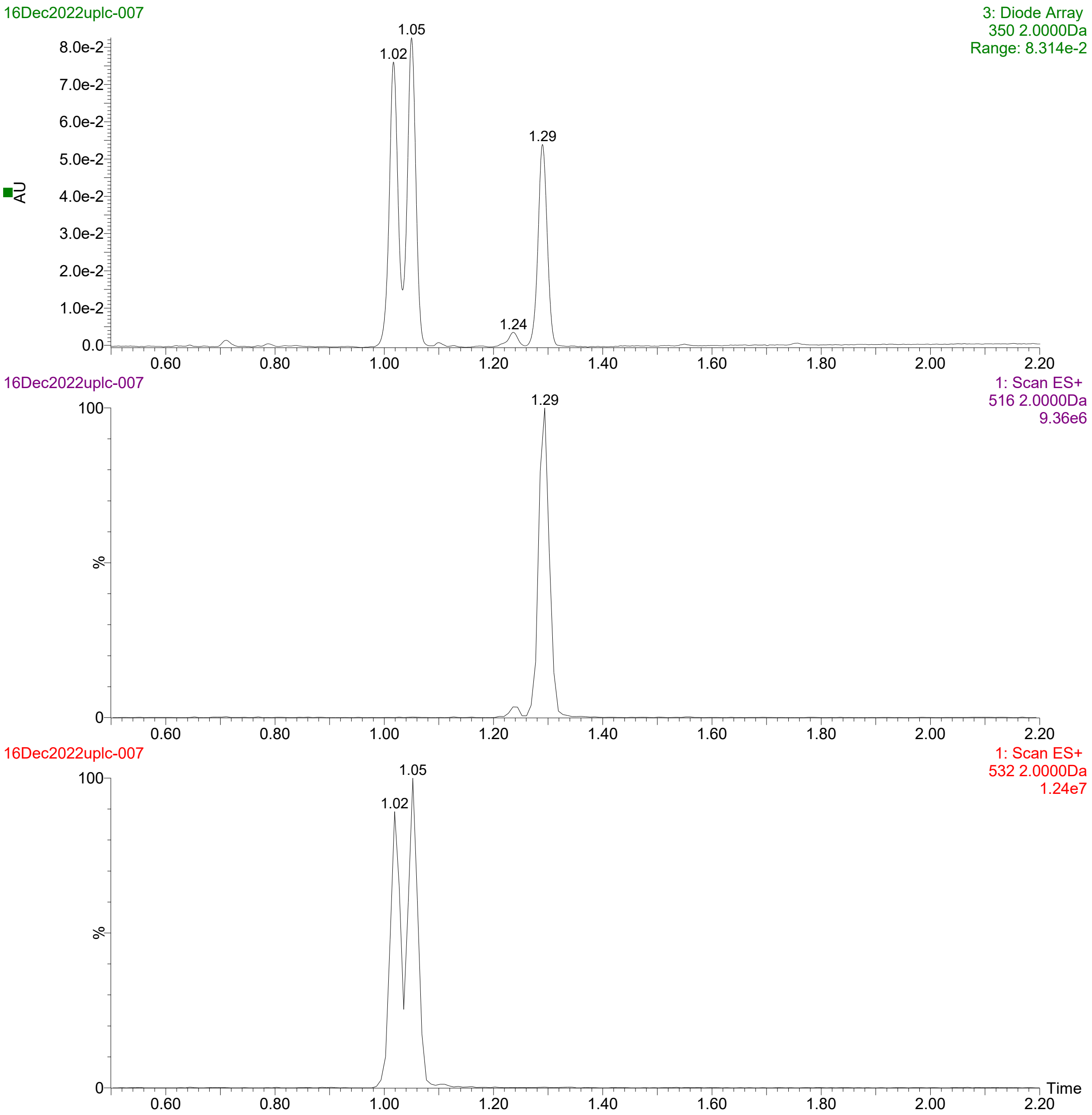

Figure S9 - UPLC-MS chromatograms of post-reaction extracts using PolyCYP194 cytochrome P450 and redox partners on **10.** Parent compound **10** (EIC: 516 m/z; 2nd trace) elutes at 1.29 minutes. Target +16Da hydroxylated metabolites **10a** and **10b** (EIC: 532 m/z; 3rd trace) elute at 1.02 and 1.05 mins with conversions of 35.5% and 37.8%, respectively.

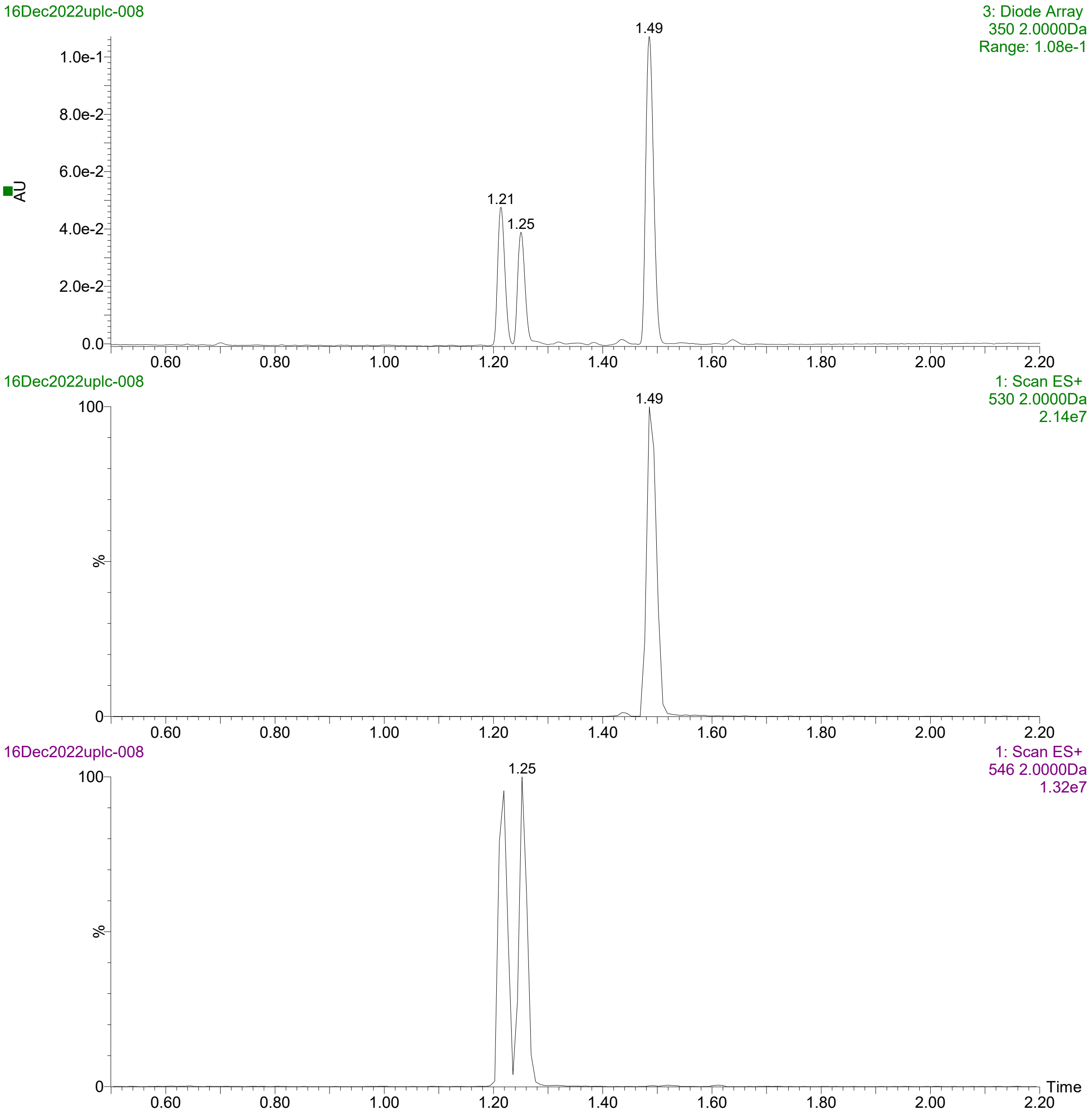

Figure S10 - UPLC-MS chromatograms of post-reaction extracts using PolyCYP194 cytochrome P450 and redox partners on **11.** Parent compound **11** (EIC: 530 m/z; 2nd trace) elutes at 1.49 minutes. Target +16Da hydroxylated metabolites **11a** and **11b** (EIC: 546 m/z; 3rd trace) elute at 1.21 and 1.25 mins with conversions of 23.4% and 20.0%, respectively.

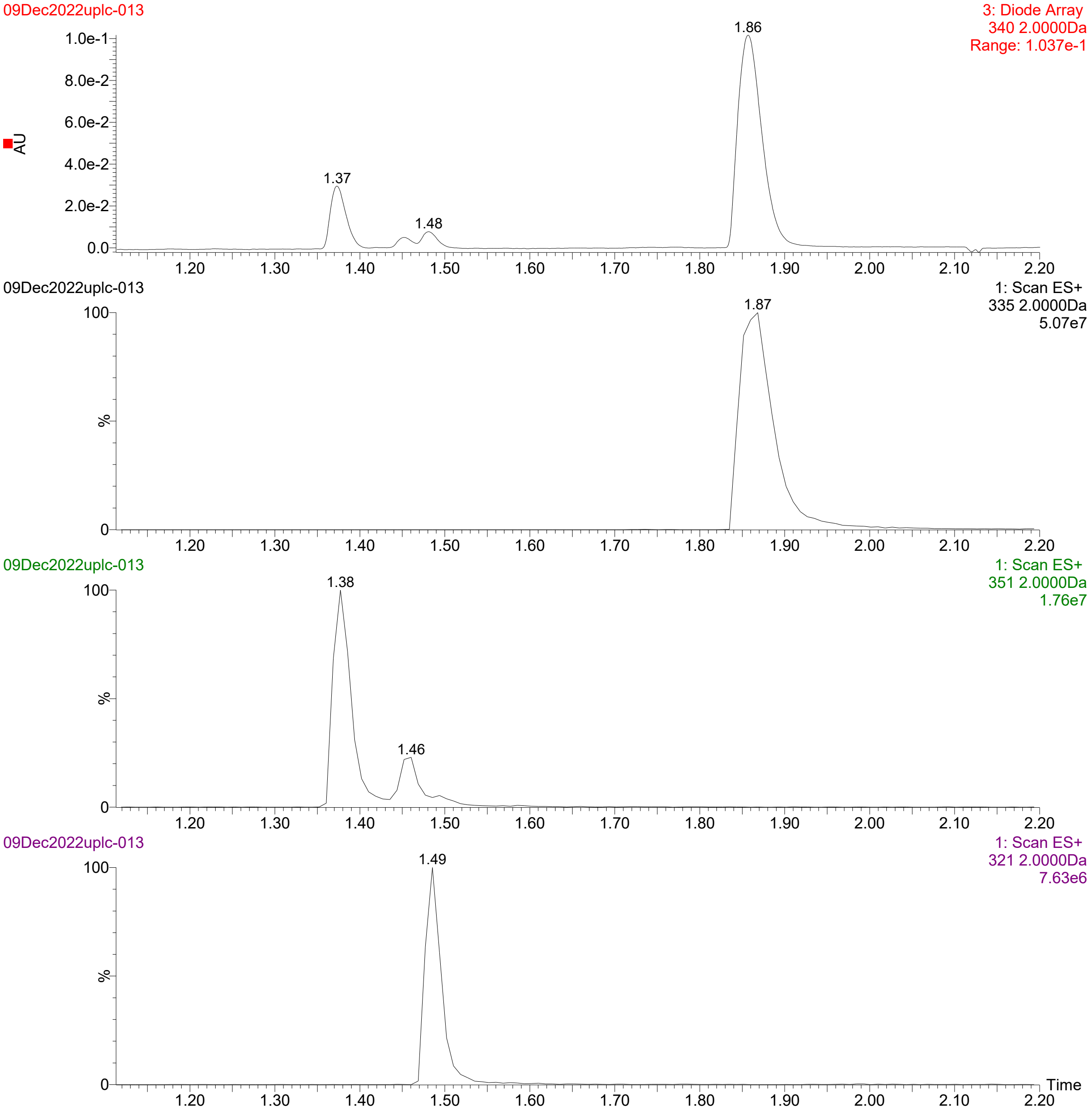

Figure S11 - UPLC-MS chromatograms of post-reaction extracts using PolyCYP168 cytochrome P450 and redox partners on **12.** Parent compound **12** (EIC: 335 m/z; 2nd trace) elutes at 1.87 minutes. Target +16Da hydroxylated metabolites **12a** and **12b** (EIC: 351 m/z; 3rd trace) elute at 1.38 and 1.46 mins with conversions of 15.0% and 2.3%, respectively. Target -14Da desmethylated metabolite **12c** (EIC: 321 m/z; 4th trace) elutes at 1.49 mins with a conversion of 4.0%.

Compound	PolyCYP® isoform	Scale-up approach	Parent dosed (mg)	Reaction Volume (ml)	Formulation in reaction (% CD)	Reaction time (hrs)	UPLC-MS analysis condition
**5**	194	Lyophilised vials	10.00	100	None	17.5	Base
	488	Lyophilised vials	25.75	256	None	17.5	
**6**	152	Lyophilised vials	26.00	259	2	18.0	Acid
	194	Lyophilised vials	3.00	31	2	17.5	
	483	Lyophilised vials	7.00	71	2	17.5	
**7**	194	Lyophilised vials	10.75	106	4	17.5	Base
**8**	166	Lyophilised vials	10.00	100	None	4.0	Base
**9**	166	Lyophilised vials	26.00	259	2	17.5	Acid
**10**	194	Lyophilised vials	12.25	122	None	18.0	Base
**11**	194	Lyophilised vials	10.75	106	2	18.0	Base
**12**	168	Pellet processed	17.35	201	None	18.0	Base

Table S1: Reaction conditions for scaled up biotransformation reactions

3. Generic purification of scaled-up reactions

The following procedure was generally applied to purify the products from scaled-up reactions. The harvested material was defrosted at 40°C. Ammonium sulphate was dissolved in the harvested material at 100 g/L, which was extracted twice with an equal volume of MeCN. Pellets were centrifuged at 4,000 x*g* for 15 minutes at room temperature and the upper MeCN phases were combined.

The purification of target metabolites were performed using 1-2 fractionations over a Waters XSelect CSH C_{18} (5µM, 30mm i.d. x 100mm), Waters XBridge Phenyl (5 µM, 30 mm i.d. x 100mm), Waters Atlantis T3 (5 µM, 19mm i.d. x 100mm) or Waters SunFire C18 (5 µM, 30 mm i.d. x 100mm) columns with an appropriate guard column at room temperature (ca. 20°C). The elution program used H_2O:MeCN: either 2% formic acid or 200 mM ammonium

bicarbonate in water starting at 85:10:5 at 10 mL/min and increasing the flow rate to 40 mL/min over 1 minute (and continued at this flow thereafter). The elution program then changed linearly to 78:17:5 over 20 minutes followed by washing and re-equilibration. The organic extract fractions containing target metabolites were pooled and lyophilised under vacuum to provide a solid powder with LC-UV and LC-ELSD purities of both mostly >90%. (where the purities dropped below 90%, it was still possible to identify the main product). The results are shown in Table S2.

Compound	PolyCYP isoform	Reaction volume (ml)	Parent dosed (mg)	Product	Amount produced (mg)	Isolated Yield (%)	Purity (%)	
							UPLC-UV	UPLC-ELSD
**5**	194	100	10	**5a**	1.95	19.5	98.0	>99.0
				**5b**	0.94	9.4	94.6	>96.0
	488	256	25.75	**5c**	1.41	5.5	96.6	>99.0
				**5d**	0.68	2.6	95.9	>99.0
				**5e**	1.45	5.6	91.9	>99.0
**6**	152	259	26	**6a**	0.66	2.5	>99.0	98.2
				**6b**	2.68	10.3	>99.0	>99.0
	194	31	3	**6c**	2.5	83.3	95.3	>99.0
	483	71	7	**6d**	2.36	33.7	88.8	98.7
				**6e**	0.59	8.4	89.9	>99.0
**7**	194	106	10.75	**7a**	2.74	25.5	96.7	>99.0
				**7b**	2.57	23.9	94.6	>99.0
**8**	166	100	10	**8a**	0.73	7.3	97.4	>99.0
**9**	166	259	26	**9a**	2.91	11.2	83.2	83.9
				**9b**	1.15	4.4	91.0	99.5
				**9c**	11.16	42.9	96.4	>99.0
**10**	194	122	12.25	**10a**	0.97	7.9	81.1	99.5
				**10b**	1.41	11.5	88.0	99.7
**11**	194	106	10.75	**11a**	1.2	11.2	>99.0	>99.0
				**11b**	0.98	9.1	>99.0	>99.0
**12**	168	201	17.35	**12a**	2.95	17	98.6	>99.0
				**12b**	1.04	6	93.2	>99.0
				**12c**	0.74	4.3	97.0	97.9

Table S2: Isolated products from scaled up biotransformation reactions

Compound Characterisation

Compound **5a**

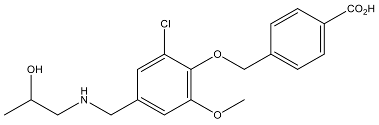

δ_H (500 MHz; DMSO-*d*6) 1.06 (3 H, d, J = 6.1), 2.49, m, 3.76 (3 H, m), 3.84 (3 H, s), 5.03 (2 H, s), 7.07 (1 H, s), 7.12 (1H, s), 7.56 (2 H, d, *J* = 7.9), 7.95 (2 H, d, *J* = 7.9). δ_C (125 MHz; DMSO-*d*6) 21.2, 51.2, 55.4, 55.6, 55.8, 64.2, 73.4, 111.7, 120.5, 120.9, 127.5, 129.0, 131.9, 141.2, 142.2, 153.3, 167.7. HRMS(ESI) Expected m/z 380.12 [M+H]+ Found m/z 380.126 [M+H]+

Compound **5b**

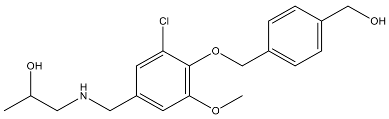

δ_H (500 MHz; DMSO-*d*6) 1.05 (3 H, d, *J* = 5.8), 2.48 (m), 3.75 (3H, m), 3.85 (3H, s), 4.50 (2 H, s), 4.95 (2 H, s), 7.03 (1 H, br. s), 7.08 (1 H, br. s) 7.31 (2 H, d, *J* = 7.9), 7.41 (2 H, d, *J* = 8.1) δ_C (125 MHz; DMSO-*d*6) 21.3, 51.4, 55.5, 55.9, 62.7, 64.5, 74.0, 111.8, 120.9, 126.3 128.1, 135.3, 142.4, 142.5, 153.9. HRMS(ESI) Expected m/z 366.14 [M+H]+ Found m/z 366.147 [M+H]+

Compound **5e**

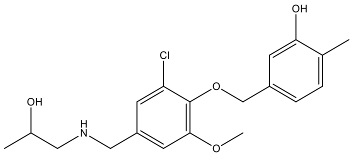

δ_H (500 MHz; DMSO-*d*6) 1.05 (3 H, br. d), 2.11 (3 H, s), 3.73 (1 H, br. s), 3.84 (3 H, s), 4.82 (2 H, s), 6.76 (1 H, d, *J* = 7.5), 6.95 (1 H, s), 7.03 (2 H, m), 7.07 (1 H, br. s) δ_C (125 MHz; DMSO-*d*6) 15.6, 21.2, 56.6, 64.5, 73.6 111.3, 114.4, 118.4, 120.0, 123.1, 130.0 134.9, 141.6, 153.0, 154.6. HRMS(ESI) Expected m/z 366.14 [M+H]+ Found m/z 366.147 [M+H]+

Compound **6b**

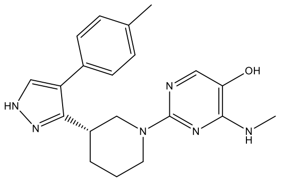

^{1}H NMR (500 MHz, DMSO) δ 8.35 (s, 1H), 7.67 (s, 1H), 7.37 – 7.31 (m, 3H), 7.16 (d, *J* = 7.9 Hz, 2H), 6.44 (q, *J* = 4.7 Hz, 1H), 4.70 (d, *J* = 12.9 Hz, 1H), 4.59 (d, *J* = 12.9 Hz, 1H), 2.91 (s, 1H), 2.83 (t, *J* = 11.9 Hz, 1H), 2.77 – 2.65 (m, 4H), 2.30 (s, 3H), 1.88 (td, *J* = 10.4, 7.2 Hz, 2H), 1.66

(dt, *J* = 13.2, 3.0 Hz, 1H), 1.44 (ddt, *J* = 12.6, 8.4, 4.5 Hz, 1H). ^{13}C NMR (126 MHz, DMSO) δ 136.9, 129.6, 128.1, 40.6, 40.4, 40.3, 40.1, 39.9, 27.14, 21.1.

Compound **6c**

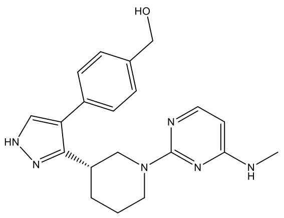

δ_H (500 MHz; DMSO-*d*6) 1.41 (1 H, m), 1.68 (1 H, d, *J* = 13.1 Hz), 1.91 (2 H, m), 2.69 (3 H, d, *J* = 4.6 Hz), 2.75 (1H, td, *J* = 12.8, 2.2 Hz), 2.90 (1 H, m), 4.47 (2 H, s), 4.74 (1 H, d, *J* = 12.5 Hz), 4.87 (1 H, m), 5.71 (1 H, d, *J* = 5.8 Hz), 6.87 (1 H, br. s), 7.29 (2 H, d, *J* = 8.1 Hz), 7.40 (2 H, d, *J* = 8.1 Hz) 7.70 (1 H, br. s) δ_C (125 MHz; DMSO-*d*6) 24.7, 26.4, 30.5, 33.7, 43.4, 49.2, 62.7, 94.9, 118.5, 126.7, 127.3, 131.9, 140.0, 154.1, 163.1. HRMS(ESI) Expected m/z 365.20 [M+H]+ Found m/z 365.209 [M+H]+

Compound **6d**

Structure unconfirmed but based on likely position of hydroxylation

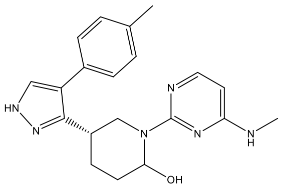

^{1}H NMR (500 MHz, DMSO) δ 8.25 (s, 1H), 7.17 (dd, *J* = 8.2, 2.4 Hz, 3H), 5.69 (d, *J* = 5.7 Hz, 1H), 5.65 – 5.61 (m, 1H), 5.57 (t, *J* = 4.8 Hz, 0H), 3.82 – 3.60 (m, 1H), 2.73 – 2.67 (m, 3H), 2.30 (s, 4H), 2.08 – 1.74 (m, 3H). δ_C (500 MHz; DMSO-*d*6) 16.5, 21.2, 26.7, 27.4, 33.8, 40.2, 43.1, 129.8, 138.2, 162.9. HRMS(ESI) Expected m/z 365.20 [M+H]+ Found m/z 365.208 [M+H]+

The NMR spectra of this metabolite show the presence of at least two components presumably due to hemi-aminal ring opening in equilibrium.

Compound **6e**

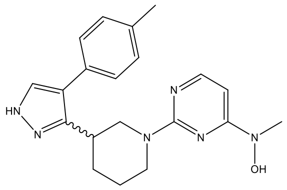

^{1}H NMR (700 MHz, MeOD) δ 8.55 (s, 1H), 7.68 (d, *J* = 21.7 Hz, 1H), 7.33 (dt, *J* = 7.1, 5.1 Hz, 2H), 7.23 (d, *J* = 7.7 Hz, 1H), 7.16 – 7.04 (m, 1H), 4.62 (d, *J* = 8.9 Hz, 1H), 3.22 (d, *J* = 9.4 Hz, 1H), 3.16 (td, *J* = 13.1, 2.9 Hz, 1H), 2.94 (s, 1H), 2.90 (s, 2H), 2.39 (s, 2H), 2.27 (d, *J* = 32.6 Hz,

1H), 2.15 – 2.10 (m, 1H), 2.08 – 1.86 (m, 1H), 1.68 (tdd, *J* = 13.0, 8.7, 3.9 Hz, 1H). δ_C (176 MHz, MeOD) 19.6, 24.3, 26.3, 30.1, 33.8, 45.1, 49.8, 120.4,128.9, 136.1, 150.4, 157.6.

Compound **7a**

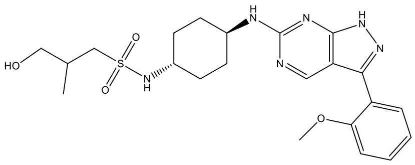

δ_H (500 MHz; DMSO-*d*6) 1.01 (3 H, d, *J* = 6.9), 1.36 (4 H, m), 1.95 – 1.97 (4 H, m), 2.03 (1 H, m), 2.74 (1 H, dd, *J* = 14.1, 8.5), 3.07 (1 H, br. s), 3.16 (1 H, dd, *J* = 14.2, 4.0), 3.27 (1 H, m), 3.35 (1 H, m), 3.67 (1 H, br. s), 3.85 (3 H, s), 4.72 (1 H, t, *J* = 5.3), 7.04 (1 H, td, *J* = 7.5, 0.7), 7.06 (1 H, d, *J* = 5.2), 7.18 (1 H, d, *J* = 8.4), 7.24 (1 H, br. s), 7.42 (1 H, td, *J* = 7.8, 1.6), 7.65 (1 H, dd, *J* = 7.6, 1.6), 8.74 (1 H, br. s), 13.09 (1 H, br. s). δ_C (125 MHz; DMSO-*d*6) 16.1, 30.3, 32.0, 32.6, 48.4, 51.2, 55.0, 55.4, 64.8, 111.4, 120.3, 121.6, 129.5, 129.8, 141.6, 154.9, 156.2. HRMS(ESI) Expected m/z 475.20 [M+H]+ Found m/z 475.213 [M+H]+

Compound **7b**

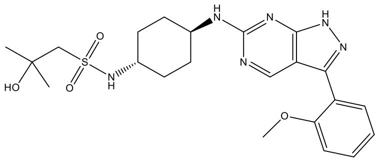

δ_H (500 MHz; DMSO-*d*6) 1.30 (6 H, s), 1.35 (4 H, m), 1.96 (4H, m), 3.10 (1 H, br. s), 3.15 (2 H, s), 3.68 (1 H, br. s), 3.85 (3 H, s), 4.71 (1 H, s), 6.95 (1 H, d, *J* = 6.5 Hz), 7.04 (1 H, t, *J* = 7.5 Hz), 7.18 (1 H, d, *J* = 8.2 Hz), 7.25 (1 H, br. s), 7.42 (1 H, td, *J* = 7.8, 1.7 Hz), 7.65 (1 H, dd, *J* = 7.6, 1.6 Hz), 8.74 (1 H, br. s), 13.09 (1 H, br. s). δ_C (125 MHz; DMSO-*d*6) 28.9, 30.3, 32.6, 48.4, 51.2, 55.0, 63.9, 68.0, 111.4, 120.3, 121.6, 129.5, 129.8, 141.6, 154.9, 156.2. HRMS(ESI) Expected m/z 475.20 [M+H]+ Found m/z 475.213 [M+H]+

Compound **8a**

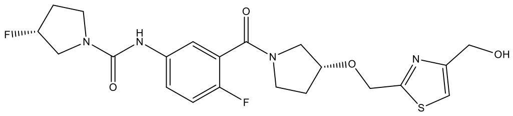

^{1}H NMR (700 MHz, DMSO) δ 2.05 (3 H, m), 2.09 (3 H, m), 2.16 (3H, m), 3.52 (4H, m), 3.61 (8 H, m), 3.66 (4 H, m), 4.26 (1 H, m), 4.34 (1 H, m), 4.50 (2 H, s), 4.54 (2 H, s), 4.69 (2 H, d, *J* = 13.8 Hz), 4.74 (2 H, d, *J* = 13.8 Hz), 4.80 (2 H, s x 2); 5.35 (2 H, d, *J* = 53.0 Hz), 7.16 (2 H, td, *J* = 9.2, 3.1 Hz), 7.35 (1 H, s), 7.39 (1 H, s), 7.55 (2 H, m), 7.58 (2 H, m), 8.41 (2 H, s x 2). 13 C NMR (175 MHz, DMSO-*d6*) δ 29.1, 30.7, 31.1, 43.0, 50.8, 51.9, 59.1, 66.7, 76.9, 77.8, 92.5, 114.3, 115.3, 118.6, 121.7, 124.2, 136.3, 152.1, 153.4, 157.1, 163.7, 167.6. HRMS(ESI) Expected m/z 465.15 [M+H]+ Found m/z 467.156 [M+H]+

The NMR spectra of this metabolite show the presence of two components, believed to be conformers, in an approximate ratio of 1:1. The majority of the proton signals are overlapped but are resolved, for example for the thiazole methine singlet signals at 7.35 and 7.39 ppm and the two oxymethylene singlets at 4.50 and 4.54 ppm generated by the metabolism.

Compound **9a**

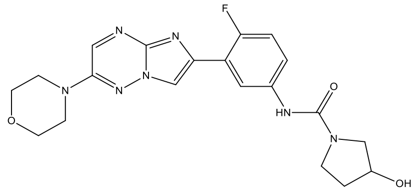

^{1}H NMR (500 MHz, DMSO) 1.81 – 1.98 (2 H, m), 3.46 (3 H, m), 3.55 (4 H, t, *J* = 4.8 Hz), 3.76 (4 H, t, *J* = 4.8 Hz), 4.30 (1 H, m), 4.98 (1 H, br s), 7.17 (1 H, dd, *J* = 10.8, 9.1 Hz), 7.61 (1 H, ddd, *J* = 9.0, 4.5, 2.9 Hz), 8.11 (1 H, d, *J* = 4.1 Hz), 8.33 (1H, s), 8.34 (1 H, dd, *J* = 7.0, 2.7 Hz), 8.48 (1 H, br s), 8.69 (1 H, s). ^{13}C NMR (125 MHz, DMSO)33.9, 43.6, 45.8, 54.1, 65.5, 68.6, 113.3, 115.1, 118.3, 119.8, 137.6, 150.3, 153.6. HRMS(ESI) Expected m/z 428.18 [M+H]+ Found m/z 428.184 [M+H]+

Compound **9b**

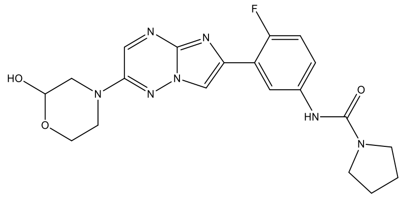

^{1}H NMR (500 MHz, DMSO) δ 1.86 (4 H, m), 3.19 (1 H, dd, *J* = 12.6, 6.3 Hz), 3.38 (t, *J* = 6.6 Hz), 3.64 (1 H, m), 3.69 (1 H, m), 3.83 (1 H, dd, *J* = 12.8, 2.3 Hz), 4.04 (1 H, ddd, *J* = 11.5, 4.5, 3.7 Hz), 4.89 (1 H, dd, *J* = 6.1, 2.3), 6.88 (1 H, br s), 7.17 (1 H, dd, *J* = 10.9, 9.1 Hz), 7.61 (1 H, ddd, *J* = 9.0, 4.5, 2.8 Hz), 8.11 (1 H, d, *J* = 4.0), 8.33 (1 H, s), 8.34 (1 H, dd, *J* = 7.0, 2.7 Hz), 8.70(1 H, s), ^{13}C NMR (125 MHz, DMSO) δ 24.7, 44.0, 45.3, 49.7, 60.3, 90.2, 118.4, 119.8, 136.4, 137.9, 139.0, 149.9, 153.2. HRMS(ESI) Expected m/z 428.18 [M+H]+ Found m/z 428.185 [M+H]+

Compound **9c**

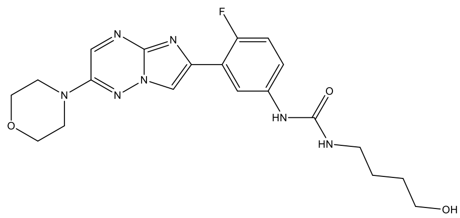

^{1}H NMR (500 MHz, DMSO) δ 1.46 (4 H, m), 3.10 (2 H, q, *J* = 5.8 Hz), 3.41 (2 H, m), 3.56 (4 H, t, *J* = 4.8 Hz), 3.77 (4 H, t, *J* = 4.8 Hz), 4.42 (1 H, br s), 6.23 (1 H, t, *J* = 5.6 Hz), 7.16 (1 H, dd, *J* = 10.8, 9.0 Hz), 7.43 (1 H, ddd, *J* = 8.7, 4.2, 3.2 Hz), 8.11 (1 H, d, *J* = 4.1 Hz), 8.22 (1 H, dd, *J* = 6.8, 2.8 Hz), 8.69 (2 H, s). ^{13}C NMR (126 MHz, DMSO) δ 26.5, 29.9, 38.7, 45.3, 60.5, 65.4, 113.8, 115.5, 116.3, 118.4, 137.0, 138.0, 139.2, 150.4, 153.2, 155.2. HRMS(ESI) Expected m/z 430.19 [M+H]+ Found m/z 430.200 [M+H]+

Compound **10a**

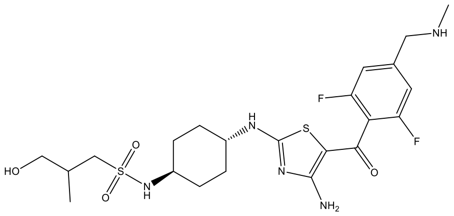

δ_H (500 MHz; DMSO-*d*6) 0.99 (3 H, d, *J* = 6.9 Hz), 1.28 (4 H, m), 1.91 (4 H, br. s), 2.01 (1 H, m), 2.27 (1 H, br. s), 2.72 (1 H, dd, *J* = 14.1, 8.5 Hz), 2.81 (1 H, br. s), 3.05 (1 H, br. s), 3.14 (1 H, dd, *J* = 13.9, 3.8 Hz), 3.25 (1 H, dd, *J* = 10.4, 6.6 Hz), 3.32 (1 H, dd, *J* = 10.5, 5.7 Hz), 4.42 (1 H, s), 6.98 (1 H, m), 7.05 (1 H, d, *J* = 7.3 Hz), 7.10 (1 H, d, *J* = 7.1 Hz), 8.05 (2 H, br. s). δ_C (125 MHz; DMSO-*d*6)16.5, 30.5, 31.8, 32.0, 51.0, 55.5, 64.9, 110.0, 110.5, 157.9. HRMS(ESI) Expected m/z 532.18 [M+H]+ Found m/z 532.186 [M+H]+

The spectra show the presence of two sets of signals in places, suggesting the presence of conformers.

Compound **10b**

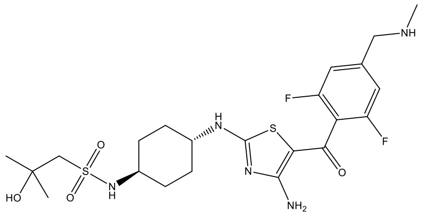

^{1}H NMR (500 MHz, DMSO) δ 8.40 (s, 2H), 8.05 (s, 2H), 7.22 – 6.87 (m, 2H), 4.41 (br s, 1H), 3.68 (s, 1H), 3.13 (s, 2H), 2.79 (br s, 1H), 2.26 (br s, 1H), 1.91 (br s, 3H), 1.28 (s, 6H). ^{13}C NMR (126 MHz, DMSO) δ 68.6, 51.5, 29.7. HRMS(ESI) Expected m/z 532.18 [M+H]+ Found m/z 532.186 [M+H]+

Compound **11a**

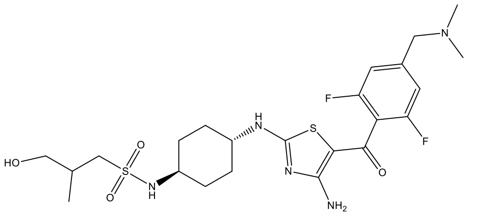

^{1}H NMR (500 MHz, DMSO) δ 0.99 (3 H, d, *J* = 6.8 Hz,), 1.28 (4 H, m), 1.90 (4 H, m), 2.03 (1 H, m), 2.16 (6 H, s), 2.72 (1 H, dd, *J* = 14.2, 8.4 Hz), 3.13 (1 H, dd, *J* = 14.1, 4.0 Hz), 3.32 (1 H, m), 3.43 (2 H, s), 4.70 (1 H, t, *J* = 5.4 Hz), 7.06 (2 H, d, *J* = 8.7 Hz), 8.05 (2 H, br s). ^{13}C NMR (126 MHz, DMSO) δ 16.4, 30.5, 32.1, 44.9, 51.0, 55.4, 62.0, 65.0, 111.7, 117.3, 143.7, 157.9. HRMS(ESI) Expected m/z 546.19 [M+H]+ Found m/z 546.200 [M+H]+

Compound **11b**

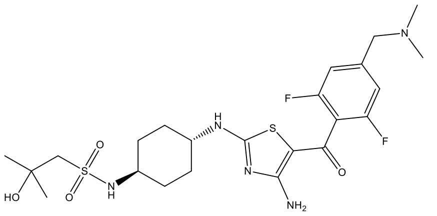

^{1}H NMR (700 MHz, DMSO) δ1.24 – 1.34 (10 H, m), 1.92 (4 H, m), 2.16 (6 H, s), 3.09 (1 H, m), 3.13 (2 H, s), 3.43 (2 H, s), 4.67 (1 H, br s), 6.91 (1 H, br s), 7.06 (2 H, d, *J* = 8.0 Hz), 8.03 (2 H, br s) . ^{13}C NMR (126 MHz, MeOD) δ 171.89, 167.10, 159.93, 159.86, 157.94, 157.88, 112.84, 112.66, 68.73, 63.65, 61.35, 51.61, 43.25, 43.23, 32.37, 30.65, 28.14. HRMS(ESI) Expected m/z 546.19 [M+H]+ Found m/z 546.200 [M+H]+

Compound **12a**

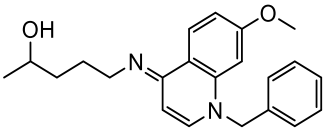

δ_H (500 MHz; DMSO-*d6*) 1.07 (3 H, d, *J* = 6.0 Hz), 1.44 (2 H, m), 1.74 (2 H, m), 3.53 (2 H, m), 3.65 (1 H, m), 3.83 (3 H, s), 5.78 (2 H, s), 6.87 (1 H, d, *J* = 5.1 Hz), 7.20 (1 H, s), 7.28 – 7.31 (4 H, m), 7.38 (2 H, t, *J* = 7.2 Hz), 8.55 (1 H, s), 8.65 – 8.69 (2 H, m). δ_C (125 MHz; DMSO-*d6*) 23.8, 24.5, 36.0, 43.4, 56.0, 56.3, 65.5, 98.0, 100.4, 112.3, 116.0, 126.6, 126.8, 128.2, 129.0, 135.5, 139.7, 146.6, 155.0, 162.8, 165.7. HRMS(ESI) Expected m/z 351.20 [M+H]+ Found m/z 351.207 [M+H]+

Compound **12b**

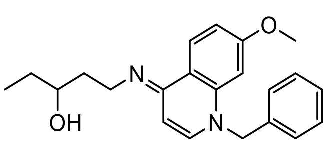

δ_H (500 MHz; DMSO-*d*6) 0.87 (3 H, t, *J* = 7.3 Hz), 1.40 (2 H, m), 1.62 (1 H, m), 1.82 (1 H, m), 3.50 (1 H, m), 3.61 (2 H, m), 3.83 (3 H, s), 5.77 (2 H, s), 6.85 (1 H, d, *J* = 5.0 Hz), 7.20 (1 H, s) 7.29 – 7.31 (4 H, m), 7.37 (2 H, t, *J* = 7.3 Hz), 8.59 (1 H, m), 8.67 (1 H, m). δ_C (500 MHz; DMSO-*d*6) 10.9, 29.8, 34.9, 40.5, 55.6, 55.9, 68.6, 97.6, 100.2, 115.8, 126.2, 126.8, 129.0, 129.2, 135.3, 146.4, 162.8. HRMS(ESI) Expected m/z 351.20 [M+H]+ Found m/z 351.196 [M+H]+

Compound **12c**

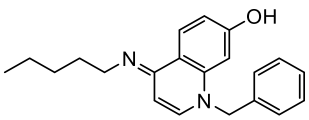

^{1}H NMR (500 MHz, DMSO-d6) δ 0.90 (3 H, t, *J* = 6.9 Hz), 1.33 – 1.37 (4 H, m), 1.65 (2 H, p, *J* = 7.2 Hz), 3.42 (2 H, t, *J* = 6.4 Hz), 5.46 (2H, s), 6.43 (1 H, s), 6.52 (1 H, d, *J* = 7.5 Hz), 6.68 (1 H, d, *J* = 9.3 Hz), 7.18 (2 H, d, *J* = 7.3 Hz), 7.26 (1 H, t, *J* = 7.3 Hz), 7.33 (2 H, t, J = 7.4 Hz), 8.02 (1 H, d, *J* = 9.5 Hz), 8.34 (1 H, d, *J* = 7.5 Hz). ^{13}C NMR (126 MHz, DMSO-d6) δ 13.9, 21.9, 28.2, 28.6, 42.6, 95.0, 101.6, 105.7, 122.1, 224.6, 126.4, 127.7, 128.8, 135.9, 141.2, 144.6, 153.7, 171.2. HRMS(ESI) Expected m/z 321.19 [M+H]+ Found m/z 321.196 [M+H]+

Whole cell biotransformation scaled-up reaction of compound **6**

Streptomyces lividans TK24 (John Innes Centre, UK) expressing a polycistronic operon consisting of the PolyCYP 194 cytochrome P450 enzyme and redox partners under a strong constitutive *ermE** promoter was constructed as described in patent US20220025416A1 and designated as *Streptomyces lividans* HD005.

To assess the scalability of using whole cell biotransformation to produce **6c**, a dose-confirmation experiment using parent compound **6** was performed against *Streptomyces lividans* HD005. Seed shake-flask cultures containing 50 mL of M3G medium in a 250 ml flask were inoculated with 0.1 mL *S. lividans* HD005 glycerol stocks and grown for 3 days at 27 °C and shaking on a Kuhner (AG Switzerland) 5 cm orbital shaker at 200 rpm. After the specified growth period, production cultures were setup by inoculating fresh flasks containing 50 mL of M3G medium in a 250 ml flask with 1 ml of overnight culture and were further incubated for 2 days at 27 °C. Dose timings were investigated between 24 to 72 hour post-inoculation with different concentrations of parent compound **6** to a final concentration of 100 to 500 mg/L. All reactions were shaken at 200 rpm at 27 °C, sampled at 24-, 48- and 72-hours post-dosage and immediately extracted with an equal volume of acetonitrile prior to UPLC-MS analysis.

A 3-fold improvement in volumetric production was obtained when the parent compound **6** was dosed at 24 hours post-inoculation with final concentration of 500 mg/L, 2% HP-*β*-CD formulant and harvested at 72 hours post-dosing. The scaled-up production of **6c** was prepared in a total volume of 3 L. The reactions were pooled and stored in the freezer until ready for processing; UPLC-MS chromatograms of harvested material sample are shown in Figure S12.

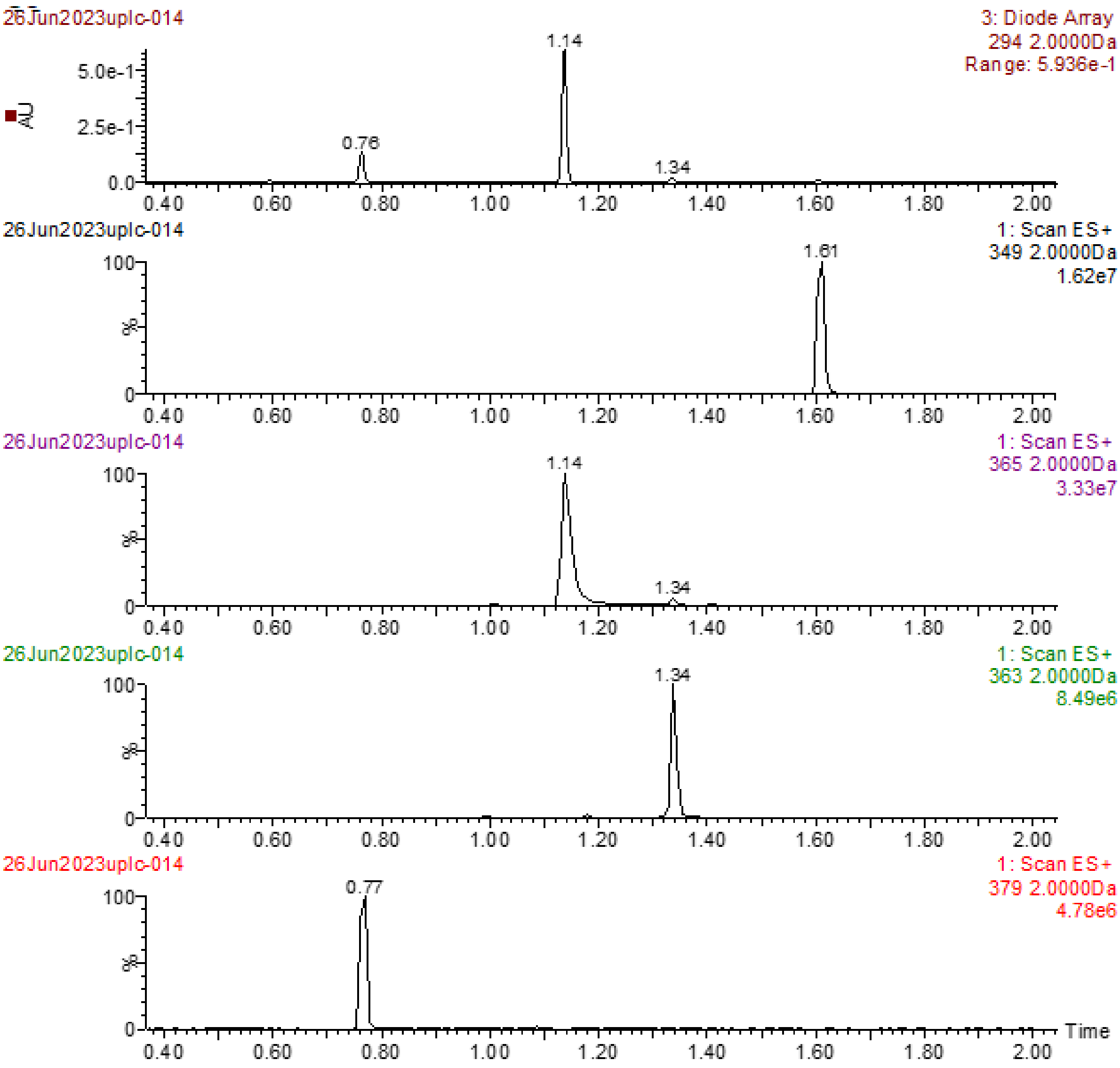

Figure S12 - UPLC-MS chromatograms of post-reaction extracts using Streptomyces lividans HD005 expressing PolyCYP194 cytochrome P450 and redox partners on **6.** Parent compound **6** (EIC: 349 m/z; 2nd trace) elutes at 1.61 minutes and was almost fully converted. Target hydroxylated metabolite **6c** (EIC: 365 m/z; 3rd trace) elutes at 1.14 min with an conversion of 77.7%. In addition, +14 Da (EIC: 363 m/z; 4th trace) and +30 Da metabolites (EIC: 379 m/z; 5th trace) were also detected.

Purification of compound **6c**

The total 3 L fermentation reaction was defrosted in a water bath set at 40°C, centrifuged at 4000 x *g* for 30 minutes and the supernatant decanted. The cell pellets were extracted with acetonitrile and centrifuged, the supernatant of approximately 430 mL was equally decanted into two 5L bottles. To each bottle, 1.7 L of acetonitrile and 340 grams of ammonium sulphate were added. Bottles were mixed and allowed to stand to separate the organic

upper layer. Organic phases were collected, combined and concentrated *in vacuo* to a residual aqueous solution.

Primary fractionation was performed over a Waters Sunfire C18 column (5 µM, 30 mm i.d. x 100 mm) with a corresponding guard column at room temperature (ca. 21°C). The elution program used a water:MeCN:2% formic acid starting at 85:10:5 and 20 mL/min then increasing the flow rate to 40 mL/min over 1 minute (and continued at this flow thereafter). After 2 minutes of flow, the elution program changed linearly to 78:17:5 over 14 minutes followed by wash and re-equilibration steps; resulting in **6c** eluting between 8 and 11 minutes.

A secondary fractionation was performed over a Waters Xbridge Phenyl column (5 µM, 30 mm i.d. x 100 mm) with a corresponding guard column at room temperature (ca. 21°C). The elution program used a water:MeCN:2% formic acid starting at 85:10:5 and 20 mL/min then increasing the flow rate to 40 mL/min over 1 minute (and continued at this flow thereafter). The elution program changed linearly to 67:28:5 over 12 minutes and followed by wash and re-equilibration steps; resulting in **6c** eluting between 5 and 10 minutes.

The pooled fractions containing **6c** were lyophilised to give 885.58 mg as a light brown solid.

Whole cell biotransformation scaled-up reaction of compound **7**

Dose confirmation experiment using *Streptomyces lividans* HD005 to produce **7a** and **7b** using parent compound **7** was performed as described above.

Maximum conversion was obtained when the parent compound **7** was dosed at 48 hours post-inoculation with final concentration of 150 mg/L, 2% HP-*β*-CD formulant and harvested at 24 hours post-dosing. The scaled-up production of **7a** and **7b** was prepared in a total volume of 4.7 L. The reactions were pooled and stored in the freezer until ready for processing; UPLC-MS chromatogram of harvested material sample is shown in Figure S13.

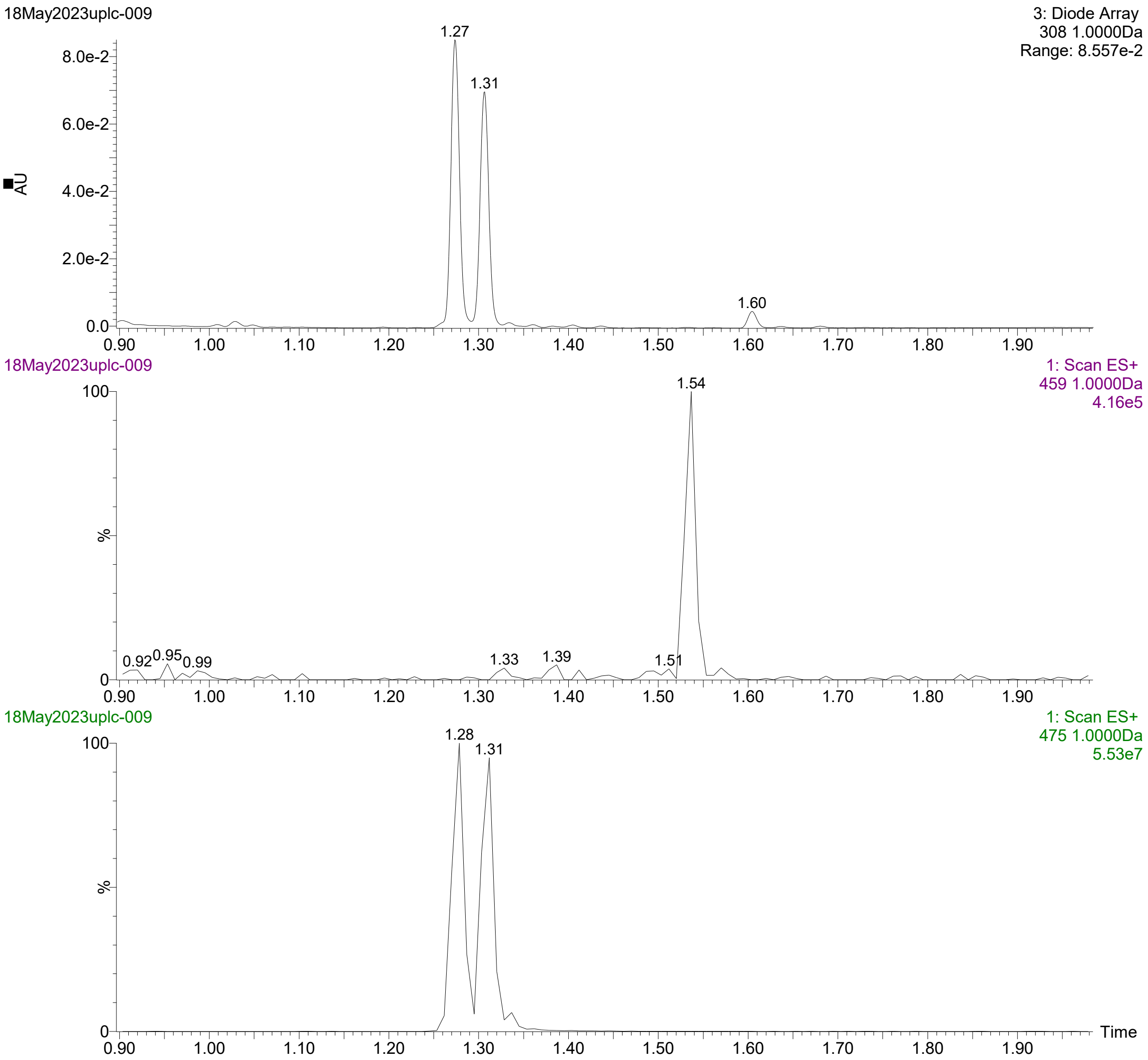

Figure S13 - UPLC-MS chromatograms of post-reaction extracts using Streptomyces lividans HD005 expressing PolyCYP194 cytochrome P450 and redox partners on **7.** Parent compound **7** (EIC: 459 m/z; 2nd trace) elutes at 1.54 minutes and was almost fully converted into the 2 hydroxylated metabolite **7a** and **7b** (EIC: 475 m/z; 3rd trace) eluting at 1.27 and 1.31 mins with conversions of 55.0% and 45.0%, respectively.

Purification of compounds **7a** and **7b**

The total 4.7 L fermentation reaction was centrifuged at 4000 x *g* for 30 minutes and the supernatant decanted. The cell pellets were extracted with 420 mL of acetonitrile and centrifuged, the aqueous supernatant was absorbed onto Diaion HP20 resin column (500 mL resin preconditioned in methanol and rinsed with 2 column volumes of water), rinsed with 2 column volumes of water and eluted with 2 L of acetonitrile. A total of 2 L of eluants were combined and approximately 200 grams of ammonium sulphate was added. Bottles were mixed and allowed to stand to separate the organic upper layer. Organic phases were

collected, concentrated *in vacuo* to a residual aqueous solution and diluted with 600 ml water:MeCN (1:1).

Primary fractionation was performed using a Waters Atlantis T3 column (5 µM, 19 mm i.d. x 100 mm) with a corresponding guard column at room temperature (ca. 21°C). The elution program used a water:MeCN:2% formic acid starting at 85:10:5 and 10 mL/min then increasing the flow rate to 17 mL/min over 1 minute (and continued at this flow thereafter). After 1 minutes of flow, the elution program changed linearly to 80:15:5 over 1 minute followed by another change linearly to 61:34:5 over 19 minutes followed by wash and re-equilibration steps; resulting in peak 1 containing a mixture of **7a** and **7b** eluting between 18.5 and 20 minutes and peak 2 containing **7b** eluting between 20 and 21 minutes.

A secondary fractionation was performed on peak 1 fraction containing a mixture of both compounds over a Waters Sunfire C_{18} column (5 µM, 30 mm i.d. x 100 mm) with a corresponding guard column at room temperature (ca. 21°C). The elution program used a water:MeCN:2% formic acid starting at 75:20:5 and 10 mL/min then increasing the flow rate to 40 mL/min over 1 minute (and continued at this flow thereafter). The elution program changed linearly to 68:27:5 over 14 minutes and followed by wash and re-equilibration steps; resulting in peak 1 containing **7a** eluting between 13.5 and 15.0 minutes and **7b** eluting between 15 and 16 minutes. Peak 1 containing **7a** from secondary fractionation were pooled and lyophilised to give 243.88 mg of beige powder. Peak 2 containing **7b** were pooled from the primary and secondary fractions and lyophilised to give 194.12 mg of beige powder.

Whole cell biotransformation scaled-up reaction of compound **11**

Dose confirmation experiment using *Streptomyces lividans* HD005 to produce **11a** and **11b** using parent compound **11** was performed as described above.

Maximum conversion was obtained when the parent compound **11** was dosed at 24 hours post-inoculation with final concentration of 500 mg/L, 2% HP-*β*-CD formulant and harvested at 98 hours post-dosing. The scaled-up production of **11a** and **11b** was prepared in a total volume of 0.45 L. The reactions were pooled and stored in the freezer until ready for processing; UPLC-MS chromatogram of harvested material sample is shown in Figure S14.

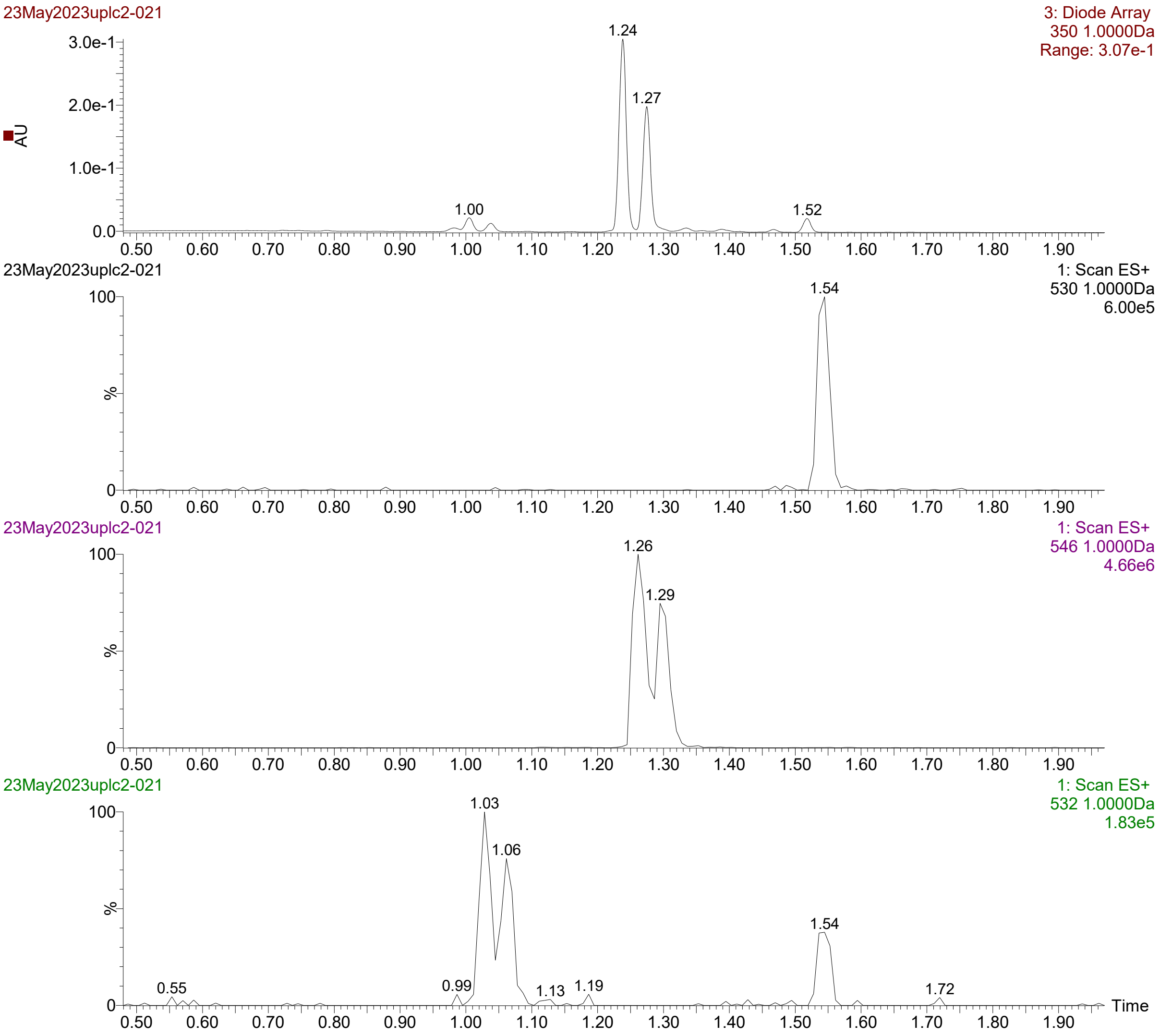

Figure S14 - UPLC-MS chromatograms of post-reaction extracts using Streptomyces lividans HD005 expressing PolyCYP194 cytochrome P450 and redox partners on **11.** Parent compound **11** (EIC: 530 m/z; 1st trace) elutes at 1.54 minutes and was almost fully converted. The 2 hydroxylated metabolites, **11a** and **11b** (EIC: 475 m/z; 2nd trace), elute at 1.27 and 1.31 mins with conversions of 51.9% and 35.9% respectively. In addition, +2 Da metabolites (EIC: 532 m/z; 4th trace) were also detected.

Purification of compounds **11a** and **11b**

The total 0.45 L fermentation reaction was diluted two-fold with dH_2O and centrifuged at 4000 x *g* for 15 minutes. The clarified supernatant was applied onto a Diaion HP20 resin column (129 mL resin pre-equilibrated in water), and first washed with 150 mL of water, then eluted with 150 mL of increasing proportions of acetonitrile in water (10:90, 30:70 & 60:40) and finally washed with 240 mL of acetonitrile (100:0)

The majority of the target hydroxylated metabolites were located in the 60:40 and 100:0 fractions (water:MeCN) and these were concentrated *in vacuo* to a residual aqueous solution and diluted with 250 mL of water:MeCN (5:2).

Primary fractionation was performed over a Waters XSelect CSH C_{18} column (5 µM, 30 mm i.d. x 100 mm) with a corresponding guard column at room temperature (ca. 21°C). The elution program used a water:MeCN:200 mM NH_4HCO_3/H_2O starting at 85:10:5 and 10 mL/min then increasing the flow rate to 40 mL/min over 1 minute (and continued at this flow thereafter). After 1 minutes of flow, the elution program changed linearly to 61:34:5 over 16 minutes followed by wash and re-equilibration steps; resulting in two overlapping peaks with peak 1 between 14.6 and 15.3 minutes and peak 2 between 15.3 to 15.9 minutes

A secondary fractionation was performed on pooled peak 2 over a Waters Atlantis T3 column (5 µM, 19 mm i.d. x 100 mm) with a corresponding guard column at room temperature (ca. 21°C). The elution program used a water:MeCN:2% formic acid starting at 85:10:5 and 5 mL/min then increasing the flow rate to 17 mL/min over 1 minute (and continued at this flow thereafter). The elution program changed linearly to 73:22:5 over 12 minutes followed by wash and re-equilibration steps; resulting in two peaks with peak 1 containing a mixture of **11a** and **11b** and peak 2 containing **11b** between 8 and 11 minutes**.** Peak 2 containing **11b** were pooled from the secondary fractions and lyophilised to give 26.37 mg of white powder.

Peak 1 from the secondary fractionation was combined with peak 1 from the primary fractionation. A further fractionation was performed on the combined peaks over a Waters XSelect CSH C_{18} column (5 µM, 30 mm i.d. x 100 mm) with a corresponding guard column at room temperature (ca. 21°C). The elution program used a water:MeCN: 200 mM NH_4HCO_3/H_2O starting at 85:10:5 and 10 mL/min then increasing the flow rate to 40 mL/min over 1 minute (and continued at this flow thereafter). The elution program changed linearly to 61:34:5 over 16 minutes followed by wash and re-equilibration steps; resulting in **11a** eluting between 14 and 17 minutes. Fractions containing **11a** were pooled and lyophilised to give 54.33 mg of white powder.

The biomass was extracted with 100 mL of acetonitrile, combined with dose-escalation samples and centrifuged at 4,000 xg for 15 minutes. A primary fractionation was performed on the supernatant over a Waters XSelect CSH C_{18} column (5 µM, 30 mm i.d. x 100 mm) with a corresponding guard column at room temperature (ca. 21°C). The elution program used a water:MeCN: 200 mM NH_4HCO_3/H_2O starting at 85:10:5 and 10 mL/min then increasing the flow rate to 40 mL/min over 1 minute (and continued at this flow thereafter). The elution program changed linearly to 61:34:5 over 16 minutes followed by wash and re-equilibration steps; resulting in two peaks containing between 14.4 and 16.8 minutes. Fractions containing **11a** and **11b** were pooled separately and lyophilised to give 26.49 and 22.27 mg of white powder respectively.

A total of 80.82 mg and 48.64 mg of **11a** and **11b** were purified from the whole cell biotransformation scale up of compound **11**.

Biological Assays

Schistosomula

All procedures performed in mice adhered to the United Kingdom Home Office Animals (Scientific Procedures) Act of 1986 (project licenses: PP2955700 and P3B8C46FD) as well as the European Union Animals Directive 2010/63/EU and were approved by Aberystwyth University's Animal Welfare and Ethical Review Body.

A Puerto Rican strain of Schistosoma mansoni (NMRI, Naval Medical Research Institute) was used in this study and passaged between Mus musculus (Tuck Ordinary) and Biomphalaria glabrata (both NMRI albino and pigmented outbred strains[1]) snail hosts. Cercariae were shed from B. glabrata by exposure to light in a heated room (26°C) for 90 min. The schistosomula stage was obtained by mechanical transformation of cercariae and dispensed into 384-well tissue culture plates (120 parasites/well) containing Basch Media (Basch, 1981) and dosed with serial dilutions of each compound starting from 10 µM in a 7-point titration. DMSO (0.625%) and auranofin (10 µM in 0.625% DMSO) were used as negative and positive controls respectively. Cultures were incubated for 72h at 37 °C with 5% CO_2. Compounds were screened for activity using a high-throughput, high content imaging platform, Roboworm, as described previously[2,3]. Compounds were quantified according to their effect on schistosomula motility and phenotype. Any compound that resulted in a score threshold that is equal to or falls below the values of -0.35 for motility and/ or −0.15 for phenotype, in ≥70% of the assayed larva was considered a hit. Z´ values obtained from all schistosomula screens were greater than 0.35 for both motility and phenotype.

EC_{50} values were calculated from the titrated concentrations by non-linear regression, after log transformation of concentrations and data normalization using GraphPad Prism 7.02.

Ld pro

The Ld pro assay was performed as previously described[4].

Tc-Invero

Parasite and mammalian cell culture as well as compound efficacy against intracellular stage *T. cruzi* were performed as previously described[5,6] *T. cruzi* strain Silvio X10/7 subclone A1 and host Vero cells (African green monkey kidney cells, ECCAC 84113001) were used for this assay with a 96-hour treatment duration followed by fixation, Hoechst 33342 staining and automated imaging. Ten-point potency curves were generated. All data was normalised to percent inhibition based on the raw data values for the 100% effect control (16 µM

nifurtimox) and the 0% effect control (DMSO) on each plate. Curve fitting was carried out using a four-parameter equation as previously described[7].

Tb bsf

The Tb bsf assay was performed as previously described[8].

Assay Results

Leishmania

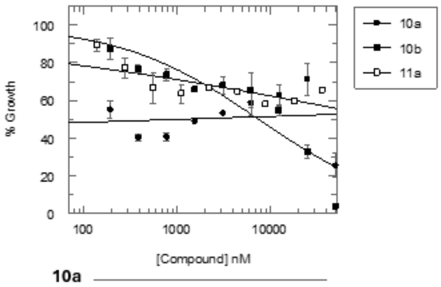

10a

Parameter	Value	Std. Error
IC 50	1081.6799	8378.2802
Slope factor	-0.0275	0.1022

10b

Parameter	Value	Std. Error
IC 50	7526.8780	2350.5001
Slope factor	0.5838	0.1234

11a

Parameter	Value	Std. Error
IC 50	199438.7092	281263.3903
Slope factor	0.1697	0.0574

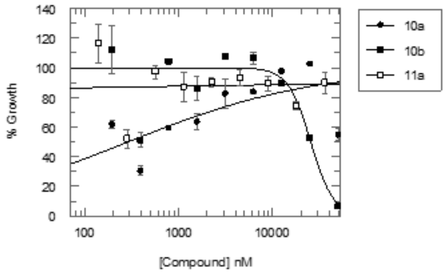

10a

Parameter	Value	Std. Error
IC 50	288.4819	282.7938
Slope factor	-0.4344	0.2127

10b

Parameter	Value	Std. Error
IC 50	25411.9796	4989.4093
Slope factor	3.6084	2.6573

11a

Parameter	Value	Std. Error
IC 50	1.96186e-018	2.48865e-017
Slope factor	-0.0409	0.0092

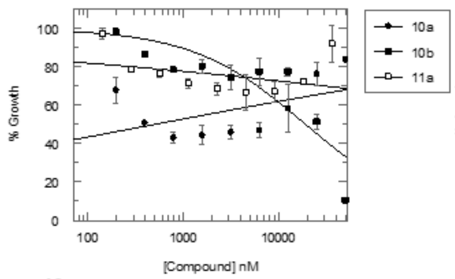

10a

Parameter	Value	Std. Error
IC 50	498.9703	791.7519
Slope factor	-0.1631	0.1023

10b

Parameter	Value	Std. Error
IC 50	19064.0101	7148.4093
Slope factor	0.7274	0.2010

11a

Parameter	Value	Std. Error
IC 50	43449383.3943	73680946.3680
Slope factor	0.1165	0.0466

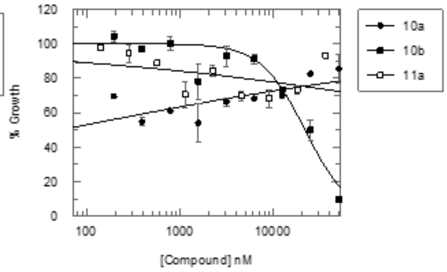

10a

Parameter	Value	Std. Error
IC 50	52.4190	71.9656
Slope factor	-0.1860	0.0673

10b

Parameter	Value	Std. Error
IC 50	21624.4486	2900.0740
Slope factor	1.8349	0.4033

11a

Parameter	Value	Std. Error
IC 50	9893441.9675	17151179.2978
Slope factor	0.1812	0.0652

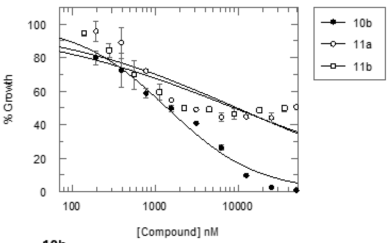

10b

Parameter	Value	Std. Error
IC 50	1385.2995	143.1021
Slope factor	0.8043	0.0692

11a

Parameter	Value	Std. Error
IC 50	9598.4203	4643.6348
Slope factor	0.3752	0.1036

11b

Parameter	Value	Std. Error
IC 50	8887.6017	4107.1402
Slope factor	0.3382	0.0835

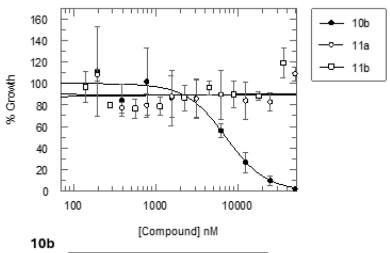

10b

Parameter	Value	Std. Error
IC 50	7162.1380	839.9276
Slope factor	1.7883	0.3097

11a

Parameter	Value	Std. Error
IC 50	8.32090e-019	2.30560e-017
Slope factor	-0.0430	0.0561

11b

Parameter	Value	Std. Error
IC 50	8.27701e+079	8.49397e+080
Slope factor	0.0122	0.0060

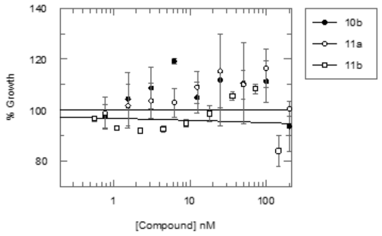

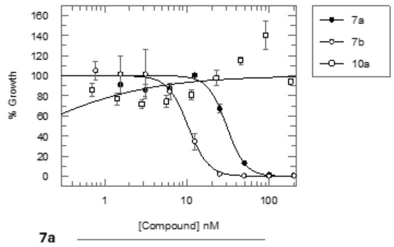

7a

Parameter	Value	Std. Error
IC 50	30.3345	2.5463
Slope factor	3.8214	0.9692

7b

Parameter	Value	Std. Error
IC 50	10.3080	0.2591
Slope factor	3.5211	0.2677

10a

Parameter	Value	Std. Error
IC 50	0.1323	0.2433
Slope factor	-0.6091	0.5380

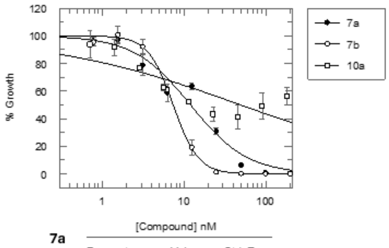

7a

Parameter	Value	Std. Error
IC 50	11.9991	1.7095
Slope factor	1.2464	0.1999

7b

Parameter	Value	Std. Error
IC 50	7.3706	0.1662
Slope factor	2.8383	0.1606

10a

Parameter	Value	Std. Error
IC 50	49.1795	27.5720
Slope factor	0.3666	0.1123

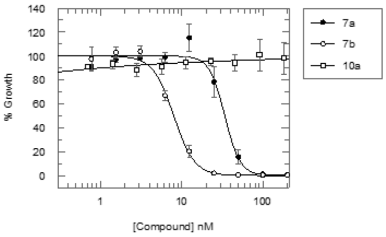

7a

Parameter	Value	Std. Error
IC 50	33.8506	2.4750
Slope factor	4.6066	0.9774

7b

Parameter	Value	Std. Error
IC 50	7.9876	0.3013
Slope factor	3.4049	0.3535

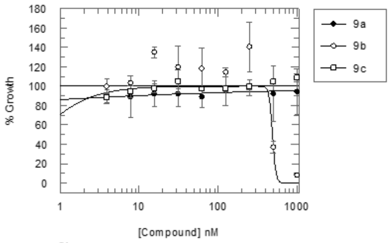

9b

Parameter	Value	Std. Error
IC 50	488.2668	184.8693
Slope factor	21.5436	247.4415

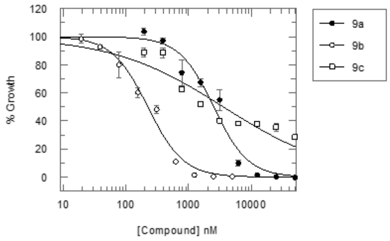

9a

Parameter	Value	Std. Error
IC 50	2505.0220	309.7623
Slope factor	1.5829	0.2812

9b

Parameter	Value	Std. Error
IC 50	222.9329	18.6628
Slope factor	1.5285	0.1727

9c

Parameter	Value	Std. Error
IC 50	3566.5765	1006.0042
Slope factor	0.4879	0.0872

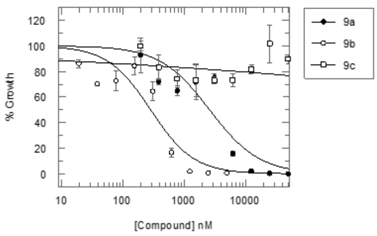

9a

Parameter	Value	Std. Error
IC 50	2602.1911	771.9493
Slope factor	1.0744	0.3091

9b

Parameter	Value	Std. Error
IC 50	285.7614	73.3242
Slope factor	1.2375	0.3328

9c

Parameter	Value	Std. Error
IC 50	1.00698e+010	1.66398e+010
Slope factor	0.0982	0.0452

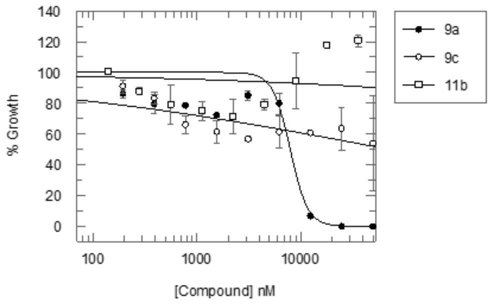

9a

Parameter	Value	Std. Error
IC 50	7944.8590	1383.3467
Slope factor	5.1892	2.7275

9c

Parameter	Value	Std. Error
IC 50	71206.0569	68760.6906
Slope factor	0.2214	0.0704

11b

Parameter	Value	Std. Error
IC 50	1.88649e+009	4.16819e+009
Slope factor	0.2115	0.0807

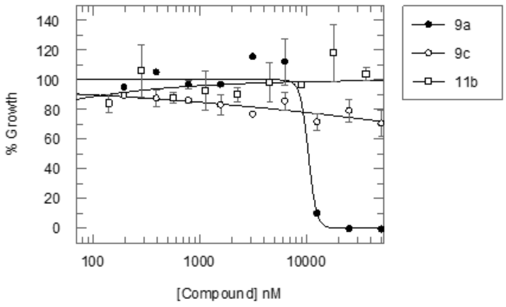

9a

Parameter	Value	Std. Error
IC 50	10328.3841	646.4182
Slope factor	11.6729	3.4657

9c

Parameter	Value	Std. Error
IC 50	5683191.8737	9745188.8307
Slope factor	0.1988	0.0491

11b

Parameter	Value	Std. Error
IC 50	1.4784	3.1149
Slope factor	-0.4890	0.2646

Figure S15:- Leishmania biological results

T. brucei

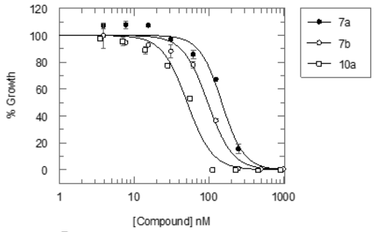

7a

Parameter	Value	Std. Error
IC 50	149.6026	10.0117
Slope factor	2.9383	0.4842

7b

Parameter	Value	Std. Error
IC 50	97.0033	5.8407
Slope factor	2.6225	0.3482

10a

Parameter	Value	Std. Error
IC 50	51.5125	4.2356
Slope factor	2.5773	0.4761

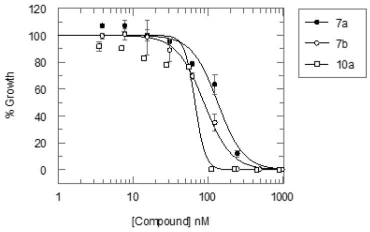

7a

Parameter	Value	Std. Error
IC 50	136.1015	10.5776
Slope factor	2.5178	0.4295

7b

Parameter	Value	Std. Error
IC 50	89.1133	3.9238
Slope factor	2.3835	0.2183

10a

Parameter	Value	Std. Error
IC 50	67.6570	8.5077
Slope factor	6.2877	3.3211

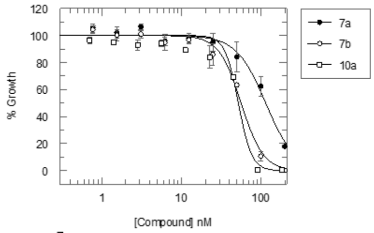

7a

Parameter	Value	Std. Error
IC 50	114.6601	6.5178
Slope factor	2.3627	0.2934

7b

Parameter	Value	Std. Error
IC 50	56.3149	2.6523
Slope factor	3.1138	0.4044

10a

Parameter	Value	Std. Error
IC 50	51.9978	4.0792
Slope factor	5.1914	1.8654

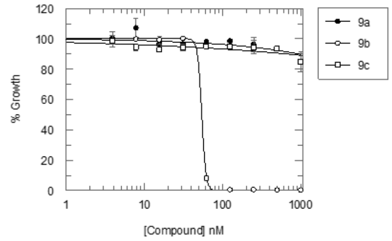

9a

Parameter	Value	Std. Error
IC 50	103890.9237	53069.7850
Slope factor	0.4671	0.0377

9b

Parameter	Value	Std. Error
IC 50	54.1356	6.2979
Slope factor	16.9779	12.8436

9c

Parameter	Value	Std. Error
IC 50	10128011.1796	14181029.0884
Slope factor	0.2278	0.0259

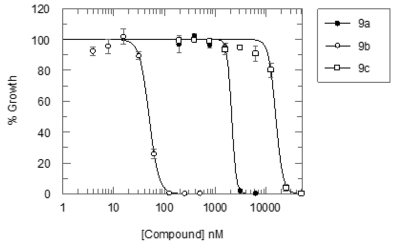

9a

Parameter	Value	Std. Error
IC 50	2104.2235	92.1222
Slope factor	10.0545	1.3257

9b

Parameter	Value	Std. Error
IC 50	49.6975	1.7306
Slope factor	4.6618	0.5359

9c

Parameter	Value	Std. Error
IC 50	15537.7049	834.4670
Slope factor	6.2907	1.2503

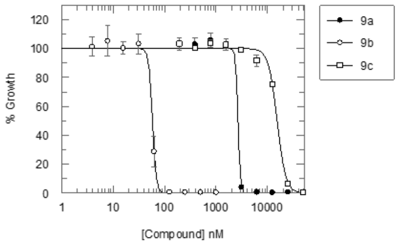

9a

Parameter	Value	Std. Error
IC 50	2674.3206	579.1249
Slope factor	20.0018	26.4376

9b

Parameter	Value	Std. Error
IC 50	57.9897	1.2689
Slope factor	12.0947	3.3414

9c

Parameter	Value	Std. Error
IC 50	15388.3891	555.8389
Slope factor	5.1815	0.6924

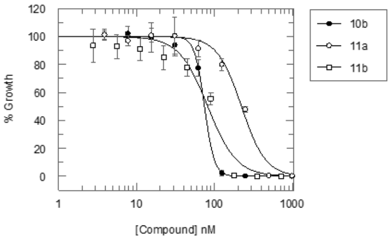

10b

Parameter	Value	Std. Error
IC 50	74.6383	2.4762
Slope factor	6.8801	1.1654

11a

Parameter	Value	Std. Error
IC 50	222.7994	12.5926
Slope factor	2.8059	0.3940

11b

Parameter	Value	Std. Error
IC 50	82.2317	9.3199
Slope factor	2.3604	0.5395

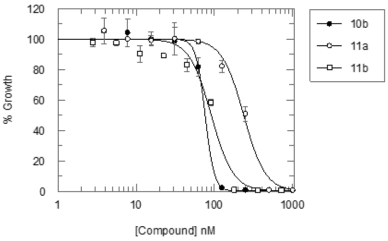

10b

Parameter	Value	Std. Error
IC 50	76.1446	3.2300
Slope factor	7.5338	1.4815

11a

Parameter	Value	Std. Error
IC 50	236.4345	11.8720
Slope factor	3.1551	0.4683

11b

**Parameter**	**Value**	**Std. Error**
**IC 50**	**91.1177**	**7.4252**
**Slope factor**	**3.1088**	**0.7127**

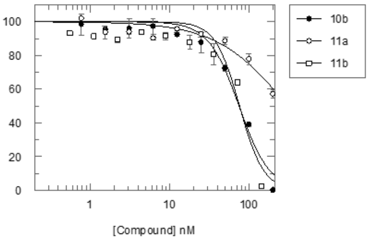

10b

Parameter	Value	Std. Error
IC 50	74.7789	5.3202
Slope factor	2.2650	0.3189

11a

Parameter	Value	Std. Error
IC 50	305.9214	73.6368
Slope factor	0.9756	0.1763

11b

Parameter	Value	Std. Error
IC 50	75.7928	9.0544
Slope factor	2.8649	0.8606

Figure S16:- T. brucei biological results

Chemical Synthesis

Synthesis of Compound **6 is shown in Figure S17**

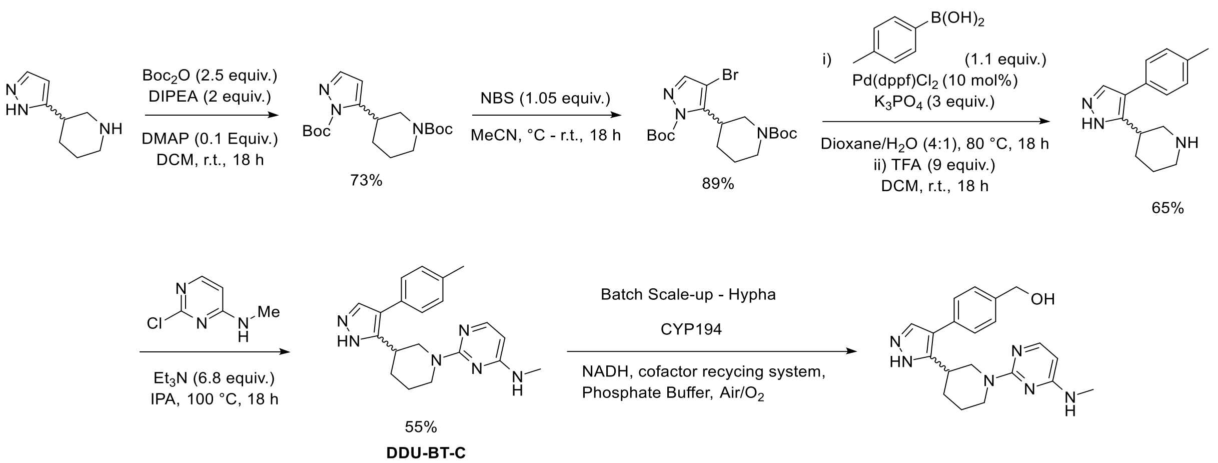

Figure S17:- Synthesis of Compound 6

Synthesis of tert-butyl 3-(1-(tert-butoxycarbonyl)-1H-pyrazol-3-yl)piperidine-1-carboxylate

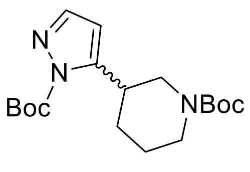

In a 100 mL flask, 3-(1H-pyrazol-3-yl)piperidine (1 g, 6.3 mmol, 1 equiv.), DMAP (80.8 mg, 0.66 mmol, 0.1 equiv.), and Di-tert-butyl dicarbonate (3.6 g, 16.5 mmol, 2.5 equiv.) were sealed under N_2. DCM (22 mL, 0.3 M) was added, followed by dropwise addition of DIPEA (1.7 mL, 12.6 mmol, 1.9 equiv.). The reaction mixture was then stirred at room temperature for 18 h. The Reaction mixture was then washed with H_2O (20 mL), and aqueous phase was back extracted with DCM (20 mL). The combined organic phases were washed with brine (20 mL), passed through a phase separator and concentrated under vacuum. The crude material was then purified by column chromatography (silica gel, 24 g, dry load, 0-100% EtOAc/heptanes) and fractions containing product were combined and concentrated to afford the title compound as a white solid (1.7 g, 73% yield).

^{1}H NMR (400 MHz, $CDCl_3$): δ 7.96 (d, *J* = 2.8 Hz, 1H), 6.24 (d, *J* = 2.8 Hz, 1H), 4.21 (br. s, 1H), 4.01 (br. s, 1H), 3.03 – 2.77 (m, 3H), 2.09 (d, *J* = 11.1 Hz, 1H), 1.77 – 1.68 (m, 2H), 1.64 (s, 9H), 1.46 (s, 9H), 1.33 – 1.29 (m, 1H).

LCMS (ESI) m/z Expected 351[M+H]$^+$ Found 352 [M+H]$^+$

Synthesis of tert-butyl 3-(4-bromo-1-(tert-butoxycarbonyl)-1H-pyrazol-3-yl)piperidine-1-carboxylate

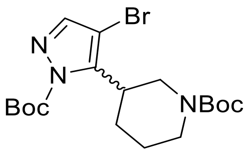

In a 100 mL flask, tert-butyl 3-(1-tert-butoxycarbonylpyrazol-3-yl)piperidine-1-carboxylate (1.7 g, 4.8 mmol, 1 equiv.) was dissolved in MeCN (32 mL, 0.15 M) and cooled to 0 ^{o}C before adding *N*-bromosuccinimide (904 mg, 5.1 mmol, 1.05 equiv.). The reaction mixture was allowed to warm to room temperature and stirred for 18 h. The reaction mixture was concentrated under vacuum and the crude material was then dissolved in EtOAc (20 mL), washed with sat. $NaHCO_3$ (20 mL x 3), passed through a phase separator and concentrated under vacuum to afford the title compound as an off-white gum (1.85 g, 89% yield) which was used without further purification.

^{1}H NMR (500 MHz, $CDCl_3$): δ 7.99 (s, *J* = 16.6 Hz, 1H), 4.27 (br. s, 1H), 4.15 (br. s, 1H), 3.03 – 2.93 (m, 1H), 2.86 (t, *J* = 11.4 Hz, 1H), 2.78 – 2.68 (m, 1H), 2.07 (d, *J* = 13.5 Hz, 1H), 1.89 – 1.73 (m, 2H), 1.62 (s, 9H), 1.46 (s, 9H).

LCMS (ESI) m/z Expected 230 $[M\text{-}Boc_2]^+$ Found 230 $[M\text{-}Boc_2]^+$

Synthesis of 3-(4-(p-tolyl)-1H-pyrazol-3-yl)piperidine

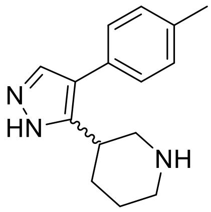

To an oven dried 100 mL flask was added tert-butyl 3-(4-bromo-1-tert-butoxycarbonyl-pyrazol-3-yl)piperidine-1-carboxylate (4.42 g, 10.3 mmol, 1 equiv.), p-tolylboronic acid (1.54 g, 11.3 mmol, 1.1 equiv.) Pd(dppf)Cl_2 (841 mg, 1.03 mmol, 10 mol%) and potassium phosphate tribasic (4.36 g, 20.5 mmol, 3 equiv.). The flask was then capped and purged with N_2 before adding 1,4-Dioxane/H_2O (50 mL, 4:1, 0.2 M). The reaction mixture was then heated to 80 °C and stirred for 18 h. The reaction mixture was cooled to room temperature and filtered through a pad of celite and concentrated under vacuum. The crude material was then taken up in EtOAc, washed with H_2O and brine, passed through a phase separator and concentrated under vacuum to afford the crude Boc-protected product as a brown foam (4.8 g).

The crude material was then dissolved in DCM (90 mL, 0.1 M) and TFA (7.1 mL, 93 mmol, 9 equiv.) was added dropwise. The reaction mixture was then stirred at overnight at room temperature. The reaction mixture was then treated with sat. $NaHCO_3$ (100 mL) and the aqueous phase was extracted with DCM (100 mL). The combined organics were concentrated under vacuum and the residue purified by column chromatography (silica gel, 40 g, 0-20% 2M NH_3 in MeOH/DCM) to afford the title compound as a pale foam (1.63 g, 65% yield).

^{1}H NMR (400 MHz, $CDCl_3$): δ 7.62 (s, 1H), 7.26 (d, *J* = 8.1 Hz, 2H), 7.19 (d, *J* = 7.9 Hz, 2H), 3.24 – 3.19 (m, 1H), 3.15 – 3.09 (m, 1H), 3.04 (dd, *J* = 11.5, 3.2 Hz, 1H), 2.81 (td, *J* = 11.1, 3.0 Hz, 1H), 2.37 (s, 3H), 1.89 – 1.82 (m, 2H), 1.82 – 1.70 (m, 1H), 1.59 – 1.51 (m, 2H).

LCMS (ESI) Expected m/z 242 $[M+H]^+$ Found m/z 242 $[M+H]^+$

Synthesis of N-methyl-2-(3-(4-(p-tolyl)-1H-pyrazol-3-yl)piperidin-1-yl)pyrimidin-4-amine

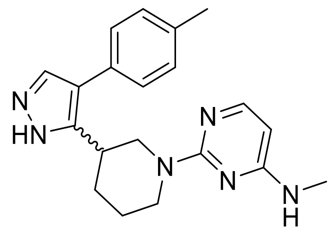

In a 100 mL flask fitted with reflux condenser, to a solution of triethylamine (6.38 mL, 46 mmol, 6.8 equiv.) and 3-(4-(p-tolyl)-1H-pyrazol-3-yl)piperidine (1.62 g, 6.71 mmol, 1 equiv.) in IPA (30 mL, 0.22 M) was added 2-chloro-N-methylpyrimidin-4-amine (2.63 g, 18.3 mmol, 2.7 equiv.) and the reaction mixture was heated at 100 ^oC for 18 h. The reaction mixture was cooled to room temperature and concentrated under vacuum. The crude residue was azeotroped with toluene to afford a crunchy foam. This material was purified by column chromatography (silica gel, 40 g, 0-10% 2M NH_3 in MeOH/DCM) to afford the title compound as an off-white foam (2.07 g, 89% yield).

^{1}H NMR (400 MHz, $CDCl_3$): δ 7.92 (d, *J* = 5.8 Hz, 1H), 7.59 (s, 1H), 7.32 (d, *J* = 8.1 Hz, 2H), 7.18 (d, *J* = 7.8 Hz, 2H), 5.69 (d, *J* = 5.8 Hz, 1H), 5.30 (s, 1H), 4.66 (br. s, 1H), 4.27 (br. s, 1H), 3.97 (br. s, 2H), 3.40 (br. s, 1H), 2.92 (d, *J* = 5.1 Hz, 3H), 2.37 (s, 3H), 2.00 – 1.82 (m, 2H), 1.54 – 1.43 (m, 2H).

LCMS (ESI) Expected m/z 349 $[M+H]^+$ Found m/z 349 $[M+H]^+$

Synthesis of Compound **6c**

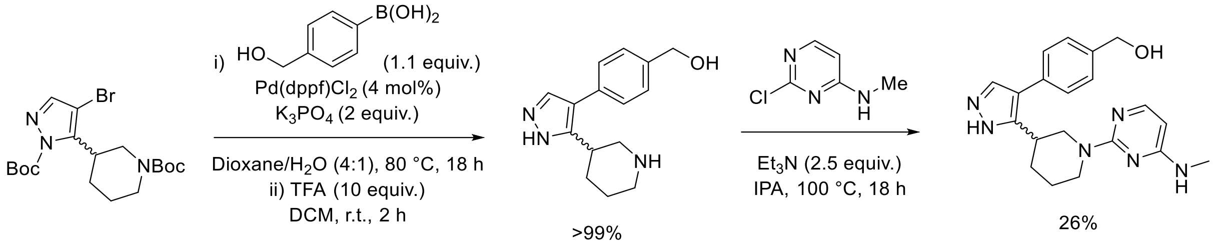

Synthesis of (4-(5-(piperidin-3-yl)-1H-pyrazol-4-yl)phenyl)methanol

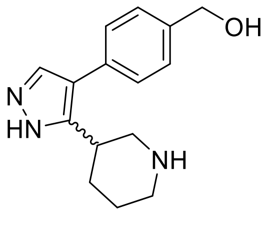

To an oven dried 50 mL flask was added tert-butyl 3-(4-bromo-1-tert-butoxycarbonyl-pyrazol-3-yl)piperidine-1-carboxylate (860 mg,2 mmol, 1 equiv.), (4-(hydroxymethyl)phenyl)boronic acid (334 mg, 2.2 mmol, 1.1 equiv.), Pd(dppf)Cl_2 (73 mg, 0.1mmol, 10 mol%) and potassium phosphate tribasic (553 mg, 4 mmol, 2 equiv.). The flask was then capped and purged with N_2 before adding 1,4-Dioxane/H_2O (10 mL, 4:1, 0.2 M). The reaction mixture was then heated to 80 °C and stirred for 18 h. The reaction mixture was cooled to room temperature and filtered through a pad of celite and concentrated under vacuum. The crude material was then taken up in EtOAc, washed with H_2O and brine, passed through a phase separator and concentrated under vacuum to afford the crude Boc-protected product as a brown foam.

The crude material was then dissolved in DCM (10 mL, 0.2 M) and TFA (1.53 mL, 20 mmol, 10 equiv.) was added dropwise. The reaction mixture was then stirred at room temperature fo 2 h. The solvent was then removed under vacuum to afford the crude product as brown oil. This was taken up in MeOH and passed through a 10 g SXC cartridge, washed with MeOH and then eluted with 7M NH_3/MeOH and concentrated under vacuum to afford the title compound as a crunchy brown foam (514 mg, >99% yield) which was used without further purification.

LCMS (ESI) Expected m/z 258 $[M+H]^+$ Found m/z 258 $[M+H]^+$

Synthesis of (4-(5-(1-(4-(methylamino)pyrimidin-2-yl)piperidin-3-yl)-1H-pyrazol-4-yl)phenyl)methanol

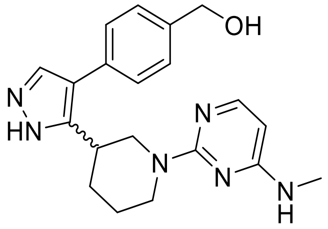

In a 100 mL flask fitted with reflux condenser, to a solution of triethylamine (0.7 mL, 5 mmol, 2.5 equiv.) and (4-(5-(piperidin-3-yl)-1H-pyrazol-4-yl)phenyl)methanol (514 mg, 2 mmol, 1 equiv.) in IPA (6.7 mL, 0.3 M) was added 2-chloro-N-methylpyrimidin-4-amine (287 mg, 2 mmol, 1 equiv.) and the reaction mixture was heated at 100 ^{o}C for 18 h. The reaction mixture was cooled to room temperature and concentrated under vacuum to afford a brown oil. The crude material was dissolved in a 2:1 mixture MeCN/H_2O (7.5 mL) and purified by ACCQPrep reverse phase chromatography (Waters Basic C18 column, 20-30% MeCN/H_2O + 0.1% NH_4OH). Fractions containing product were concentrated by Genevac to afford the title compound as an off-white solid (194 mg, 26% yield).

^{1}H NMR (500 MHz, DMSO) δ 12.76 (d, *J* = 75.1 Hz, 1H), 7.71 (t, *J* = 56.8 Hz, 2H), 7.40 (d, *J* = 7.9 Hz, 2H), 7.29 (d, *J* = 7.9 Hz, 2H), 6.86 (br. s, 1H), 5.71 (d, *J* = 4.9 Hz, 1H), 5.12 (t, *J* = 5.7 Hz, 1H), 4.86 (br.s, 1H), 4.74 (d, *J* = 12.6 Hz, 1H), 4.48 (d, *J* = 5.6 Hz, 2H), 2.98 – 2.82 (m, 2H), 2.75 (t, *J* = 12.2 Hz, 1H), 2.69 (d, *J* = 4.6 Hz, 3H), 1.99 – 1.85 (m, 2H), 1.68 (d, *J* = 12.7 Hz, 1H), 1.48 – 1.34 (m, 1H).

LCMS (ESI) Expected m/z 365 $[M+H]^+$ Found m/z 365 $[M+H]^+$

Analytical Data

tert-butyl 3-(1-(tert-butoxycarbonyl)-1H-pyrazol-3-yl)piperidine-1-carboxylate

^{1}H NMR

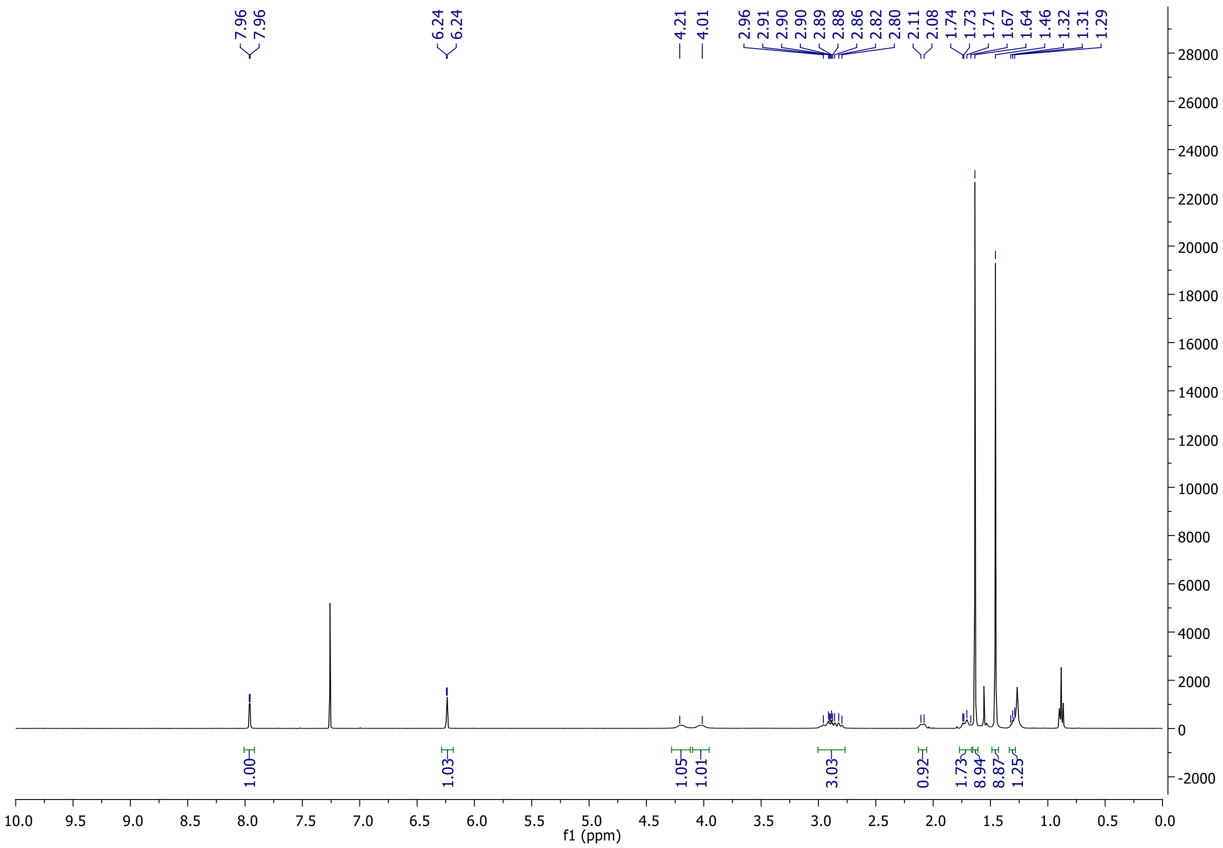

tert-butyl 3-(4-bromo-1-(tert-butoxycarbonyl)-1H-pyrazol-3-yl)piperidine-1-carboxylate

^{1}H NMR

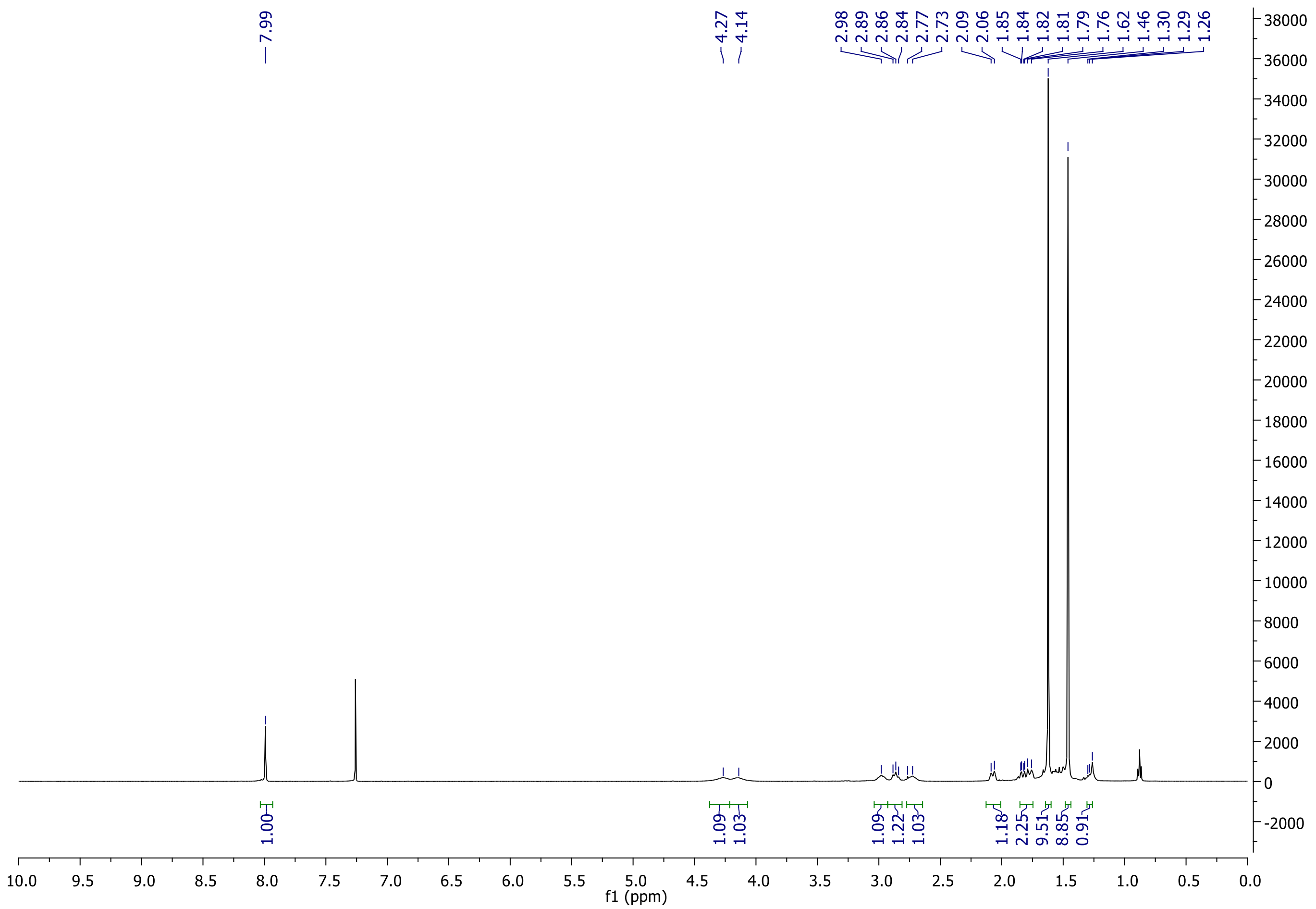

3-(4-(p-tolyl)-1H-pyrazol-3-yl)piperidine

^{1}H NMR

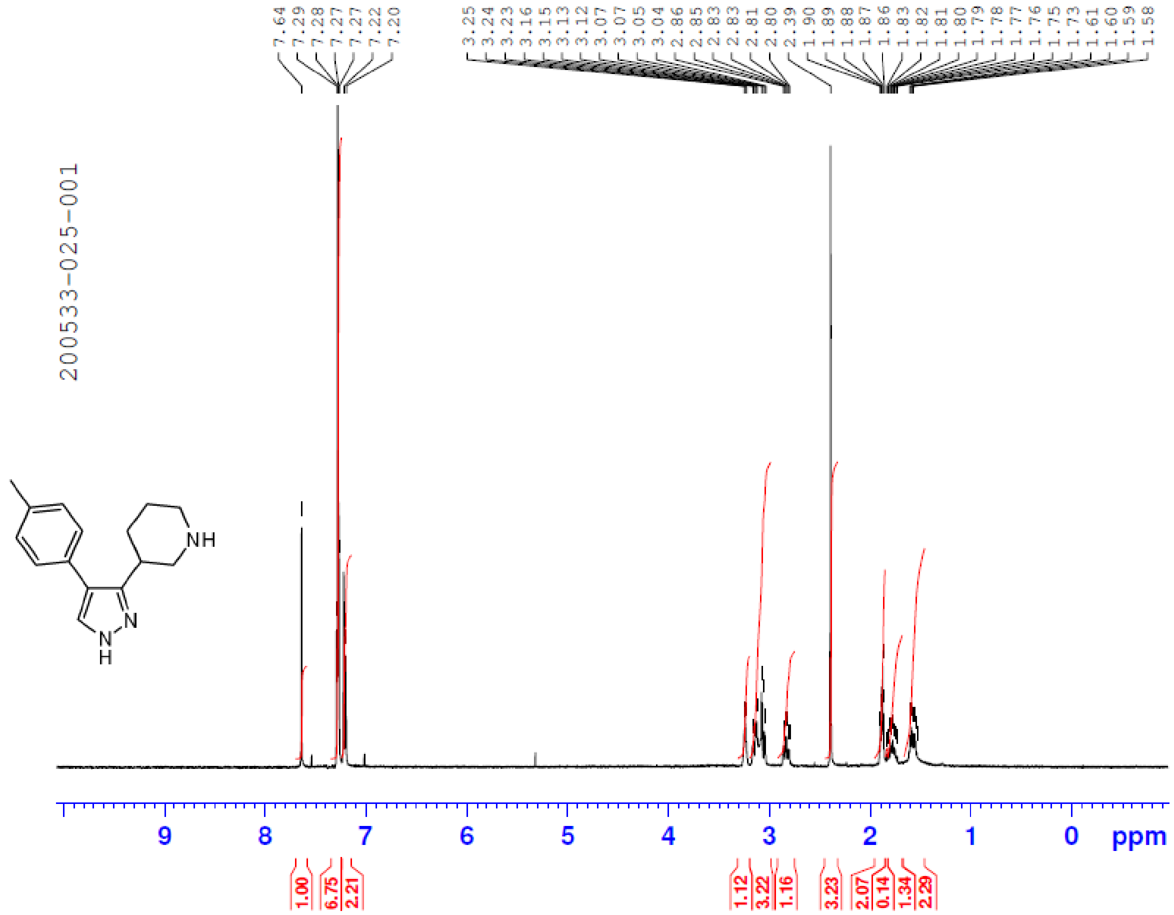

N-methyl-2-(3-(4-(p-tolyl)-1H-pyrazol-3-yl)piperidin-1-yl)pyrimidin-4-amine

^{1}H NMR

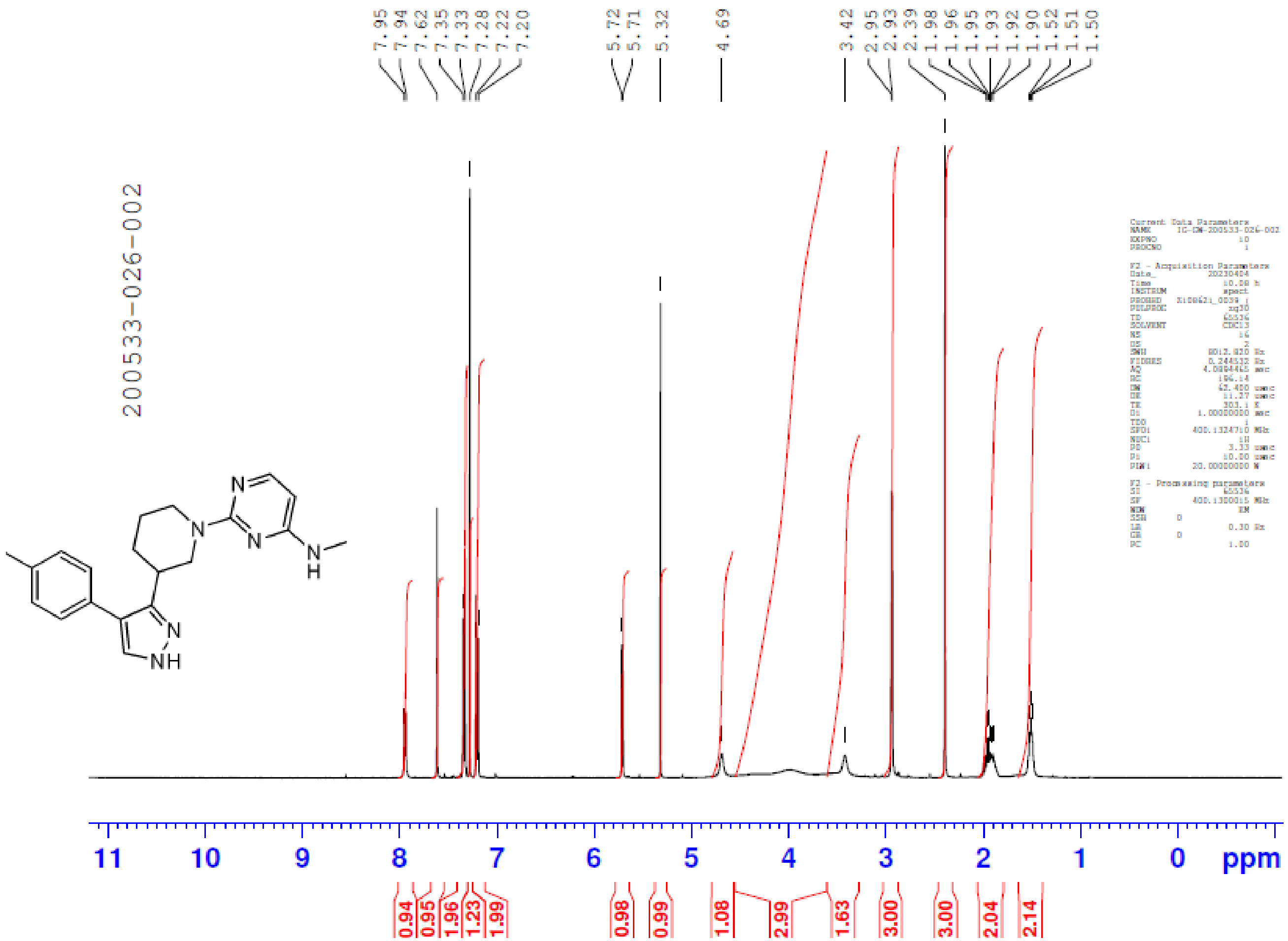

(4-(5-(1-(4-(methylamino)pyrimidin-2-yl)piperidin-3-yl)-1H-pyrazol-4-yl)phenyl)methanol

^{1}H NMR

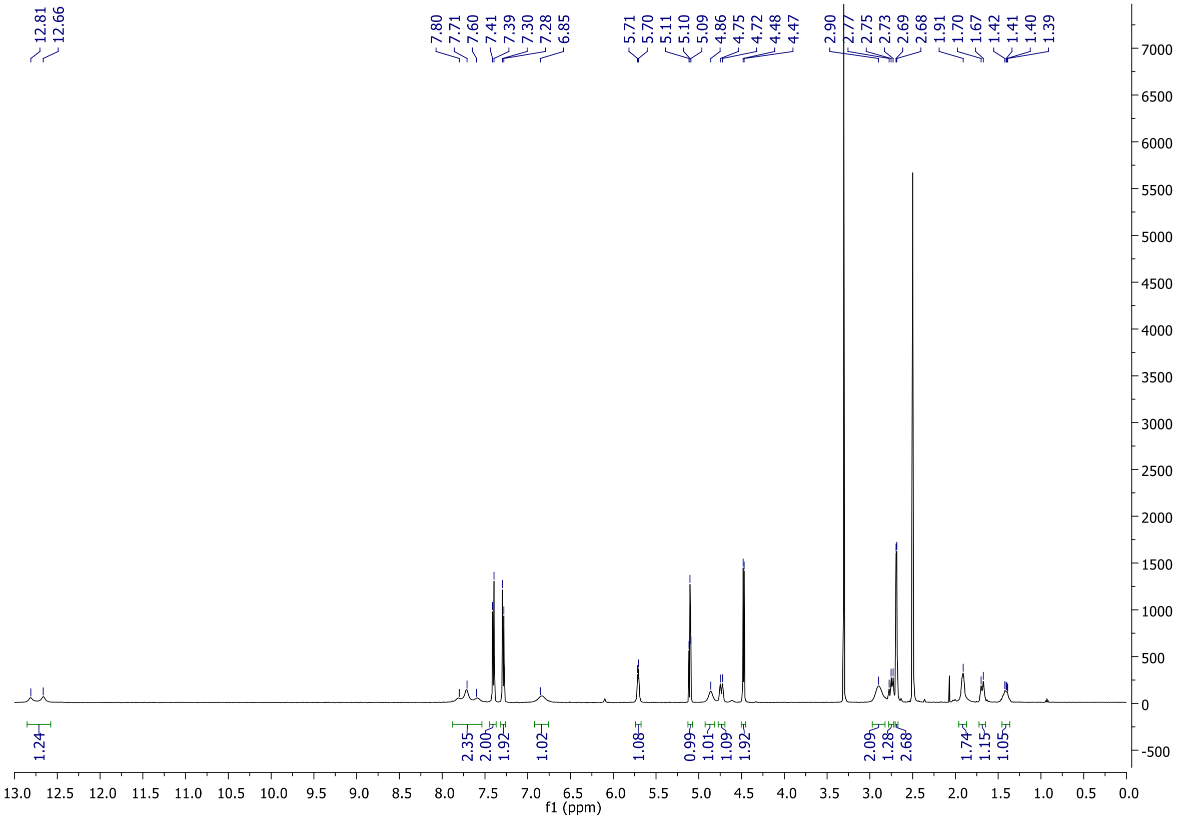

Compound **5a**

^{1}H NMR

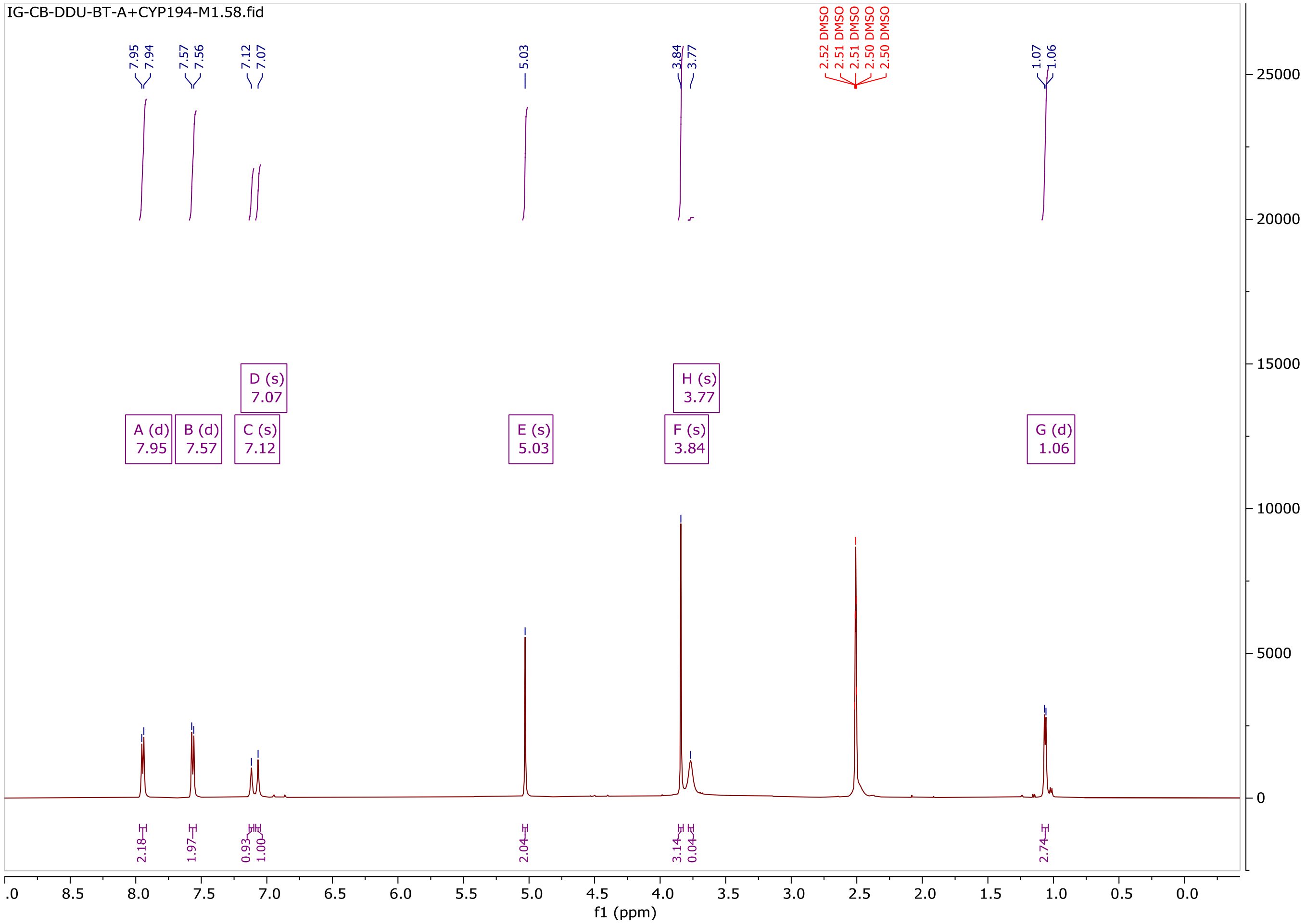

COSY

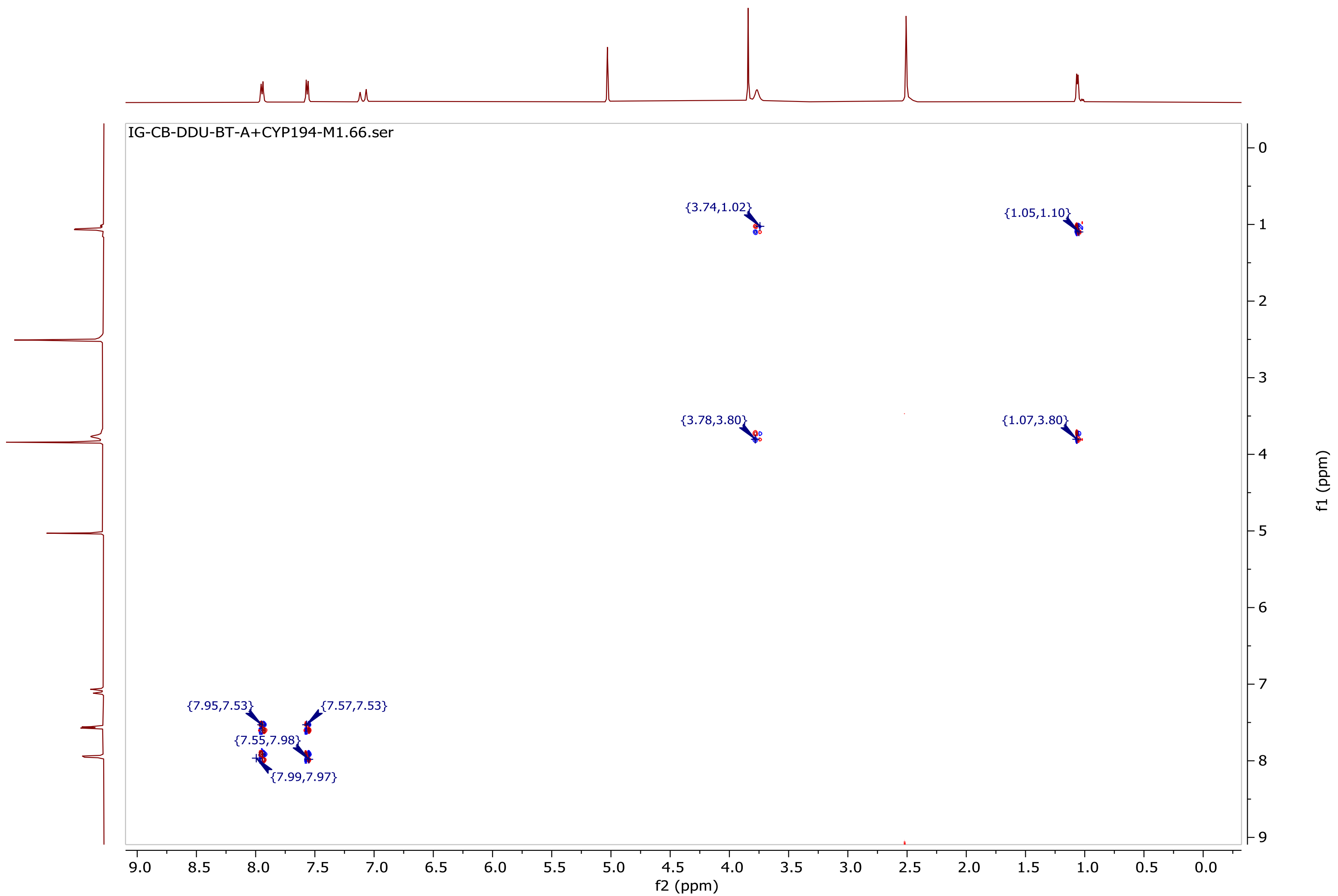

IG-CB-DDU-BT-A+CYP194-M1.66.ser
{3.74,1.02}
{1.05,1.10}
{3.78,3.80}
{1.07,3.80}
{7.95,7.53}
{7.57,7.53}
{7.55,7.98}
{7.99,7.97}
9.0
8.5
8.0
7.5
7.0
6.5
6.0
5.5
5.0
4.5
4.0
3.5
3.0
2.5
2.0
1.5
1.0
0.5
0.0
f2 (ppm)
0
1
2
3
4
5
6
7
8
9
f1 (ppm)

HSQC

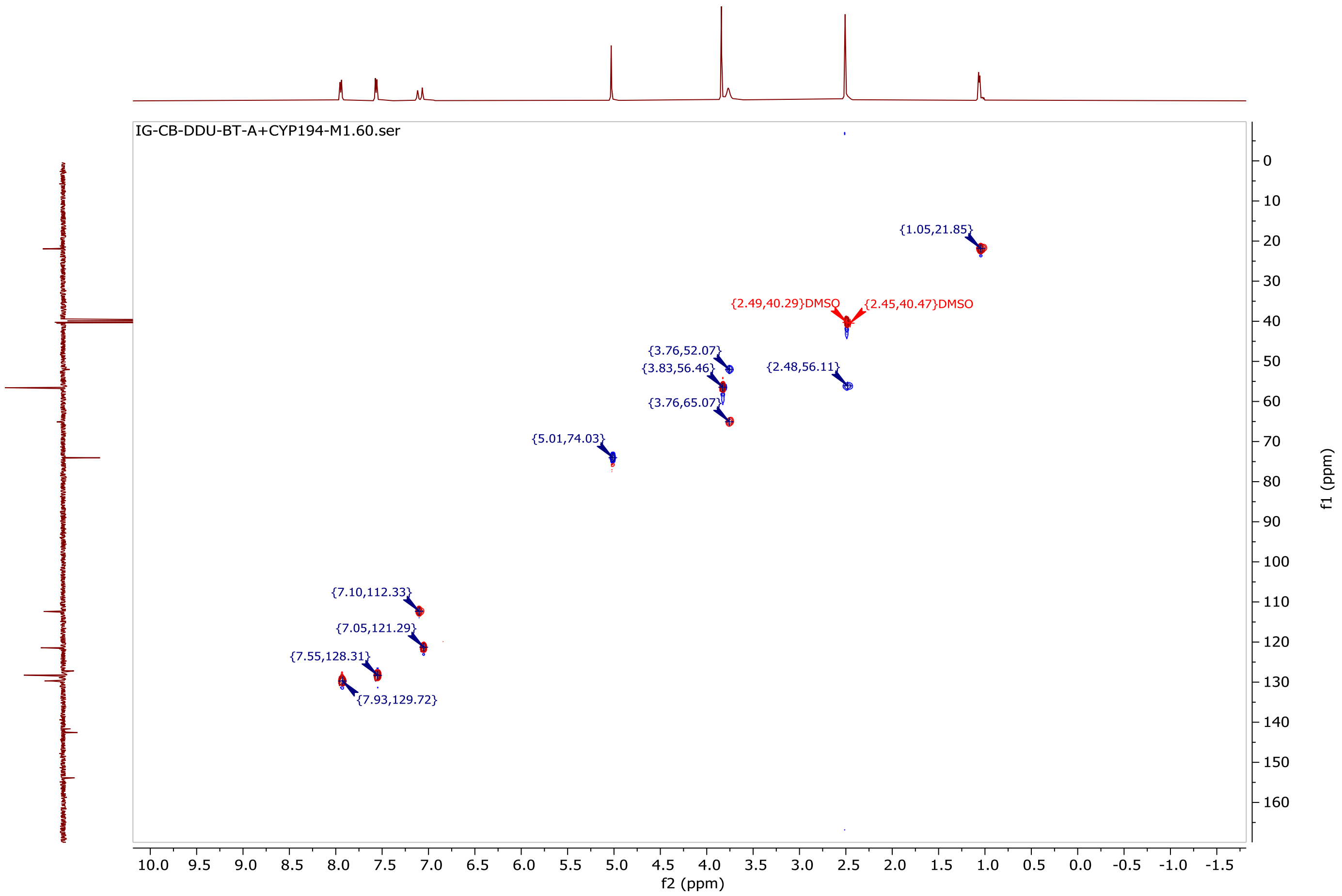
IG-CB-DDU-BT-A+CYP194-M1.60.ser
{1.05,21.85}
{2.49,40.29}DMSO
{2.45,40.47}DMSO
{3.76,52.07}
{3.83,56.46}
{2.48,56.11}
{3.76,65.07}
{5.01,74.03}
{7.10,112.33}
{7.05,121.29}
{7.55,128.31}
{7.93,129.72}
f2 (ppm)
f1 (ppm)

HMBC

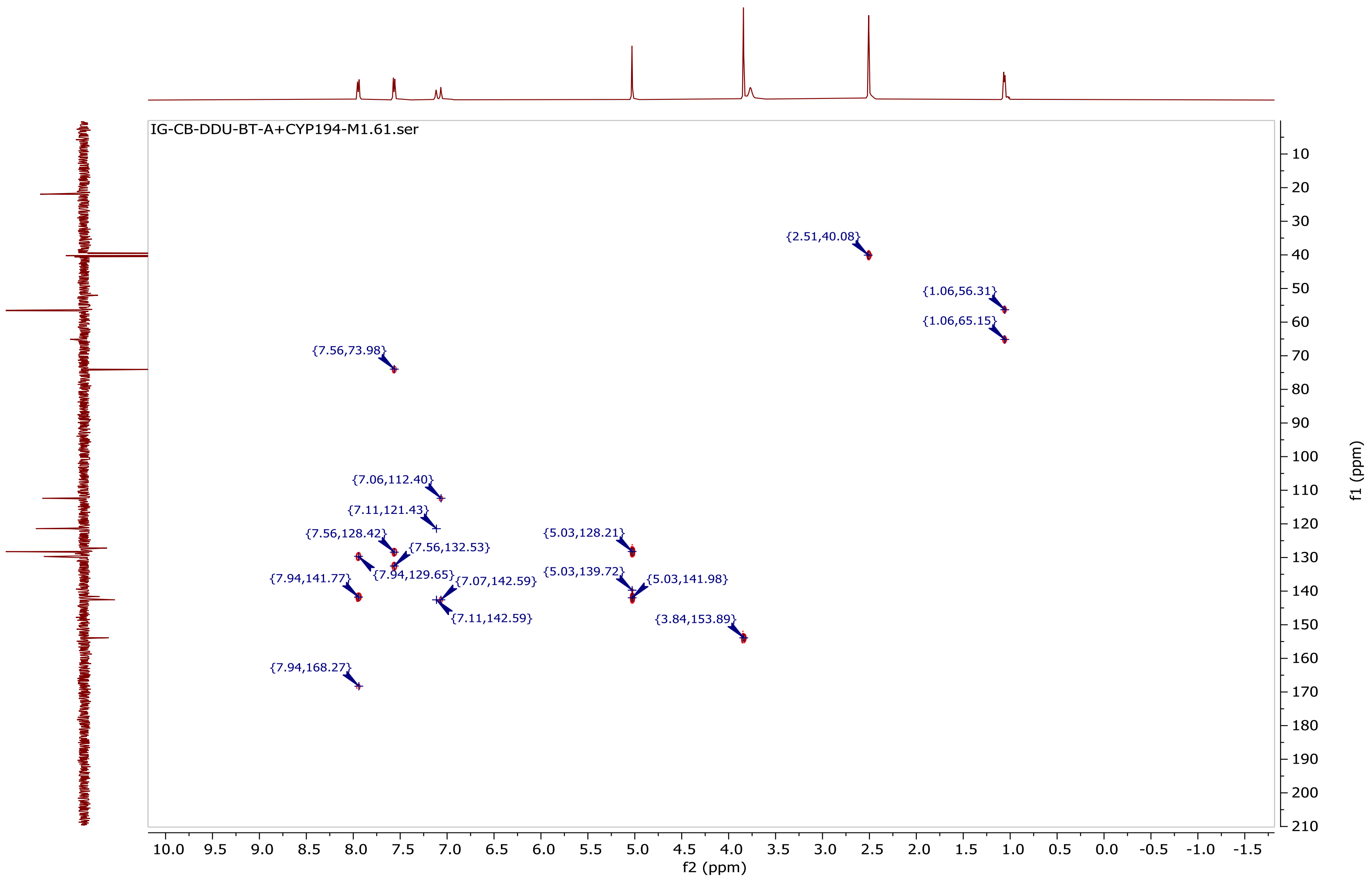
IG-CB-DDU-BT-A+CYP194-M1.61.ser
{2.51,40.08}
{1.06,56.31}
{1.06,65.15}
{7.56,73.98}
{7.06,112.40}
{7.11,121.43}
{7.56,128.42}
{7.56,132.53}
{5.03,128.21}
{7.94,129.65}
{5.03,139.72}
{5.03,141.98}
{7.94,141.77}
{7.07,142.59}
{7.11,142.59}
{3.84,153.89}
{7.94,168.27}
f2 (ppm)
f1 (ppm)

NOESY

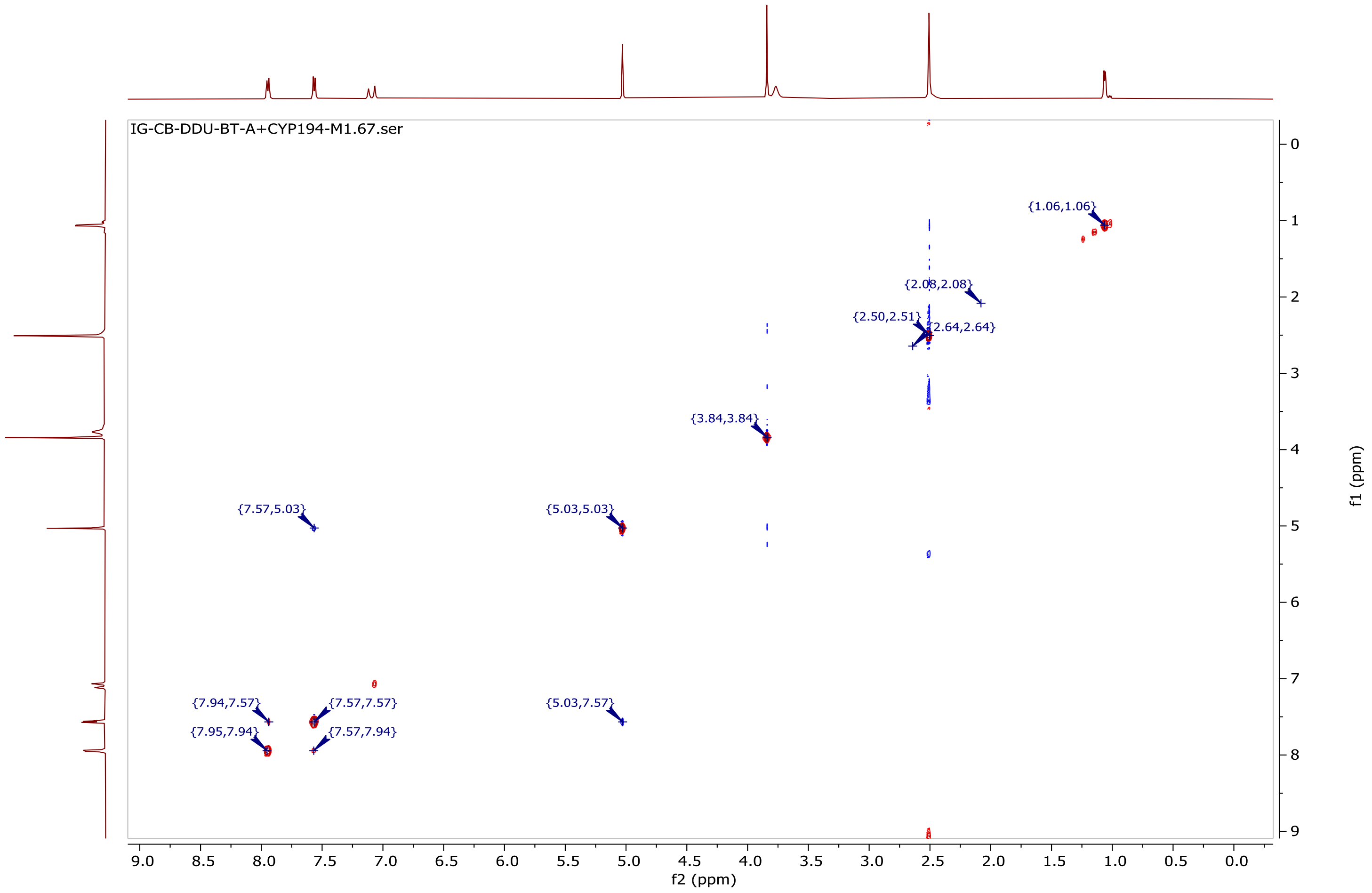

IG-CB-DDU-BT-A+CYP194-M1.67.ser
{1.06,1.06}
{2.08,2.08}
{2.50,2.51}
{2.64,2.64}
{3.84,3.84}
{7.57,5.03}
{5.03,5.03}
{7.94,7.57}
{7.57,7.57}
{5.03,7.57}
{7.95,7.94}
{7.57,7.94}
f2 (ppm)
f1 (ppm)

DEPTqgpsp

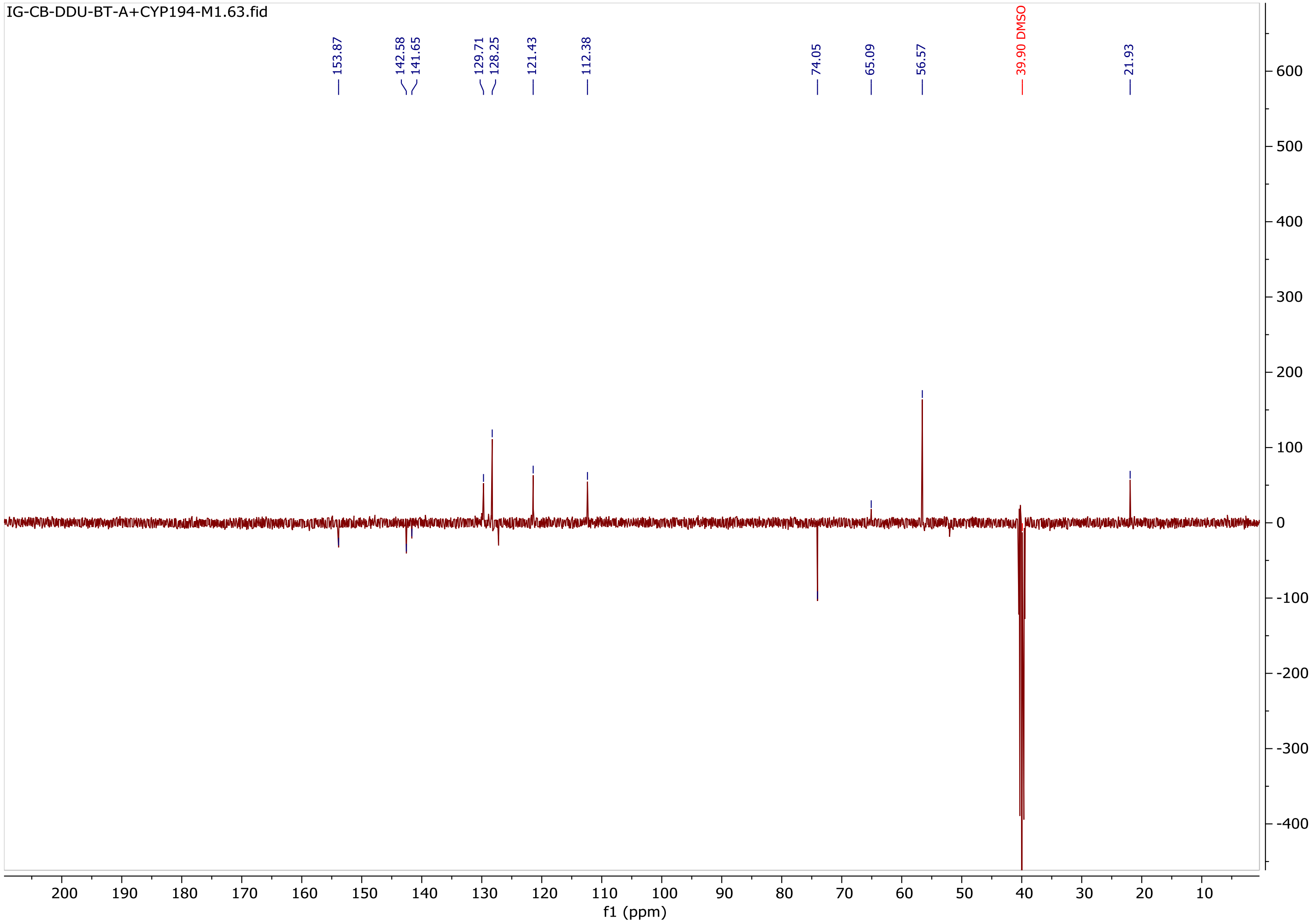
IG-CB-DDU-BT-A+CYP194-M1.63.fid
153.87
142.58
141.65
129.71
128.25
121.43
112.38
74.05
65.09
56.57
39.90 DMSO
21.93
600
500
400
300
200
100
0
-100
-200
-300
-400
200 190 180 170 160 150 140 130 120 110 100 90 80 70 60 50 40 30 20 10
f1 (ppm)

DEPT-90

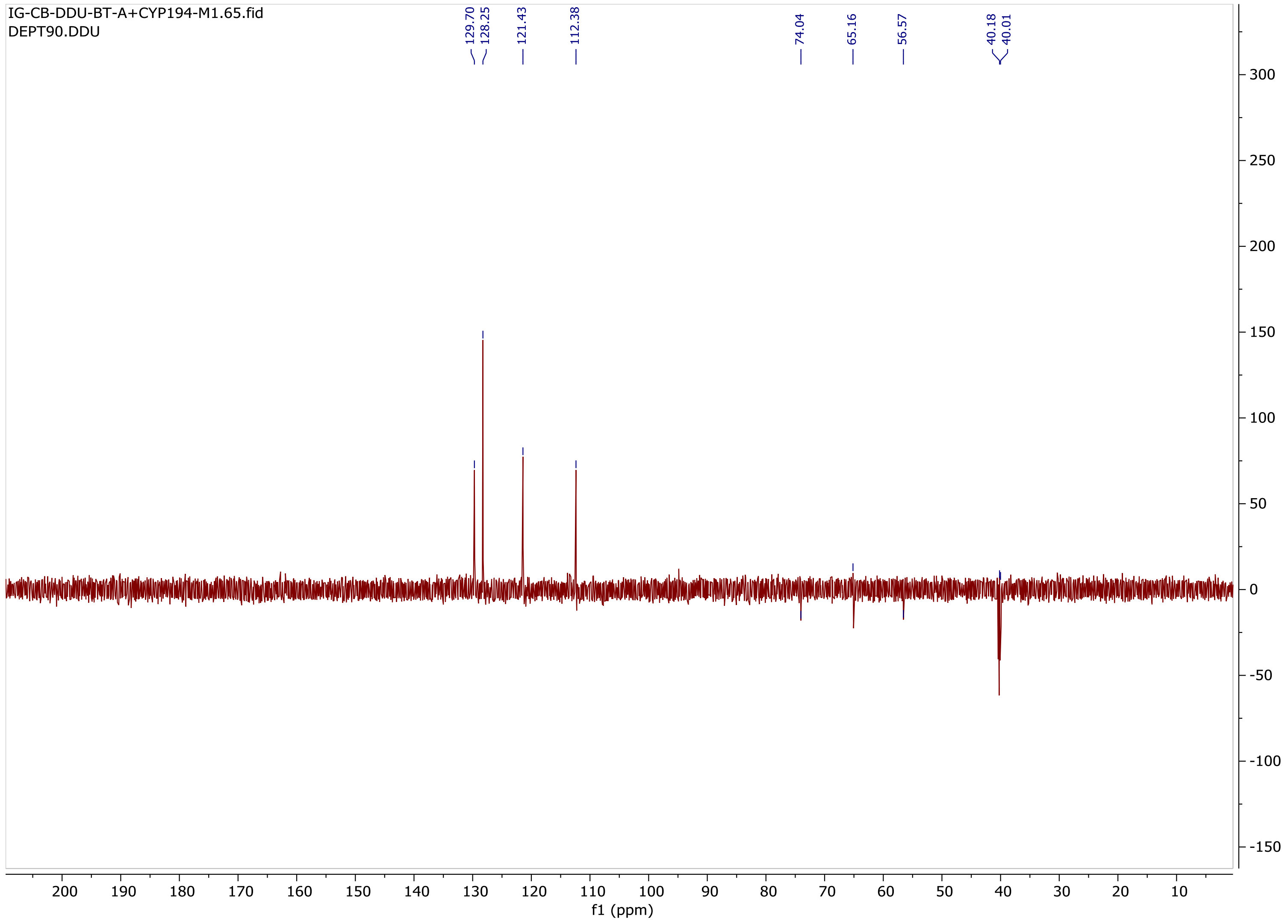
IG-CB-DDU-BT-A+CYP194-M1.65.fid
DEPT90.DDU
129.70
128.25
121.43
112.38
74.04
65.16
56.57
40.18
40.01
f1 (ppm)

DEPT-135

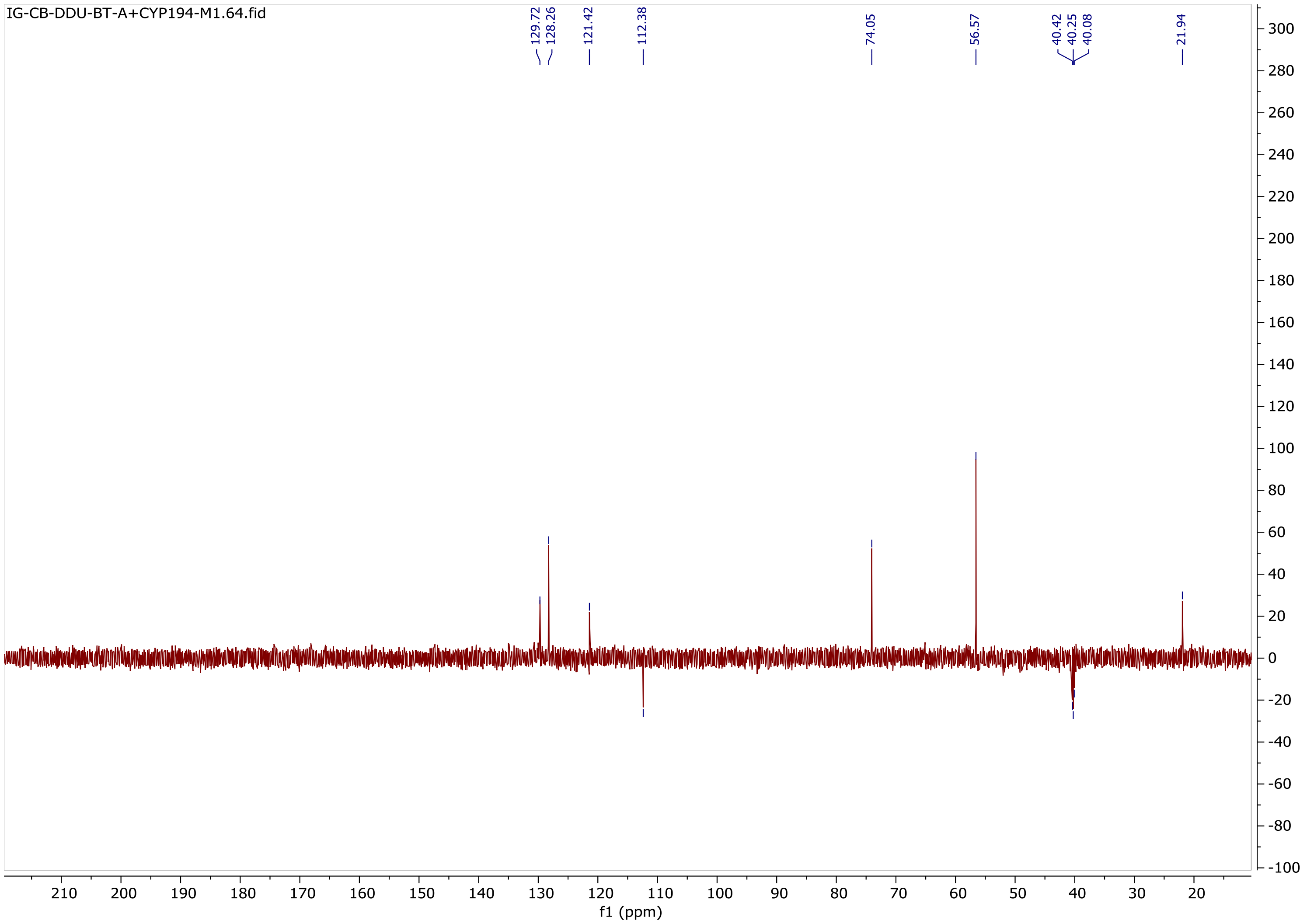

IG-CB-DDU-BT-A+CYP194-M1.64.fid
129.72
128.26
121.42
112.38
74.05
56.57
40.42
40.25
40.08
21.94
f1 (ppm)

HRMS

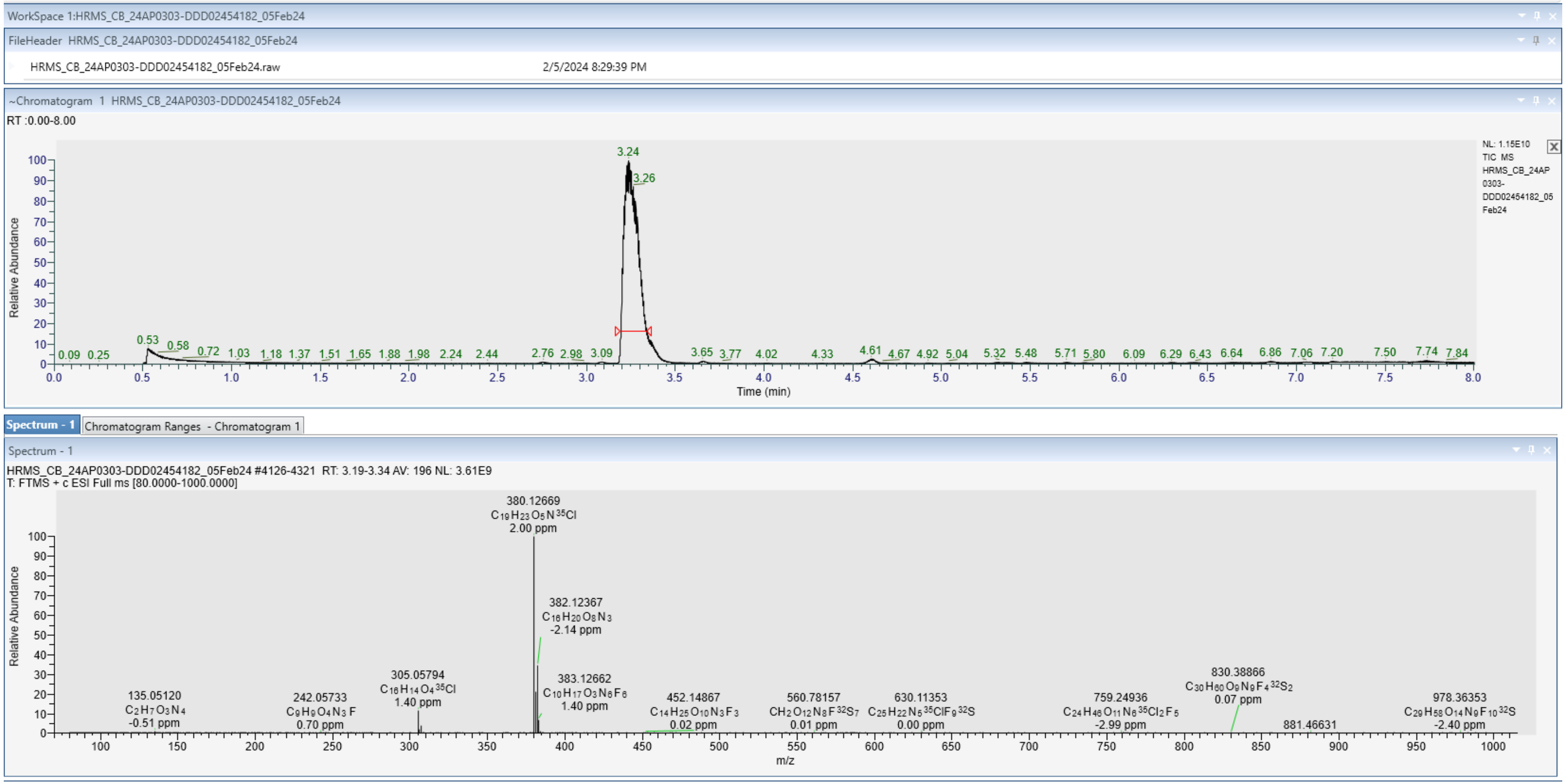
WorkSpace 1:HRMS_CB_24AP0303-DDD02454182_05Feb24
FileHeader HRMS_CB_24AP0303-DDD02454182_05Feb24
HRMS_CB_24AP0303-DDD02454182_05Feb24.raw
2/5/2024 8:29:39 PM
~Chromatogram 1 HRMS_CB_24AP0303-DDD02454182_05Feb24
RT :0.00-8.00
NL: 1.15E10
TIC MS
HRMS_CB_24AP0303-DDD02454182_05Feb24
Relative Abundance
Time (min)
Spectrum - 1
Chromatogram Ranges - Chromatogram 1
HRMS_CB_24AP0303-DDD02454182_05Feb24 #4126-4321 RT: 3.19-3.34 AV: 196 NL: 3.61E9
T: FTMS + c ESI Full ms [80.0000-1000.0000]
380.12669
C19 H23 O5 N 35Cl
2.00 ppm
382.12367
C16 H20 O8 N3
-2.14 ppm
383.12662
C10 H17 O3 N6 F6
1.40 ppm
305.05794
C16 H14 O4 35Cl
1.40 ppm
135.05120
C2 H7 O3 N4
-0.51 ppm
242.05733
C9 H9 O4 N3 F
0.70 ppm
452.14867
C14 H25 O10 N3 F3
0.02 ppm
560.78157
CH2 O12 N8 F 32S7
0.01 ppm
630.11353
C25 H22 N5 35ClF9 32S
0.00 ppm
759.24936
C24 H46 O11 N6 35Cl2 F5
-2.99 ppm
830.38866
C30 H60 O9 N9 F4 32S2
0.07 ppm
881.46631
978.36353
C29 H58 O14 N9 F10 32S
-2.40 ppm
m/z

Compound **5b**

^{1}H NMR

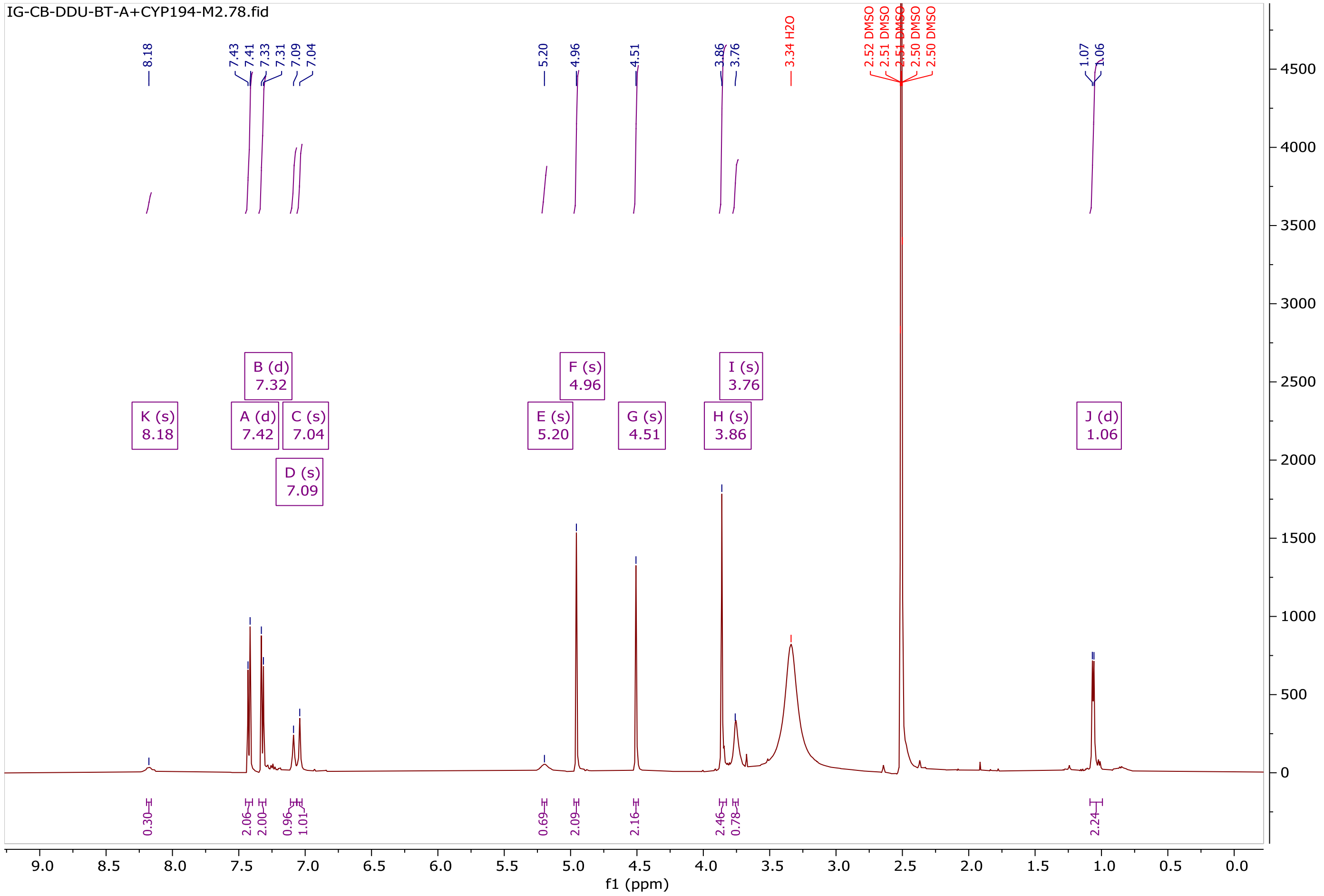

COSY

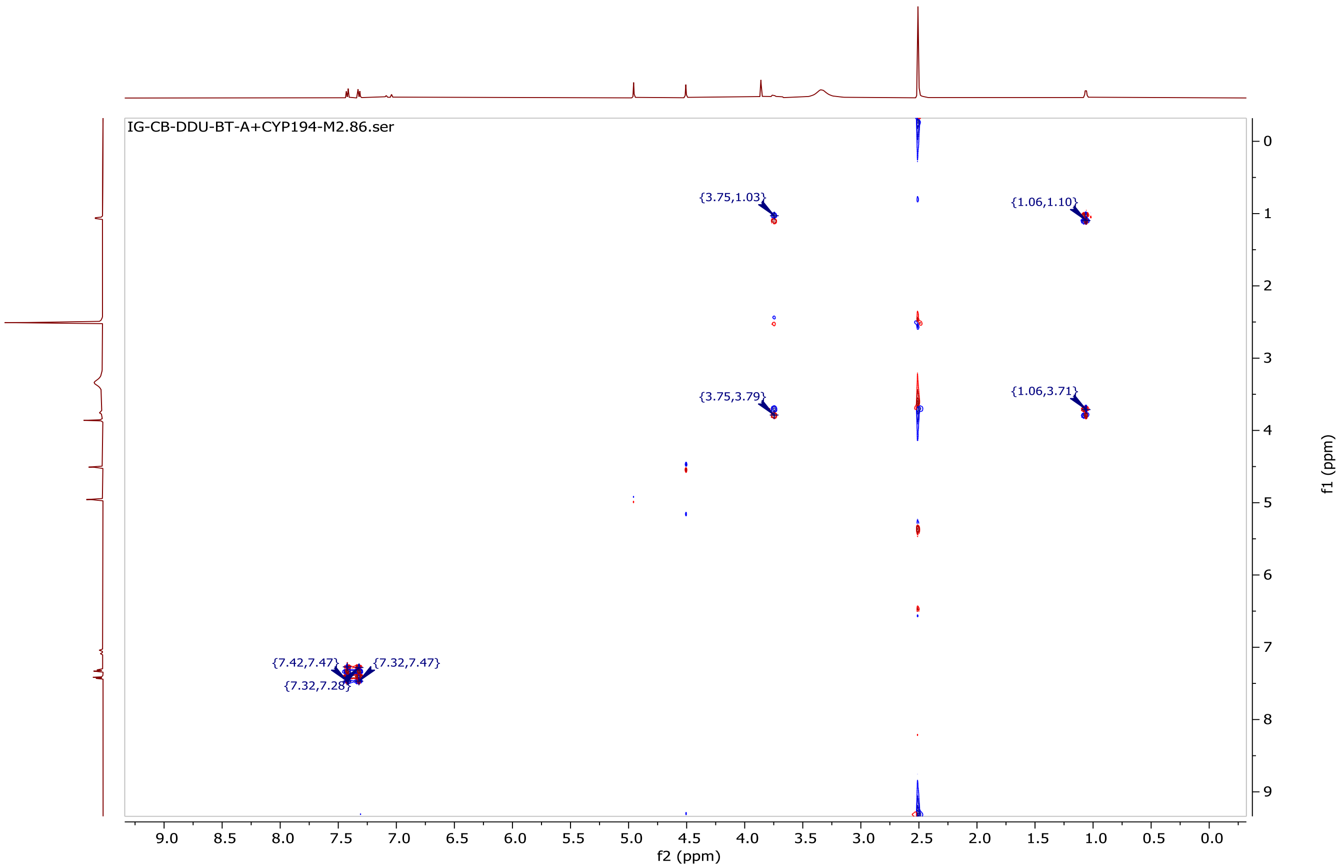
IG-CB-DDU-BT-A+CYP194-M2.86.ser
{3.75,1.03}
{1.06,1.10}
{3.75,3.79}
{1.06,3.71}
{7.42,7.47}
{7.32,7.47}
{7.32,7.28}
f2 (ppm)
f1 (ppm)
9.0 8.5 8.0 7.5 7.0 6.5 6.0 5.5 5.0 4.5 4.0 3.5 3.0 2.5 2.0 1.5 1.0 0.5 0.0
0 1 2 3 4 5 6 7 8 9

HSQC

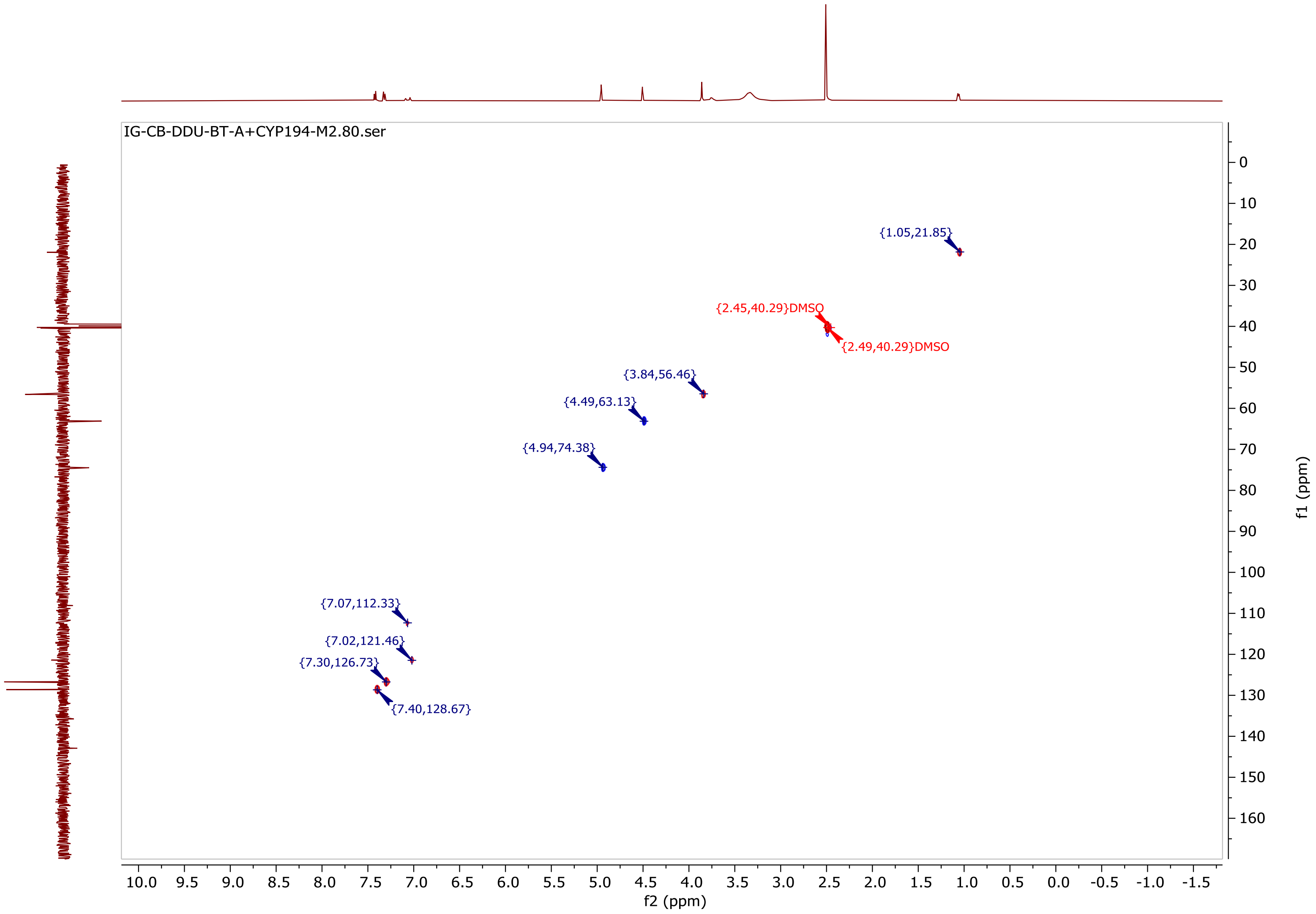
IG-CB-DDU-BT-A+CYP194-M2.80.ser
{1.05,21.85}
{2.45,40.29}DMSO
{2.49,40.29}DMSO
{3.84,56.46}
{4.49,63.13}
{4.94,74.38}
{7.07,112.33}
{7.02,121.46}
{7.30,126.73}
{7.40,128.67}
f2 (ppm)
f1 (ppm)

HMBC

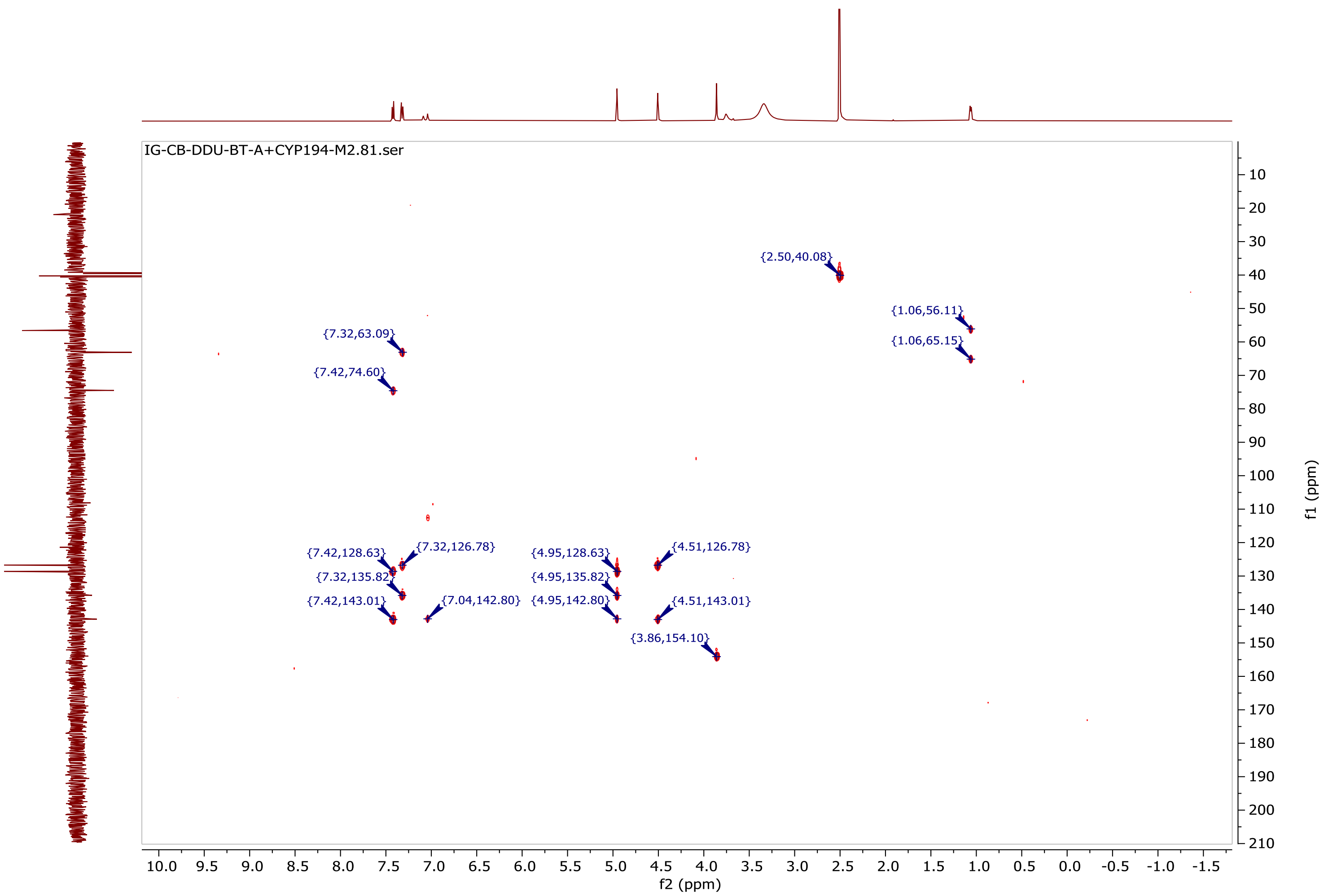

IG-CB-DDU-BT-A+CYP194-M2.81.ser
{2.50,40.08}
{1.06,56.11}
{1.06,65.15}
{7.32,63.09}
{7.42,74.60}
{7.42,128.63}
{7.32,126.78}
{7.32,135.82}
{7.42,143.01}
{7.04,142.80}
{4.95,128.63}
{4.95,135.82}
{4.95,142.80}
{4.51,126.78}
{4.51,143.01}
{3.86,154.10}
f2 (ppm)
f1 (ppm)

NOESY

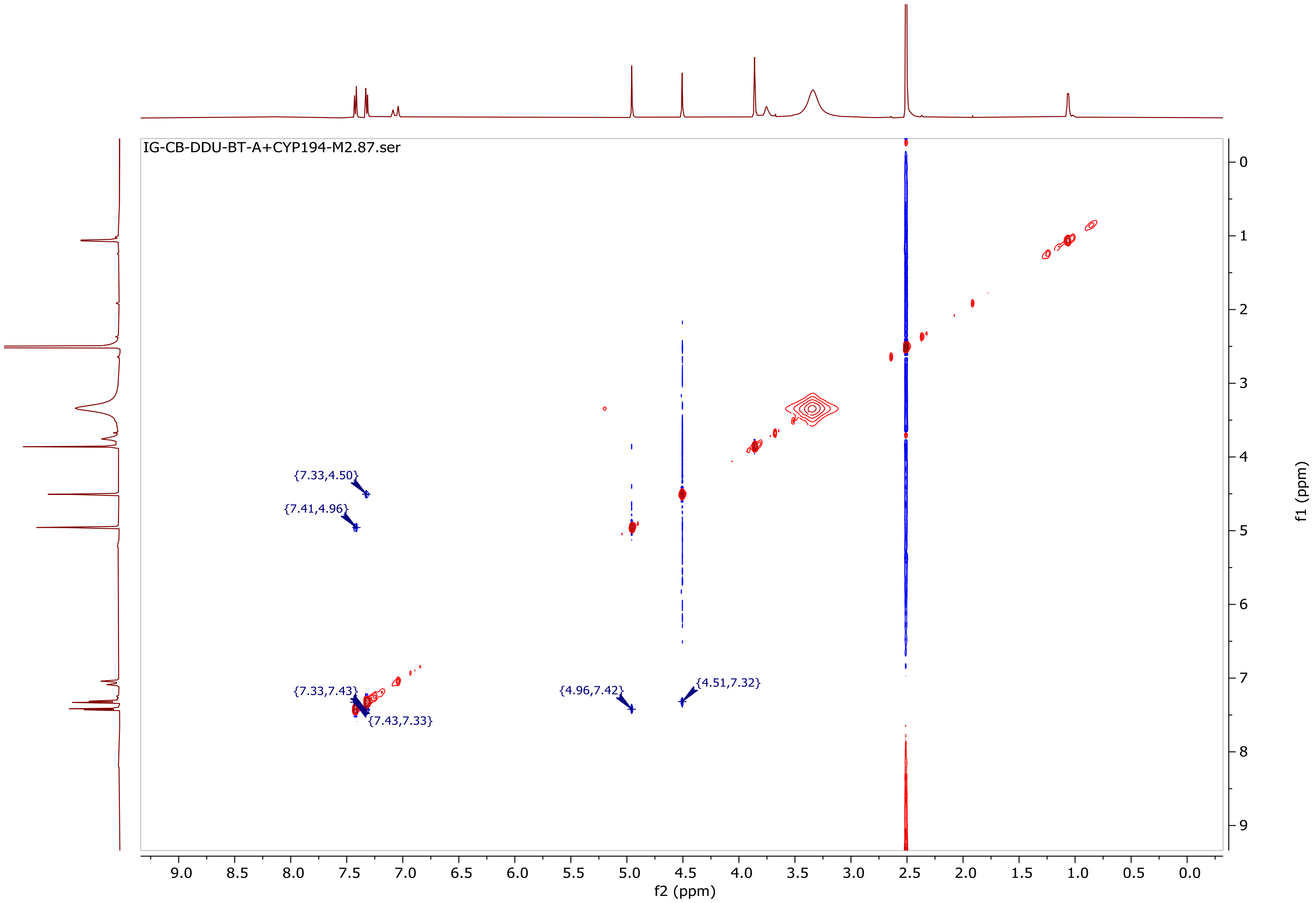

IG-CB-DDU-BT-A+CYP194-M2.87.ser
{7.33,4.50}
{7.41,4.96}
{7.33,7.43}
{7.43,7.33}
{4.96,7.42}
{4.51,7.32}
9.0
8.5
8.0
7.5
7.0
6.5
6.0
5.5
5.0
4.5
4.0
3.5
3.0
2.5
2.0
1.5
1.0
0.5
0.0
f2 (ppm)
f1 (ppm)

DEPTqgpsp

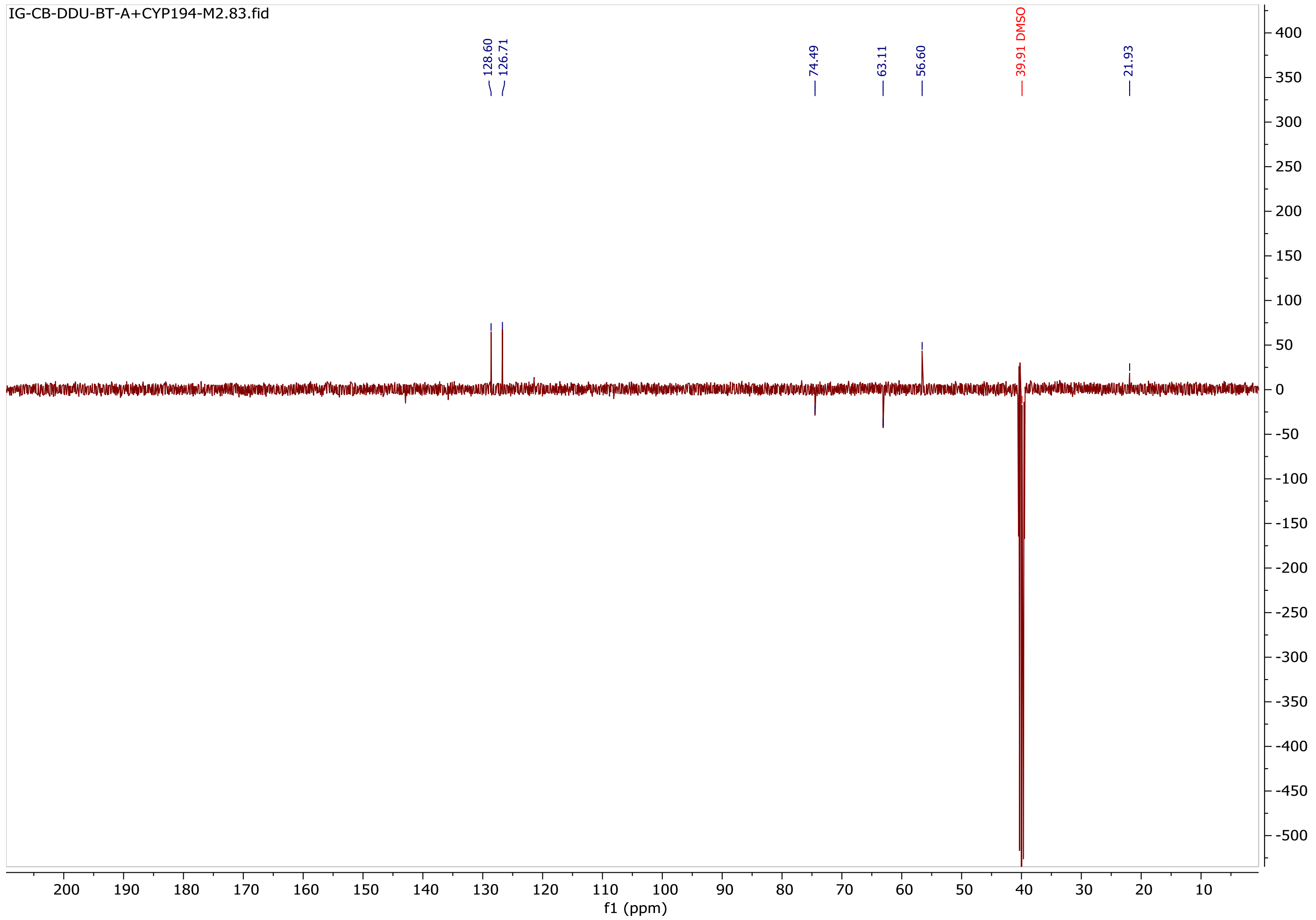
IG-CB-DDU-BT-A+CYP194-M2.83.fid
128.60
126.71
74.49
63.11
56.60
39.91 DMSO
21.93
f1 (ppm)

DEPT-90

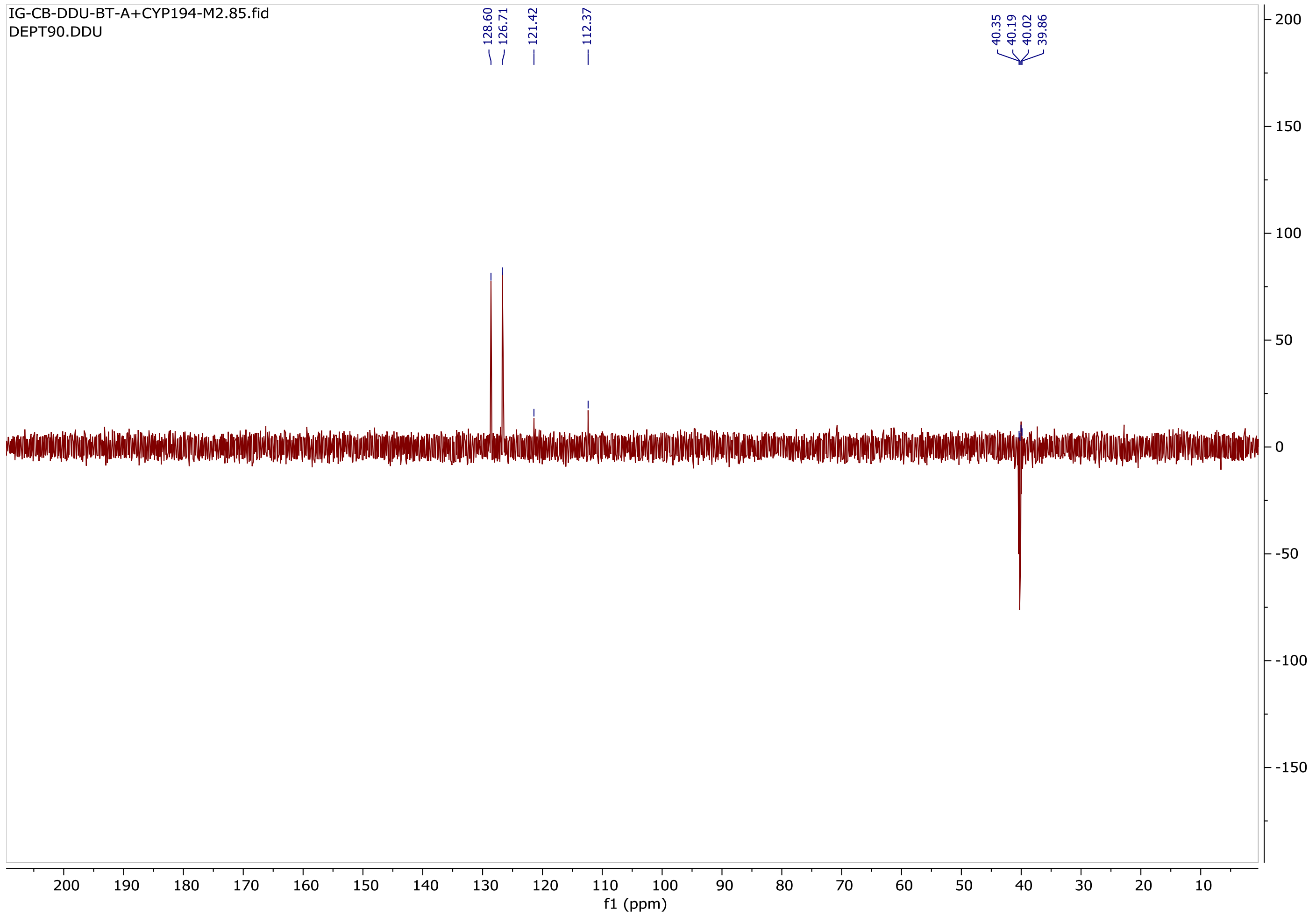

IG-CB-DDU-BT-A+CYP194-M2.85.fid
DEPT90.DDU
128.60
126.71
121.42
112.37
40.35
40.19
40.02
39.86
200
150
100
50
0
-50
-100
-150
200 190 180 170 160 150 140 130 120 110 100 90 80 70 60 50 40 30 20 10
f1 (ppm)

DEPT-135

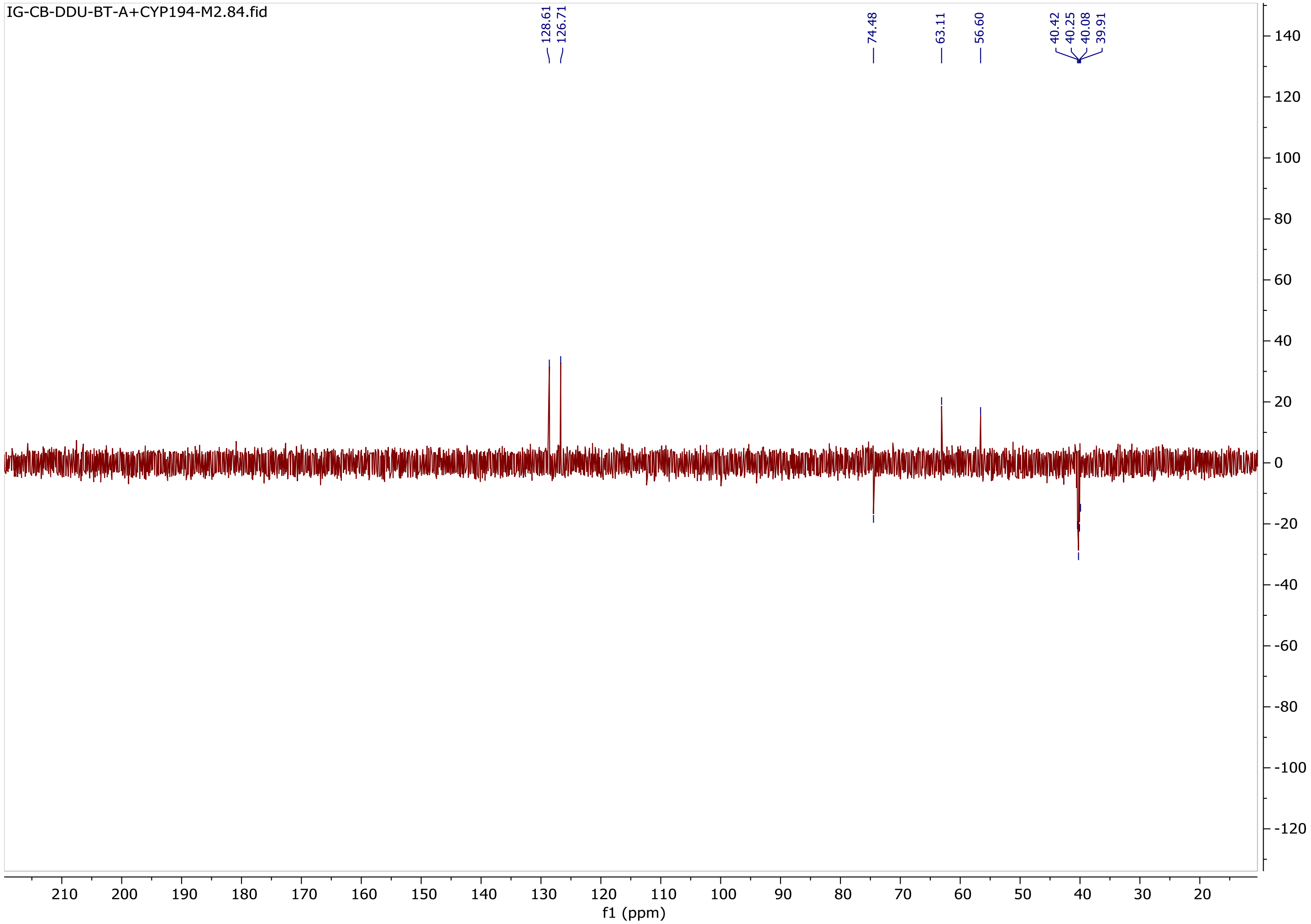

IG-CB-DDU-BT-A+CYP194-M2.84.fid
128.61
126.71
74.48
63.11
56.60
40.42
40.25
40.08
39.91
140
120
100
80
60
40
20
0
-20
-40
-60
-80
-100
-120
210
200
190
180
170
160
150
140
130
120
110
100
90
80
70
60
50
40
30
20
f1 (ppm)

HRMS

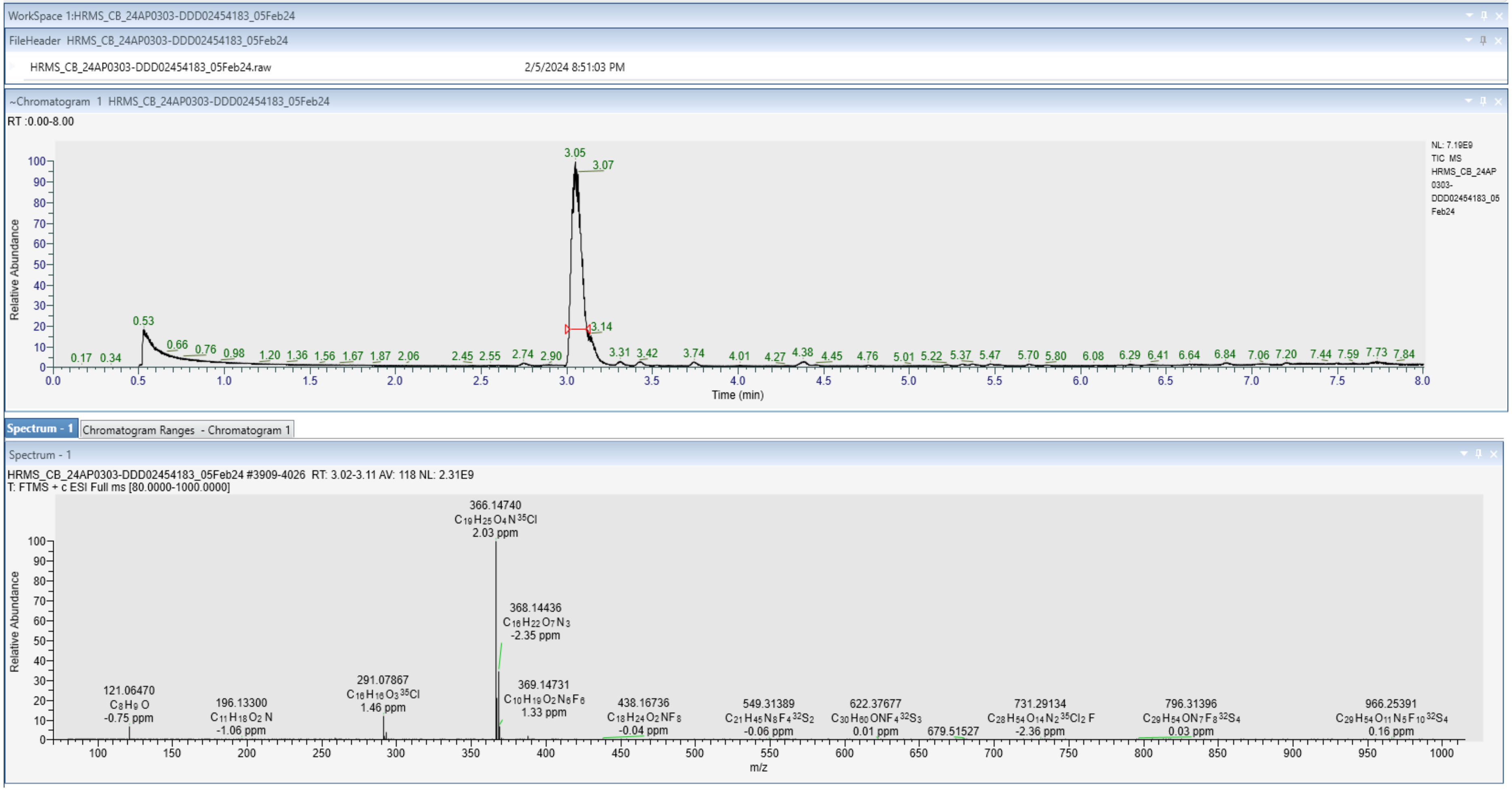

Compound **5e**

^{1}H NMR

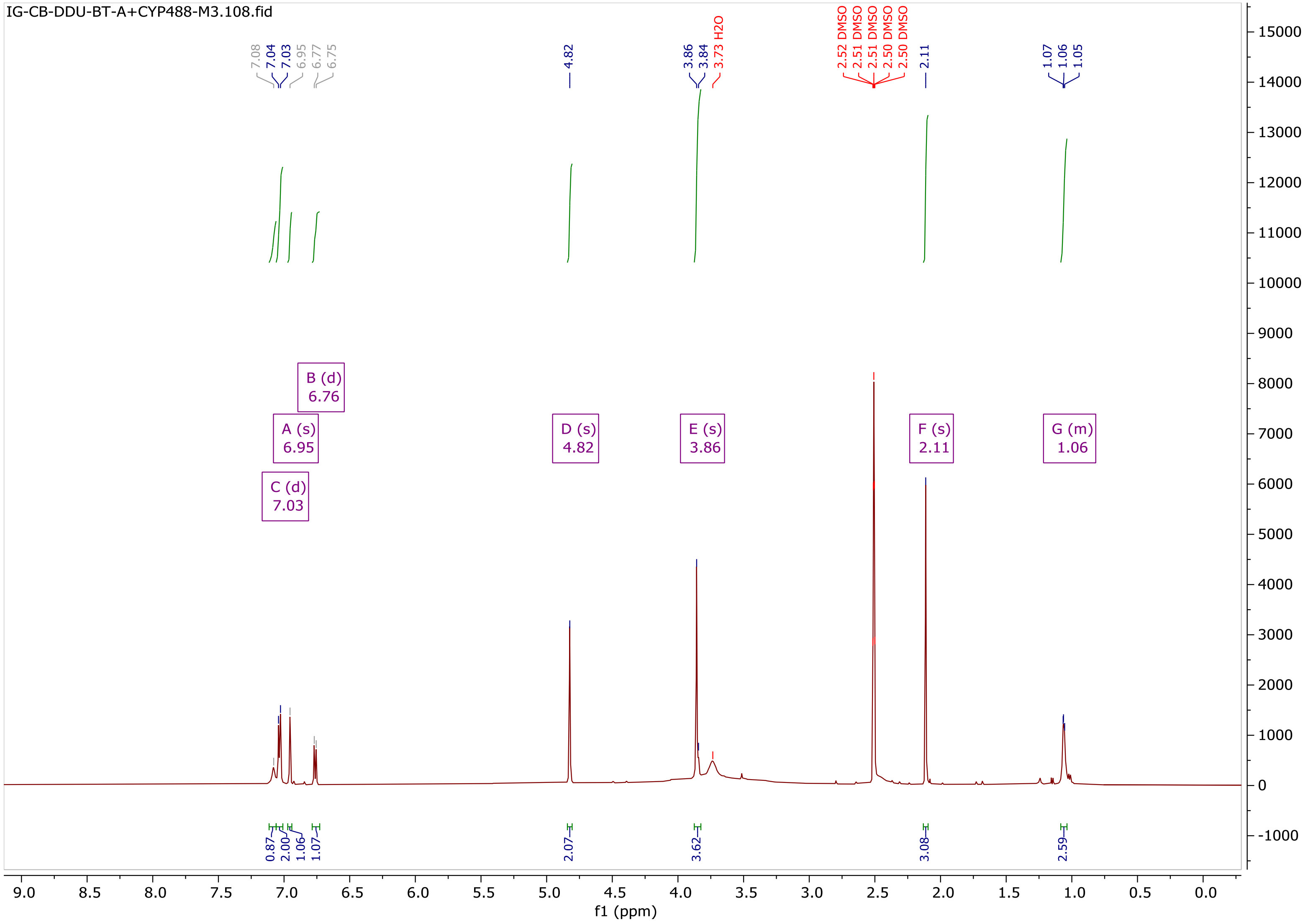
IG-CB-DDU-BT-A+CYP488-M3.108.fid
7.08
7.04
7.03
6.95
6.77
6.75
4.82
3.86
3.84
3.73 H2O
2.52 DMSO
2.51 DMSO
2.51 DMSO
2.50 DMSO
2.50 DMSO
2.11
1.07
1.06
1.05
B (d)
6.76
A (s)
6.95
C (d)
7.03
D (s)
4.82
E (s)
3.86
F (s)
2.11
G (m)
1.06
0.87
2.00
1.06
1.07
2.07
3.62
3.08
2.59
f1 (ppm)

COSY

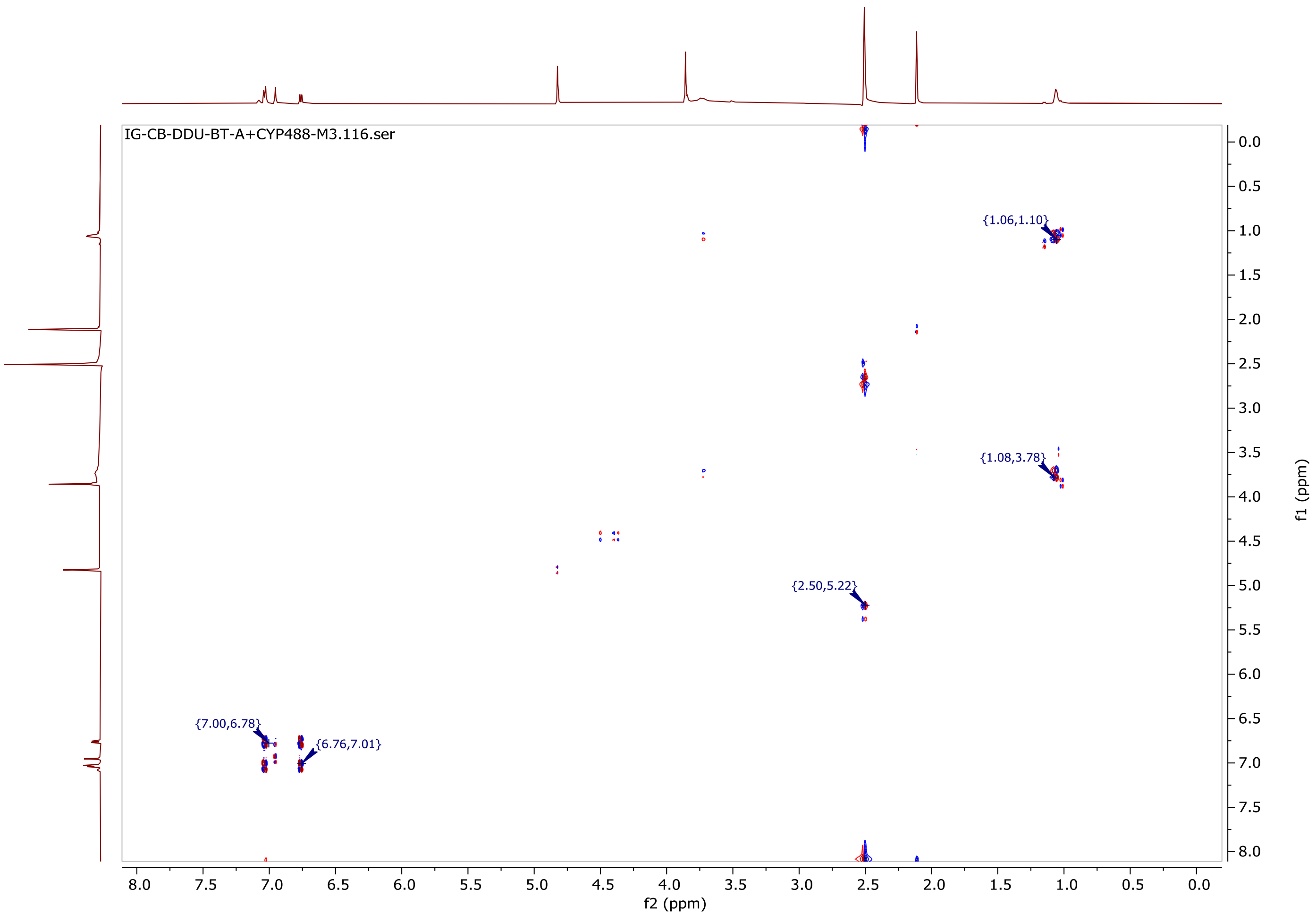

IG-CB-DDU-BT-A+CYP488-M3.116.ser
{1.06,1.10}
{1.08,3.78}
{2.50,5.22}
{7.00,6.78}
{6.76,7.01}
f2 (ppm)
f1 (ppm)

HSQC

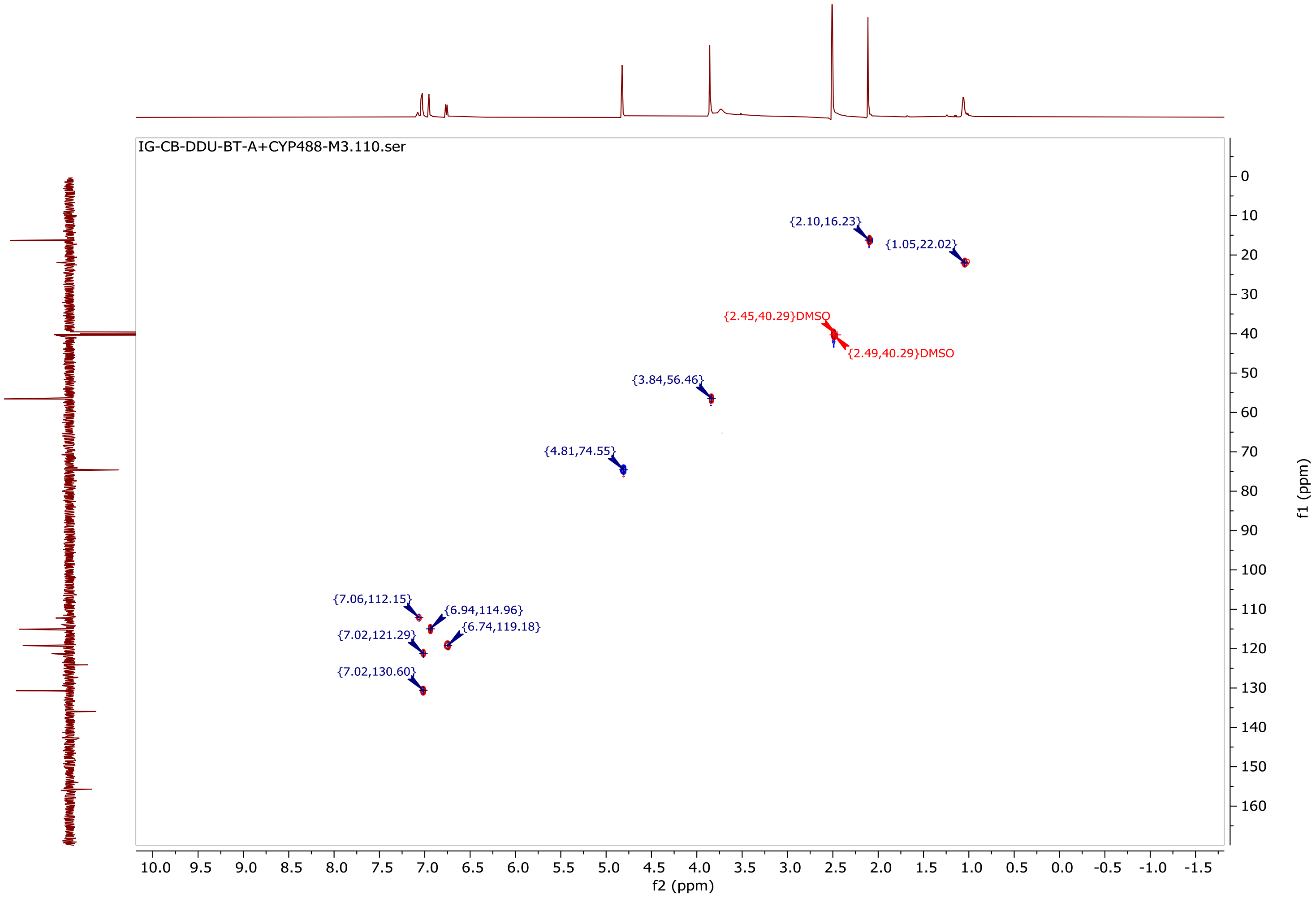

IG-CB-DDU-BT-A+CYP488-M3.110.ser
{2.10,16.23}
{1.05,22.02}
{2.45,40.29}DMSO
{2.49,40.29}DMSO
{3.84,56.46}
{4.81,74.55}
{7.06,112.15}
{6.94,114.96}
{6.74,119.18}
{7.02,121.29}
{7.02,130.60}
f2 (ppm)
f1 (ppm)

HMBC

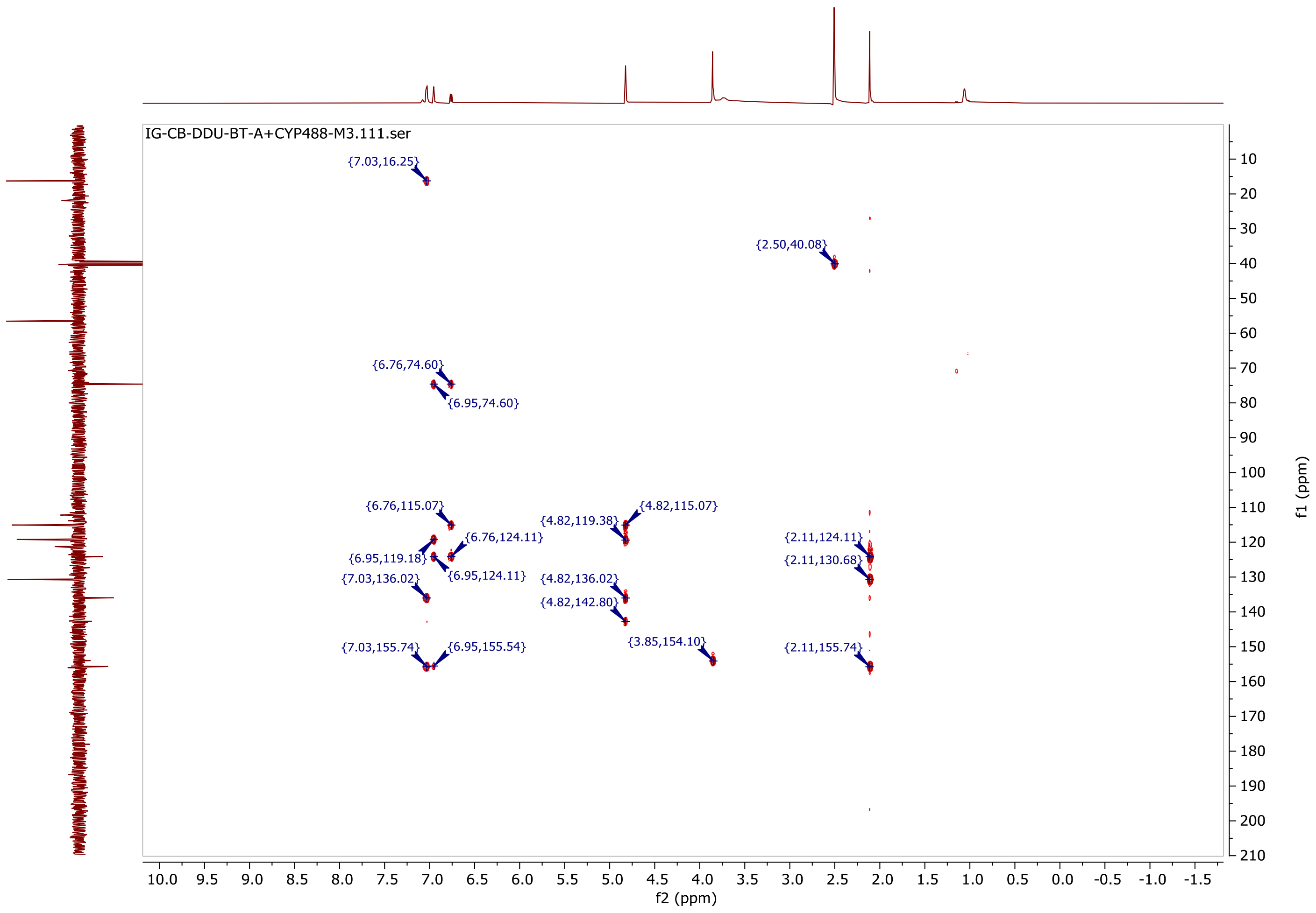

IG-CB-DDU-BT-A+CYP488-M3.111.ser
{7.03,16.25}
{2.50,40.08}
{6.76,74.60}
{6.95,74.60}
{6.76,115.07}
{4.82,119.38}
{4.82,115.07}
{6.76,124.11}
{2.11,124.11}
{6.95,119.18}
{2.11,130.68}
{6.95,124.11}
{7.03,136.02}
{4.82,136.02}
{4.82,142.80}
{3.85,154.10}
{7.03,155.74}
{6.95,155.54}
{2.11,155.74}
f2 (ppm)
f1 (ppm)

NOESY

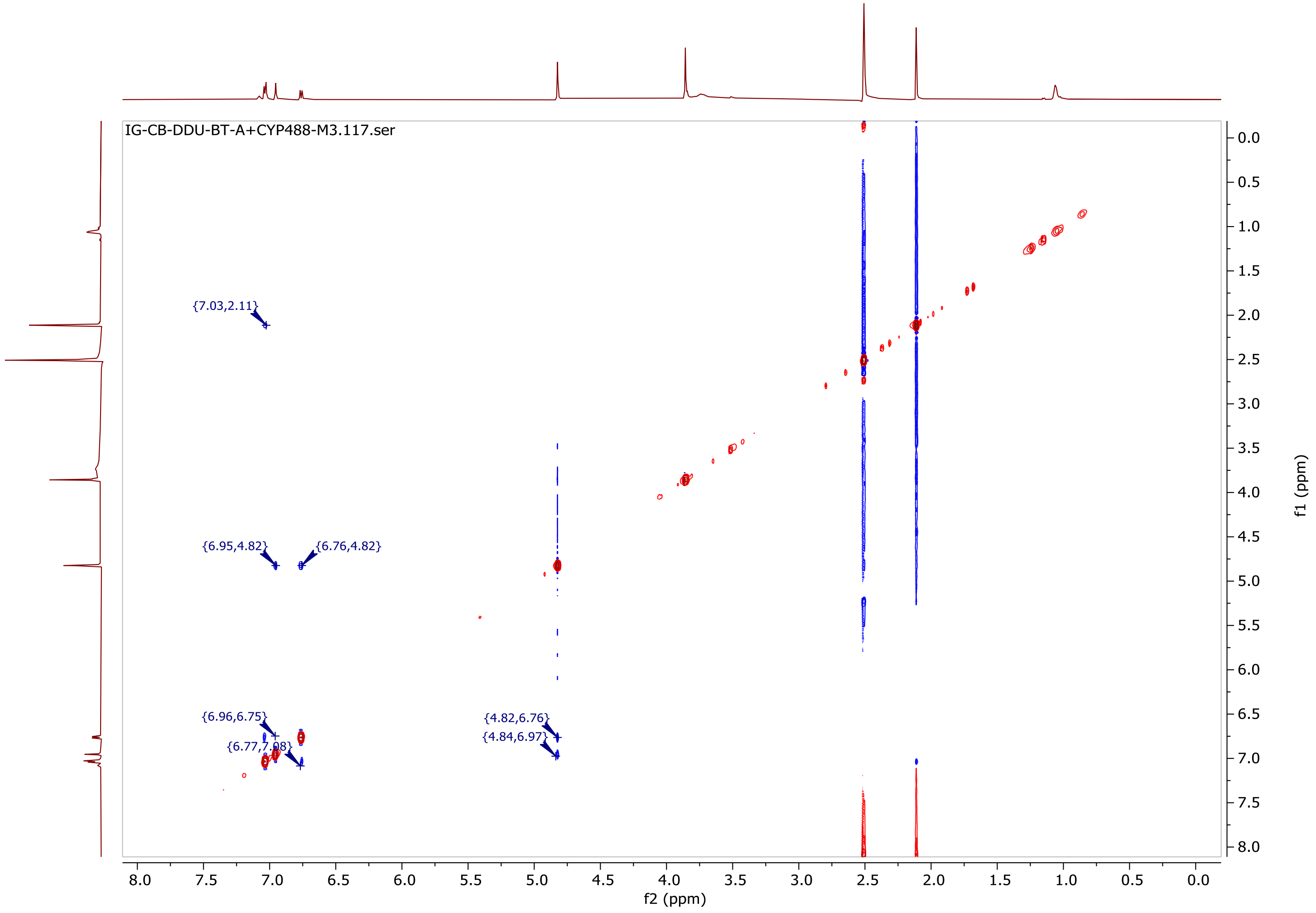

IG-CB-DDU-BT-A+CYP488-M3.117.ser
{7.03,2.11}
{6.95,4.82}
{6.76,4.82}
{6.96,6.75}
{6.77,7.08}
{4.82,6.76}
{4.84,6.97}
f2 (ppm)
f1 (ppm)

DEPTqgpsp

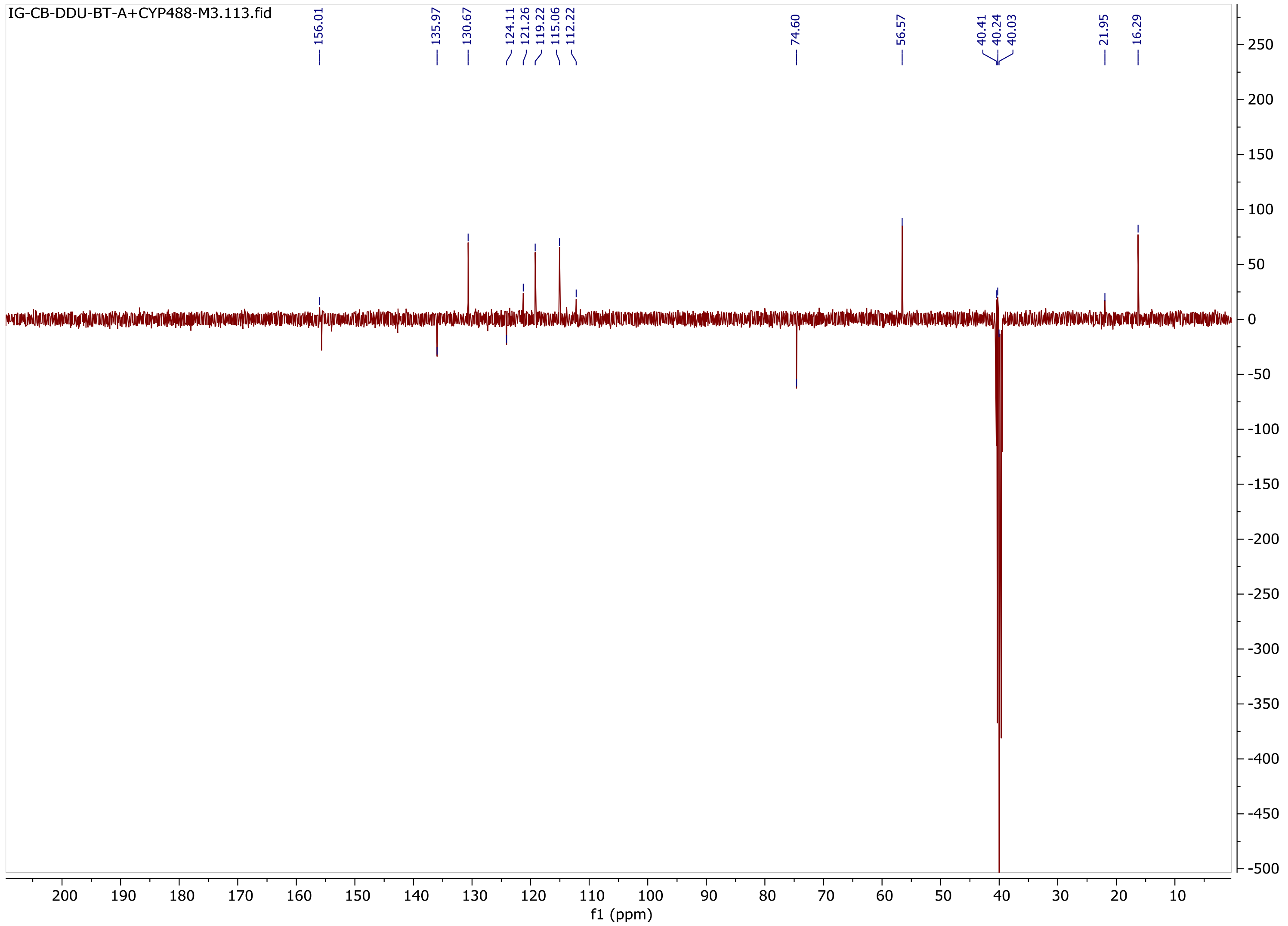
IG-CB-DDU-BT-A+CYP488-M3.113.fid
156.01
135.97
130.67
124.11
121.26
119.22
115.06
112.22
74.60
56.57
40.41
40.24
40.03
21.95
16.29
f1 (ppm)

DEPT-90

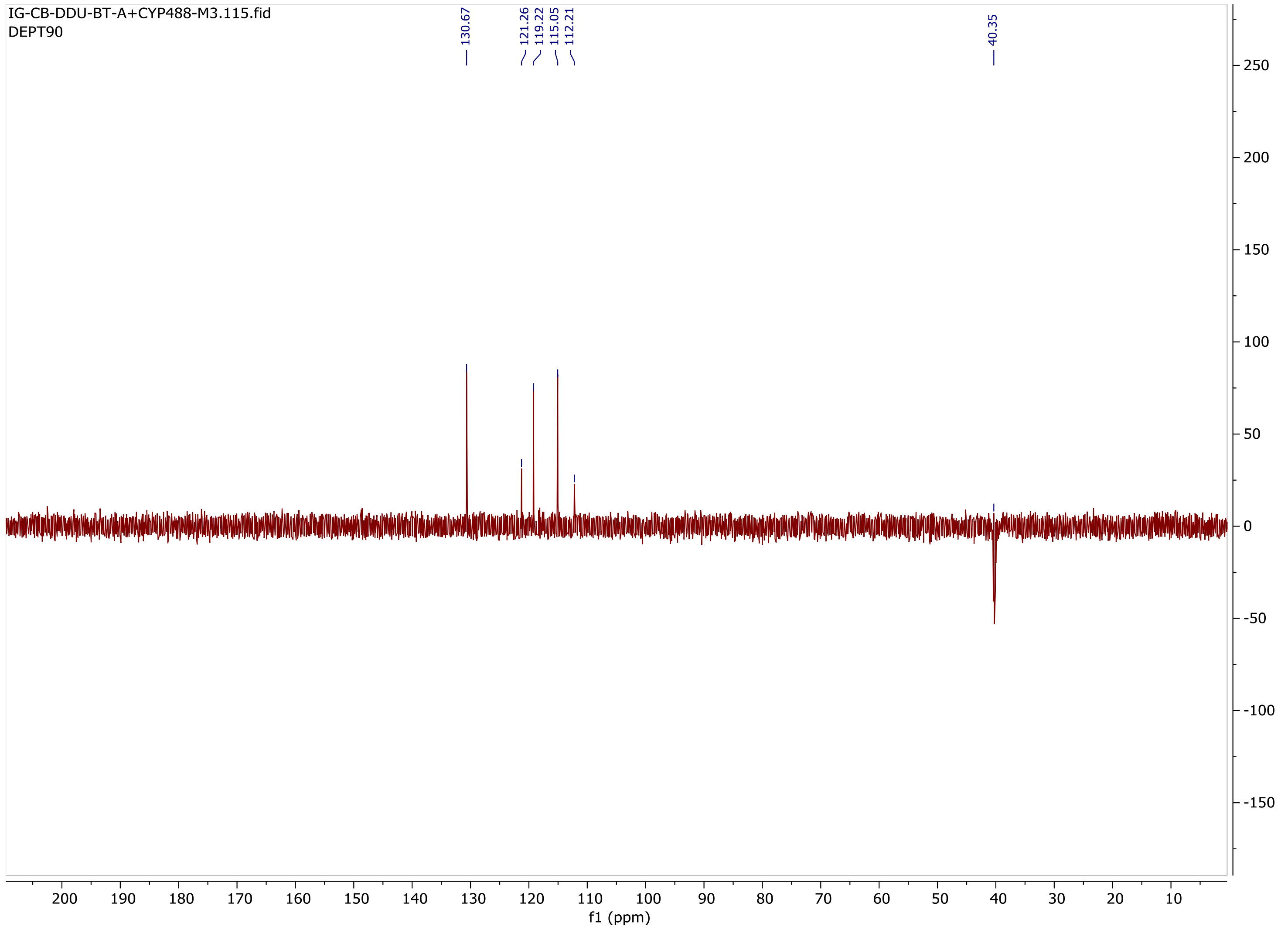
IG-CB-DDU-BT-A+CYP488-M3.115.fid
DEPT90
130.67
121.26
119.22
115.05
112.21
40.35
250
200
150
100
50
0
-50
-100
-150
200
190
180
170
160
150
140
130
120
110
100
90
80
70
60
50
40
30
20
10
f1 (ppm)

DEPT-135

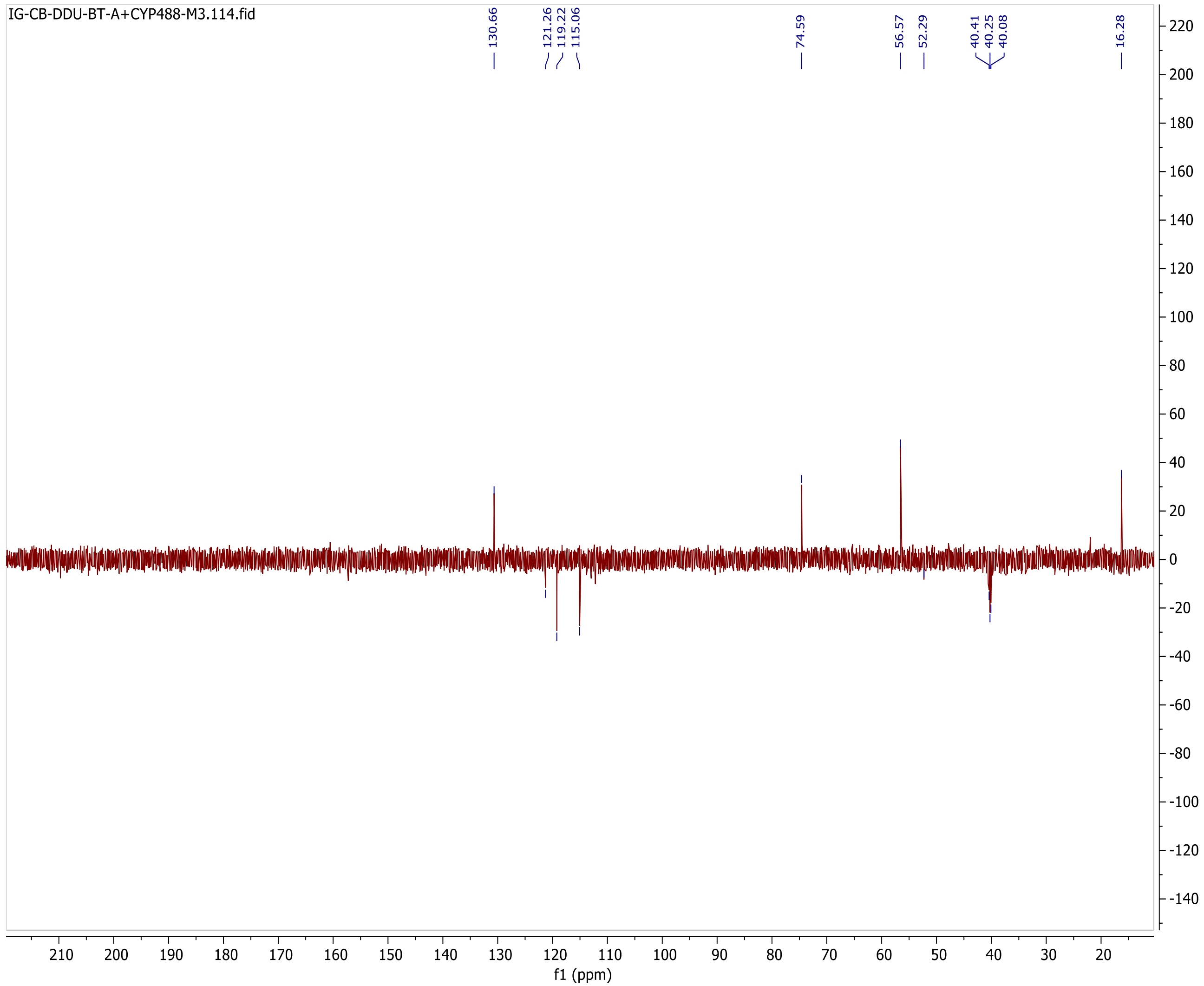

IG-CB-DDU-BT-A+CYP488-M3.114.fid
130.66
121.26
119.22
115.06
74.59
56.57
52.29
40.41
40.25
40.08
16.28
f1 (ppm)

HRMS

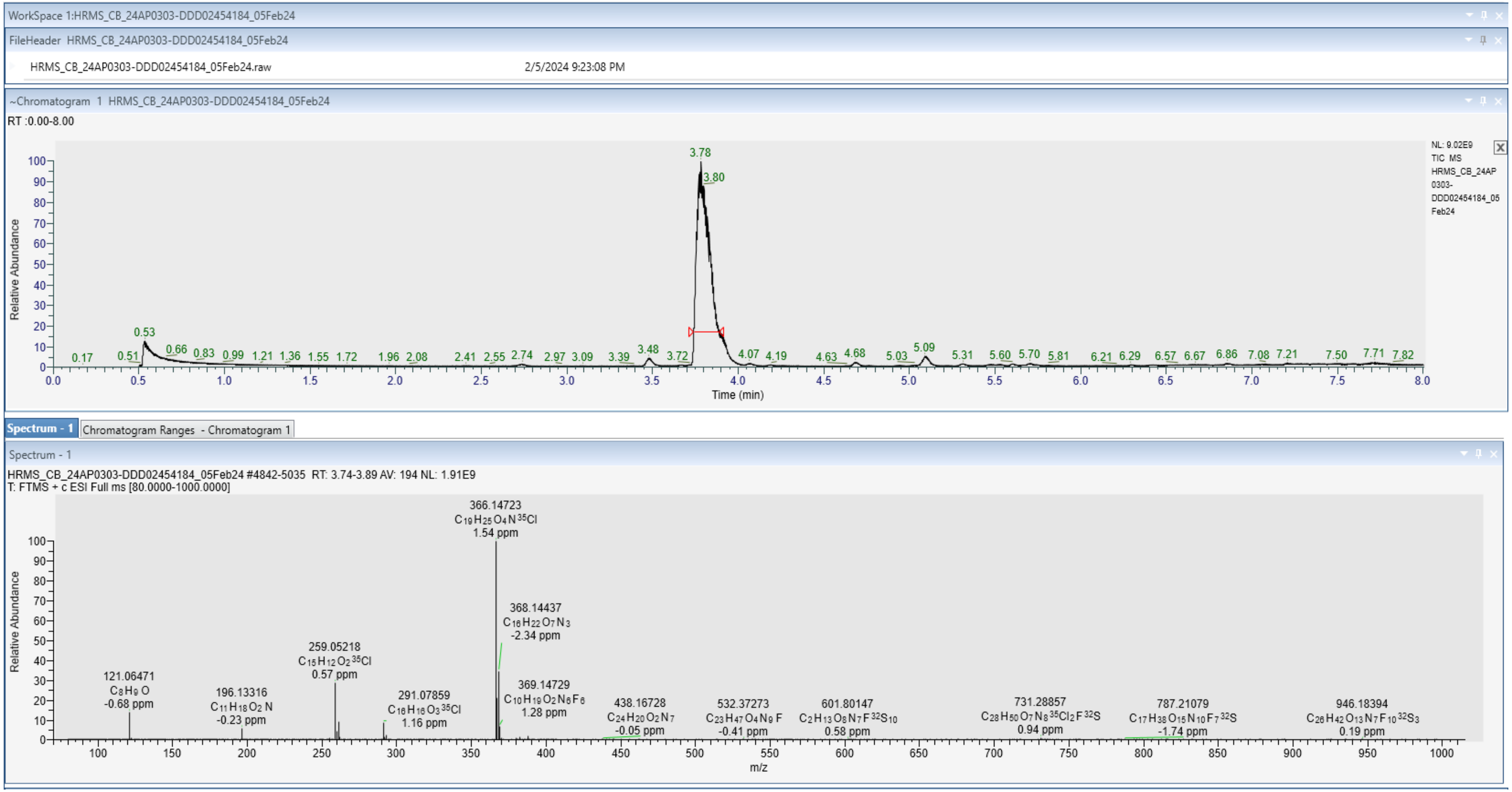
WorkSpace 1:HRMS_CB_24AP0303-DDD02454184_05Feb24
FileHeader HRMS_CB_24AP0303-DDD02454184_05Feb24
HRMS_CB_24AP0303-DDD02454184_05Feb24.raw
2/5/2024 9:23:08 PM
~Chromatogram 1 HRMS_CB_24AP0303-DDD02454184_05Feb24
RT :0.00-8.00
NL: 9.02E9
TIC MS
HRMS_CB_24AP0303-DDD02454184_05Feb24
Relative Abundance
Time (min)
Spectrum - 1
Chromatogram Ranges - Chromatogram 1
HRMS_CB_24AP0303-DDD02454184_05Feb24 #4842-5035 RT: 3.74-3.89 AV: 194 NL: 1.91E9
T: FTMS + c ESI Full ms [80.0000-1000.0000]
366.14723
C19H25O4N35Cl
1.54 ppm
368.14437
C18H22O7N3
-2.34 ppm
369.14729
C10H19O2N6F6
1.28 ppm
121.06471
C8H9O
-0.68 ppm
196.13316
C11H18O2N
-0.23 ppm
259.05218
C15H12O2 35Cl
0.57 ppm
291.07859
C16H16O3 35Cl
1.16 ppm
438.16728
C24H20O2N7
-0.05 ppm
532.37273
C23H47O4N9F
-0.41 ppm
601.80147
C2H13O8N7F 32S10
0.58 ppm
731.28857
C28H50O7N8 35Cl2F 32S
0.94 ppm
787.21079
C17H38O15N10F7 32S
-1.74 ppm
946.18394
C26H42O13N7F10 32S3
0.19 ppm
m/z

Compound **6b**

^{1}H NMR

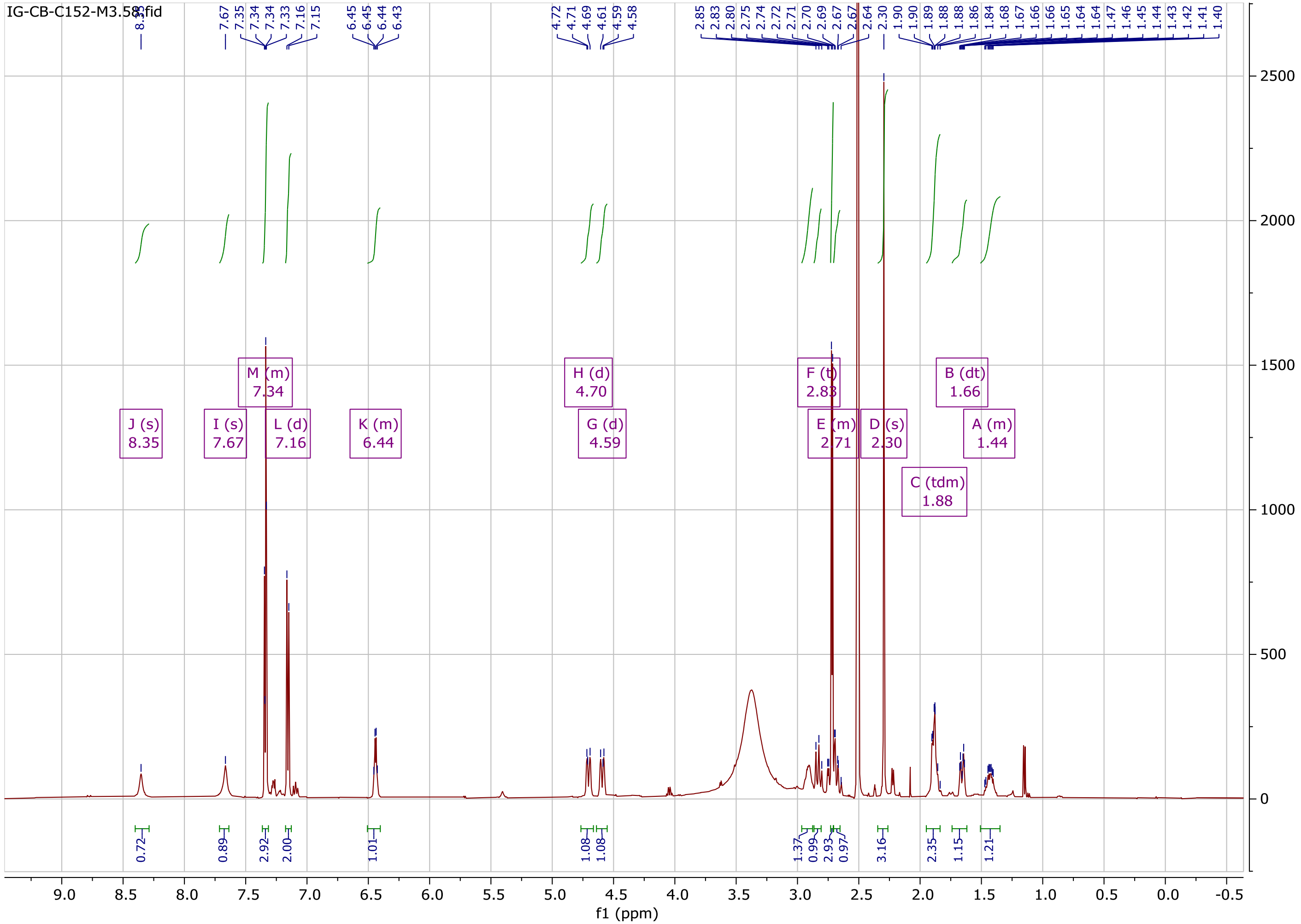

COSY

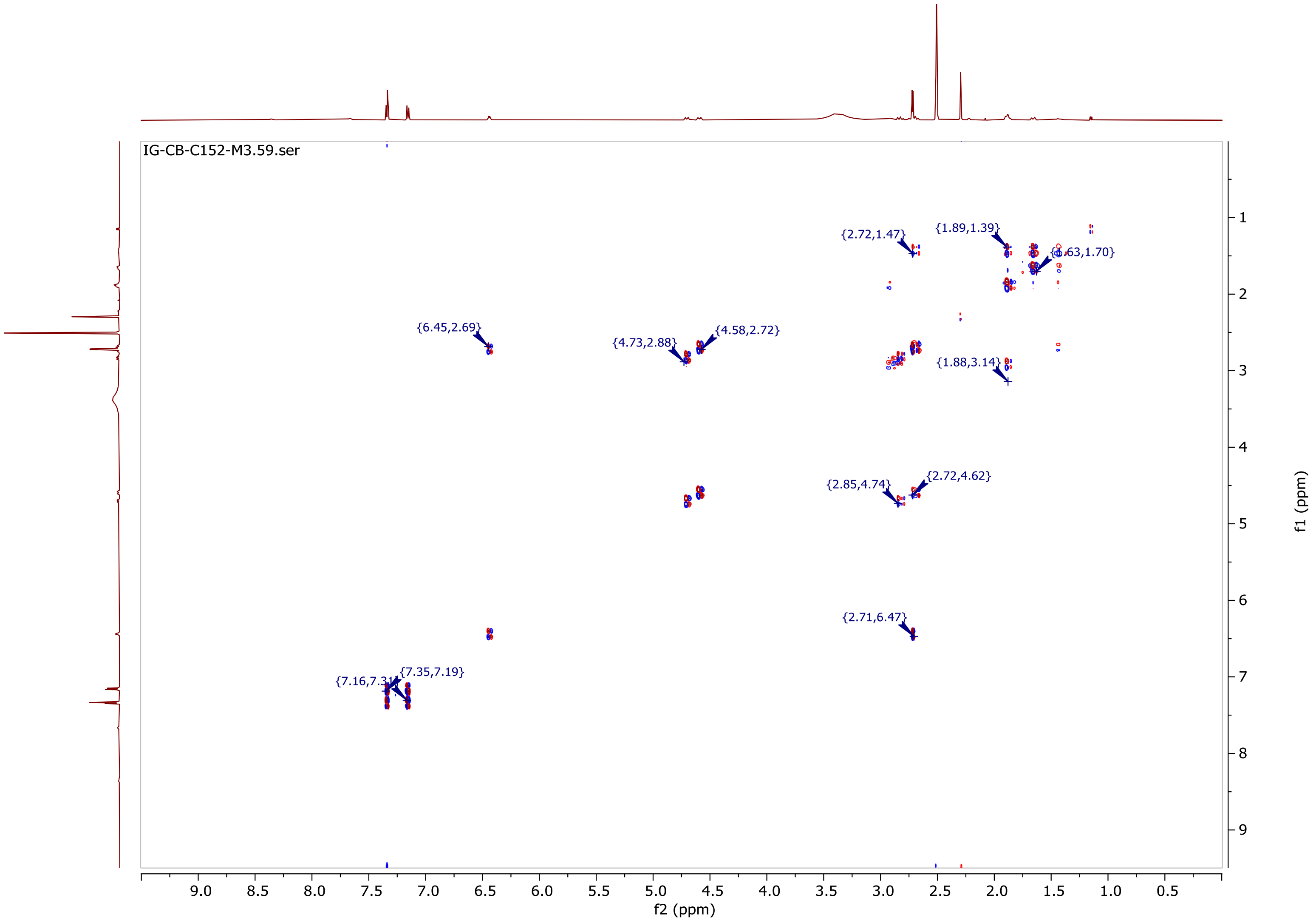
IG-CB-C152-M3.59.ser
{2.72,1.47}
{1.89,1.39}
{6.45,2.69}
{4.73,2.88}
{4.58,2.72}
{1.88,3.14}
{2.85,4.74}
{2.72,4.62}
{2.71,6.47}
{7.35,7.19}
f2 (ppm)
f1 (ppm)

HSQC

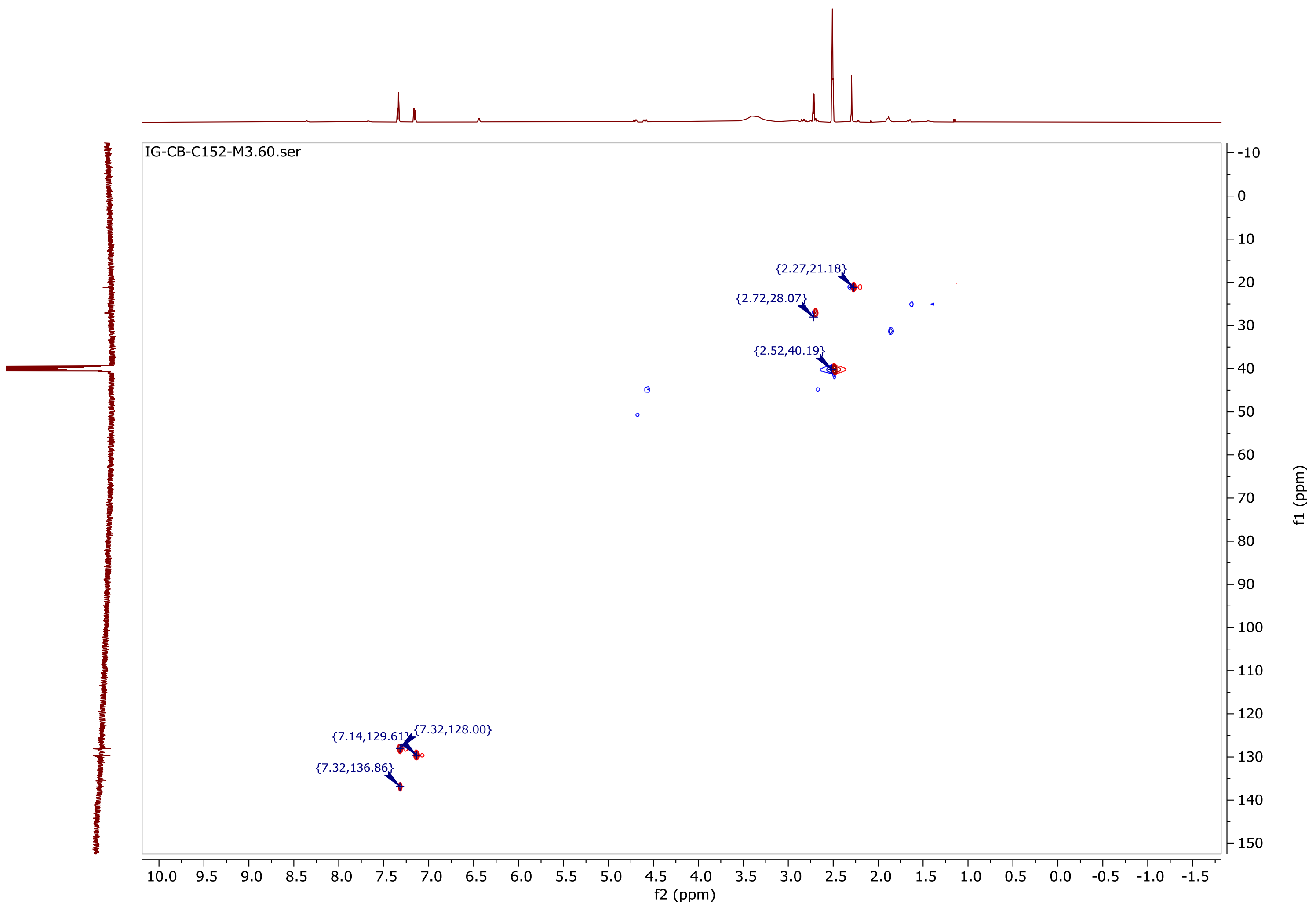
IG-CB-C152-M3.60.ser
{2.27,21.18}
{2.72,28.07}
{2.52,40.19}
{7.14,129.61}
{7.32,128.00}
{7.32,136.86}
f2 (ppm)
f1 (ppm)

HMBC

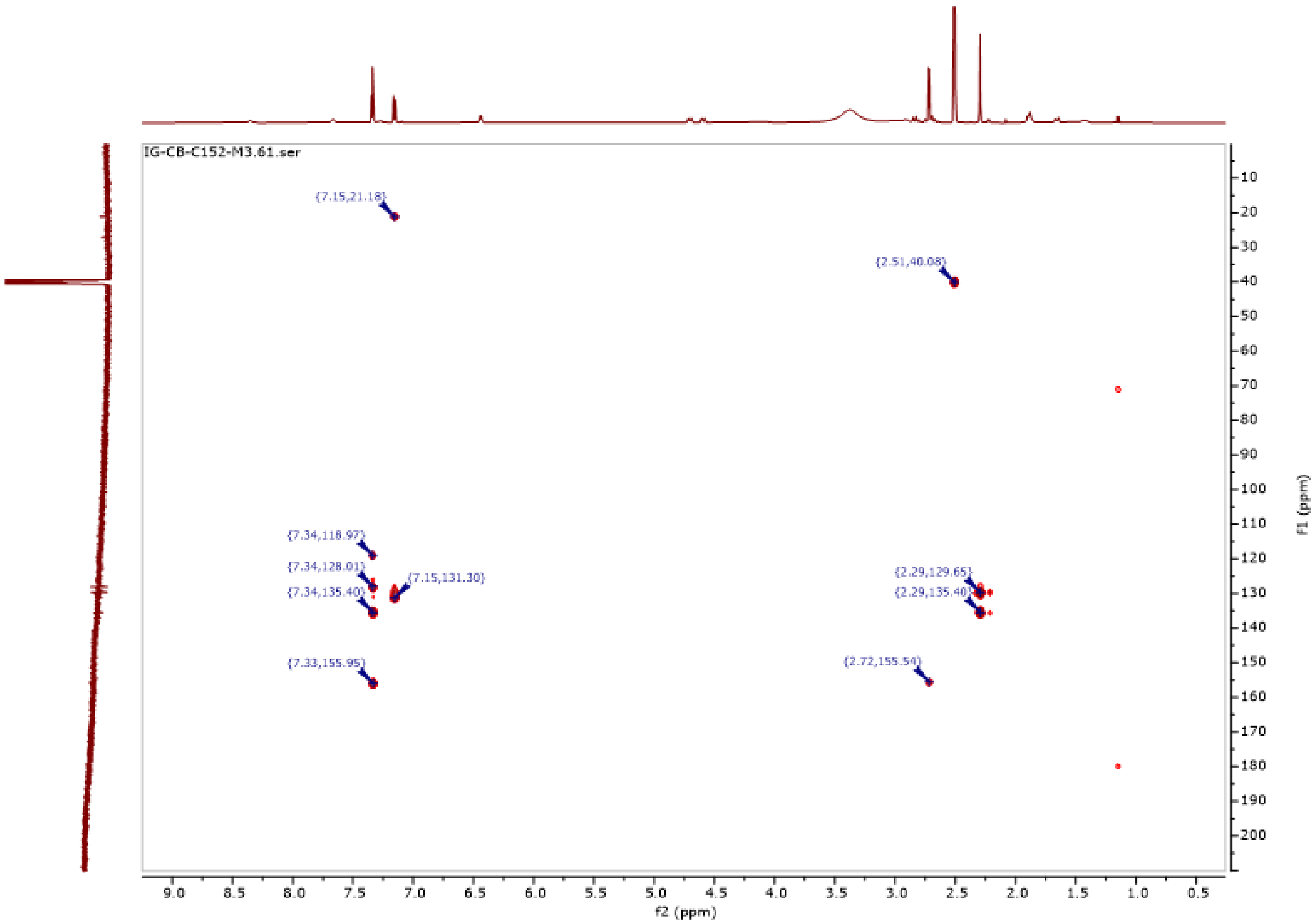
IG-CB-C152-M3.61.ser
{7.15,21.18}
{2.51,40.08}
{7.34,118.97}
{7.34,128.01}
{7.15,131.30}
{7.34,135.40}
{2.29,129.65}
{2.29,135.40}
{7.33,155.95}
{2.72,155.54}
f2 (ppm)
f1 (ppm)

DEPTqgpsp

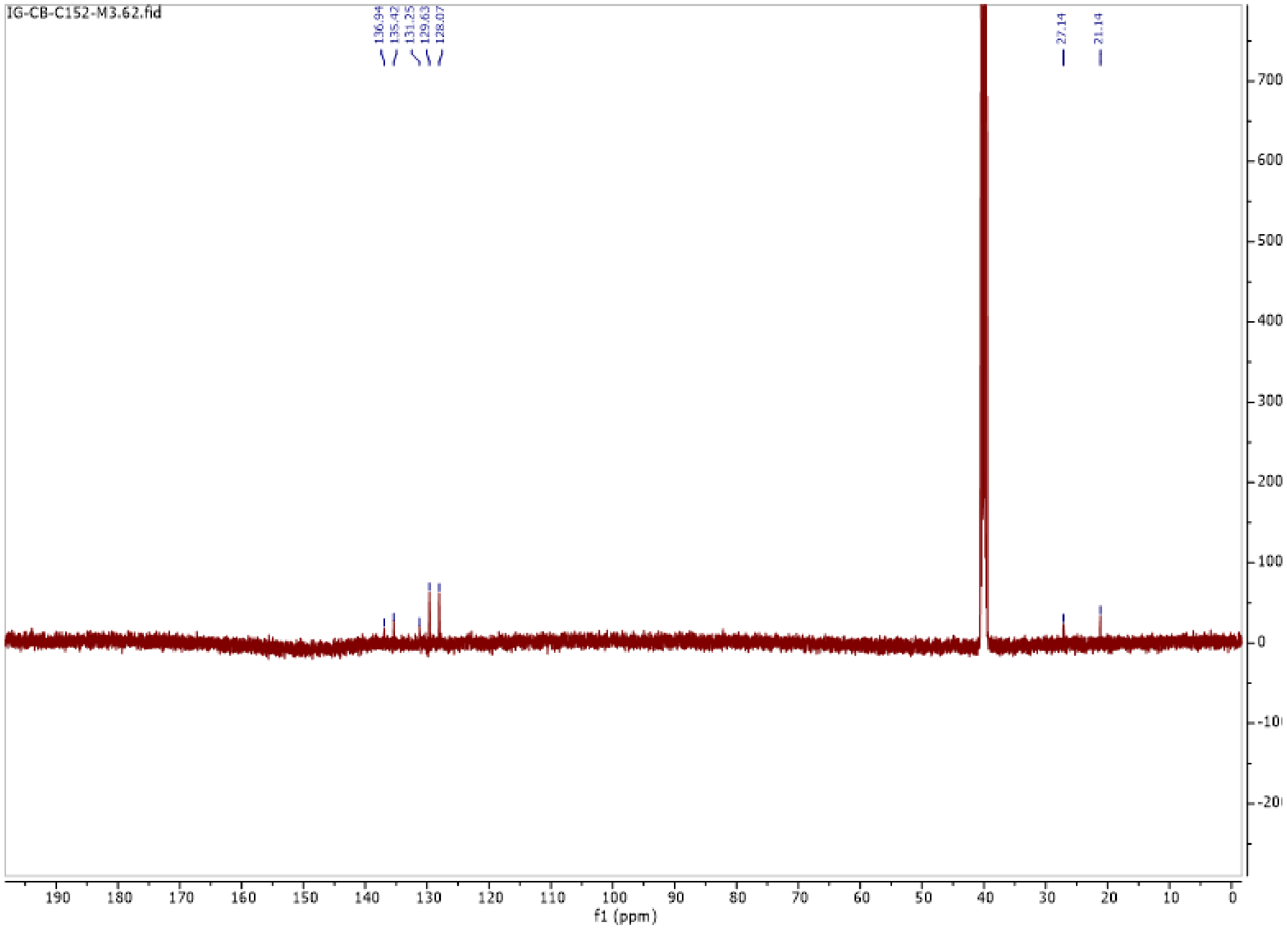

IG-CB-C152-M3.62.fid
136.94
135.42
131.25
129.63
128.07
27.14
21.14
700
600
500
400
300
200
100
0
190
180
170
160
150
140
130
120
110
100
90
80
70
60
50
40
30
20
10
0
f1 (ppm)

DEPT-90

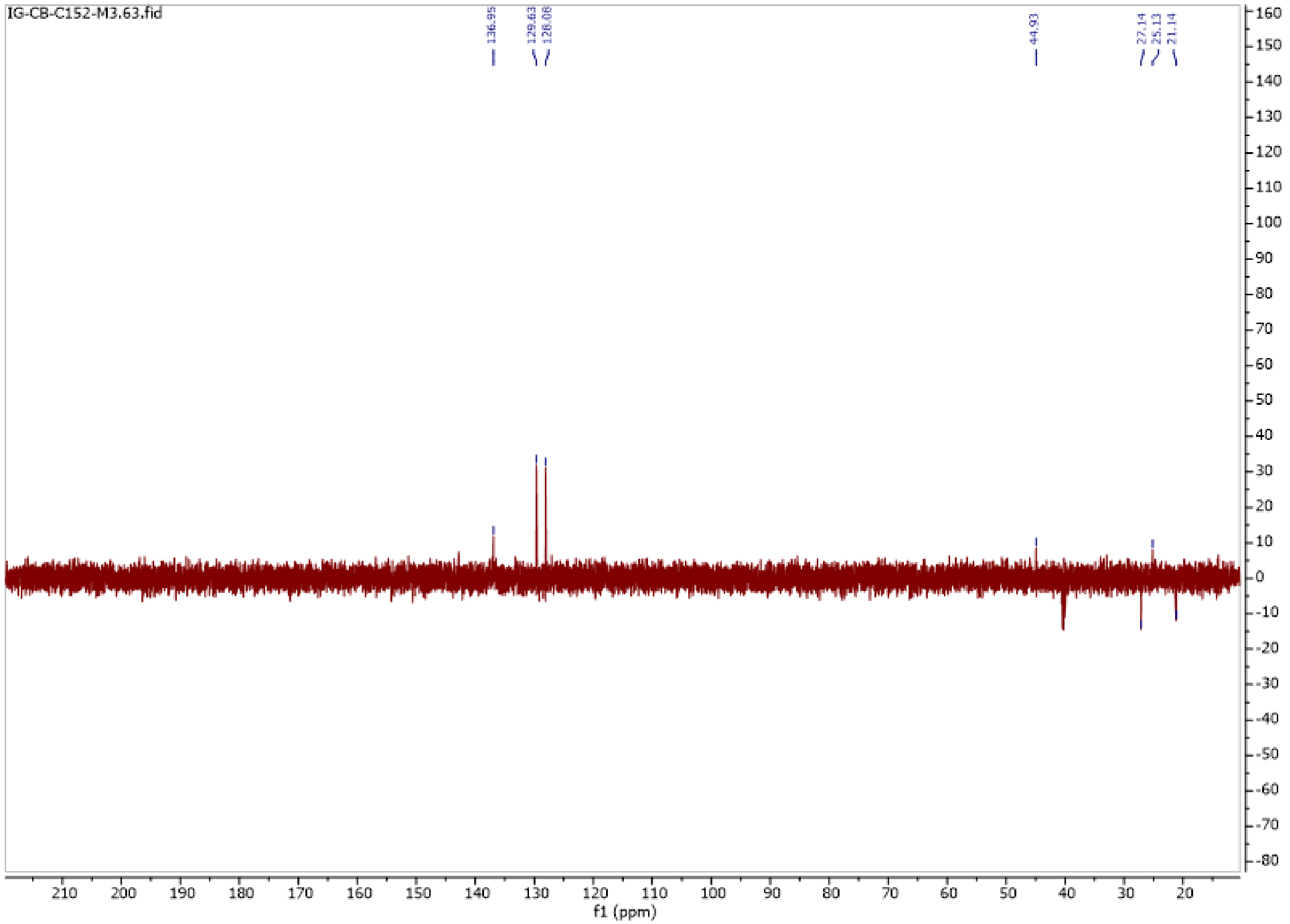
IG-CB-C152-M3.63.fid
136.95
129.63
128.08
44.93
27.14
25.13
21.14
f1 (ppm)

DEPT-135

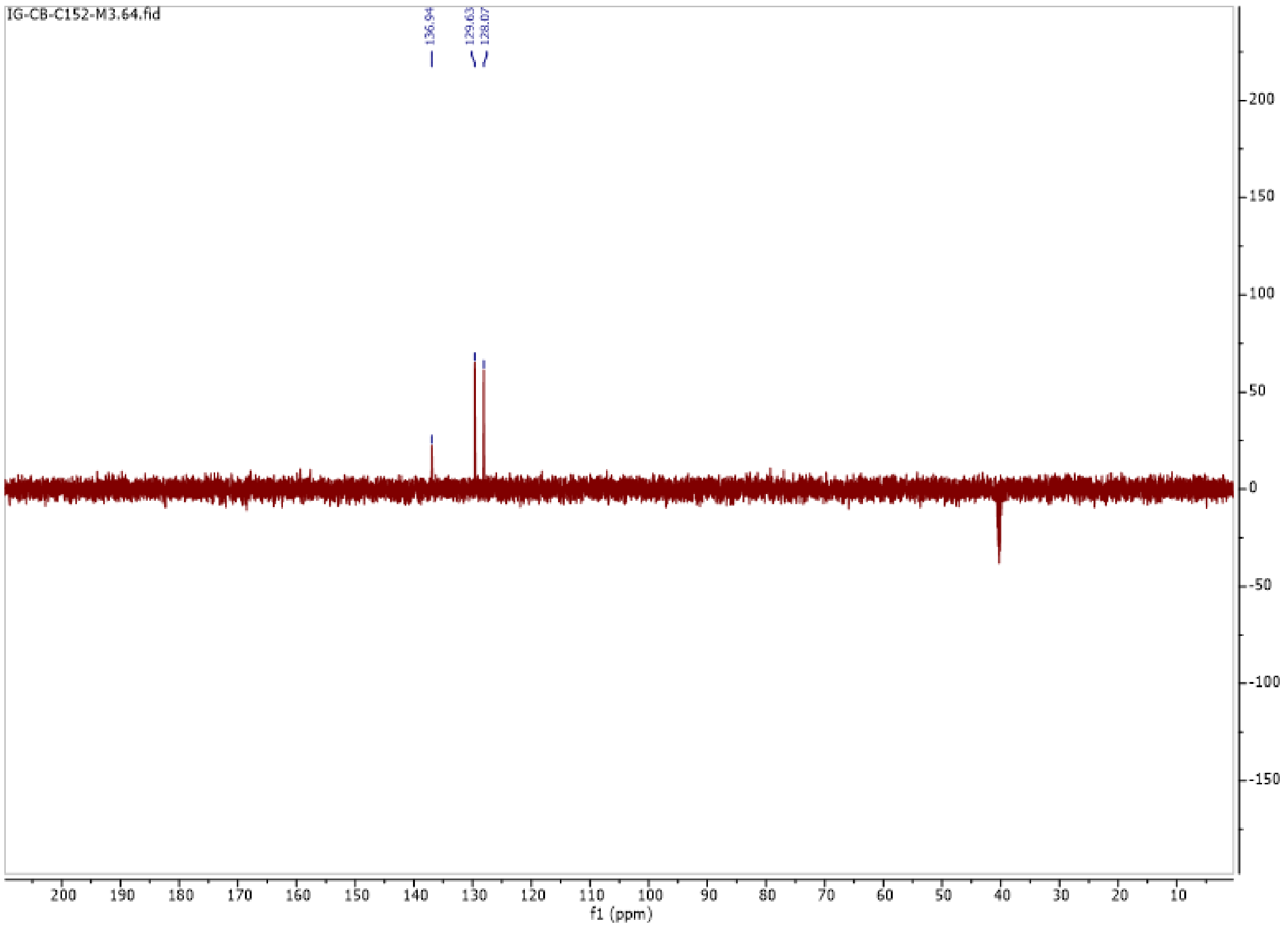
IG-CB-C152-M3.64.fid
136.94
129.63
128.07
200
150
100
50
0
-50
-100
-150
200 190 180 170 160 150 140 130 120 110 100 90 80 70 60 50 40 30 20 10
f1 (ppm)

Compound **6c**

^1H NMR

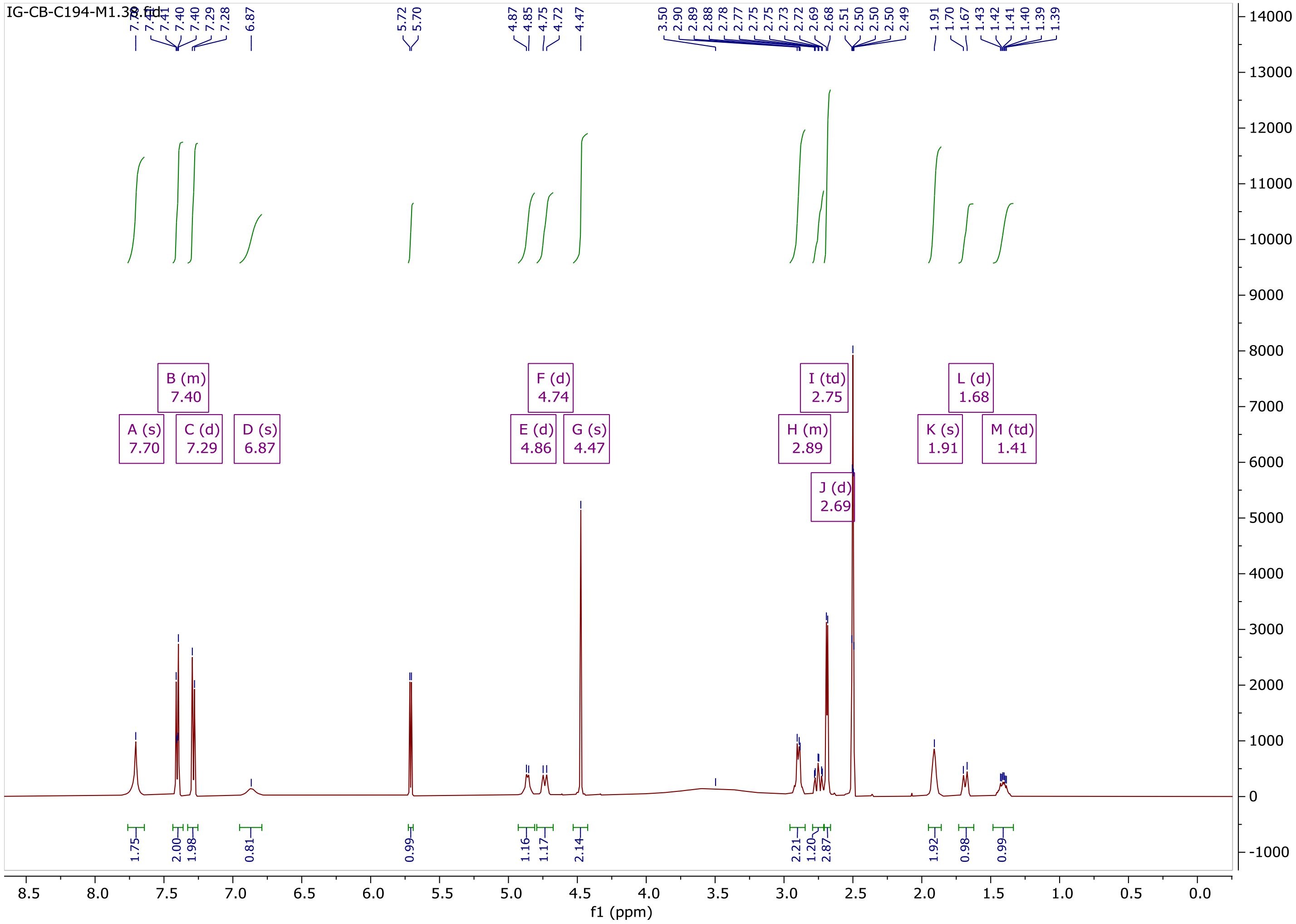

COSY

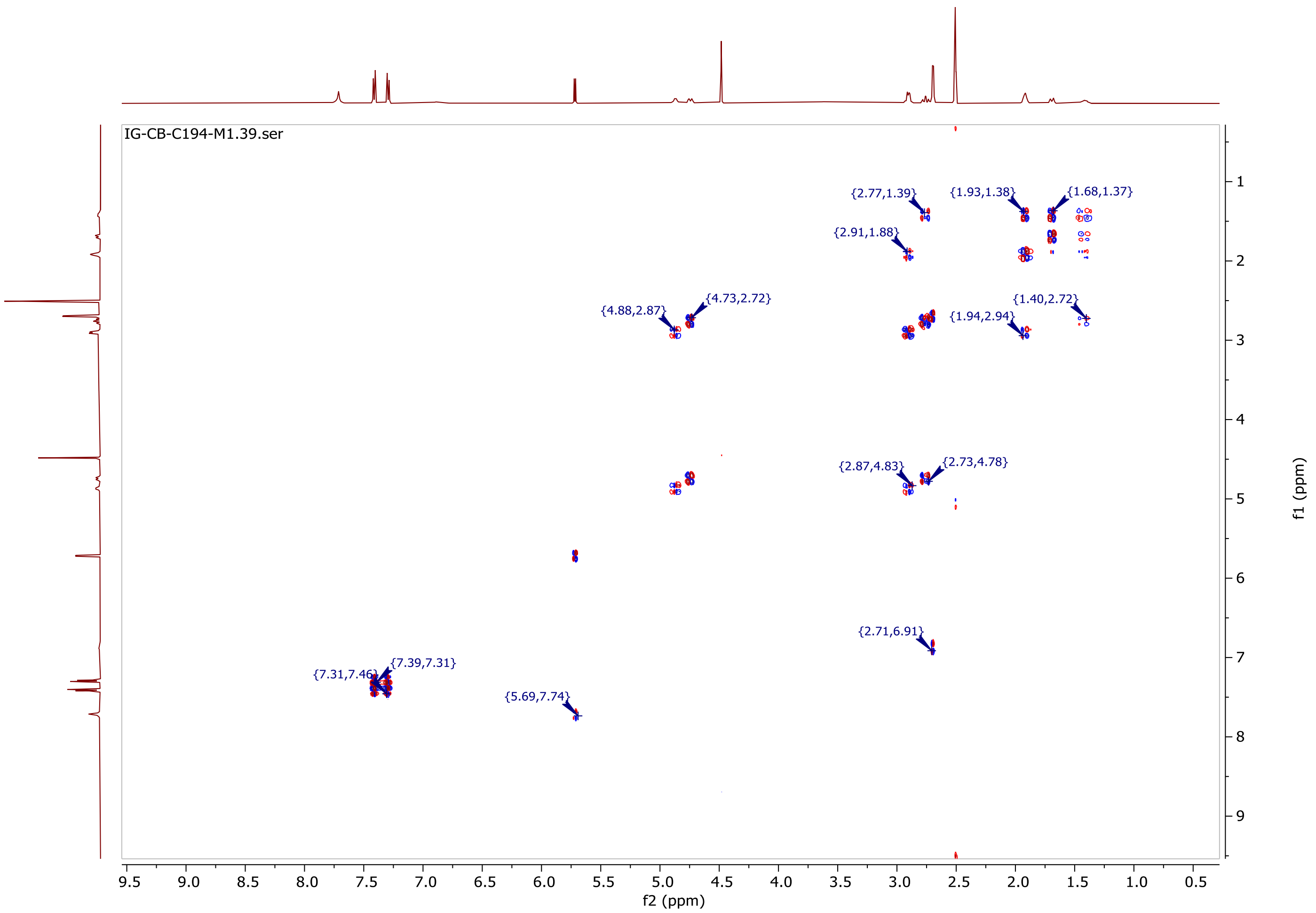
IG-CB-C194-M1.39.ser
{2.77,1.39}
{1.93,1.38}
{1.68,1.37}
{2.91,1.88}
{4.88,2.87}
{4.73,2.72}
{1.40,2.72}
{1.94,2.94}
{2.87,4.83}
{2.73,4.78}
{2.71,6.91}
{7.31,7.46}
{7.39,7.31}
{5.69,7.74}
f2 (ppm)
f1 (ppm)

HSQC

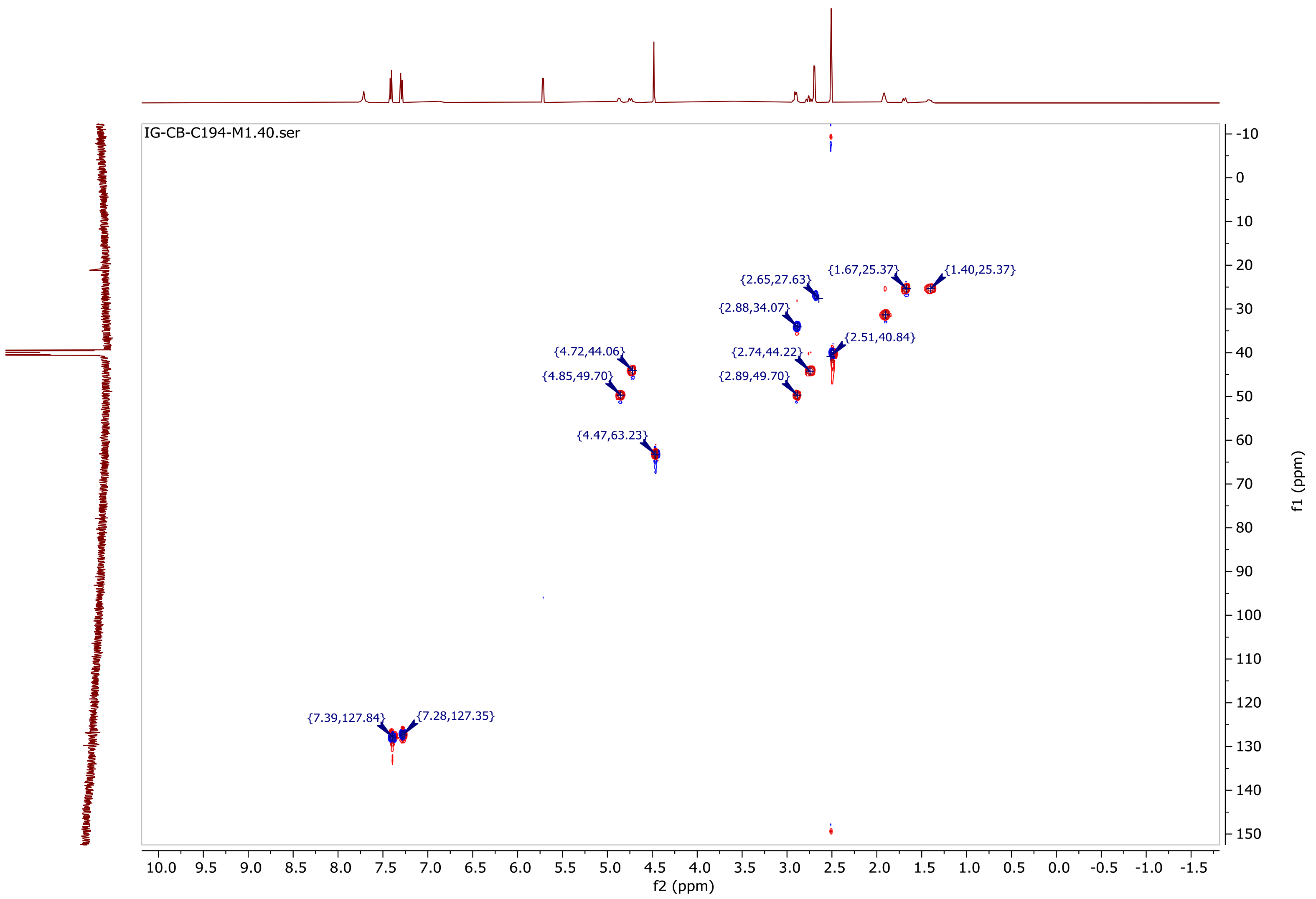
IG-CB-C194-M1.40.ser
{2.65,27.63}
{1.67,25.37}
{1.40,25.37}
{2.88,34.07}
{2.51,40.84}
{4.72,44.06}
{2.74,44.22}
{4.85,49.70}
{2.89,49.70}
{4.47,63.23}
{7.39,127.84}
{7.28,127.35}
f2 (ppm)
f1 (ppm)

HMBC

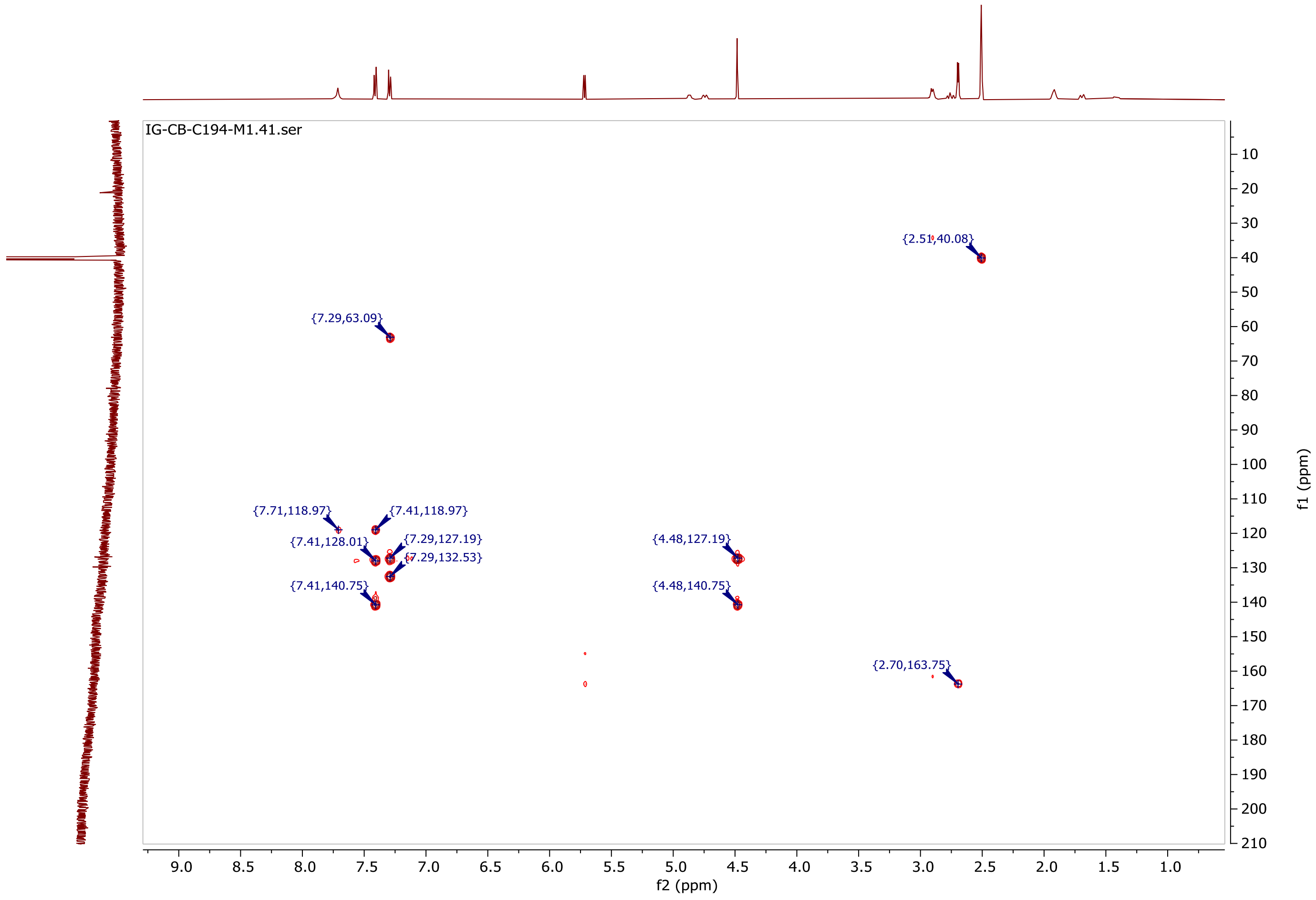
IG-CB-C194-M1.41.ser
{2.51,40.08}
{7.29,63.09}
{7.71,118.97}
{7.41,118.97}
{7.41,128.01}
{7.29,127.19}
{7.29,132.53}
{4.48,127.19}
{7.41,140.75}
{4.48,140.75}
{2.70,163.75}
f1 (ppm)
f2 (ppm)

NOESY

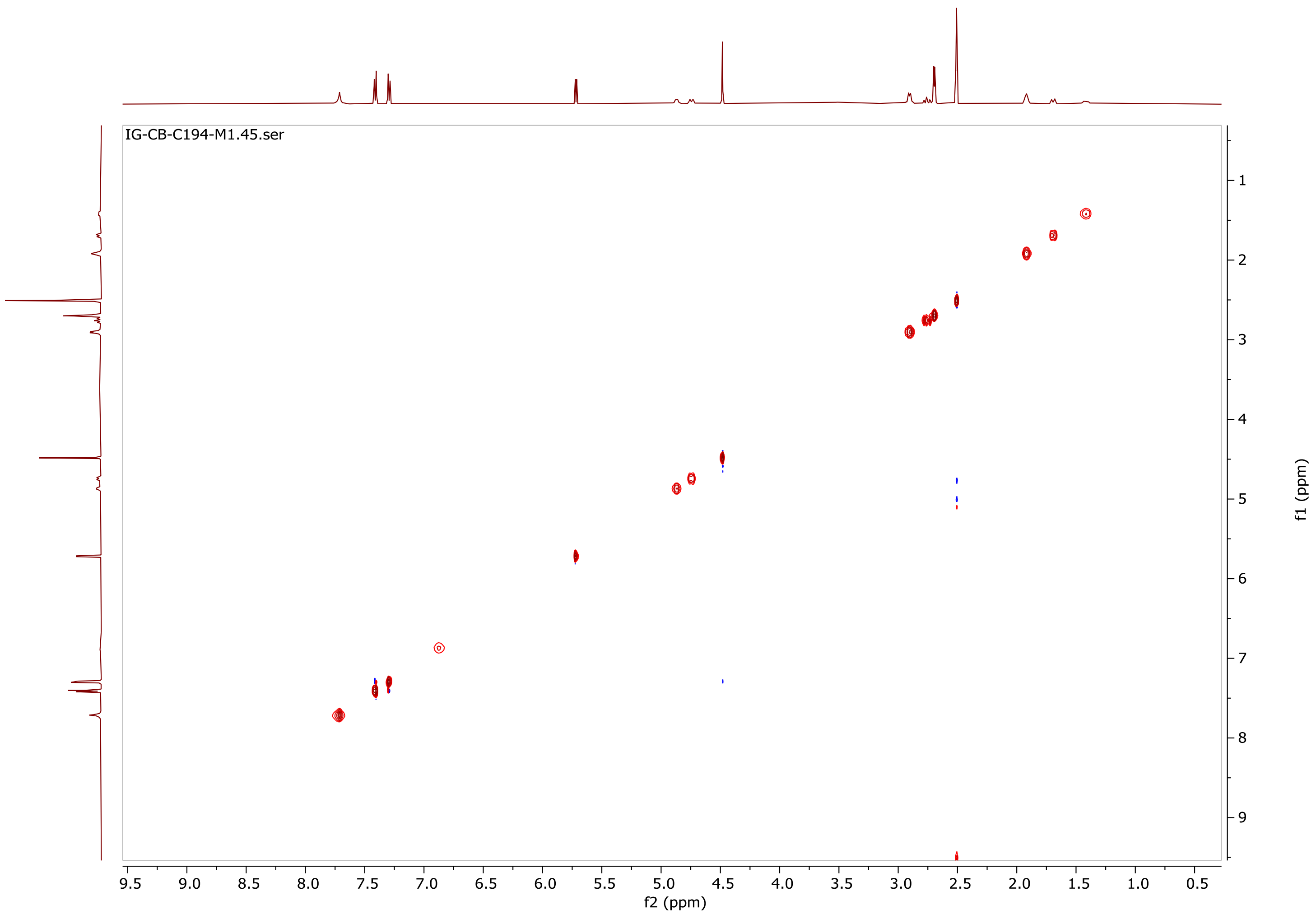
IG-CB-C194-M1.45.ser
9.5 9.0 8.5 8.0 7.5 7.0 6.5 6.0 5.5 5.0 4.5 4.0 3.5 3.0 2.5 2.0 1.5 1.0 0.5
f2 (ppm)
1 2 3 4 5 6 7 8 9
f1 (ppm)

DEPTqgpsp

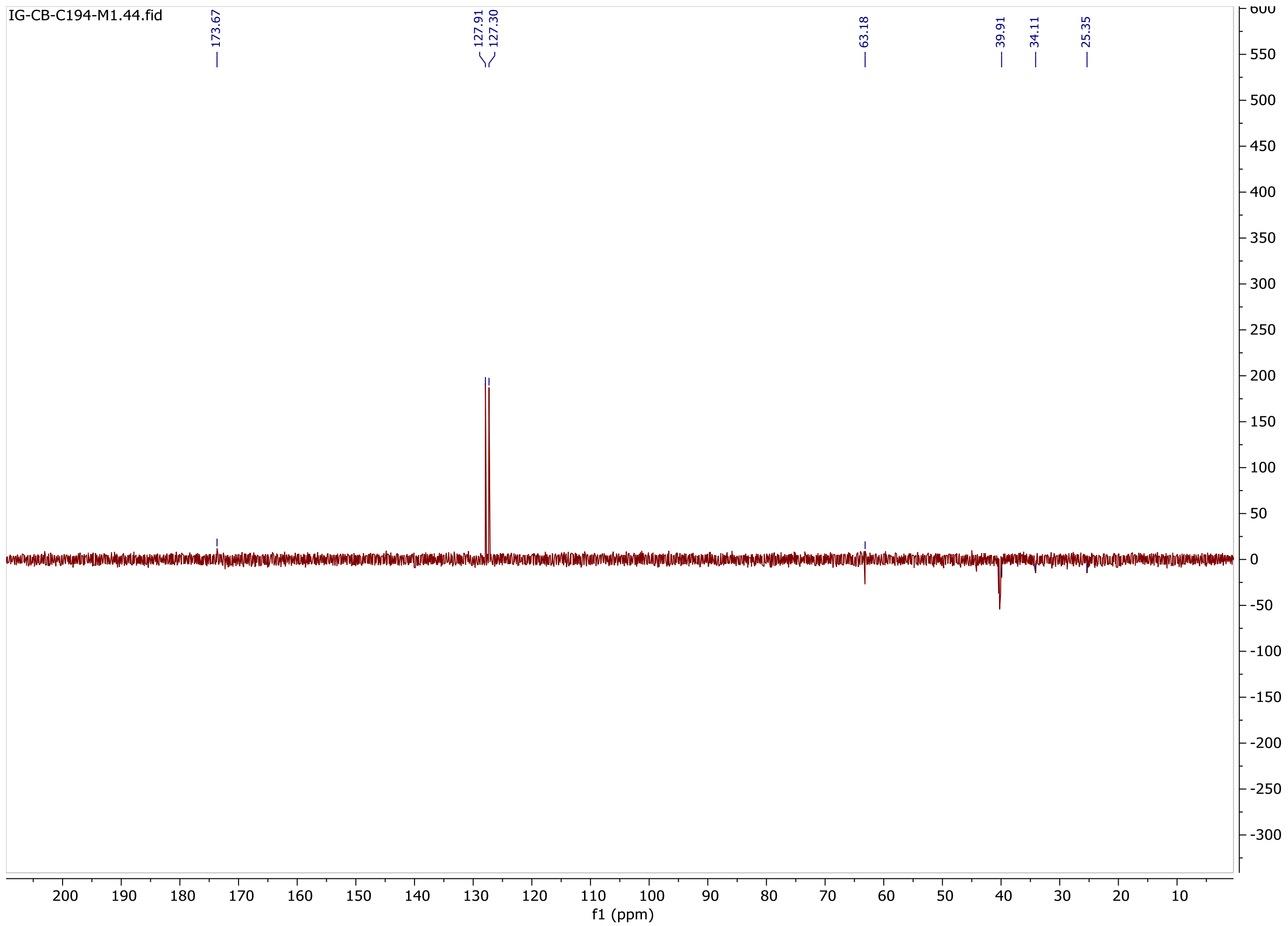
IG-CB-C194-M1.44.fid
173.67
127.91
127.30
63.18
39.91
34.11
25.35
f1 (ppm)

Zgpg30

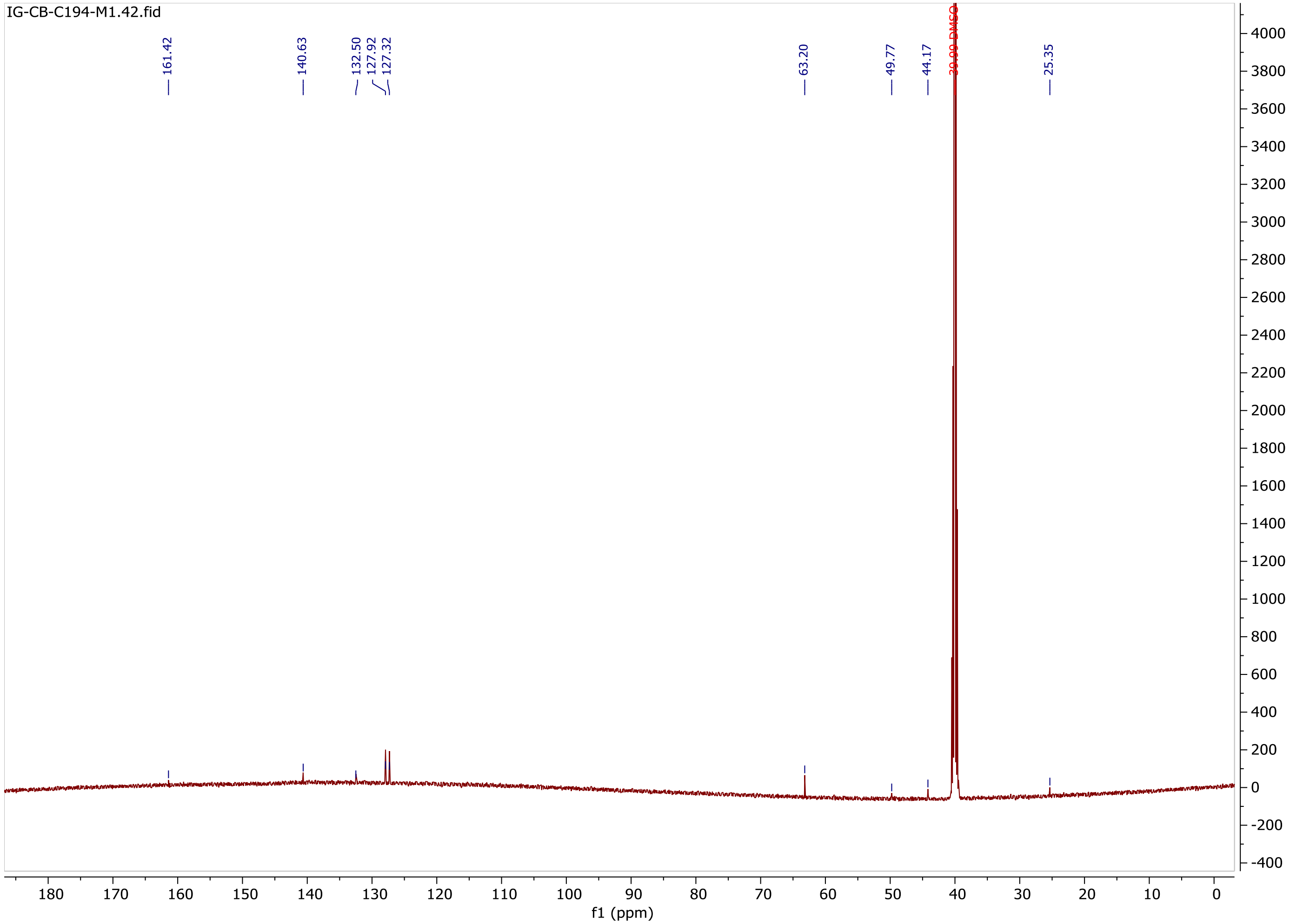
IG-CB-C194-M1.42.fid
161.42
140.63
132.50
127.92
127.32
63.20
49.77
44.17
39.99 DMSO
25.35
4000
3800
3600
3400
3200
3000
2800
2600
2400
2200
2000
1800
1600
1400
1200
1000
800
600
400
200
0
-200
-400
180
170
160
150
140
130
120
110
100
90
80
70
60
50
40
30
20
10
0
f1 (ppm)

DEPT-135

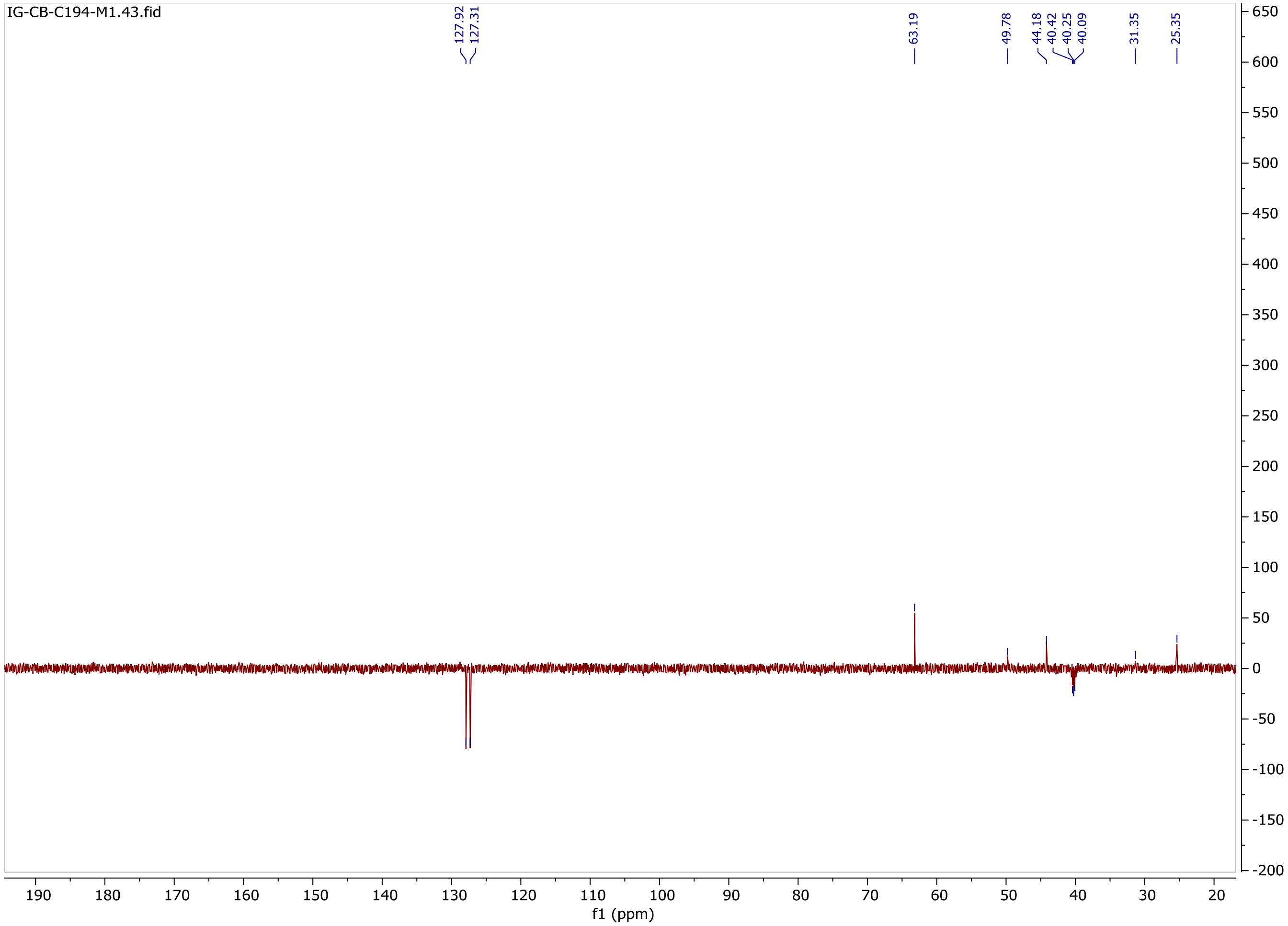

HRMS

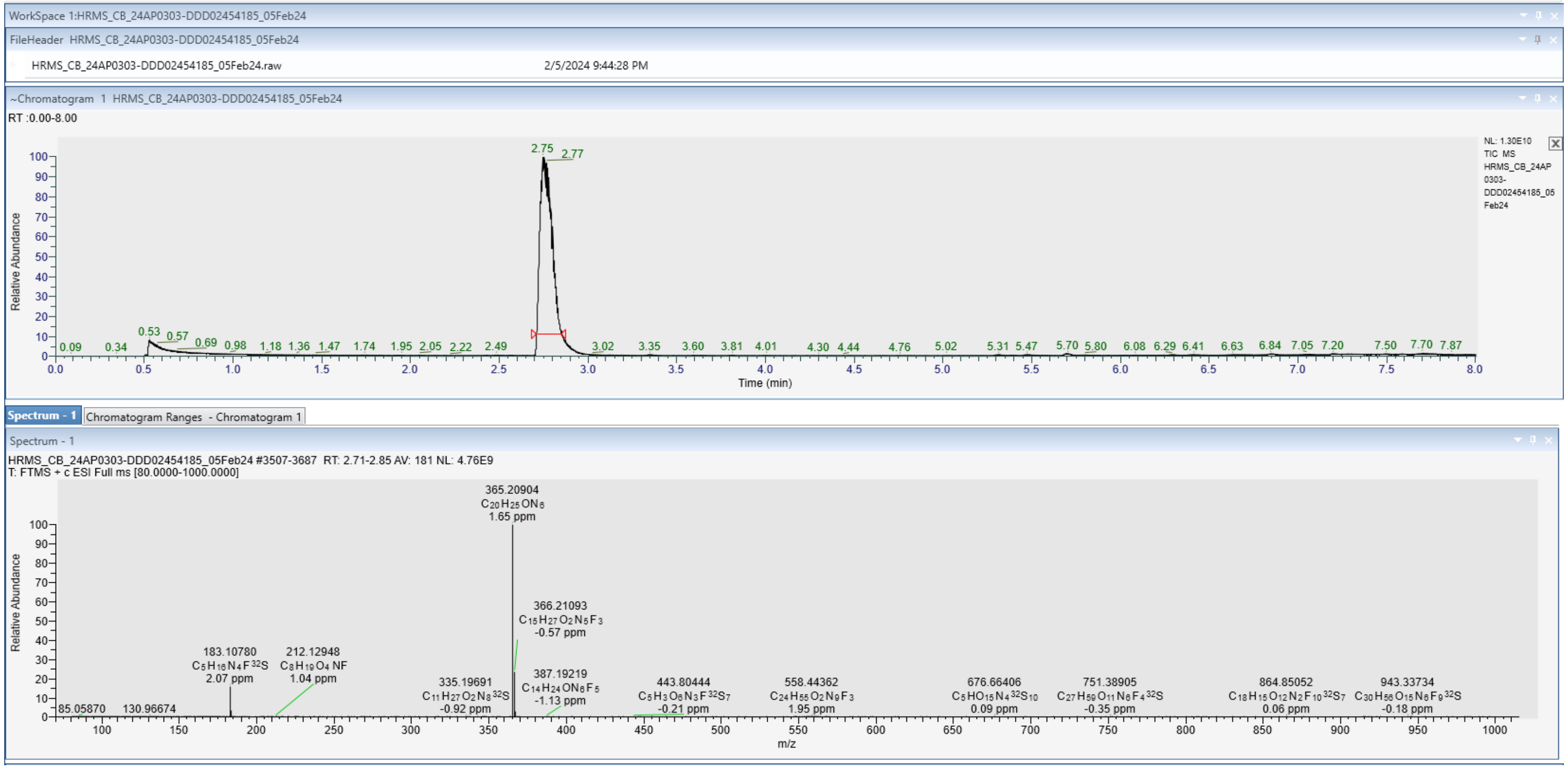
WorkSpace 1:HRMS_CB_24AP0303-DDD02454185_05Feb24
FileHeader HRMS_CB_24AP0303-DDD02454185_05Feb24
HRMS_CB_24AP0303-DDD02454185_05Feb24.raw
2/5/2024 9:44:28 PM
~Chromatogram 1 HRMS_CB_24AP0303-DDD02454185_05Feb24
RT :0.00-8.00
NL: 1.30E10 TIC MS HRMS_CB_24AP0303-DDD02454185_05Feb24
2.75
2.77
Relative Abundance
Time (min)
Spectrum - 1
Chromatogram Ranges - Chromatogram 1
HRMS_CB_24AP0303-DDD02454185_05Feb24 #3507-3687 RT: 2.71-2.85 AV: 181 NL: 4.76E9
T: FTMS + c ESI Full ms [80.0000-1000.0000]
365.20904
C20 H25 O N6
1.65 ppm
366.21093
C15 H27 O2 N6 F3
-0.57 ppm
183.10780
C5 H16 N4 F 32S
2.07 ppm
212.12948
C8 H19 O4 NF
1.04 ppm
335.19691
C11 H27 O2 N8 32S
-0.92 ppm
387.19219
C14 H24 O N8 F5
-1.13 ppm
85.05870
130.96674
443.80444
C5 H3 O6 N3 F 32S7
-0.21 ppm
558.44362
C24 H55 O2 N9 F3
1.95 ppm
676.66406
C5 H O15 N4 32S10
0.09 ppm
751.38905
C27 H50 O11 N6 F4 32S
-0.35 ppm
864.85052
C18 H15 O12 N2 F10 32S7
0.06 ppm
943.33734
C30 H56 O15 N6 F9 32S
-0.18 ppm
m/z

Compound **6d**

^{1}H NMR

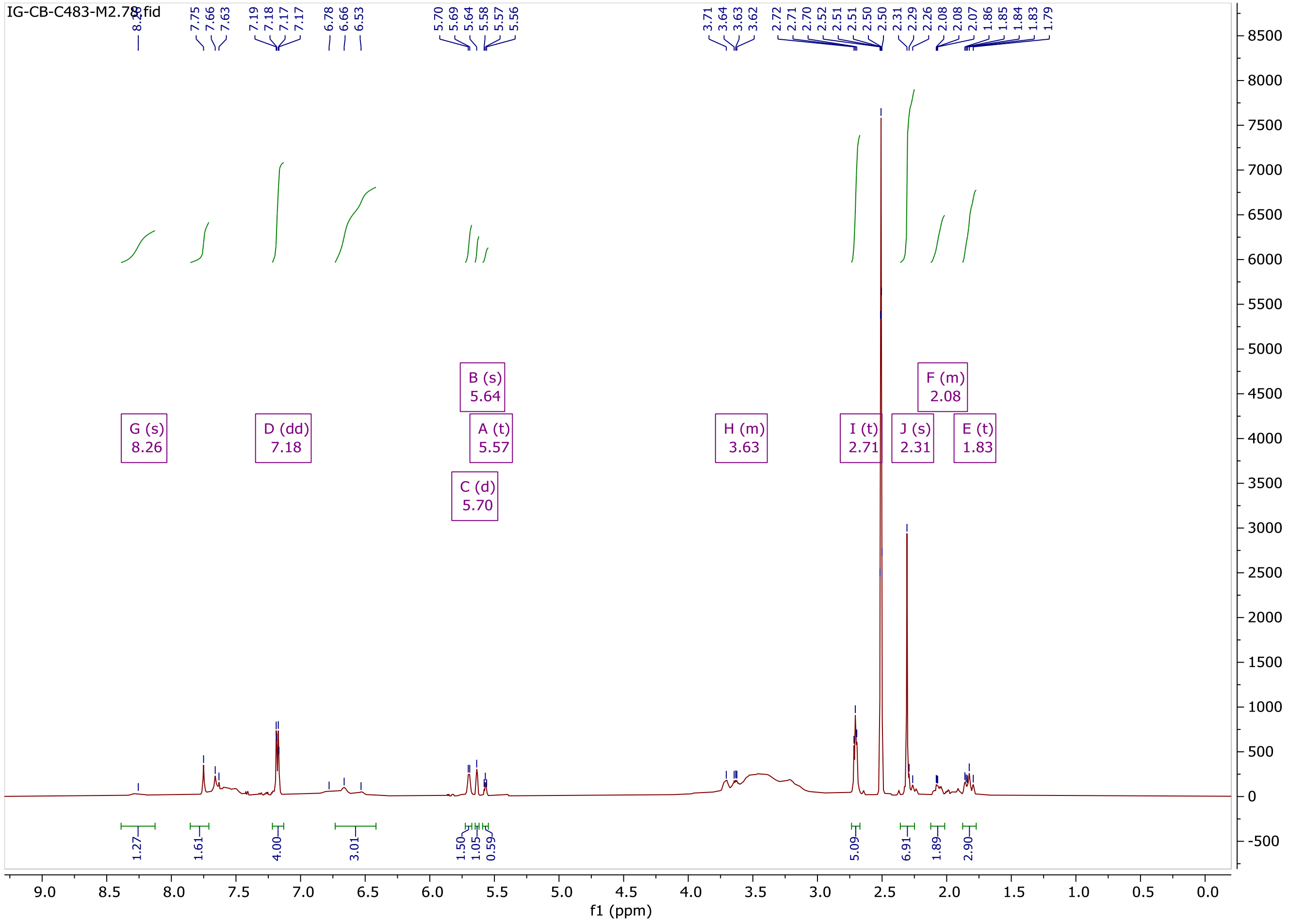

COSY

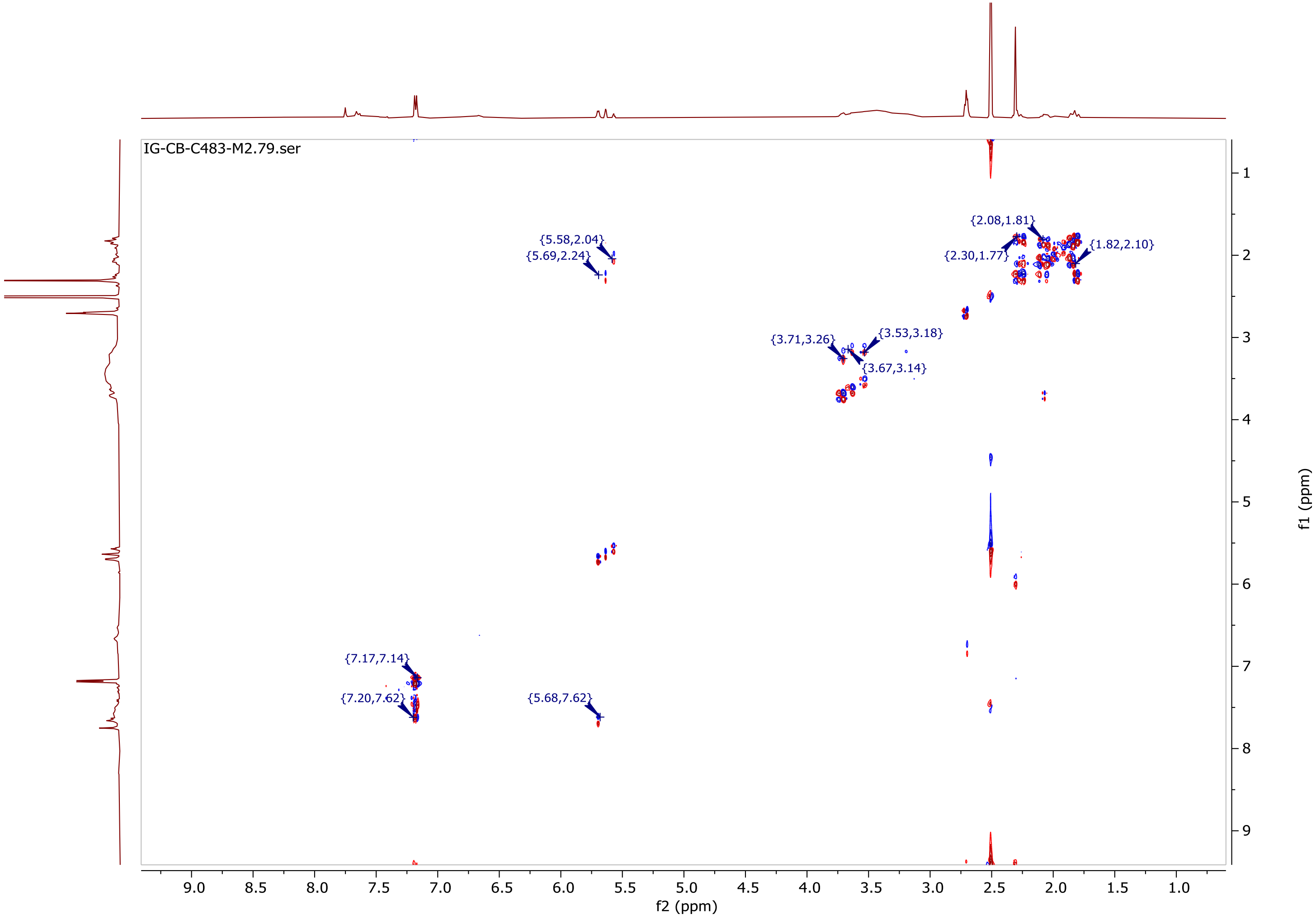
IG-CB-C483-M2.79.ser
{5.58,2.04}
{5.69,2.24}
{2.08,1.81}
{2.30,1.77}
{1.82,2.10}
{3.71,3.26}
{3.53,3.18}
{3.67,3.14}
{7.17,7.14}
{7.20,7.62}
{5.68,7.62}
f2 (ppm)
f1 (ppm)

HSQC

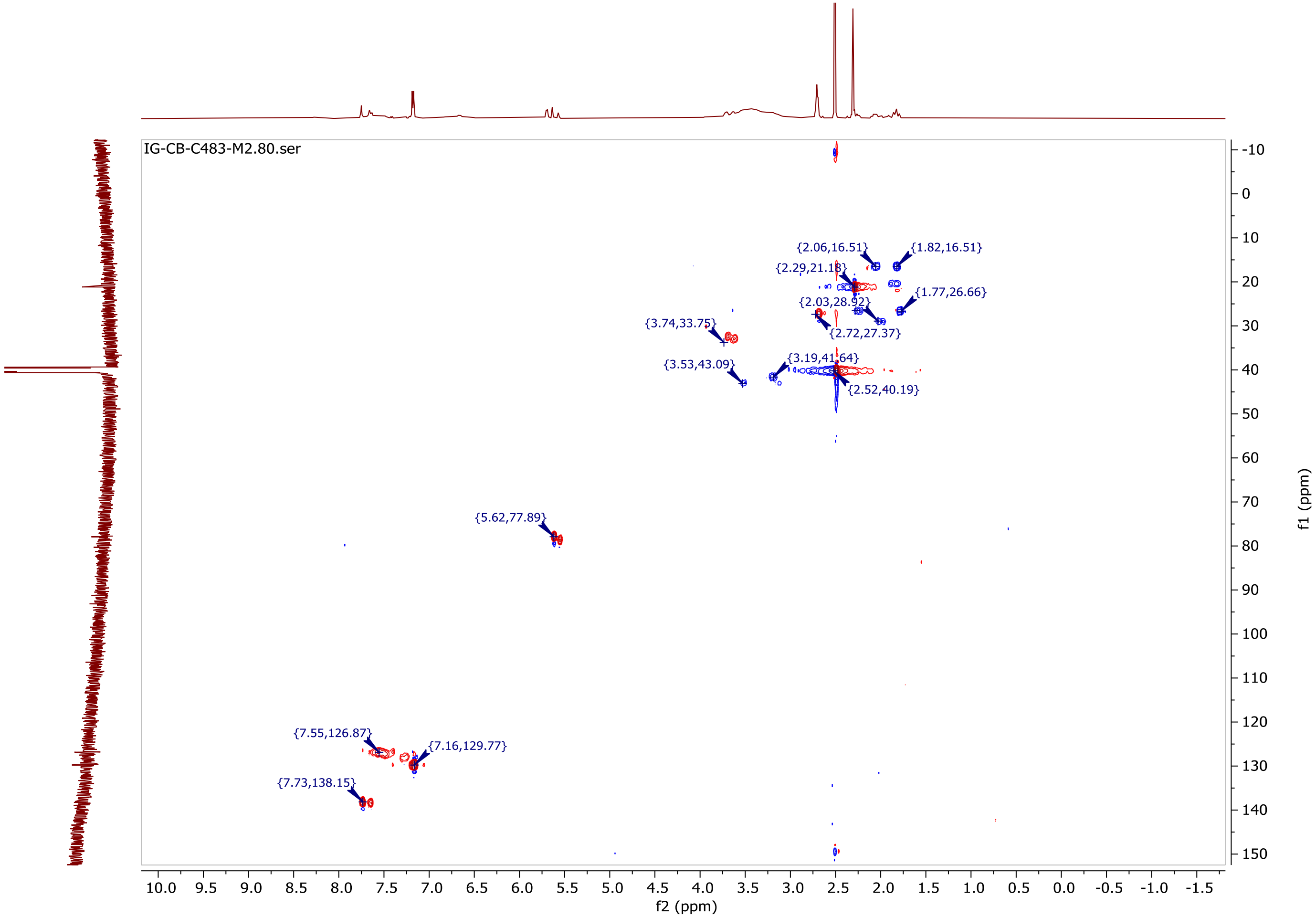
IG-CB-C483-M2.80.ser
{2.06,16.51}
{1.82,16.51}
{2.29,21.13}
{1.77,26.66}
{2.03,28.92}
{3.74,33.75}
{2.72,27.37}
{3.19,41.64}
{3.53,43.09}
{2.52,40.19}
{5.62,77.89}
{7.55,126.87}
{7.16,129.77}
{7.73,138.15}
f2 (ppm)
f1 (ppm)

HMBC

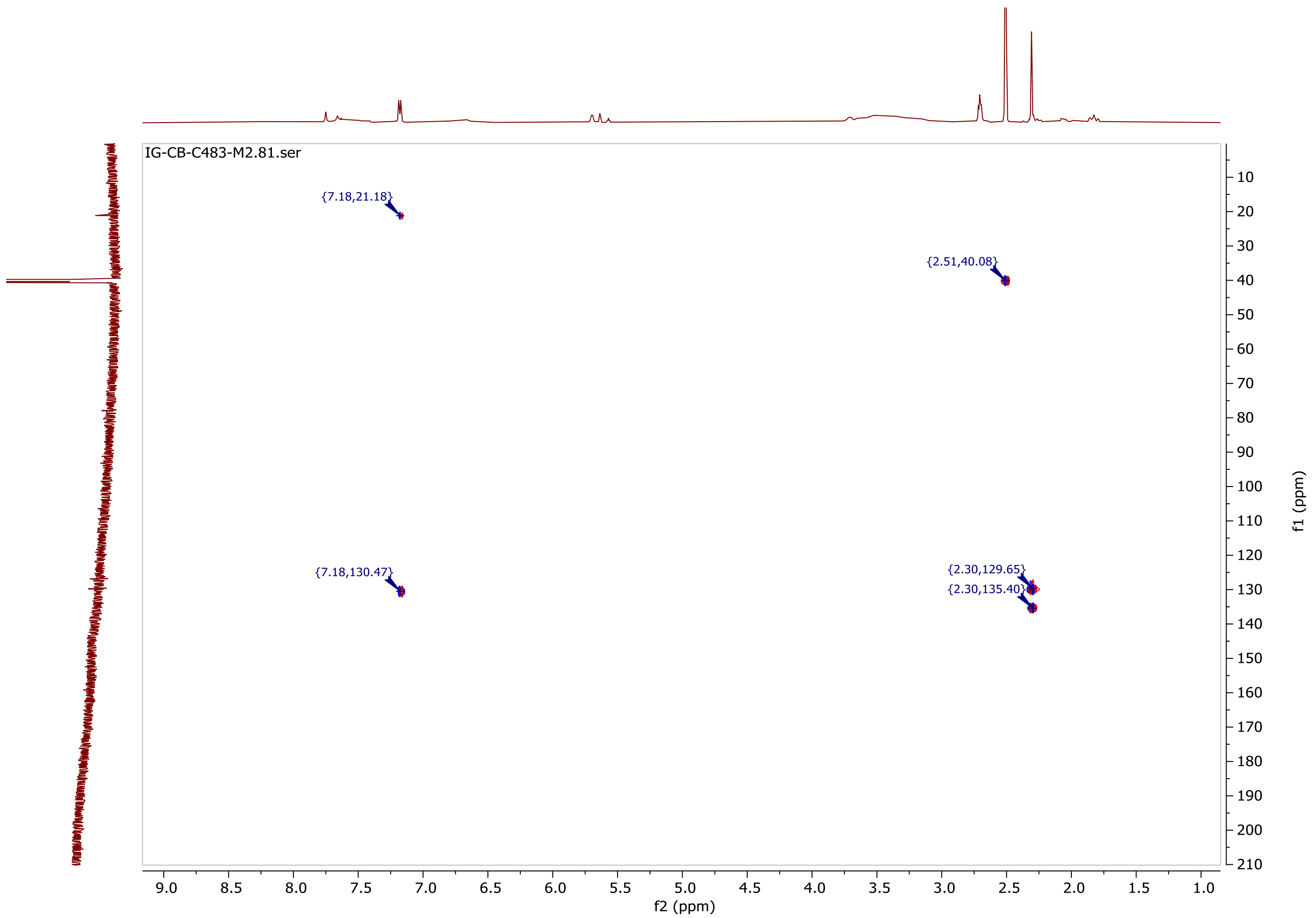
IG-CB-C483-M2.81.ser
{7.18,21.18}
{2.51,40.08}
{7.18,130.47}
{2.30,129.65}
{2.30,135.40}
f2 (ppm)
f1 (ppm)

HRMS

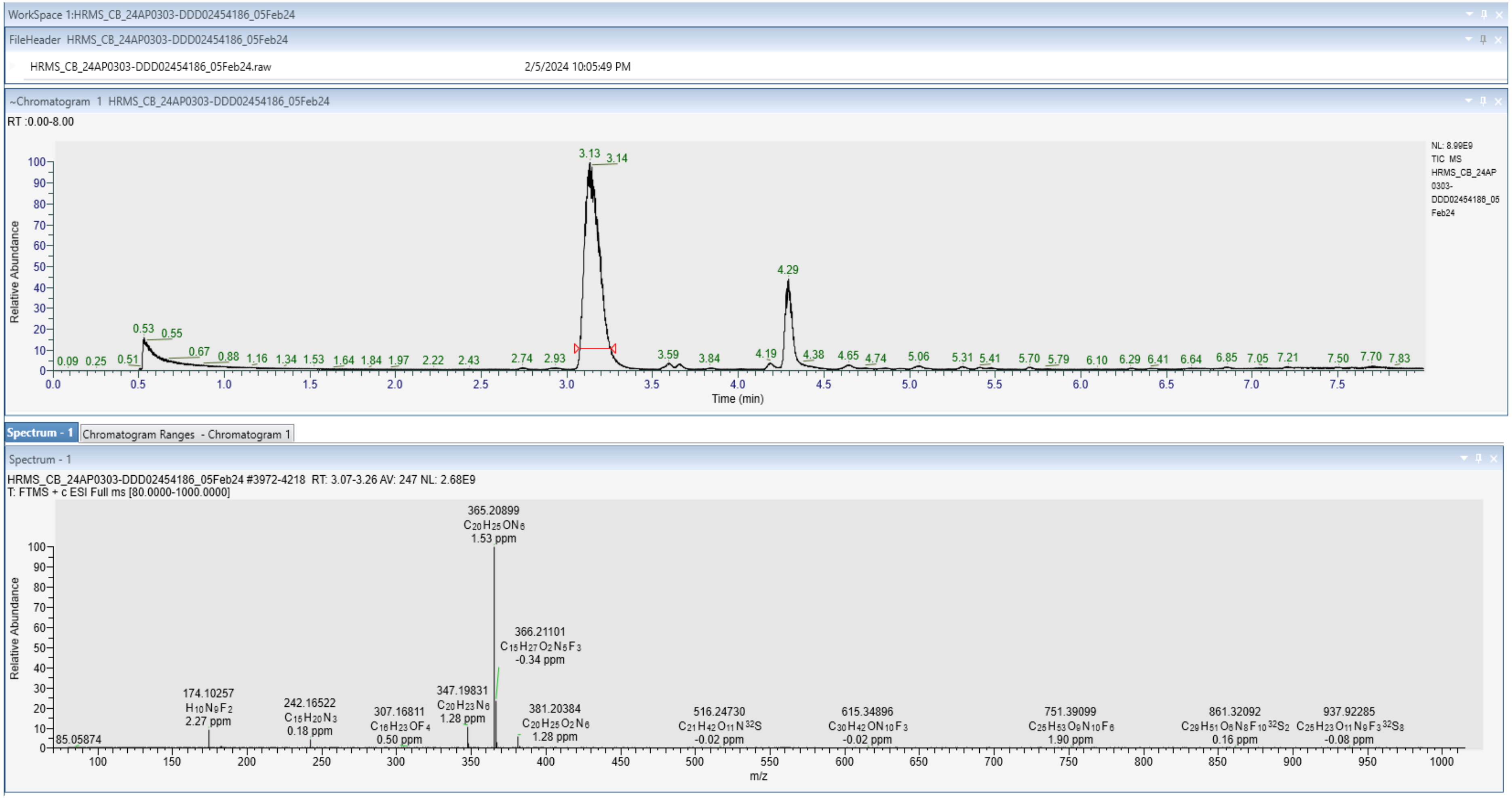

WorkSpace 1:HRMS_CB_24AP0303-DDD02454186_05Feb24
FileHeader HRMS_CB_24AP0303-DDD02454186_05Feb24
HRMS_CB_24AP0303-DDD02454186_05Feb24.raw
2/5/2024 10:05:49 PM
~Chromatogram 1 HRMS_CB_24AP0303-DDD02454186_05Feb24
RT :0.00-8.00
NL: 8.99E9 TIC MS HRMS_CB_24AP0303-DDD02454186_05Feb24
Relative Abundance
Time (min)
Spectrum - 1
Chromatogram Ranges - Chromatogram 1
HRMS_CB_24AP0303-DDD02454186_05Feb24 #3972-4218 RT: 3.07-3.26 AV: 247 NL: 2.68E9
T: FTMS + c ESI Full ms [80.0000-1000.0000]
365.20899
C20H25ON6
1.53 ppm
366.21101
C15H27O2N6F3
-0.34 ppm
m/z

Compound **6e**

^{1}H NMR

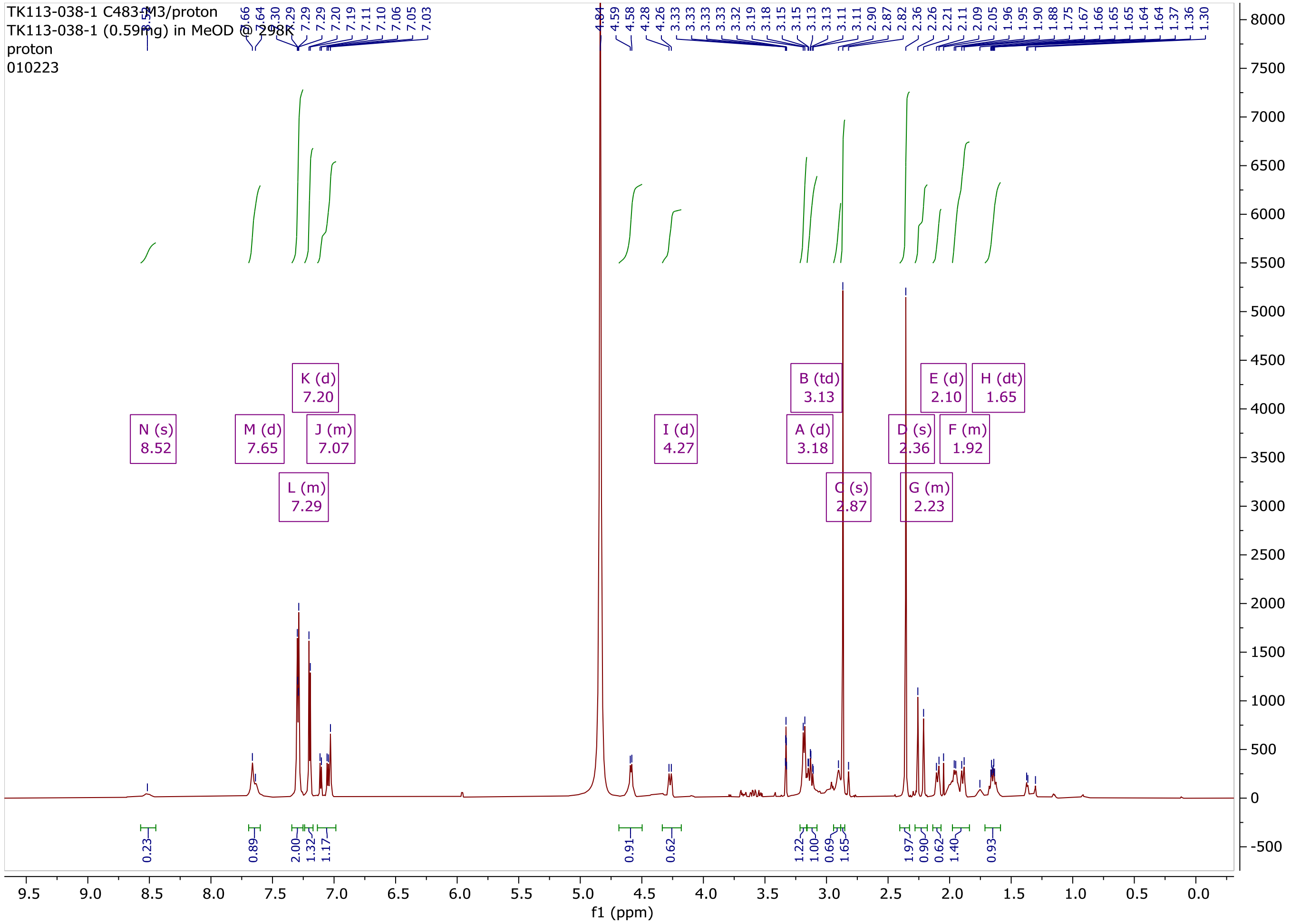

COSY

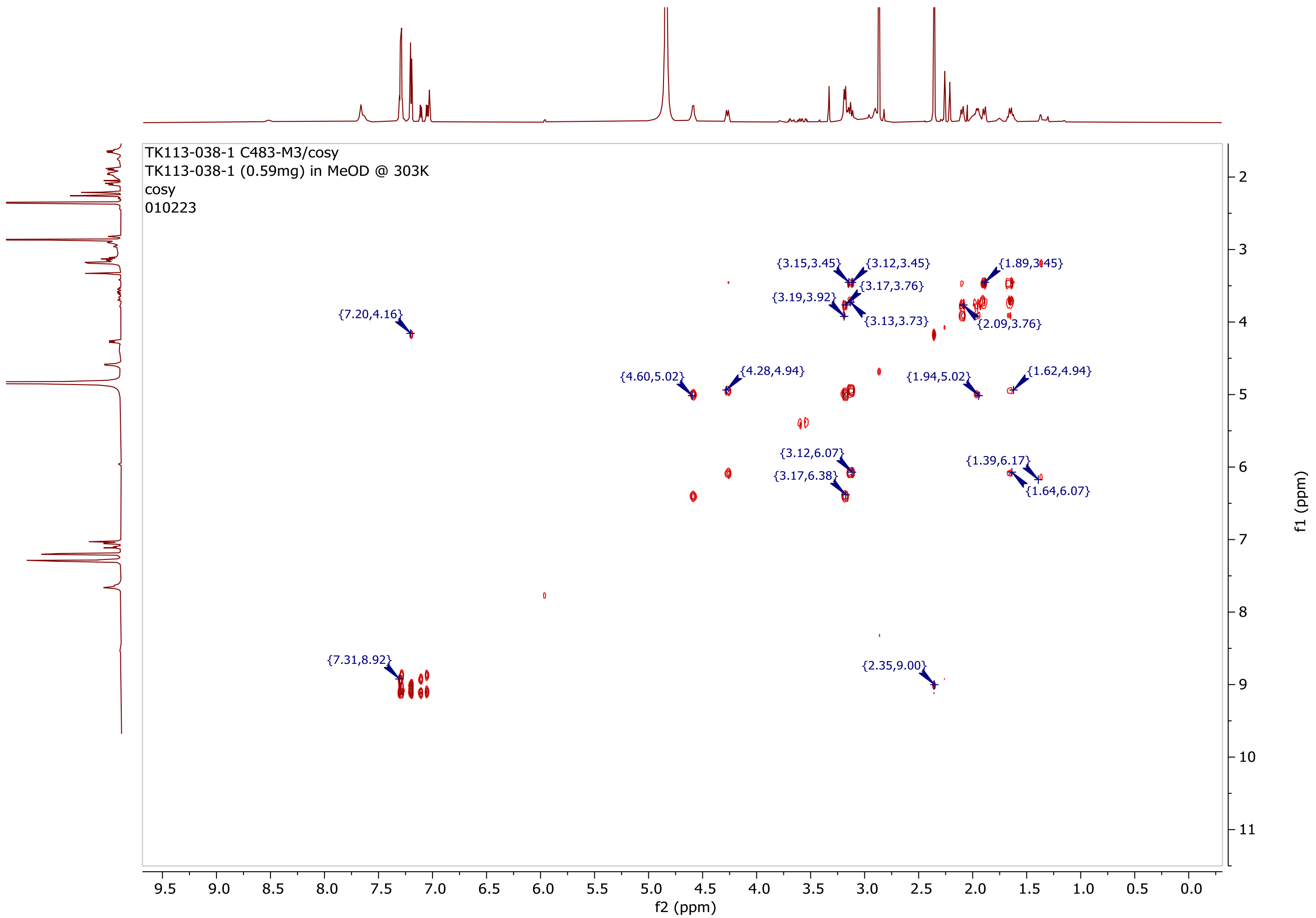

TK113-038-1 C483-M3/cosy
TK113-038-1 (0.59mg) in MeOD @ 303K
cosy
010223
{3.15,3.45}
{3.12,3.45}
{1.89,3.45}
{3.17,3.76}
{3.19,3.92}
{3.13,3.73}
{2.09,3.76}
{7.20,4.16}
{4.60,5.02}
{4.28,4.94}
{1.94,5.02}
{1.62,4.94}
{3.12,6.07}
{3.17,6.38}
{1.39,6.17}
{1.64,6.07}
{7.31,8.92}
{2.35,9.00}
f2 (ppm)
f1 (ppm)

HSQC

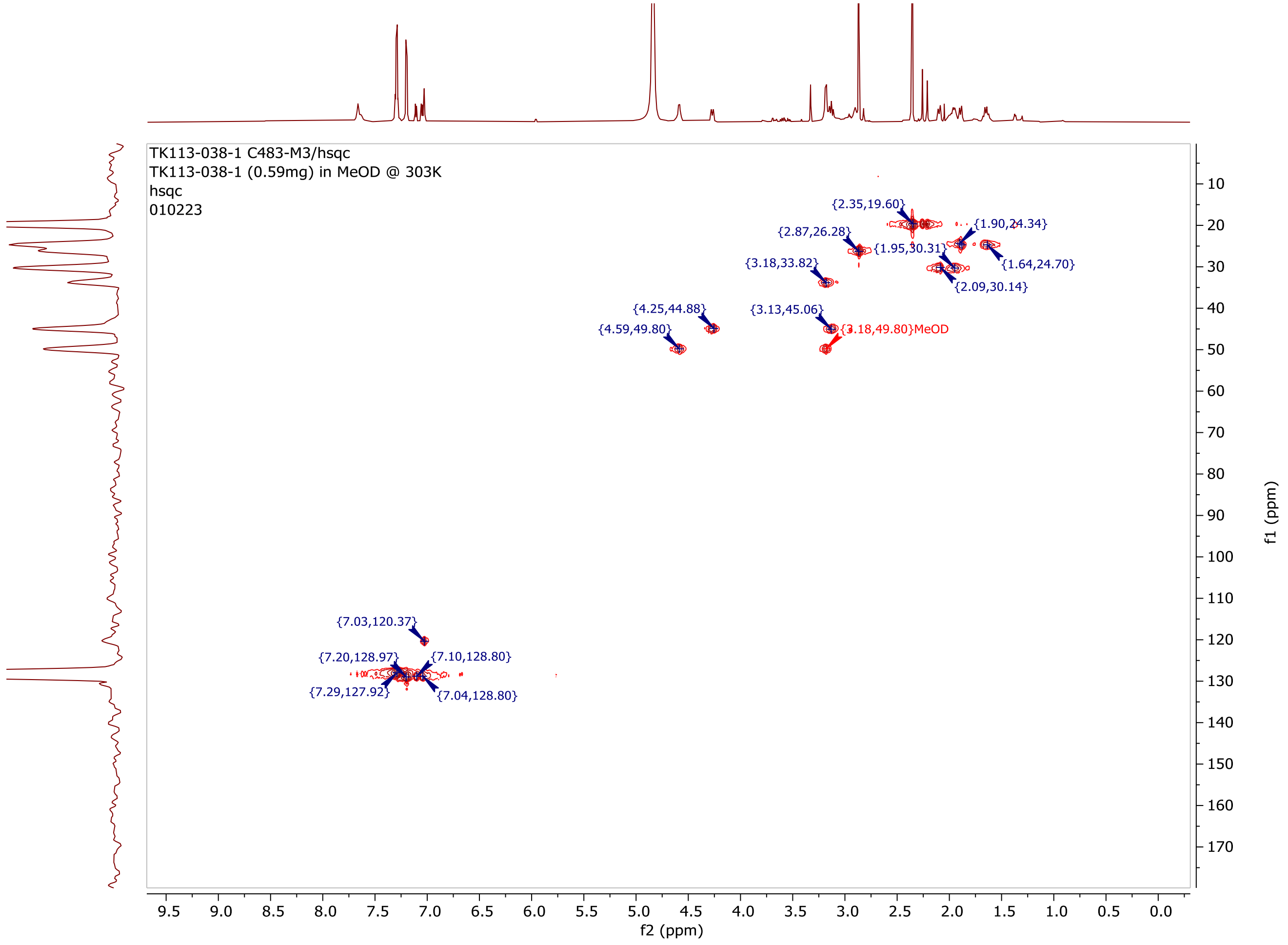
TK113-038-1 C483-M3/hsqc
TK113-038-1 (0.59mg) in MeOD @ 303K
hsqc
010223
{2.35,19.60}
{1.90,24.34}
{2.87,26.28}
{1.95,30.31}
{1.64,24.70}
{3.18,33.82}
{2.09,30.14}
{4.25,44.88}
{3.13,45.06}
{4.59,49.80}
{3.18,49.80}MeOD
{7.03,120.37}
{7.20,128.97}
{7.10,128.80}
{7.29,127.92}
{7.04,128.80}
f2 (ppm)
f1 (ppm)

HMBC

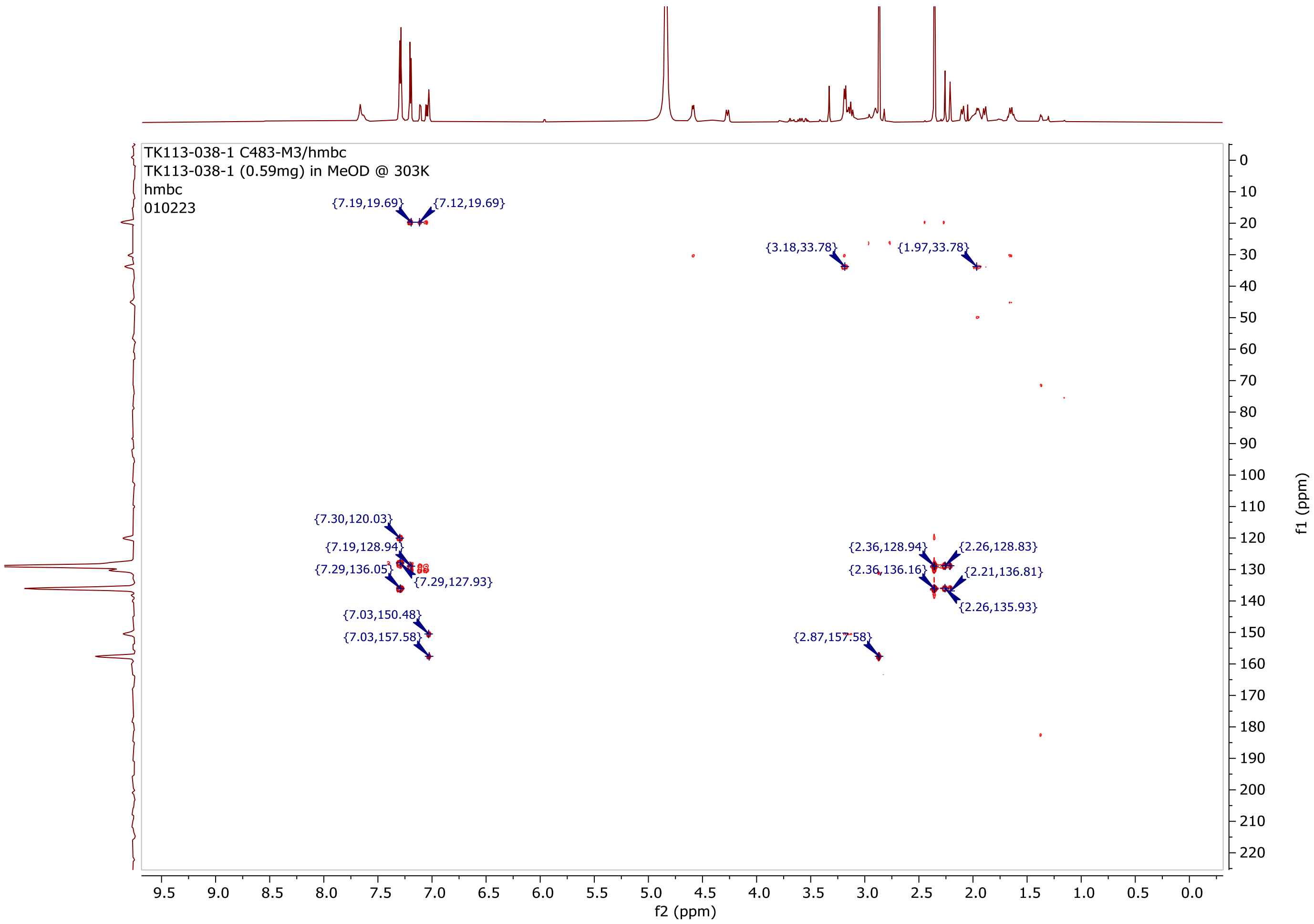
TK113-038-1 C483-M3/hmbc
TK113-038-1 (0.59mg) in MeOD @ 303K
hmbc
010223
{7.19,19.69}
{7.12,19.69}
{3.18,33.78}
{1.97,33.78}
{7.30,120.03}
{7.19,128.94}
{7.29,136.05}
{7.29,127.93}
{7.03,150.48}
{7.03,157.58}
{2.36,128.94}
{2.26,128.83}
{2.36,136.16}
{2.21,136.81}
{2.26,135.93}
{2.87,157.58}
f2 (ppm)
f1 (ppm)

Compound **7a**

^{1}H NMR

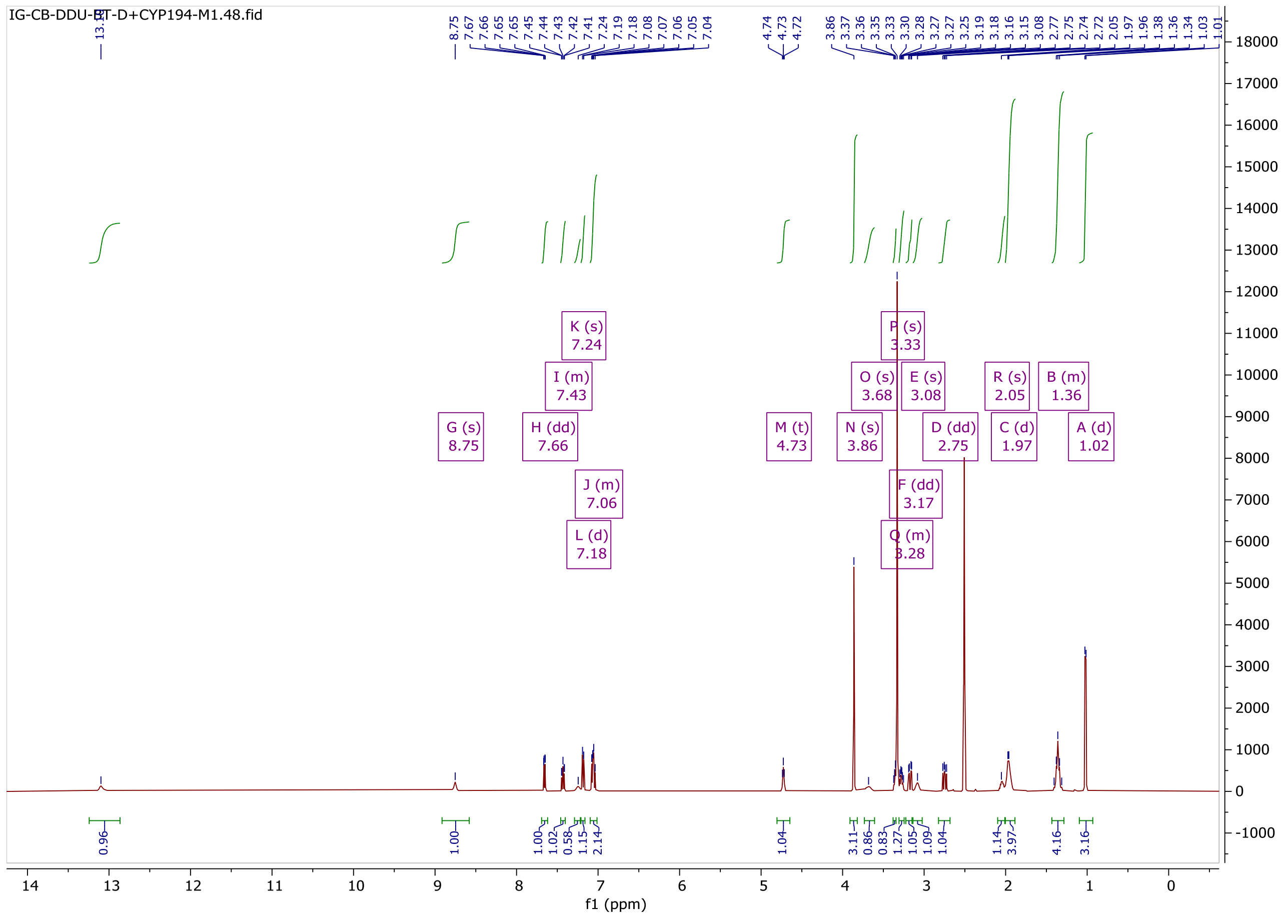

COSY

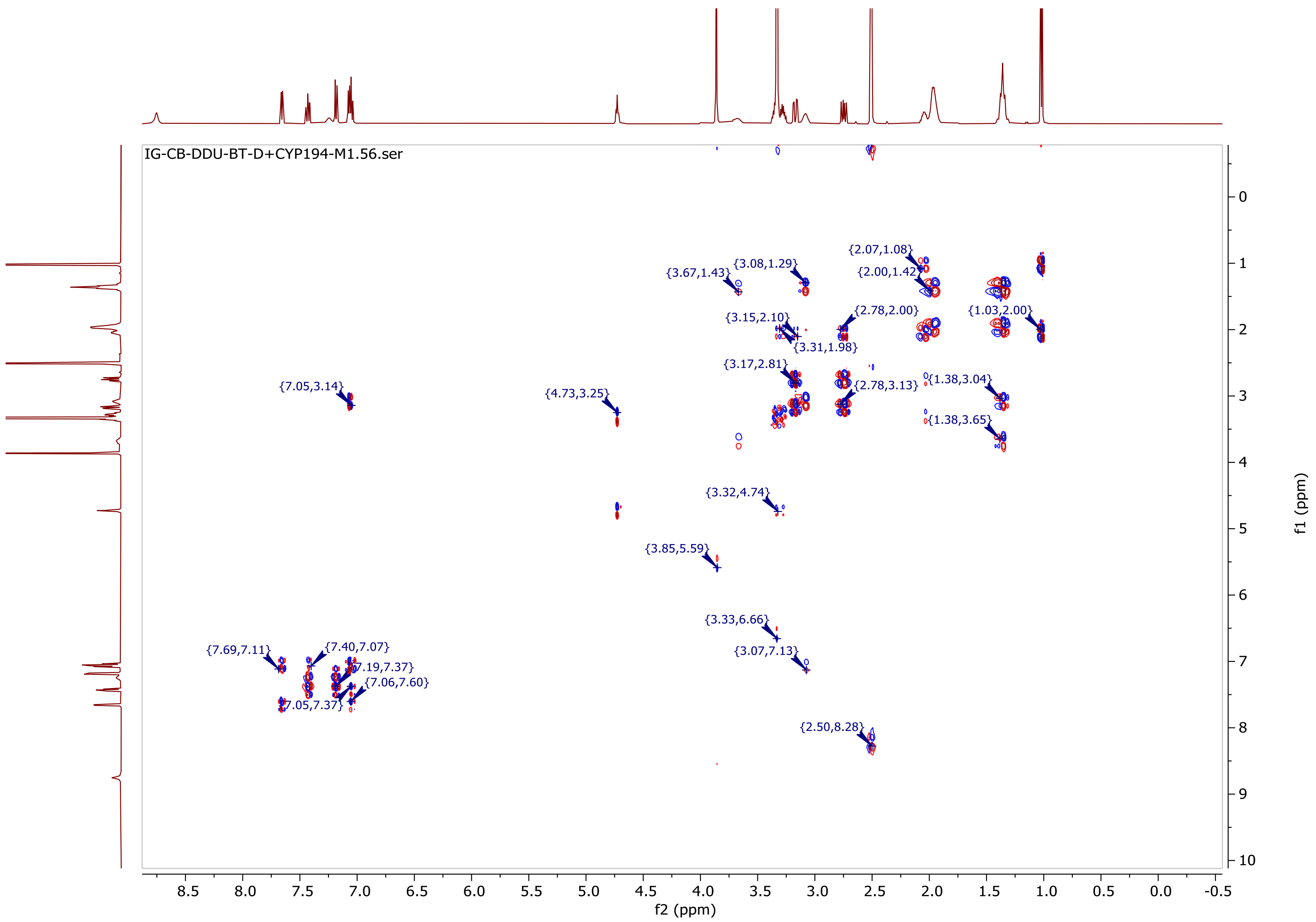
IG-CB-DDU-BT-D+CYP194-M1.56.ser
{2.07,1.08}
{3.08,1.29}
{3.67,1.43}
{2.00,1.42}
{3.15,2.10}
{2.78,2.00}
{1.03,2.00}
{3.31,1.98}
{3.17,2.81}
{2.78,3.13}
{1.38,3.04}
{7.05,3.14}
{4.73,3.25}
{1.38,3.65}
{3.32,4.74}
{3.85,5.59}
{3.33,6.66}
{7.69,7.11}
{7.40,7.07}
{3.07,7.13}
{7.19,7.37}
{7.06,7.60}
{7.05,7.37}
{2.50,8.28}
f2 (ppm)
f1 (ppm)

HSQC

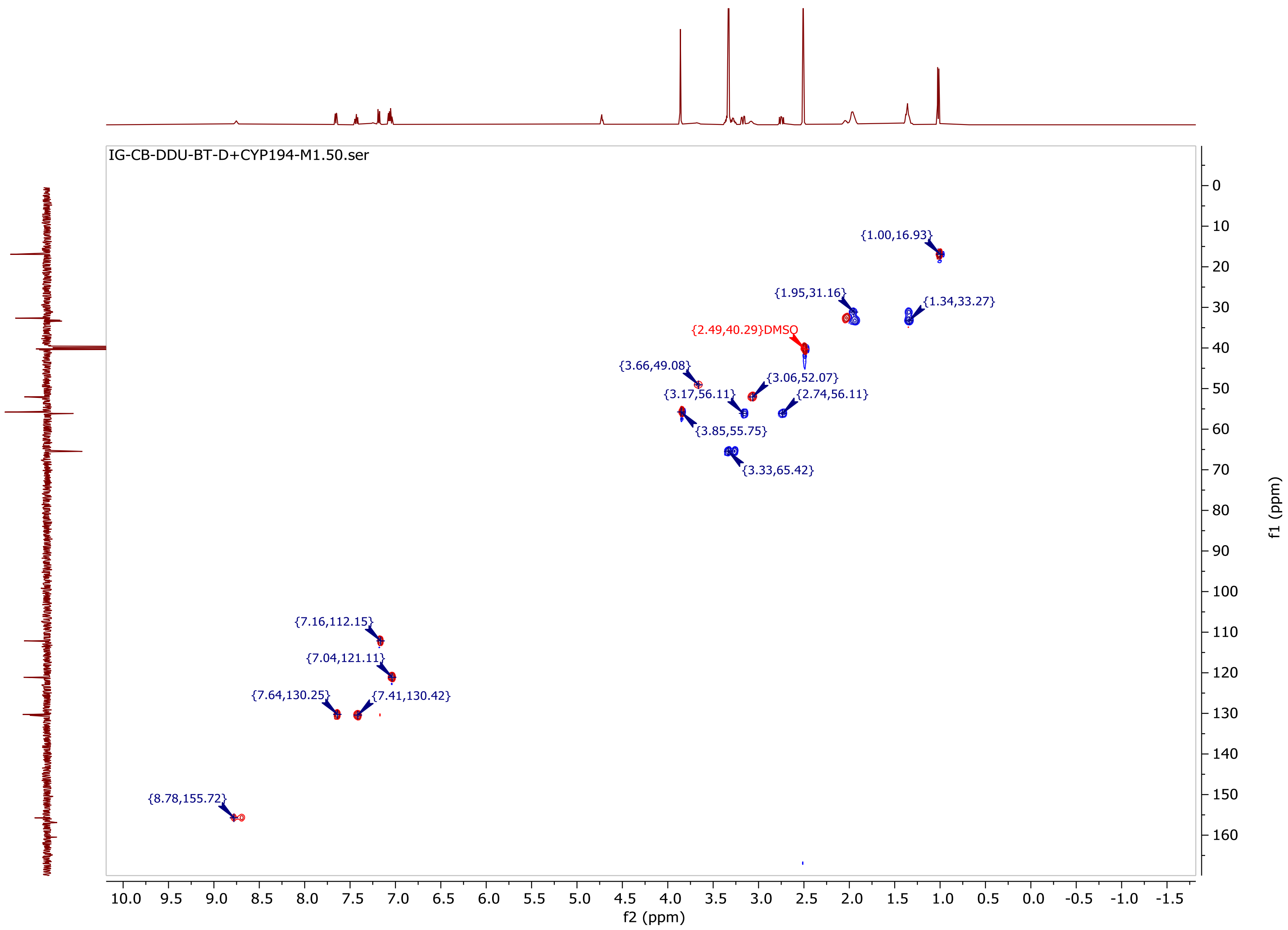
IG-CB-DDU-BT-D+CYP194-M1.50.ser
{1.00,16.93}
{1.95,31.16}
{1.34,33.27}
{2.49,40.29}DMSO
{3.66,49.08}
{3.06,52.07}
{3.17,56.11}
{2.74,56.11}
{3.85,55.75}
{3.33,65.42}
{7.16,112.15}
{7.04,121.11}
{7.64,130.25}
{7.41,130.42}
{8.78,155.72}
f2 (ppm)
f1 (ppm)

HMBC

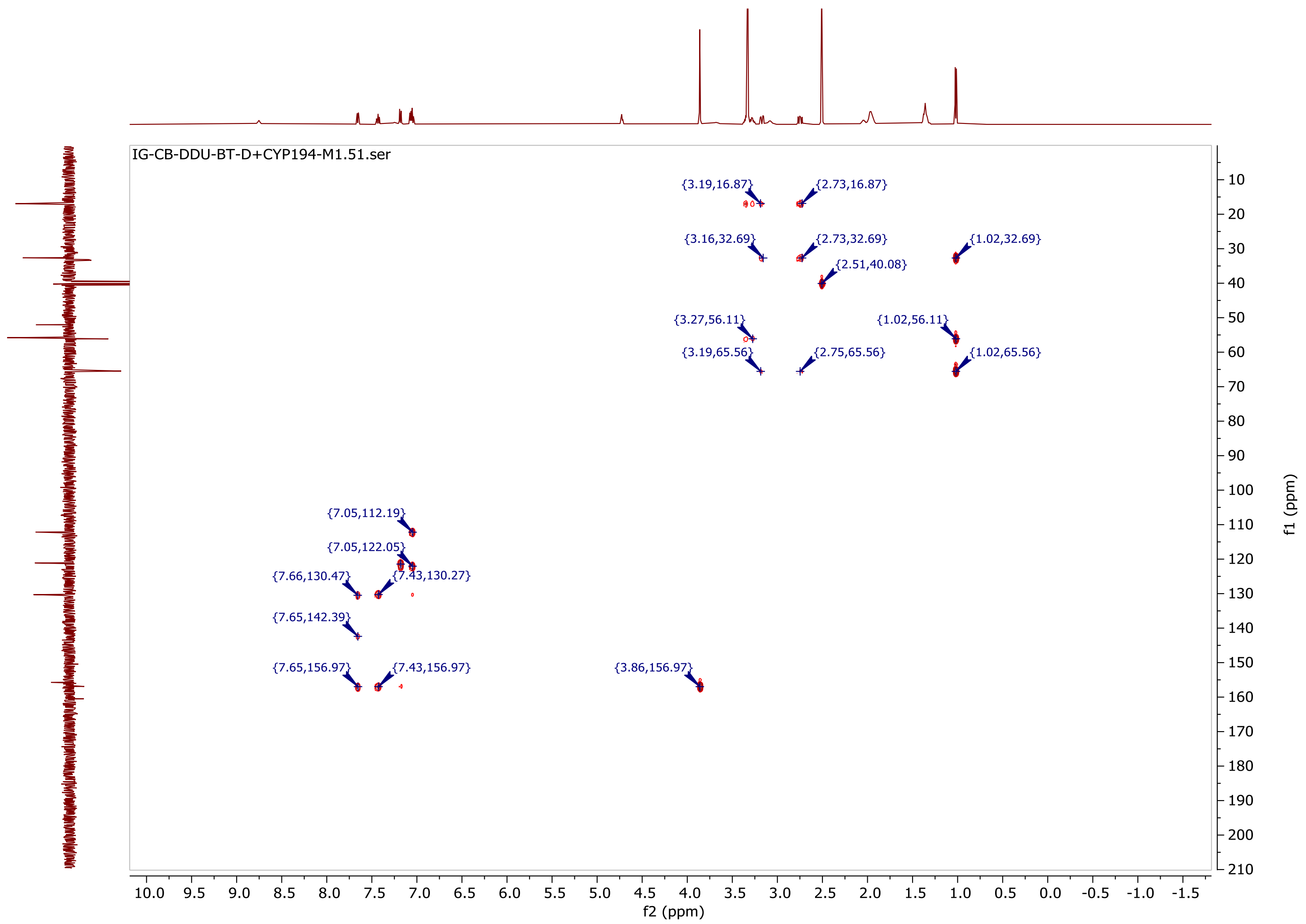

IG-CB-DDU-BT-D+CYP194-M1.51.ser
{3.19,16.87}
{2.73,16.87}
{3.16,32.69}
{2.73,32.69}
{1.02,32.69}
{2.51,40.08}
{3.27,56.11}
{1.02,56.11}
{3.19,65.56}
{2.75,65.56}
{1.02,65.56}
{7.05,112.19}
{7.05,122.05}
{7.66,130.47}
{7.43,130.27}
{7.65,142.39}
{7.65,156.97}
{7.43,156.97}
{3.86,156.97}
f2 (ppm)
f1 (ppm)

NOESY

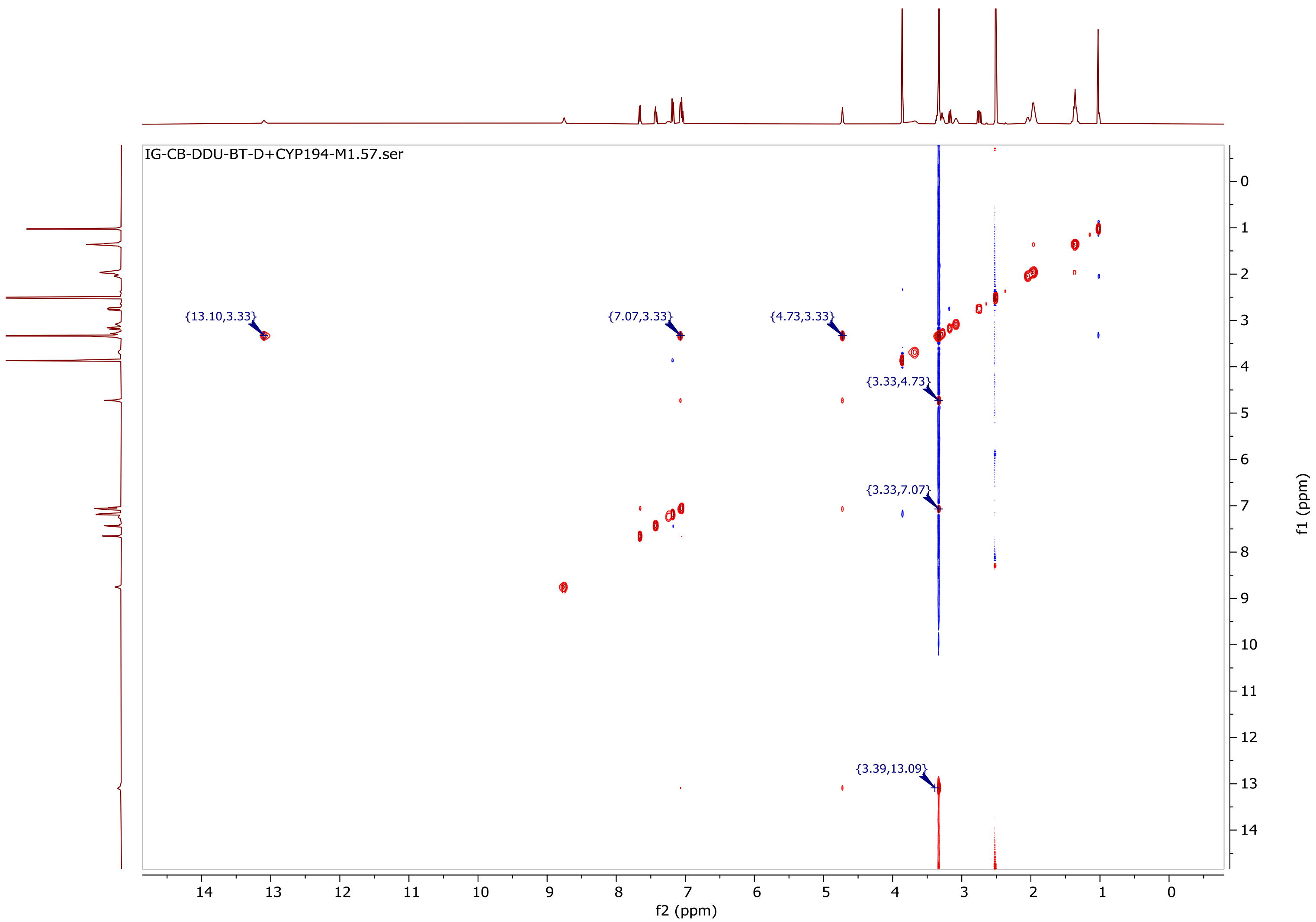
IG-CB-DDU-BT-D+CYP194-M1.57.ser
{13.10,3.33}
{7.07,3.33}
{4.73,3.33}
{3.33,4.73}
{3.33,7.07}
{3.39,13.09}
f2 (ppm)
f1 (ppm)

DEPTqgpsp

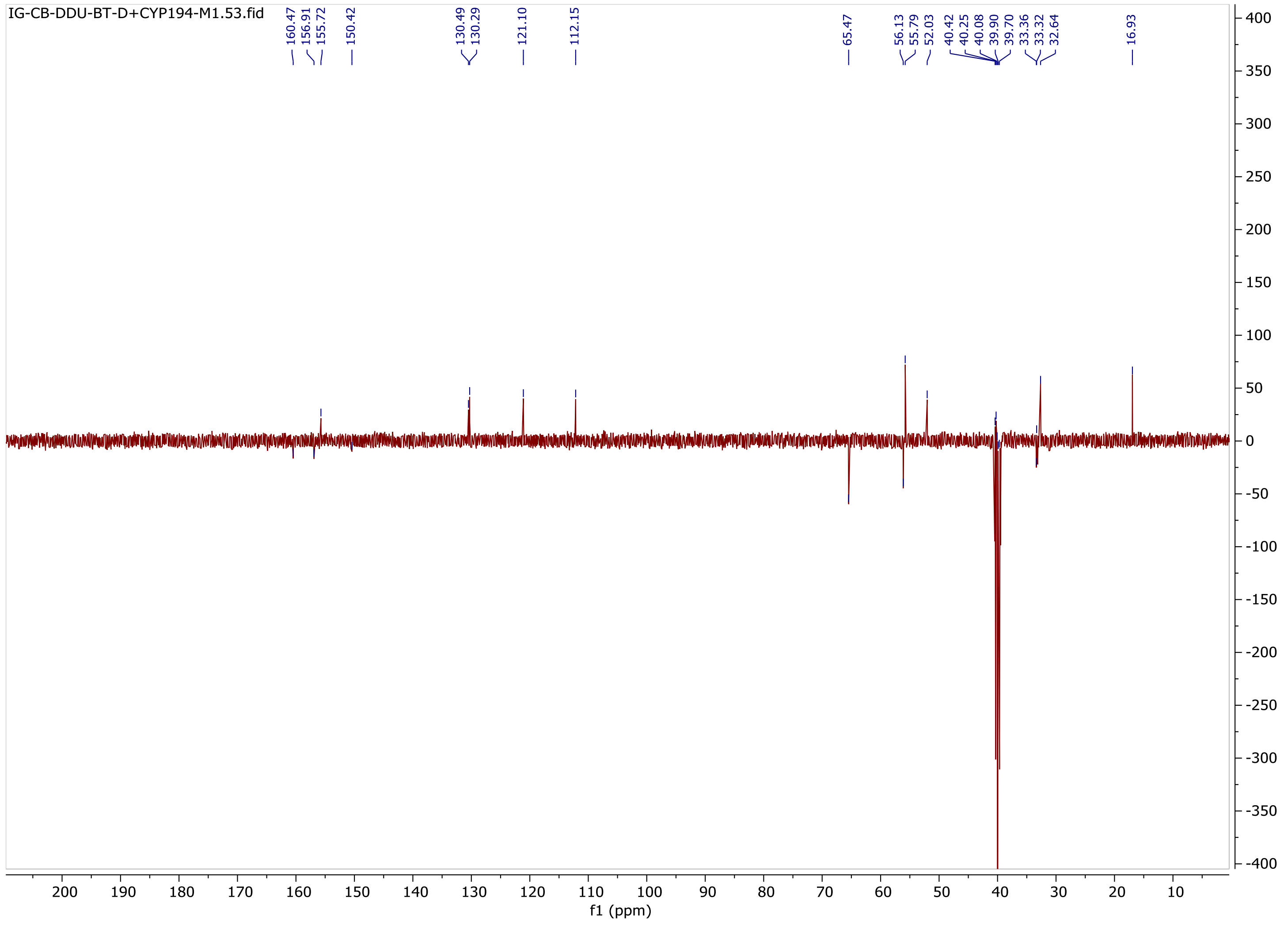
IG-CB-DDU-BT-D+CYP194-M1.53.fid
160.47
156.91
155.72
150.42
130.49
130.29
121.10
112.15
65.47
56.13
55.79
52.03
40.42
40.25
40.08
39.90
39.70
33.36
33.32
32.64
16.93
f1 (ppm)

DEPT-90

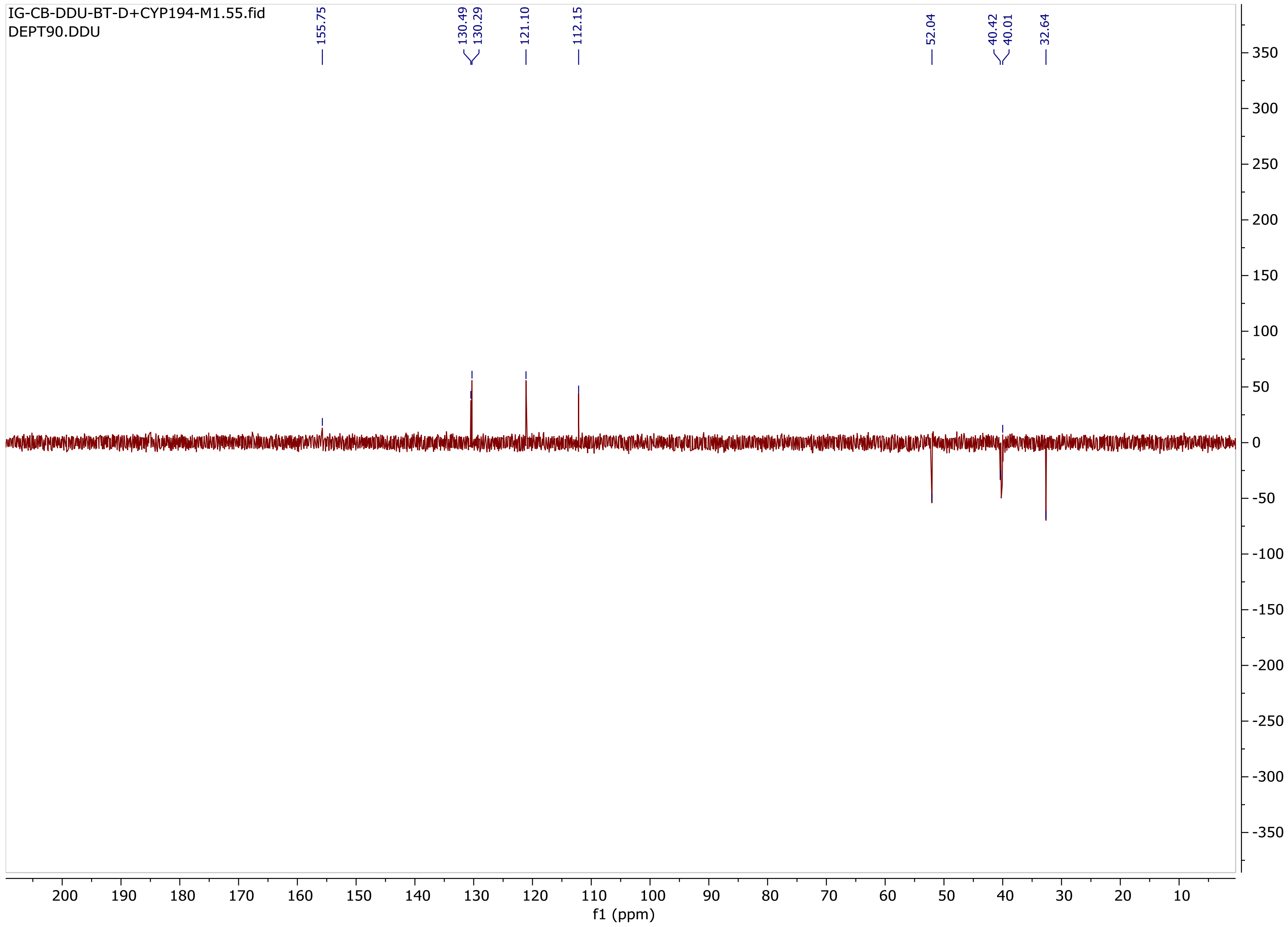

IG-CB-DDU-BT-D+CYP194-M1.55.fid
DEPT90.DDU
155.75
130.49
130.29
121.10
112.15
52.04
40.42
40.01
32.64
350
300
250
200
150
100
50
0
-50
-100
-150
-200
-250
-300
-350
200 190 180 170 160 150 140 130 120 110 100 90 80 70 60 50 40 30 20 10
f1 (ppm)

DEPT-135

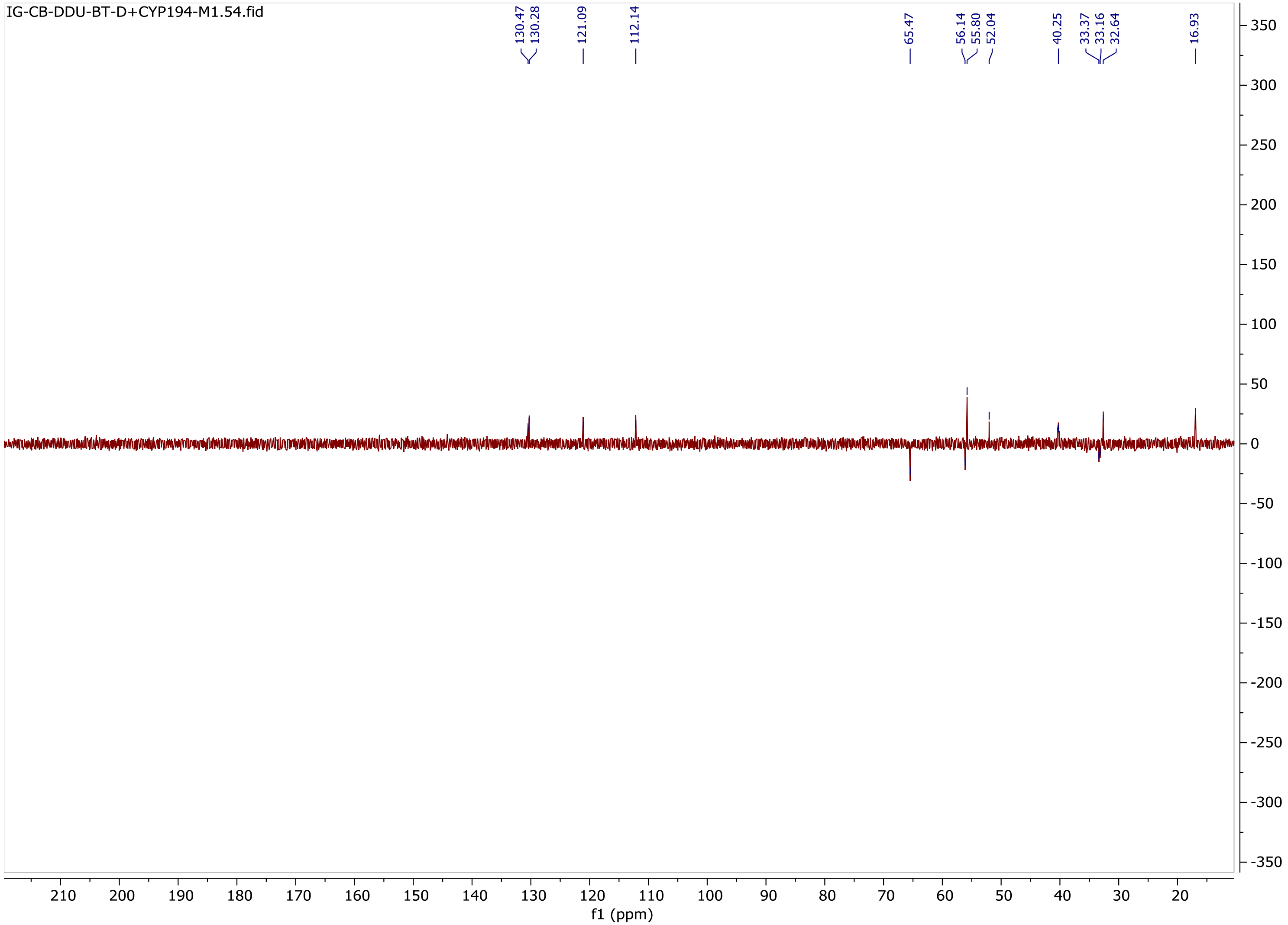
IG-CB-DDU-BT-D+CYP194-M1.54.fid
130.47
130.28
121.09
112.14
65.47
56.14
55.80
52.04
40.25
33.37
33.16
32.64
16.93
350
300
250
200
150
100
50
0
-50
-100
-150
-200
-250
-300
-350
210
200
190
180
170
160
150
140
130
120
110
100
90
80
70
60
50
40
30
20
f1 (ppm)

HRMS

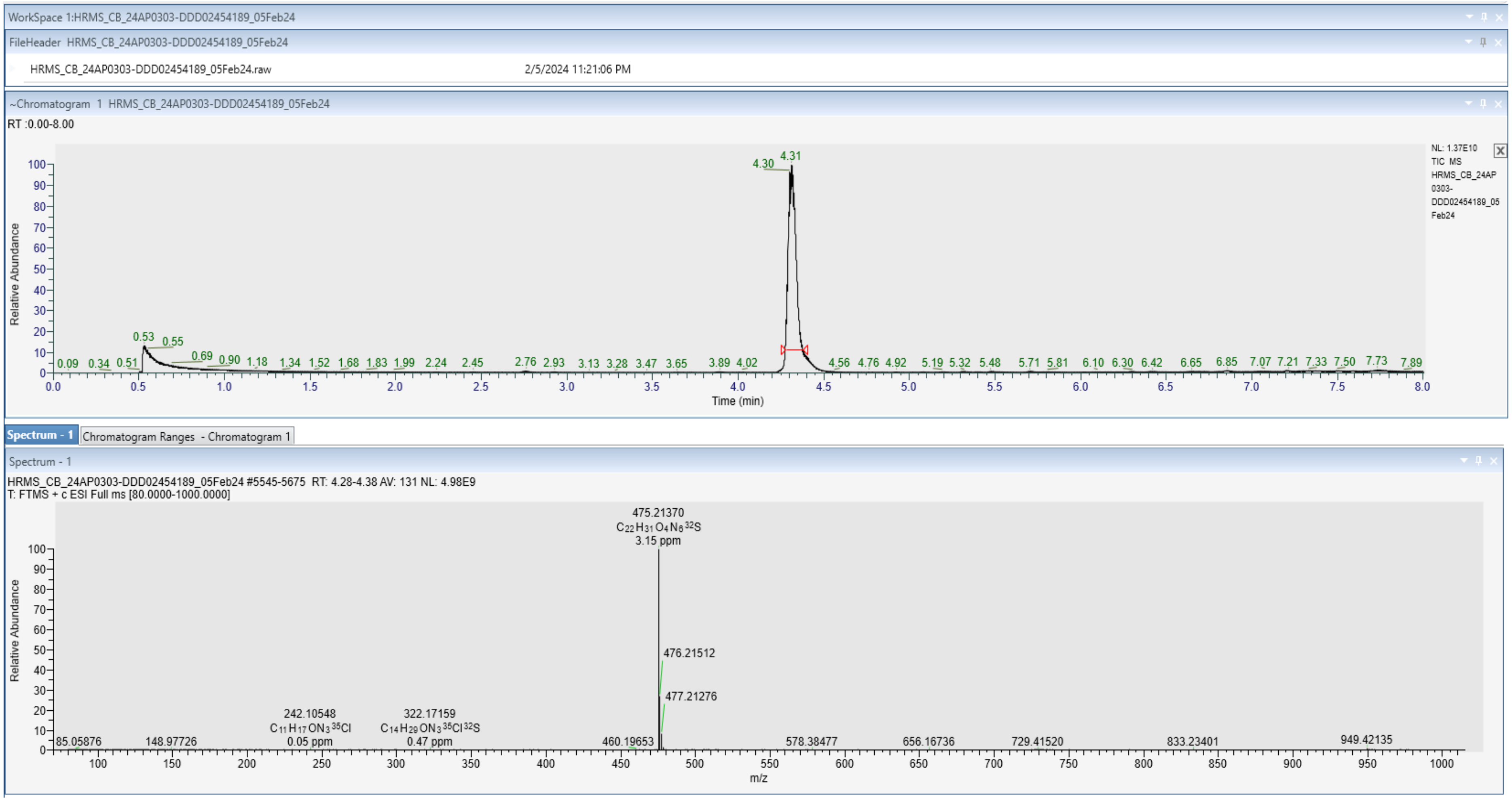
WorkSpace 1:HRMS_CB_24AP0303-DDD02454189_05Feb24
FileHeader HRMS_CB_24AP0303-DDD02454189_05Feb24
HRMS_CB_24AP0303-DDD02454189_05Feb24.raw
2/5/2024 11:21:06 PM
~Chromatogram 1 HRMS_CB_24AP0303-DDD02454189_05Feb24
RT :0.00-8.00
NL: 1.37E10
TIC MS
HRMS_CB_24AP0303-DDD02454189_05Feb24
4.30
4.31
Relative Abundance
Time (min)
Spectrum - 1
Chromatogram Ranges - Chromatogram 1
HRMS_CB_24AP0303-DDD02454189_05Feb24 #5545-5675 RT: 4.28-4.38 AV: 131 NL: 4.98E9
T: FTMS + c ESI Full ms [80.0000-1000.0000]
475.21370
C22 H31 O4 N8 32S
3.15 ppm
476.21512
477.21276
242.10548
C11 H17 ON3 35Cl
0.05 ppm
322.17159
C14 H29 ON3 35Cl 32S
0.47 ppm
85.05876
148.97726
460.19653
578.38477
656.16736
729.41520
833.23401
949.42135
m/z

Compound **7b**

^{1}H NMR

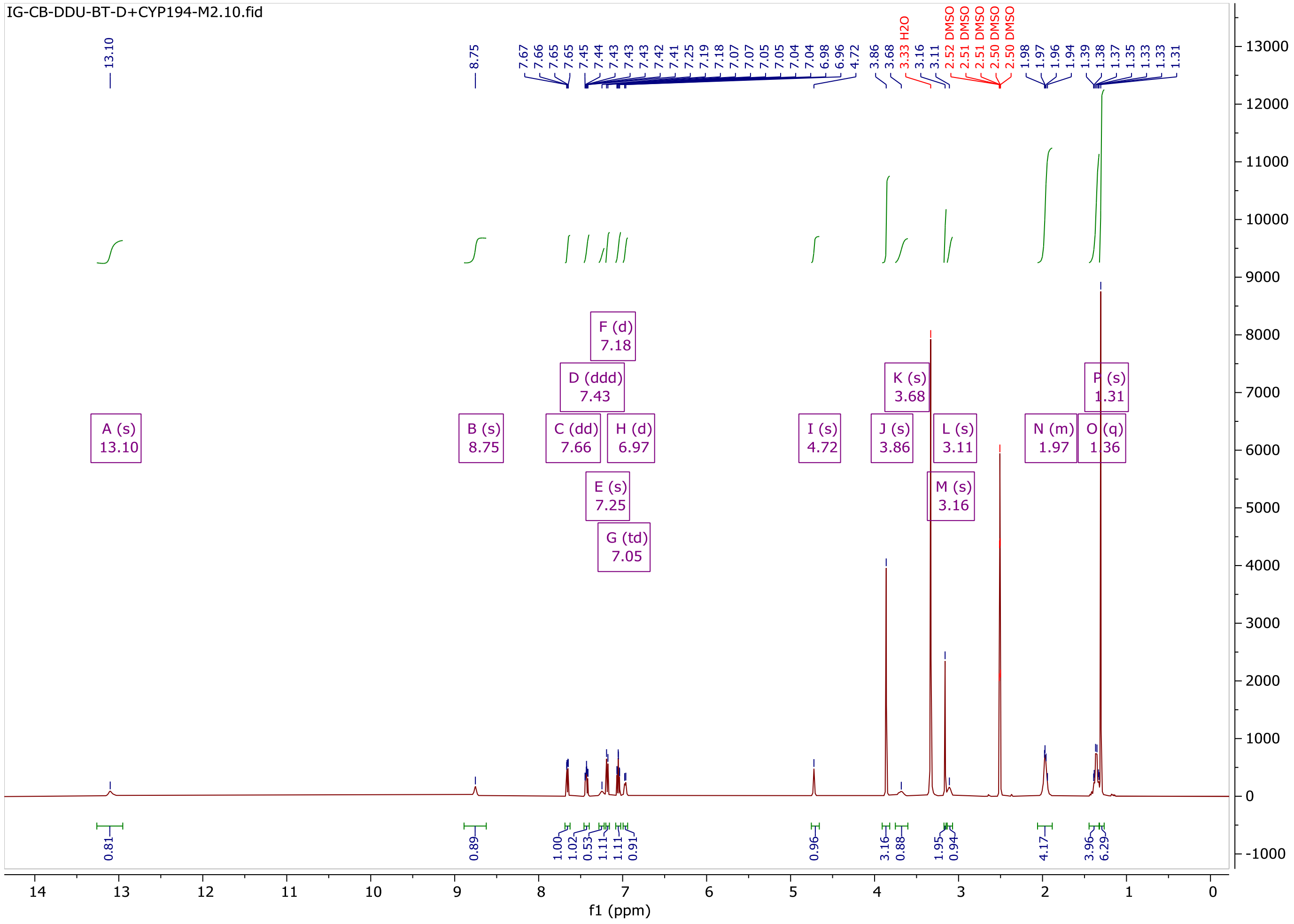

COSY

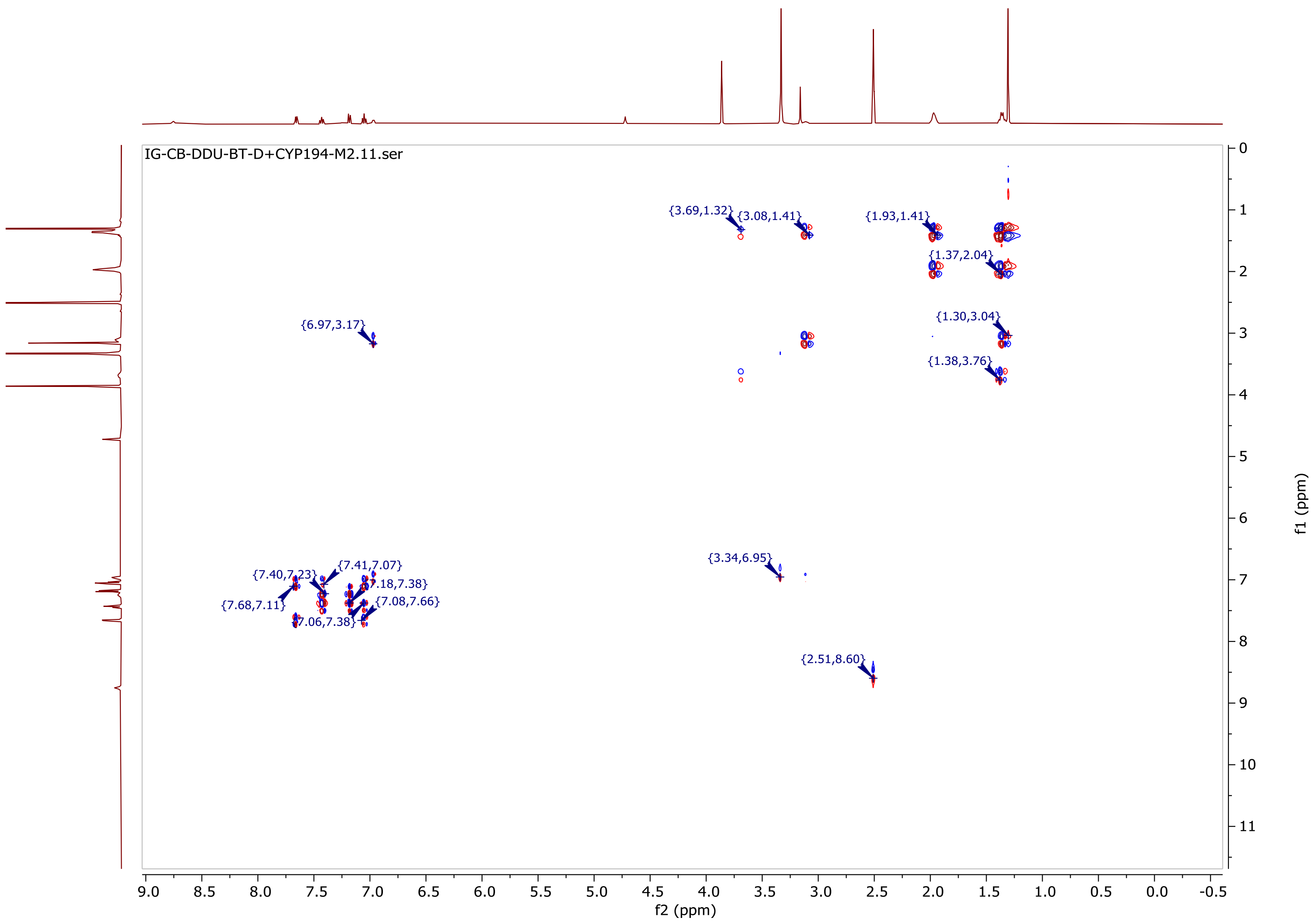

IG-CB-DDU-BT-D+CYP194-M2.11.ser
{3.69,1.32}
{3.08,1.41}
{1.93,1.41}
{1.37,2.04}
{6.97,3.17}
{1.30,3.04}
{1.38,3.76}
{3.34,6.95}
{7.41,7.07}
{7.40,7.23}
{7.18,7.38}
{7.68,7.11}
{7.08,7.66}
{7.06,7.38}
{2.51,8.60}
f2 (ppm)
f1 (ppm)

HSQC

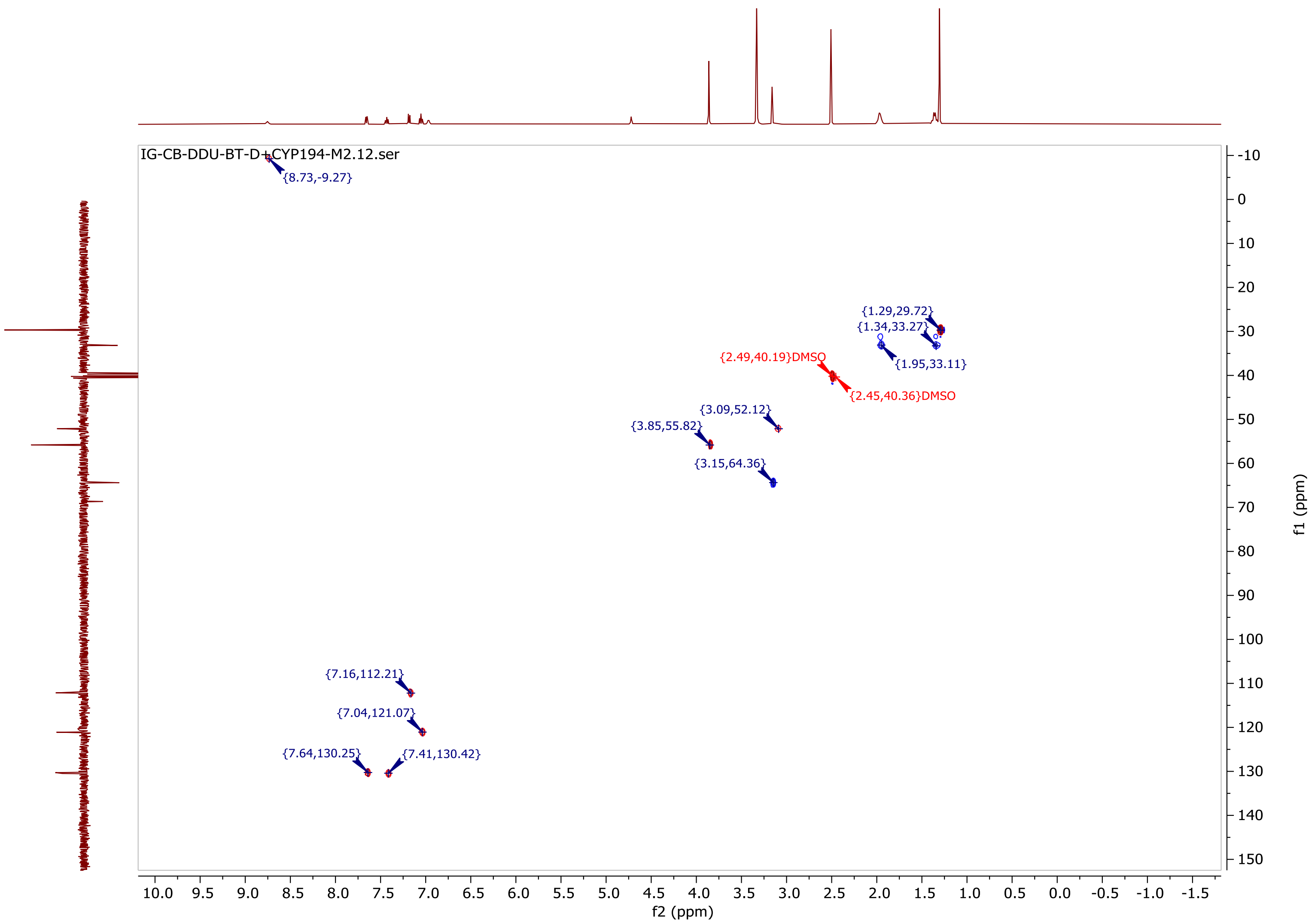
IG-CB-DDU-BT-D+CYP194-M2.12.ser
{8.73,-9.27}
{1.29,29.72}
{1.34,33.27}
{1.95,33.11}
{2.49,40.19}DMSO
{2.45,40.36}DMSO
{3.09,52.12}
{3.85,55.82}
{3.15,64.36}
{7.16,112.21}
{7.04,121.07}
{7.64,130.25}
{7.41,130.42}
f2 (ppm)
f1 (ppm)

HMBC

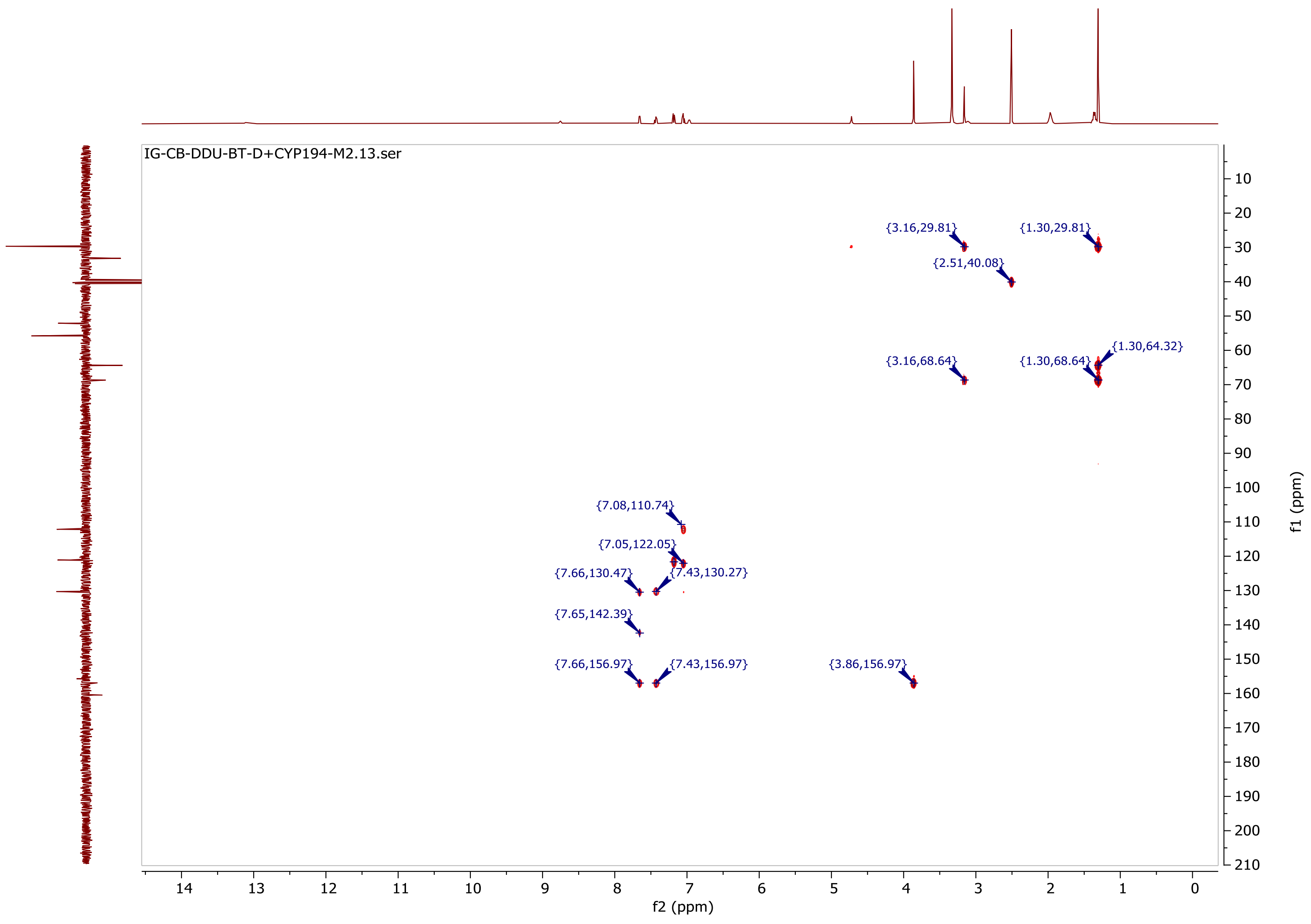
IG-CB-DDU-BT-D+CYP194-M2.13.ser
{3.16,29.81}
{1.30,29.81}
{2.51,40.08}
{1.30,64.32}
{3.16,68.64}
{1.30,68.64}
{7.08,110.74}
{7.05,122.05}
{7.66,130.47}
{7.43,130.27}
{7.65,142.39}
{7.66,156.97}
{7.43,156.97}
{3.86,156.97}
f2 (ppm)
f1 (ppm)

NOESY

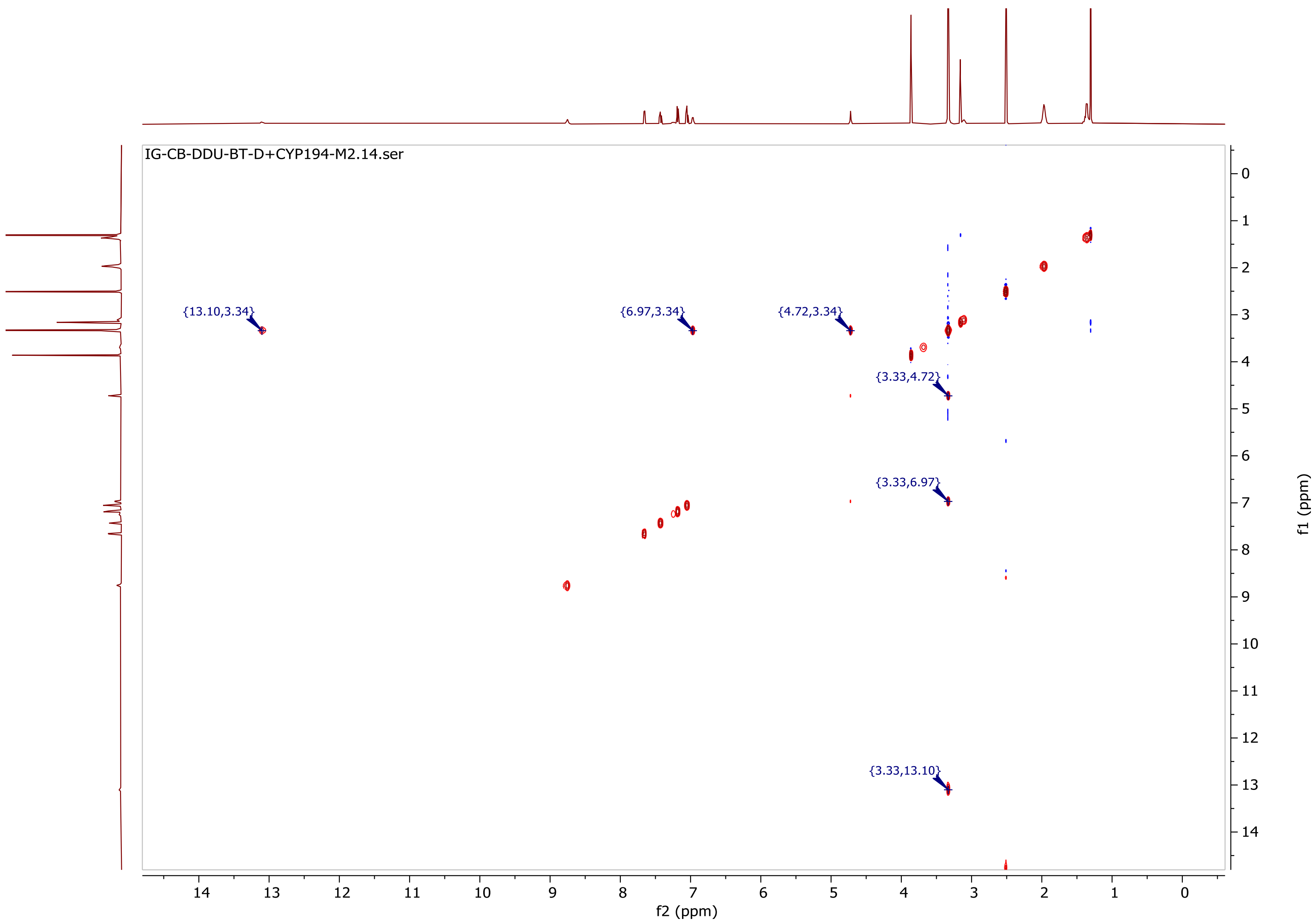
IG-CB-DDU-BT-D+CYP194-M2.14.ser
{13.10,3.34}
{6.97,3.34}
{4.72,3.34}
{3.33,4.72}
{3.33,6.97}
{3.33,13.10}
f2 (ppm)
f1 (ppm)

DEPTqgpsp

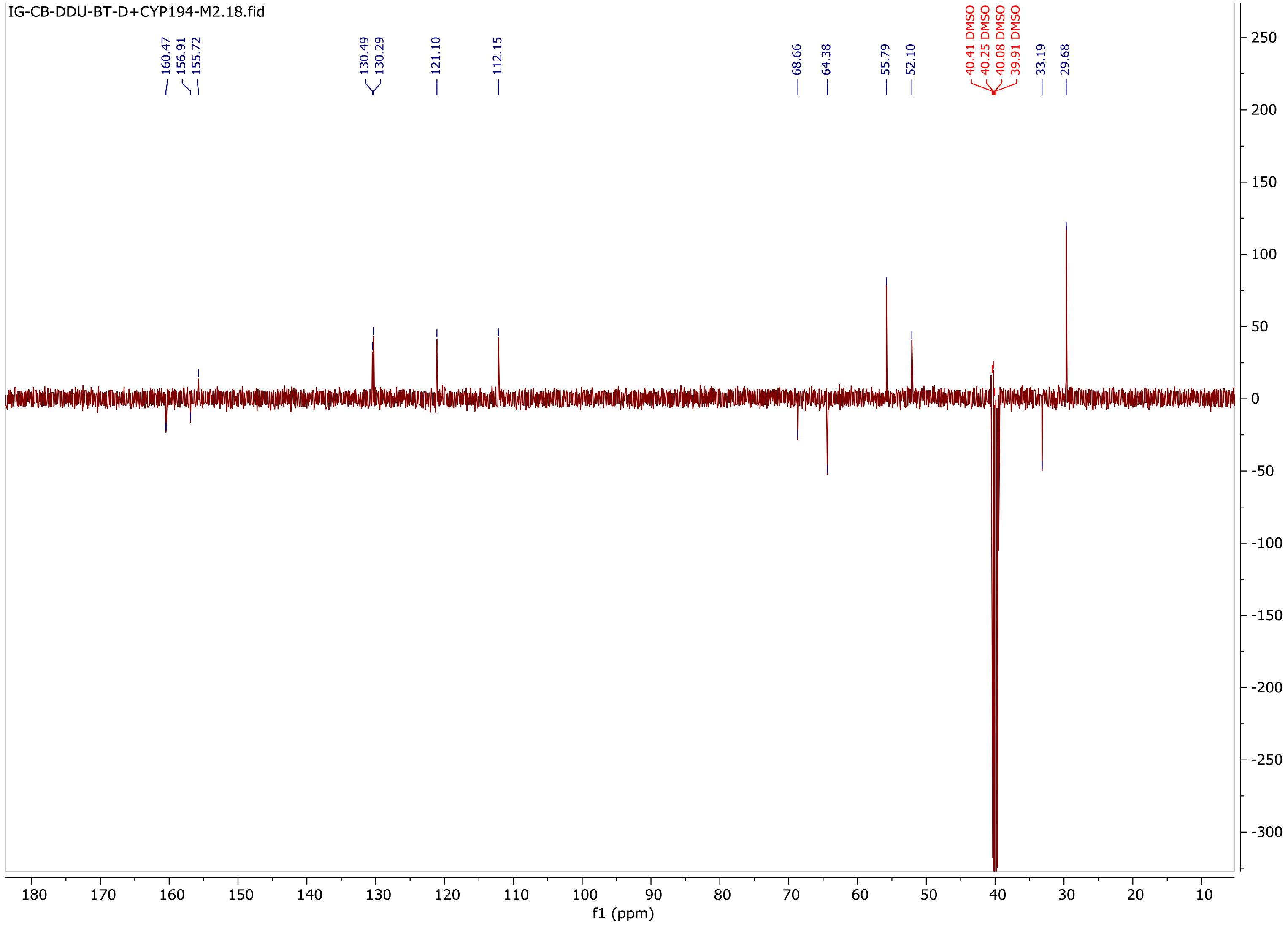

IG-CB-DDU-BT-D+CYP194-M2.18.fid
160.47
156.91
155.72
130.49
130.29
121.10
112.15
68.66
64.38
55.79
52.10
40.41 DMSO
40.25 DMSO
40.08 DMSO
39.91 DMSO
33.19
29.68
250
200
150
100
50
0
-50
-100
-150
-200
-250
-300
180
170
160
150
140
130
120
110
100
90
80
70
60
50
40
30
20
10
f1 (ppm)

DEPT-90

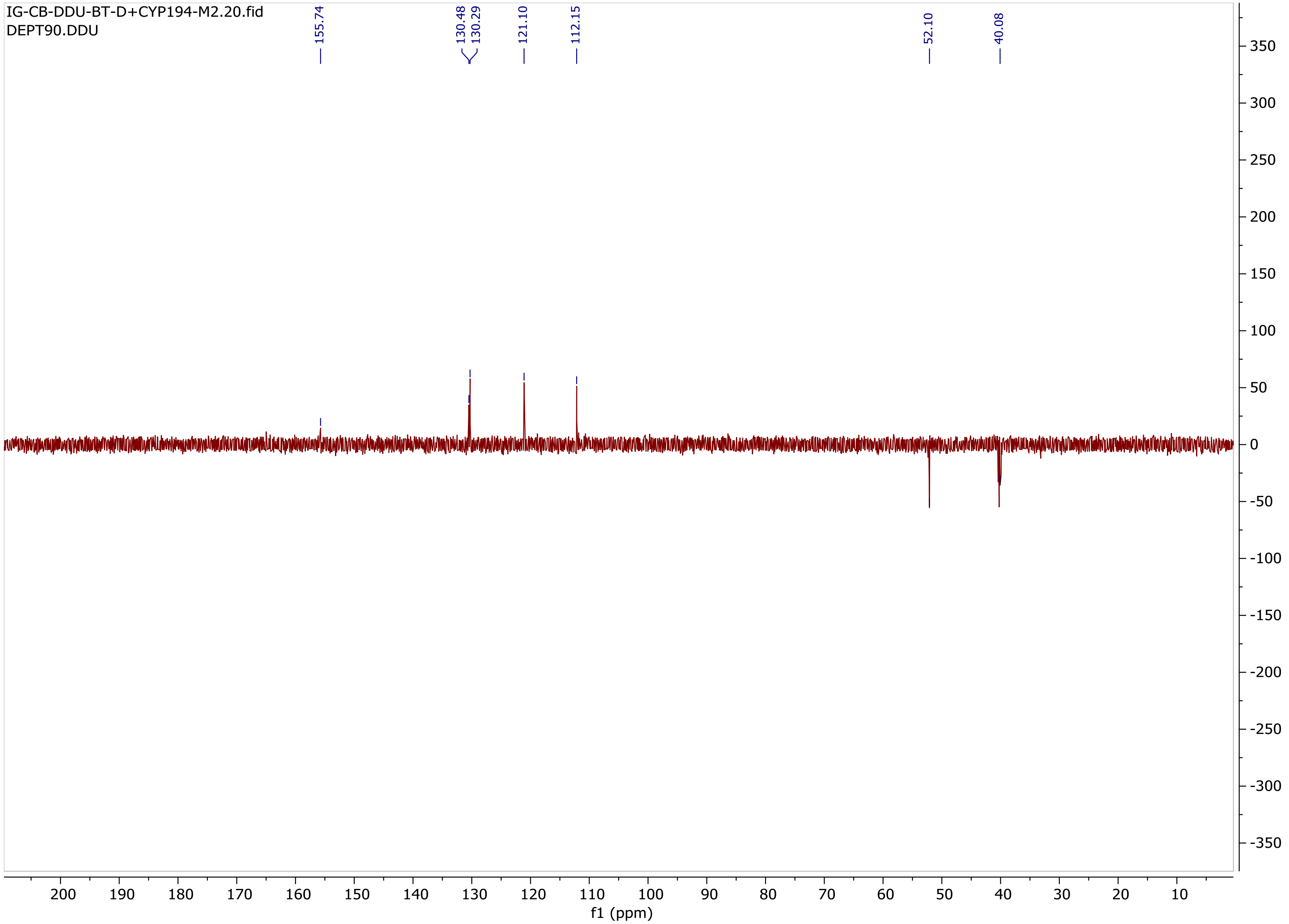

IG-CB-DDU-BT-D+CYP194-M2.20.fid
DEPT90.DDU
155.74
130.48
130.29
121.10
112.15
52.10
40.08
350
300
250
200
150
100
50
0
-50
-100
-150
-200
-250
-300
-350
200
190
180
170
160
150
140
130
120
110
100
90
80
70
60
50
40
30
20
10
f1 (ppm)

DEPT-135

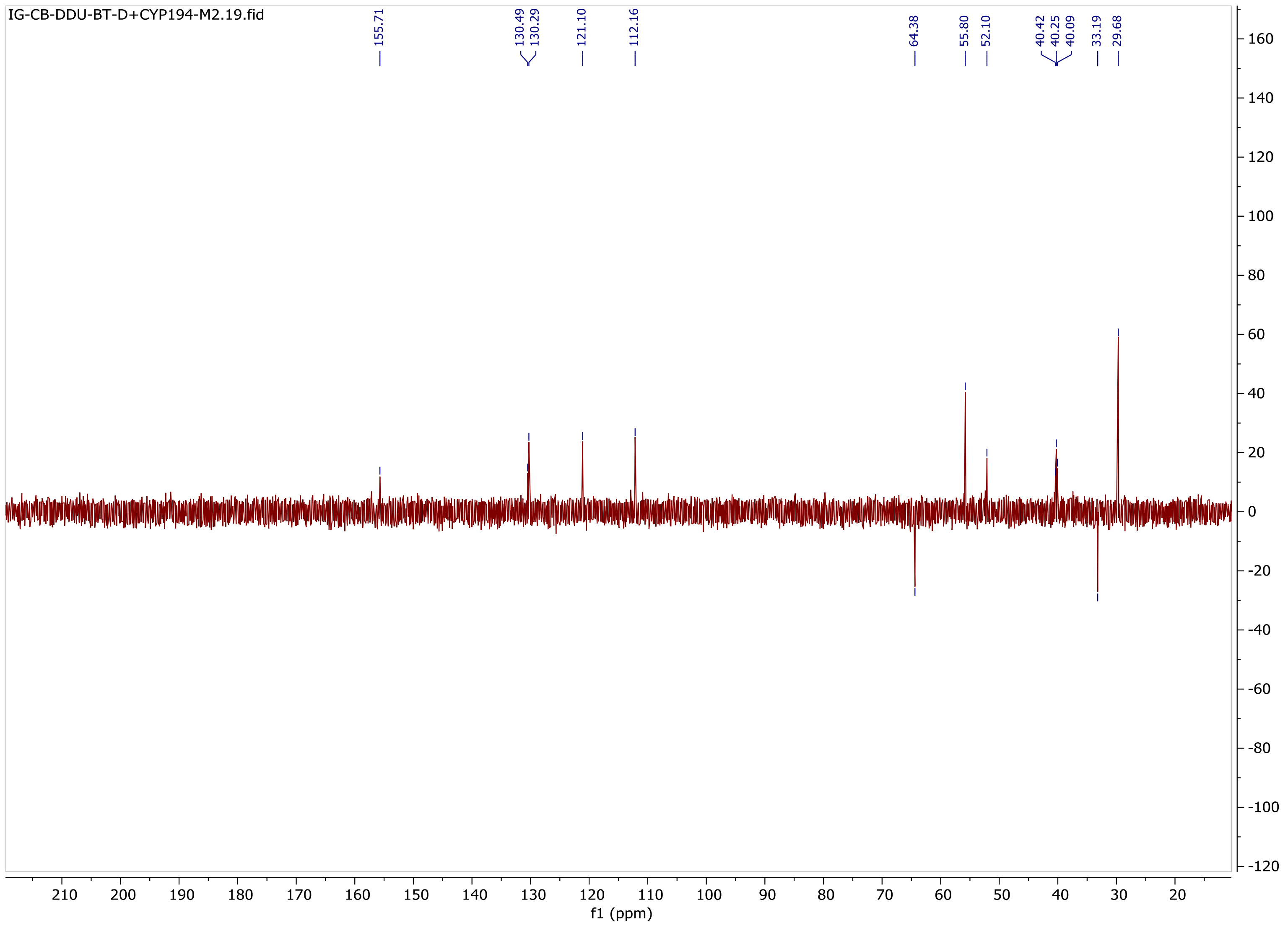
IG-CB-DDU-BT-D+CYP194-M2.19.fid
155.71
130.49
130.29
121.10
112.16
64.38
55.80
52.10
40.42
40.25
40.09
33.19
29.68
f1 (ppm)

HRMS

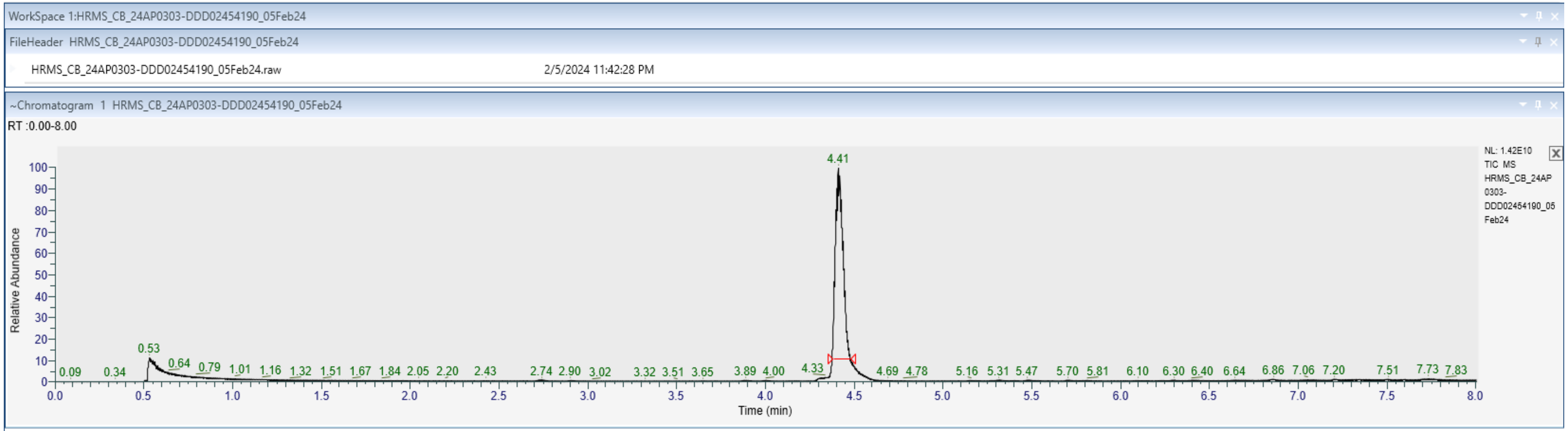

WorkSpace 1:HRMS_CB_24AP0303-DDD02454190_05Feb24
FileHeader HRMS_CB_24AP0303-DDD02454190_05Feb24
HRMS_CB_24AP0303-DDD02454190_05Feb24.raw
2/5/2024 11:42:28 PM
~Chromatogram 1 HRMS_CB_24AP0303-DDD02454190_05Feb24
RT :0.00-8.00
NL: 1.42E10
TIC MS
HRMS_CB_24AP0303-DDD02454190_05Feb24
4.41
Relative Abundance
Time (min)

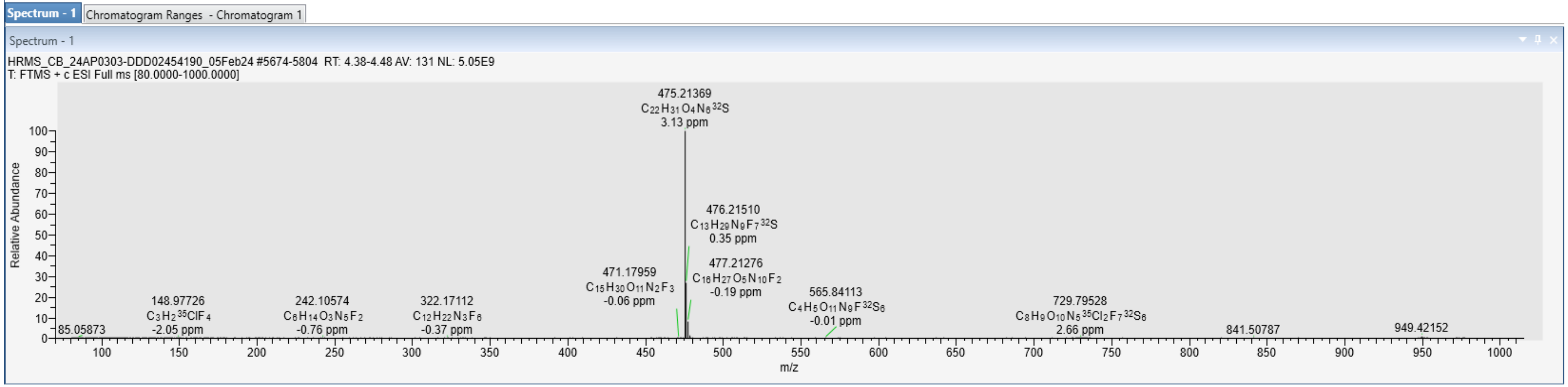

Spectrum - 1
Chromatogram Ranges - Chromatogram 1
HRMS_CB_24AP0303-DDD02454190_05Feb24 #5674-5804 RT: 4.38-4.48 AV: 131 NL: 5.05E9
T: FTMS + c ESI Full ms [80.0000-1000.0000]
475.21369
C22 H31 O4 N8 32S
3.13 ppm
476.21510
C13 H29 N9 F7 32S
0.35 ppm
477.21276
C16 H27 O5 N10 F2
-0.19 ppm
471.17959
C15 H30 O11 N2 F3
-0.06 ppm
565.84113
C4 H5 O11 N9 F 32S6
-0.01 ppm
729.79528
C8 H9 O10 N5 35Cl2 F7 32S8
2.66 ppm
148.97726
C3 H2 35ClF4
-2.05 ppm
242.10574
C8 H14 O3 N5 F2
-0.76 ppm
322.17112
C12 H22 N3 F8
-0.37 ppm
85.05873
841.50787
949.42152
Relative Abundance
m/z

Compound **8a**

^1H NMR

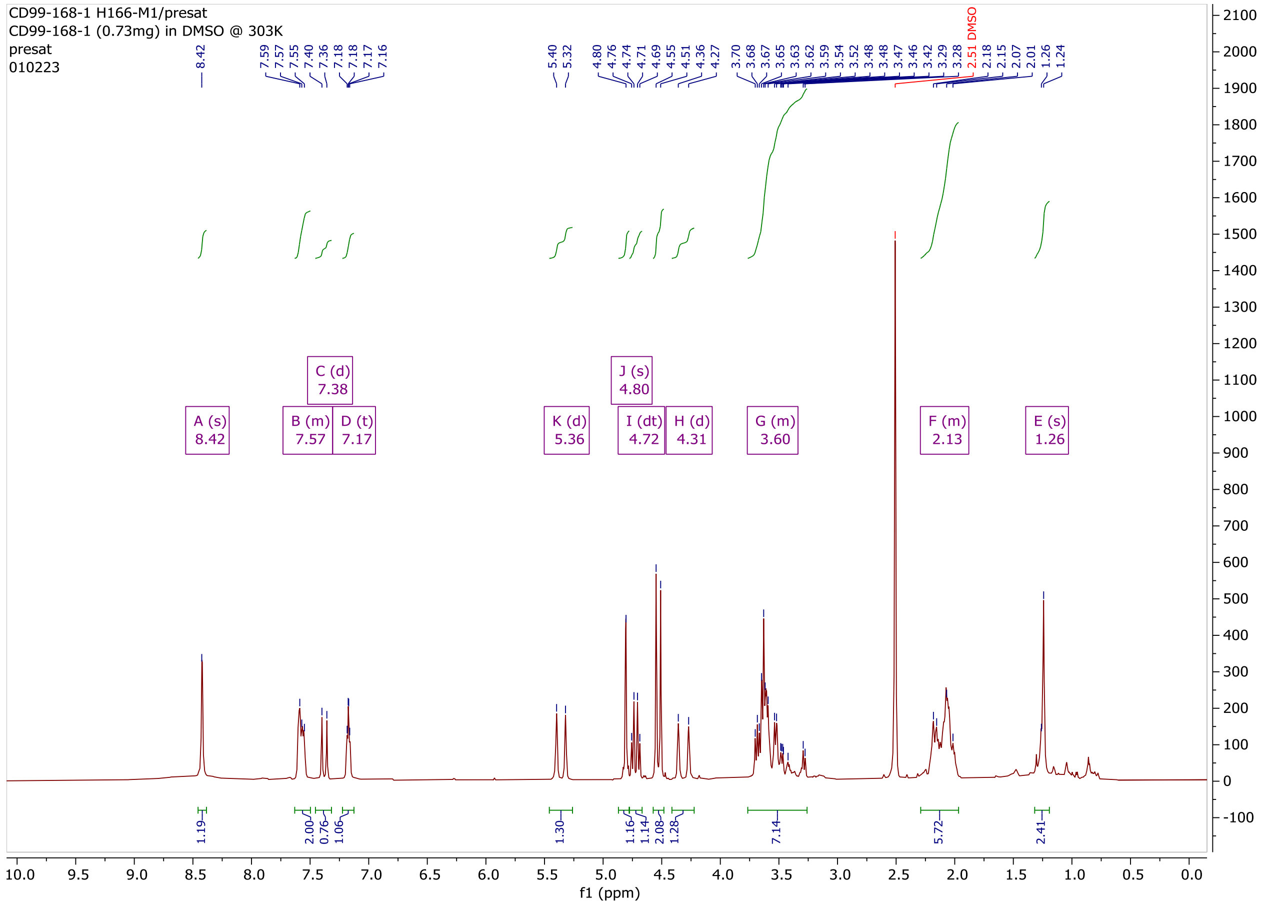

COSY

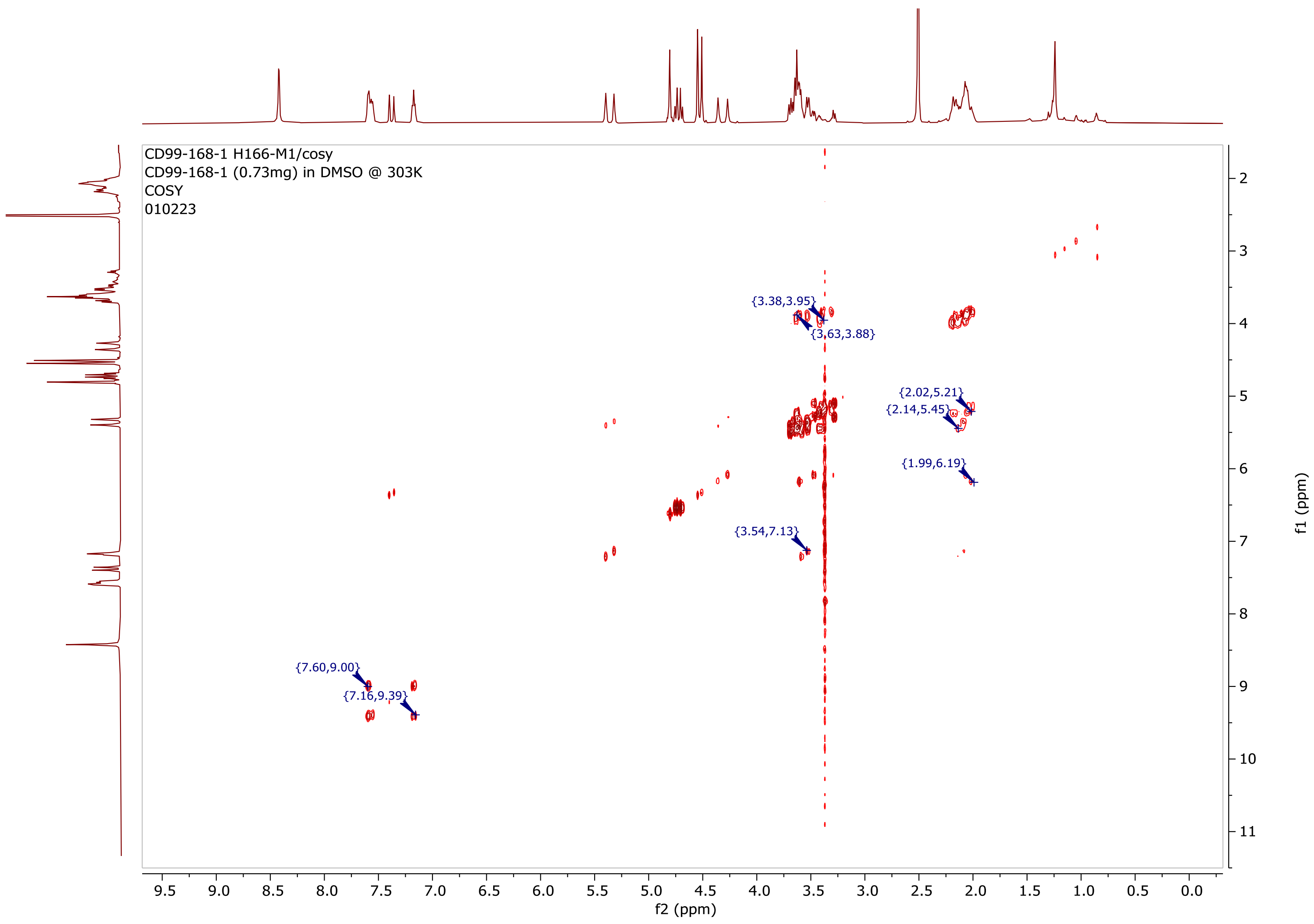

CD99-168-1 H166-M1/cosy
CD99-168-1 (0.73mg) in DMSO @ 303K
COSY
010223
{3.38,3.95}
{3,63,3.88}
{2.02,5.21}
{2.14,5.45}
{1.99,6.19}
{3.54,7.13}
{7.60,9.00}
{7.16,9.39}
f2 (ppm)
f1 (ppm)

HSQC

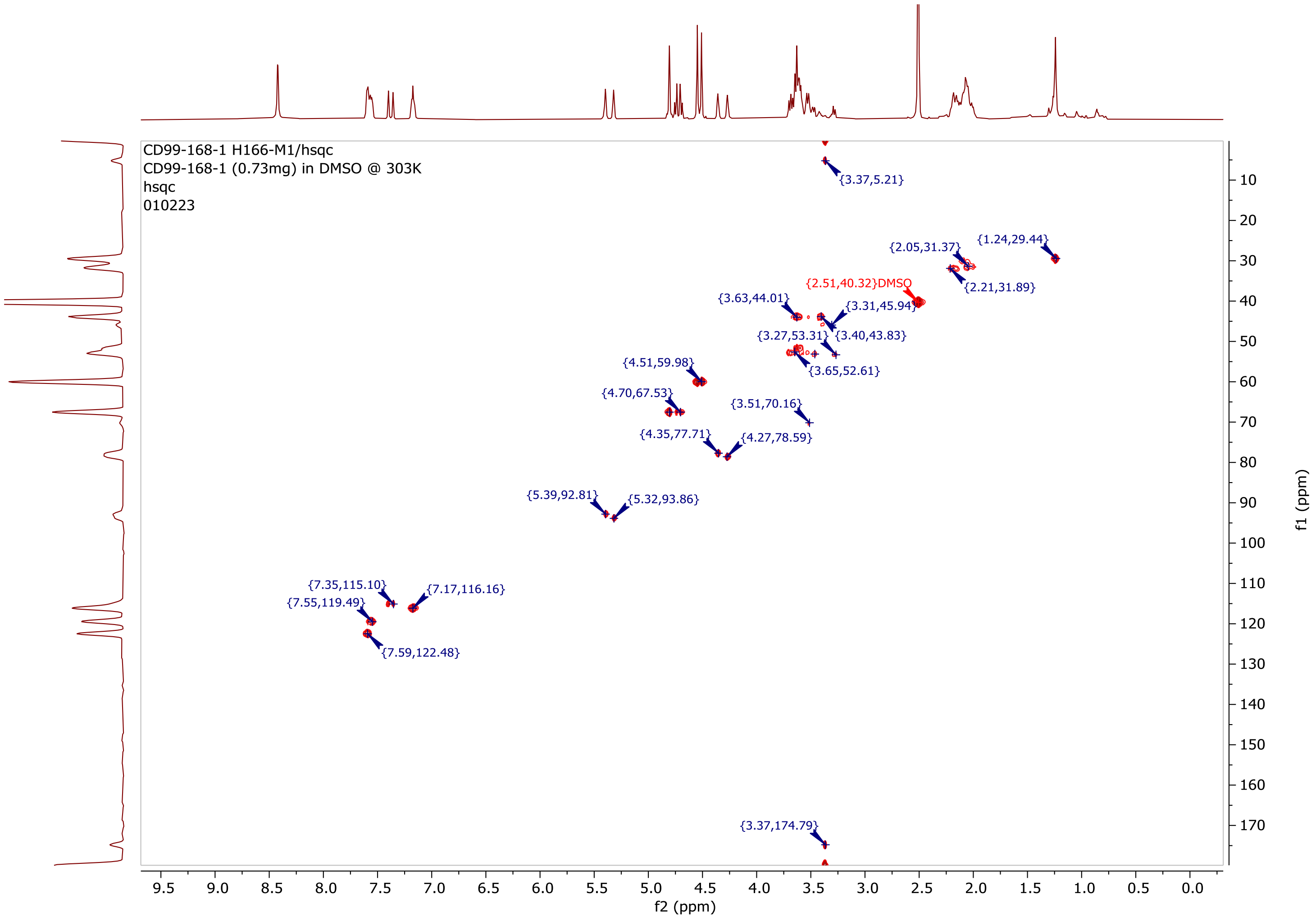
CD99-168-1 H166-M1/hsqc
CD99-168-1 (0.73mg) in DMSO @ 303K
hsqc
010223
{3.37,5.21}
{1.24,29.44}
{2.05,31.37}
{2.21,31.89}
{2.51,40.32}DMSO
{3.63,44.01}
{3.31,45.94}
{3.27,53.31}
{3.40,43.83}
{3.65,52.61}
{4.51,59.98}
{4.70,67.53}
{3.51,70.16}
{4.35,77.71}
{4.27,78.59}
{5.39,92.81}
{5.32,93.86}
{7.35,115.10}
{7.17,116.16}
{7.55,119.49}
{7.59,122.48}
{3.37,174.79}
f2 (ppm)
f1 (ppm)

HMBC

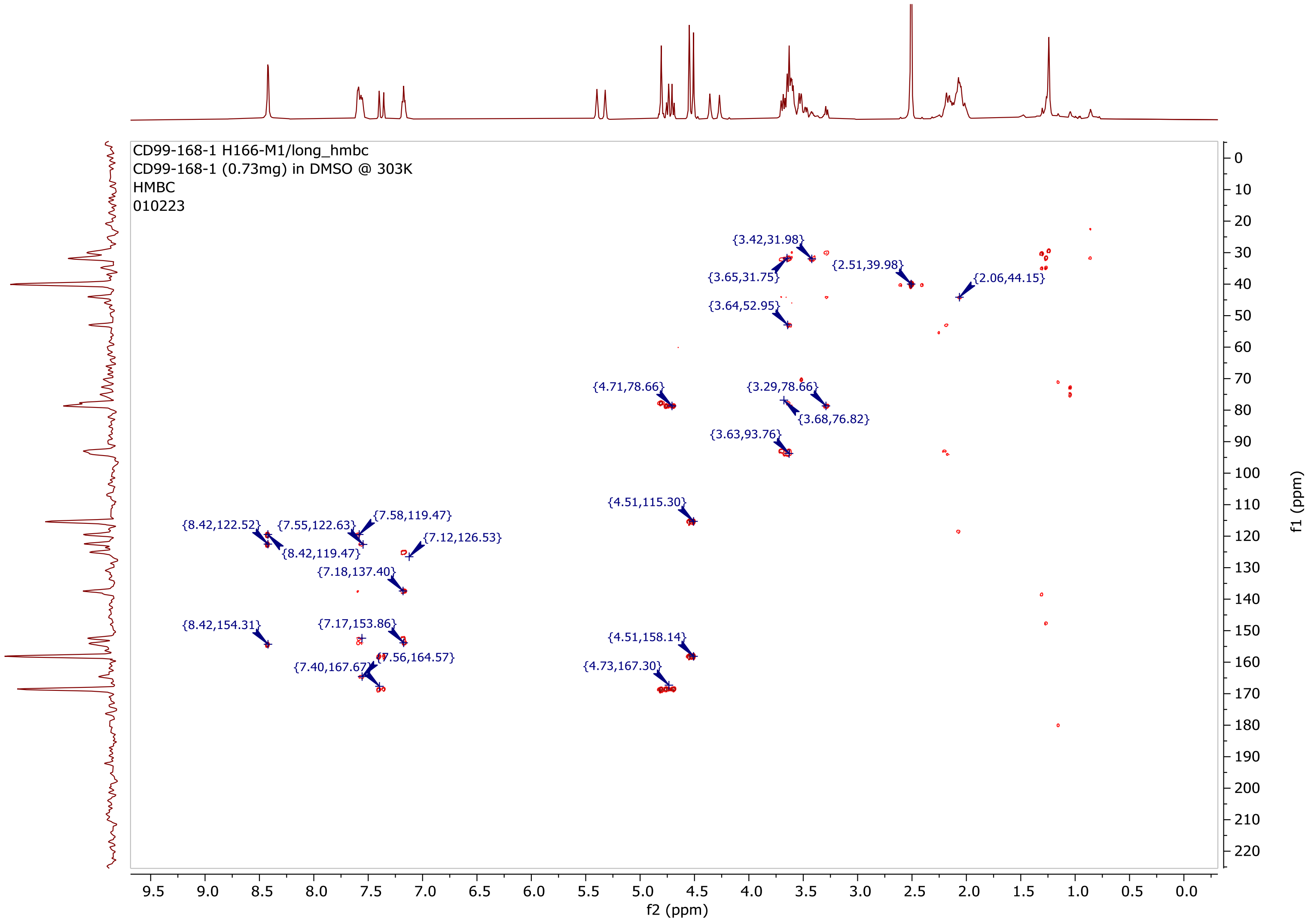

CD99-168-1 H166-M1/long_hmbc
CD99-168-1 (0.73mg) in DMSO @ 303K
HMBC
010223
{3.42,31.98}
{3.65,31.75}
{2.51,39.98}
{2.06,44.15}
{3.64,52.95}
{4.71,78.66}
{3.29,78.66}
{3.68,76.82}
{3.63,93.76}
{4.51,115.30}
{8.42,122.52}
{7.55,122.63}
{7.58,119.47}
{8.42,119.47}
{7.12,126.53}
{7.18,137.40}
{8.42,154.31}
{7.17,153.86}
{4.51,158.14}
{7.40,167.67}
{7.56,164.57}
{4.73,167.30}
f2 (ppm)
f1 (ppm)

HRMS

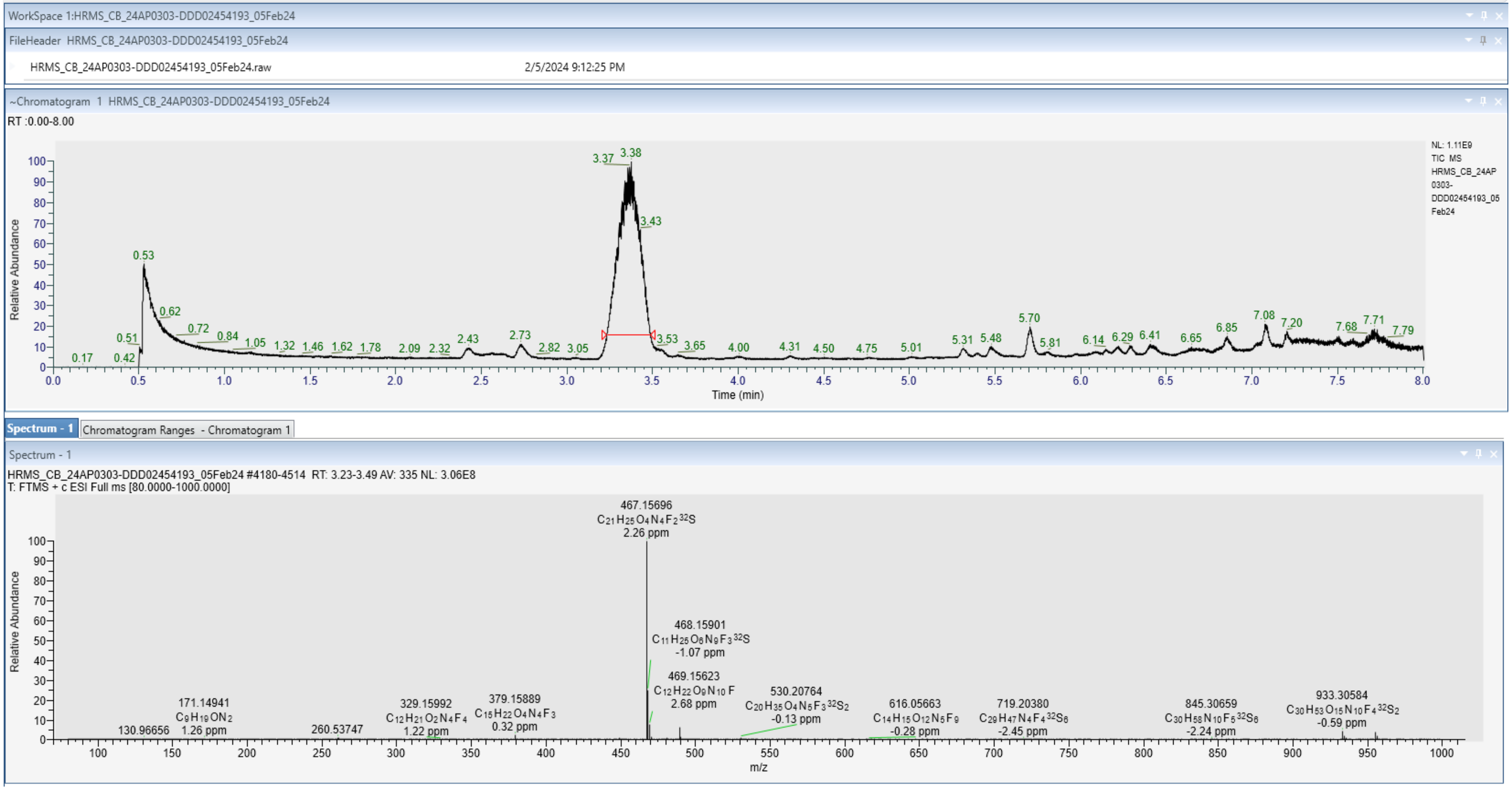
WorkSpace 1:HRMS_CB_24AP0303-DDD02454193_05Feb24
FileHeader HRMS_CB_24AP0303-DDD02454193_05Feb24
HRMS_CB_24AP0303-DDD02454193_05Feb24.raw
2/5/2024 9:12:25 PM
~Chromatogram 1 HRMS_CB_24AP0303-DDD02454193_05Feb24
RT :0.00-8.00
NL: 1.11E9
TIC MS
HRMS_CB_24AP0303-DDD02454193_05Feb24
Relative Abundance
Time (min)
Spectrum - 1
Chromatogram Ranges - Chromatogram 1
HRMS_CB_24AP0303-DDD02454193_05Feb24 #4180-4514 RT: 3.23-3.49 AV: 335 NL: 3.06E8
T: FTMS + c ESI Full ms [80.0000-1000.0000]
467.15696
$C_{21}H_{25}O_4N_4F_2{}^{32}S$
2.26 ppm
468.15901
$C_{11}H_{25}O_8N_9F_3{}^{32}S$
-1.07 ppm
469.15623
$C_{12}H_{22}O_9N_{10}F$
2.68 ppm
171.14941
$C_9H_{19}ON_2$
1.26 ppm
130.96656
260.53747
329.15992
$C_{12}H_{21}O_2N_4F_4$
1.22 ppm
379.15889
$C_{15}H_{22}O_4N_4F_3$
0.32 ppm
530.20764
$C_{20}H_{35}O_4N_5F_3{}^{32}S_2$
-0.13 ppm
616.05663
$C_{14}H_{15}O_{12}N_5F_9$
-0.28 ppm
719.20380
$C_{29}H_{47}N_4F_4{}^{32}S_8$
-2.45 ppm
845.30659
$C_{30}H_{58}N_{10}F_5{}^{32}S_8$
-2.24 ppm
933.30584
$C_{30}H_{53}O_{15}N_{10}F_4{}^{32}S_2$
-0.59 ppm
m/z

Compound **9a**

^{1}H NMR

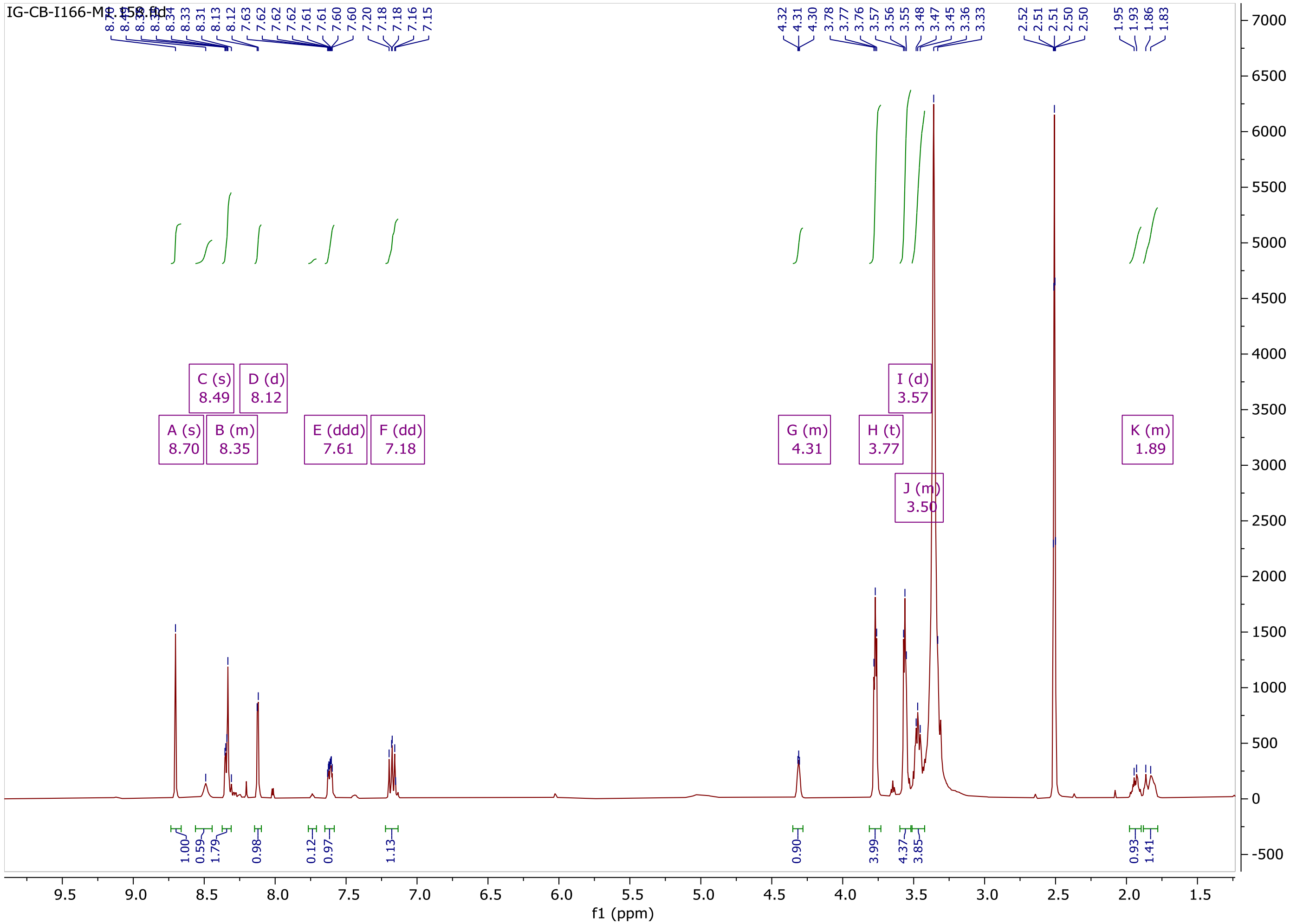

COSY

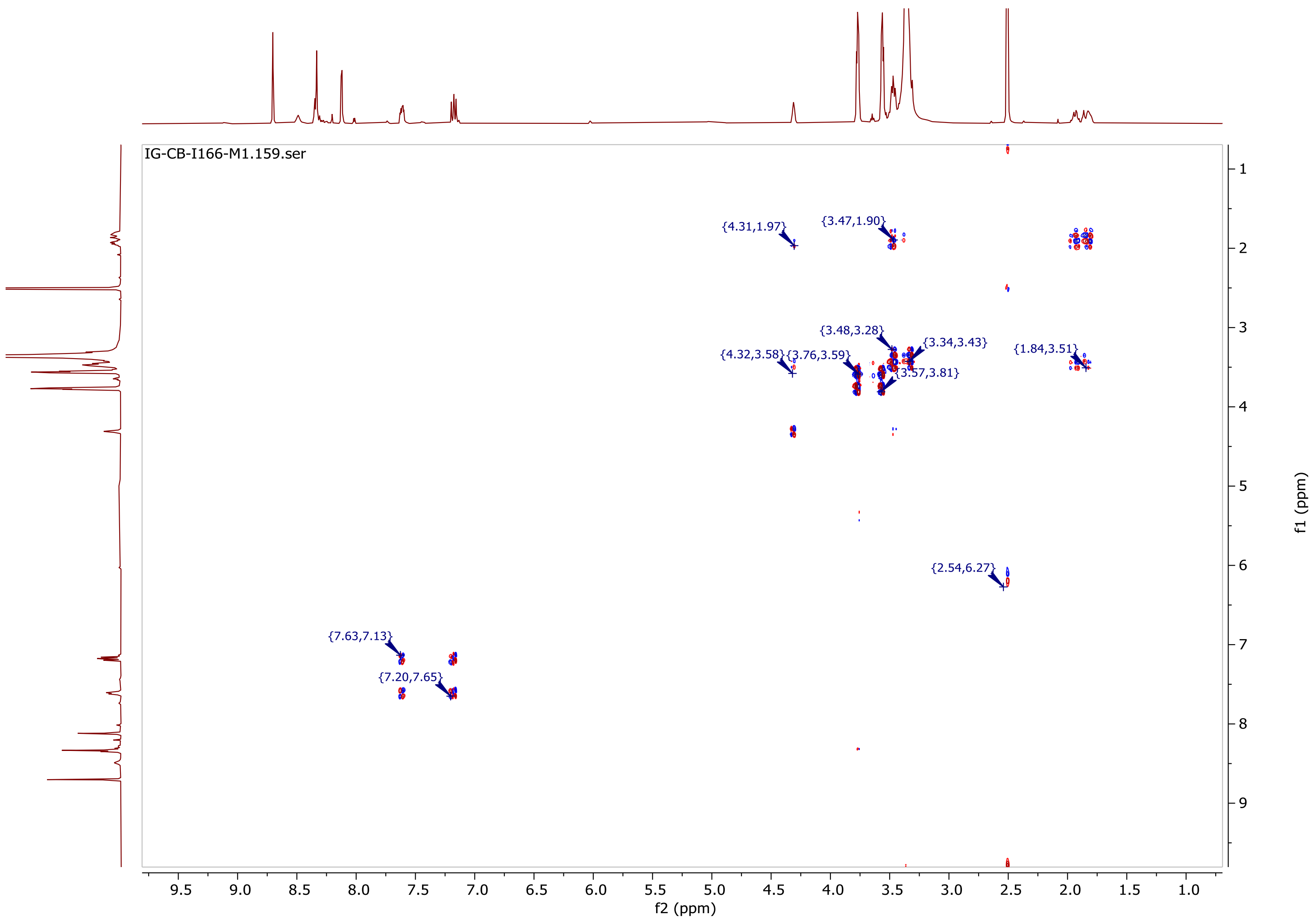
IG-CB-I166-M1.159.ser
{4.31,1.97}
{3.47,1.90}
{3.48,3.28}
{3.34,3.43}
{1.84,3.51}
{4.32,3.58}
{3.76,3.59}
{3.57,3.81}
{2.54,6.27}
{7.63,7.13}
{7.20,7.65}
f2 (ppm)
f1 (ppm)

HSQC

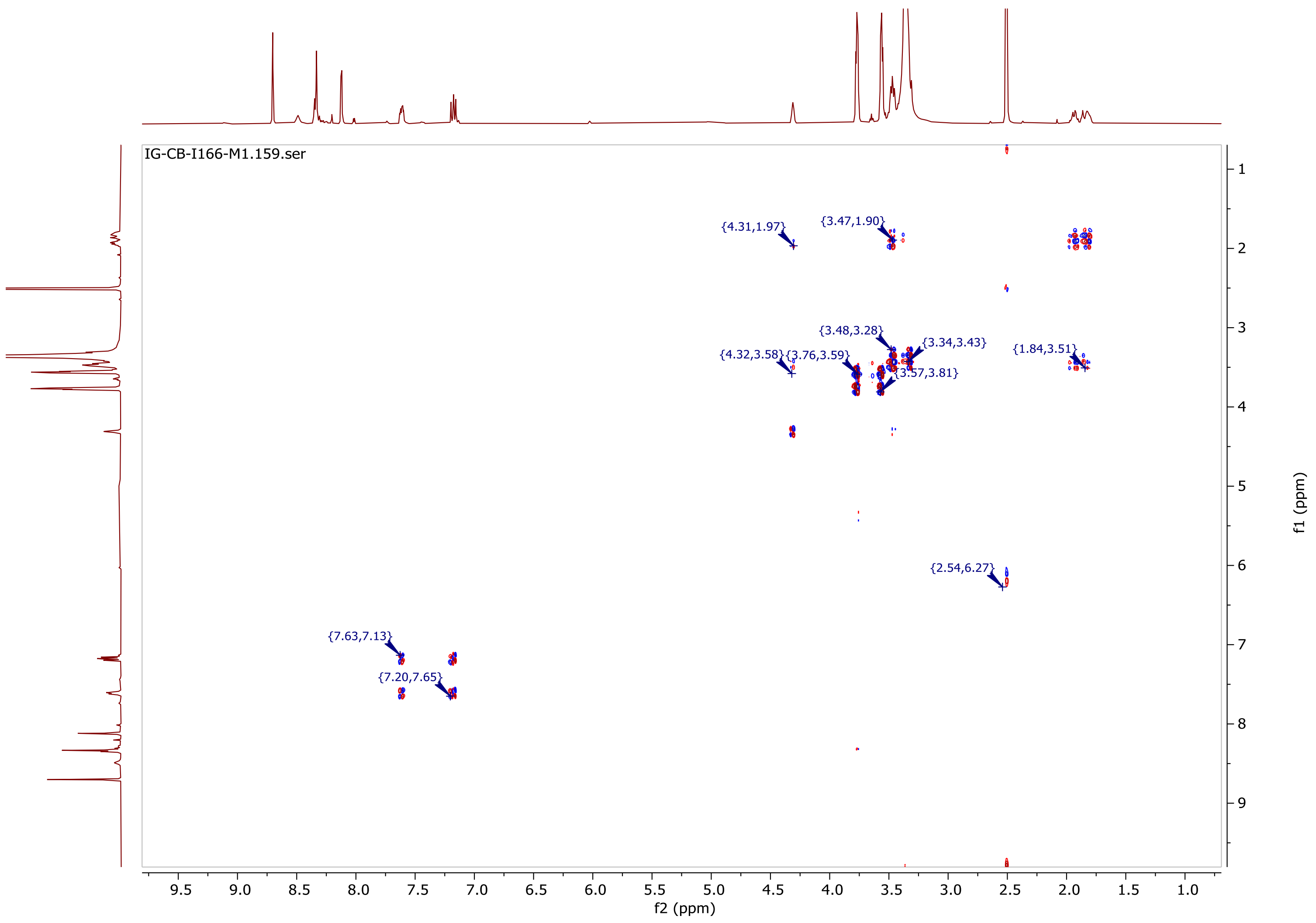

IG-CB-I166-M1.159.ser
{4.31,1.97}
{3.47,1.90}
{3.48,3.28}
{3.34,3.43}
{1.84,3.51}
{4.32,3.58}
{3.76,3.59}
{3.57,3.81}
{2.54,6.27}
{7.63,7.13}
{7.20,7.65}
f2 (ppm)
f1 (ppm)

HMBC

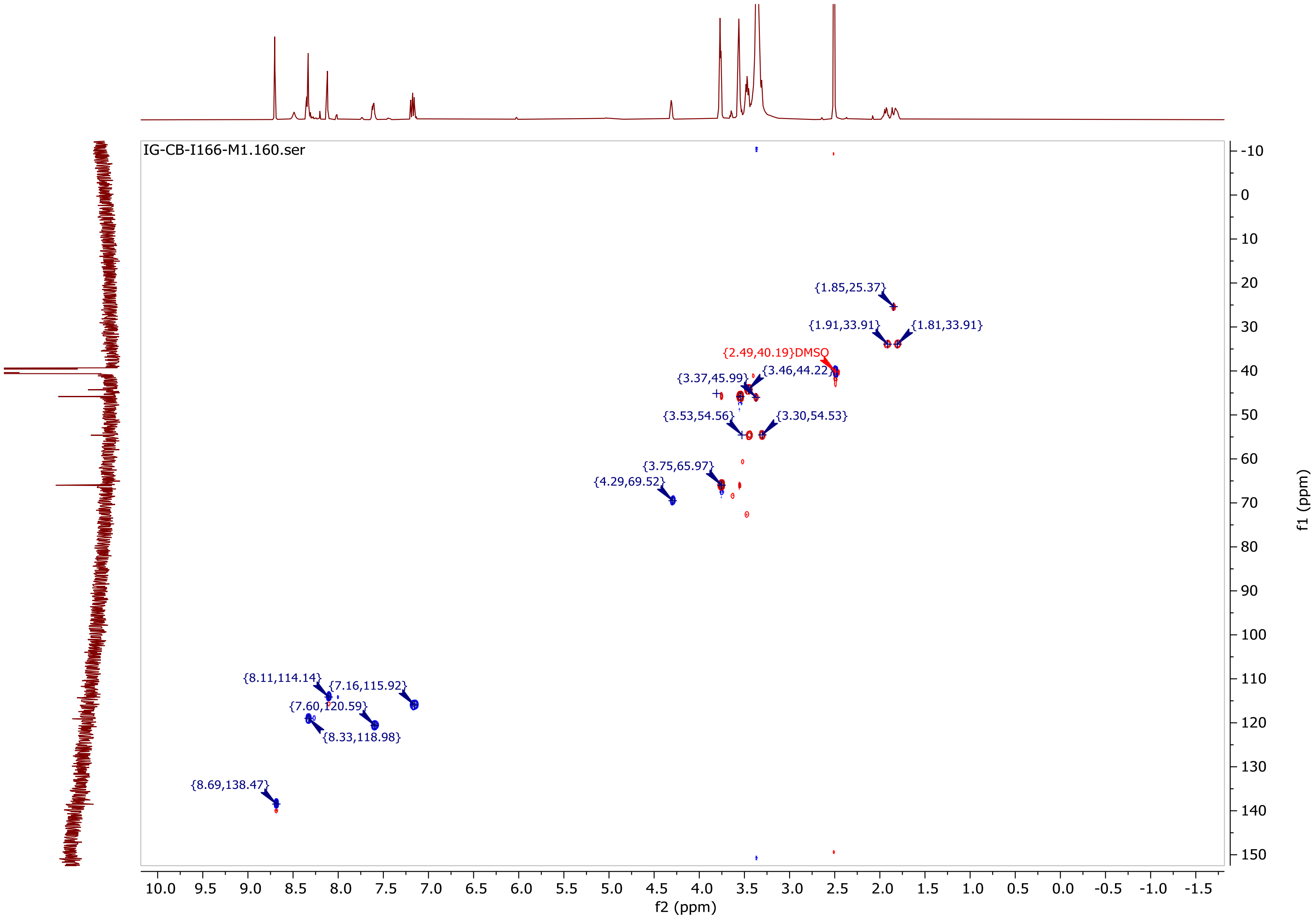

IG-CB-I166-M1.160.ser
{1.85,25.37}
{1.91,33.91}
{1.81,33.91}
{2.49,40.19}DMSO
{3.37,45.99}
{3.46,44.22}
{3.53,54.56}
{3.30,54.53}
{3.75,65.97}
{4.29,69.52}
{8.11,114.14}
{7.16,115.92}
{7.60,120.59}
{8.33,118.98}
{8.69,138.47}
f2 (ppm)
f1 (ppm)

NOESY

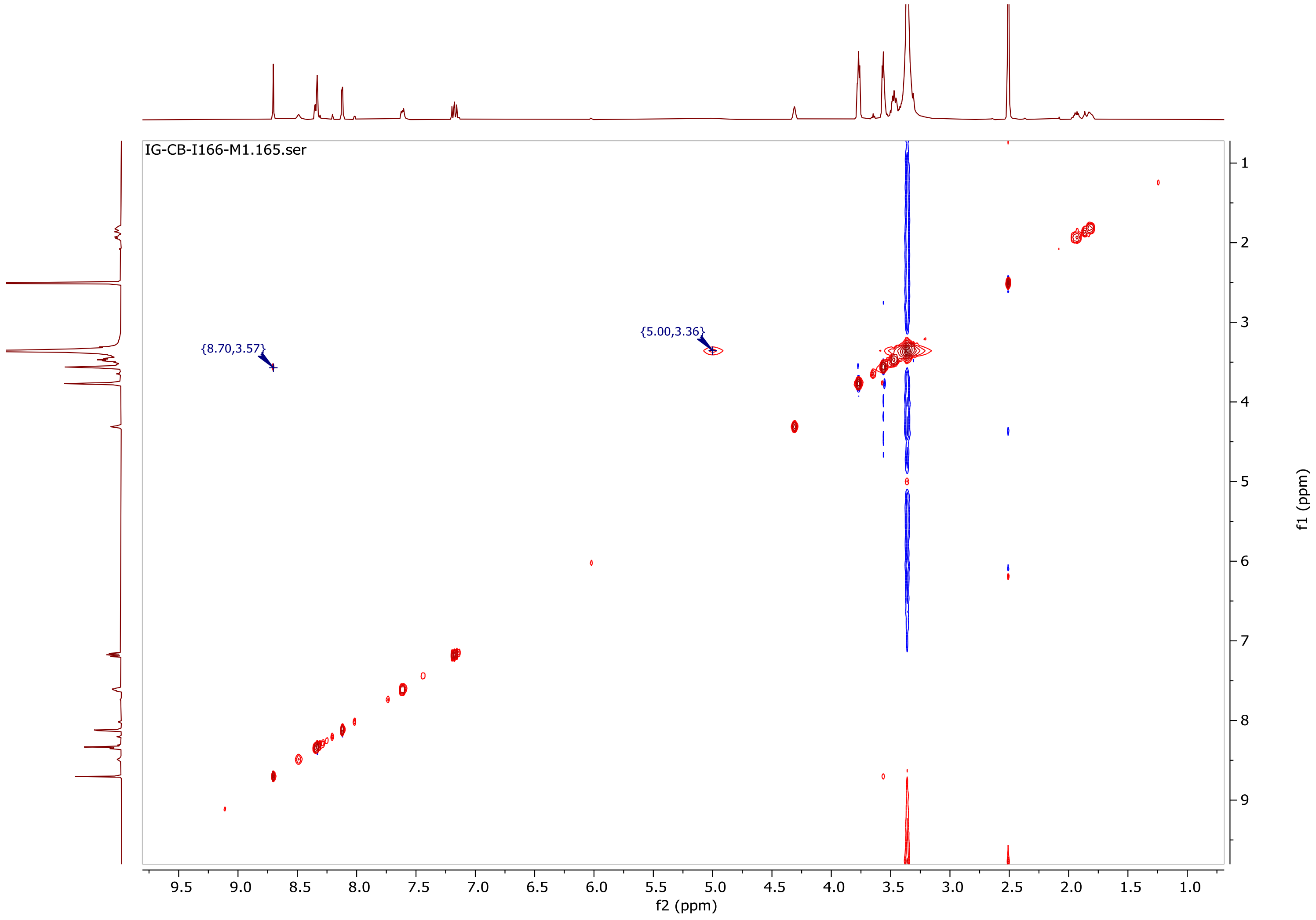

IG-CB-I166-M1.165.ser
{8.70,3.57}
{5.00,3.36}
9.5
9.0
8.5
8.0
7.5
7.0
6.5
6.0
5.5
5.0
4.5
4.0
3.5
3.0
2.5
2.0
1.5
1.0
f2 (ppm)
1
2
3
4
5
6
7
8
9
f1 (ppm)

DEPTqgpsp

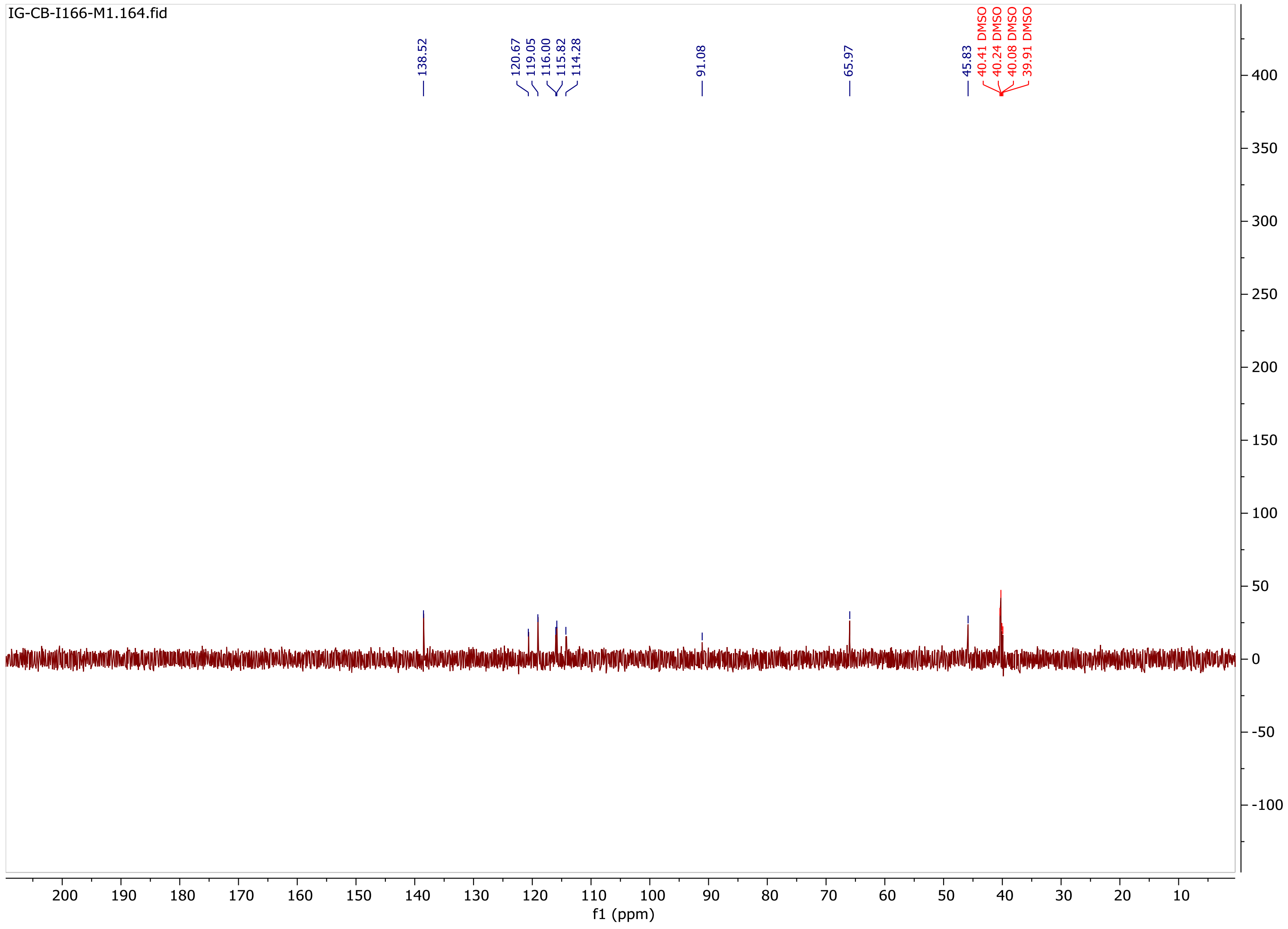

IG-CB-I166-M1.164.fid
138.52
120.67
119.05
116.00
115.82
114.28
91.08
65.97
45.83
40.41 DMSO
40.24 DMSO
40.08 DMSO
39.91 DMSO
f1 (ppm)

Zgpg30

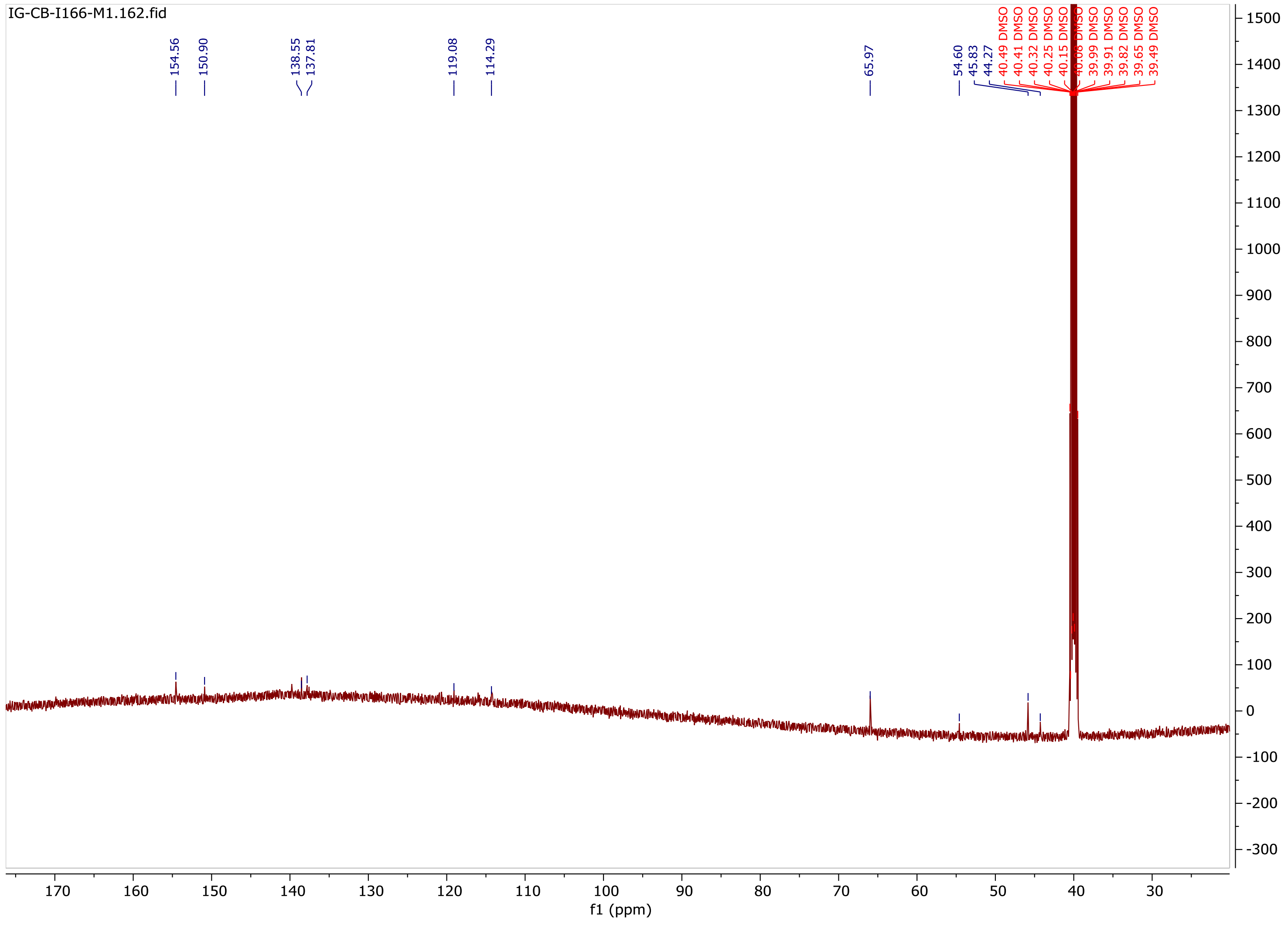
IG-CB-I166-M1.162.fid
154.56
150.90
138.55
137.81
119.08
114.29
65.97
54.60
45.83
44.27
40.49 DMSO
40.41 DMSO
40.32 DMSO
40.25 DMSO
40.15 DMSO
40.08 DMSO
39.99 DMSO
39.91 DMSO
39.82 DMSO
39.65 DMSO
39.49 DMSO
f1 (ppm)

DEPT-135

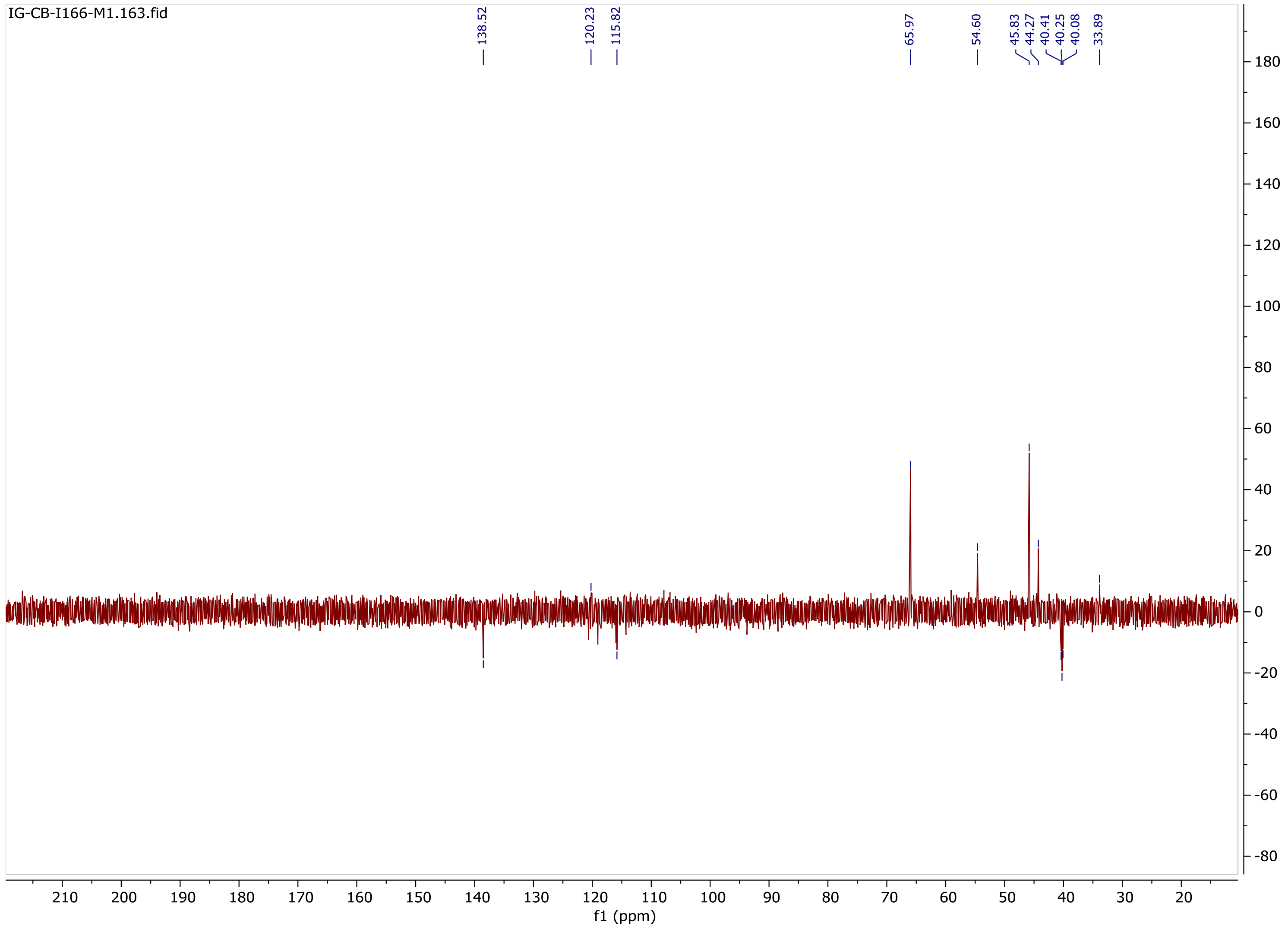
IG-CB-I166-M1.163.fid
138.52
120.23
115.82
65.97
54.60
45.83
44.27
40.41
40.25
40.08
33.89
f1 (ppm)

HRMS

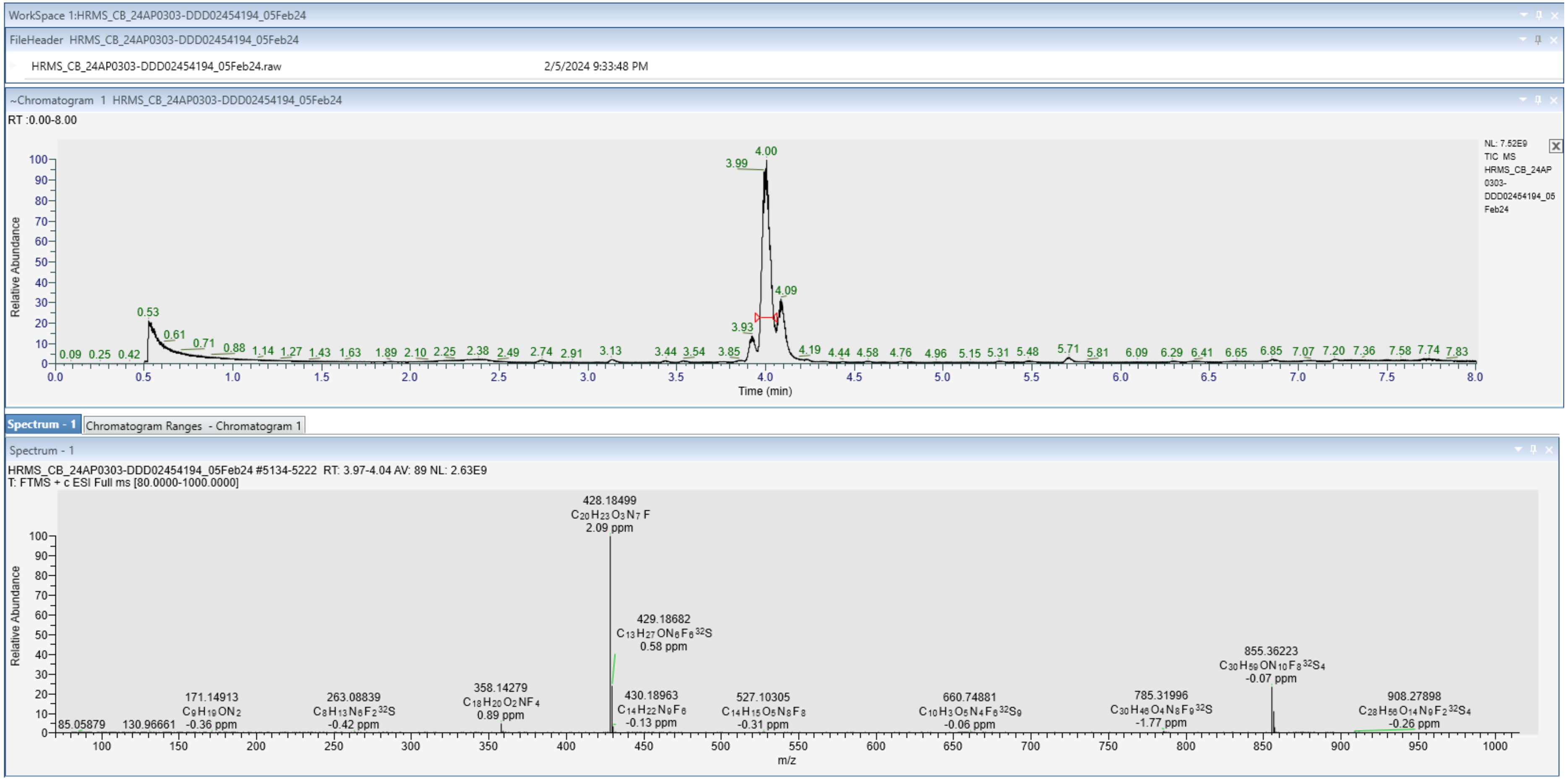

WorkSpace 1:HRMS_CB_24AP0303-DDD02454194_05Feb24
FileHeader HRMS_CB_24AP0303-DDD02454194_05Feb24
HRMS_CB_24AP0303-DDD02454194_05Feb24.raw
2/5/2024 9:33:48 PM
~Chromatogram 1 HRMS_CB_24AP0303-DDD02454194_05Feb24
RT :0.00-8.00
NL: 7.52E9
TIC MS
HRMS_CB_24AP0303-DDD02454194_05Feb24
Relative Abundance
Time (min)
Spectrum - 1
Chromatogram Ranges - Chromatogram 1
HRMS_CB_24AP0303-DDD02454194_05Feb24 #5134-5222 RT: 3.97-4.04 AV: 89 NL: 2.63E9
T: FTMS + c ESI Full ms [80.0000-1000.0000]
428.18499
C20 H23 O3 N7 F
2.09 ppm
429.18682
C13 H27 O N6 F8 32S
0.58 ppm
855.36223
C30 H59 O N10 F8 32S4
-0.07 ppm
m/z

Compound **9b**

^{1}H NMR

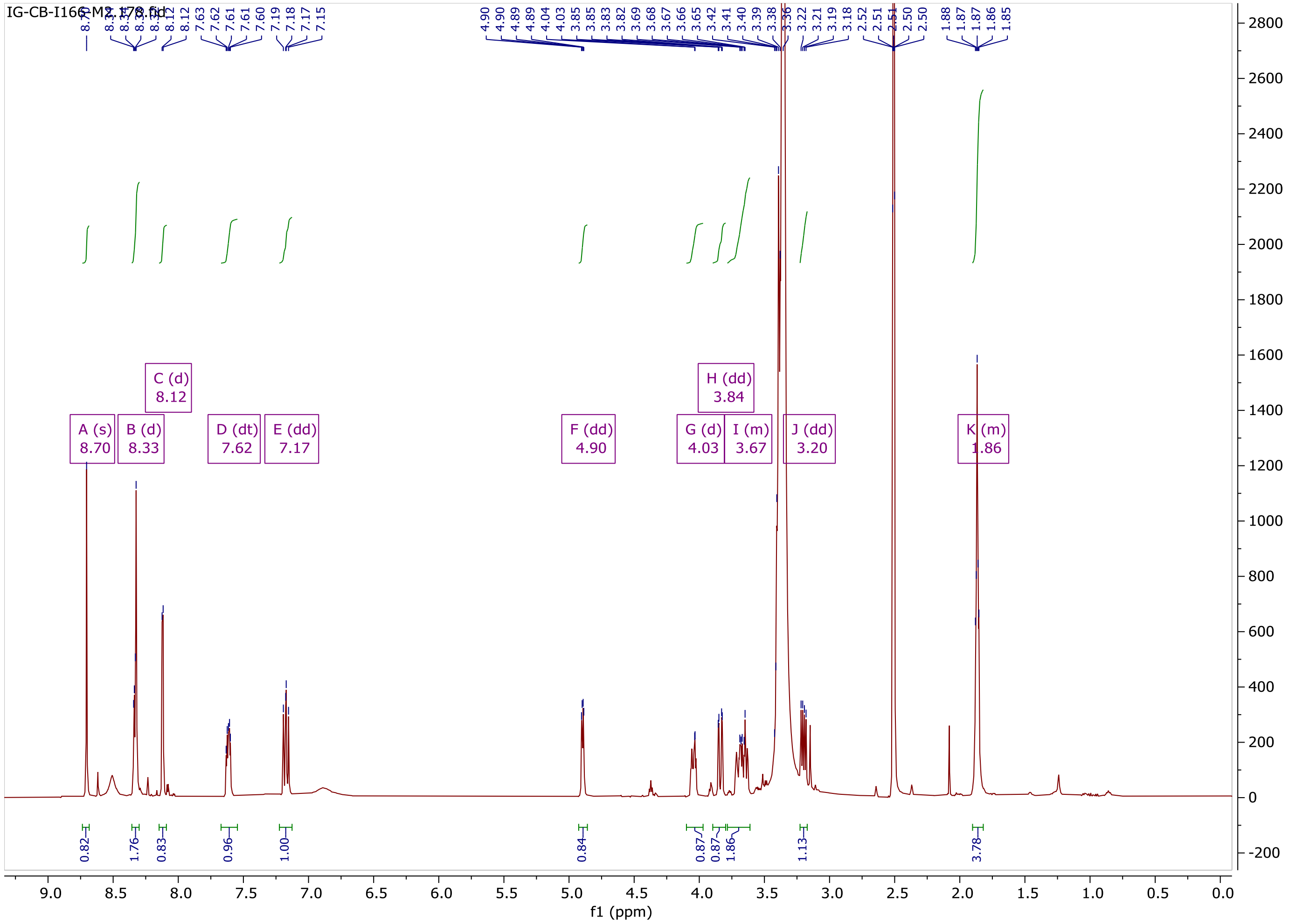

COSY

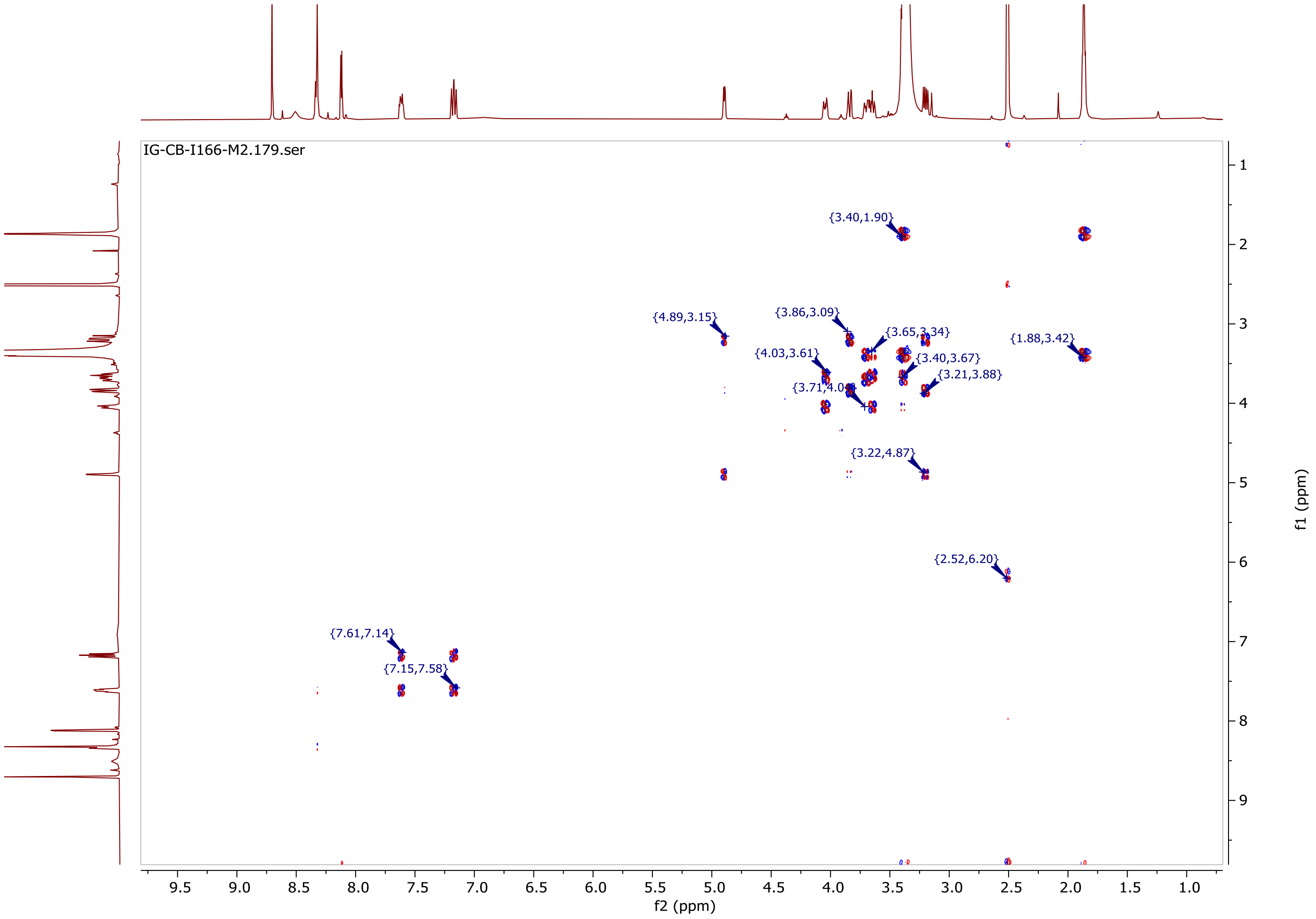
IG-CB-I166-M2.179.ser
{3.40,1.90}
{4.89,3.15}
{3.86,3.09}
{3.65,3.34}
{1.88,3.42}
{4.03,3.61}
{3.40,3.67}
{3.21,3.88}
{3.71,4.04}
{3.22,4.87}
{2.52,6.20}
{7.61,7.14}
{7.15,7.58}
f2 (ppm)
f1 (ppm)

HSQC

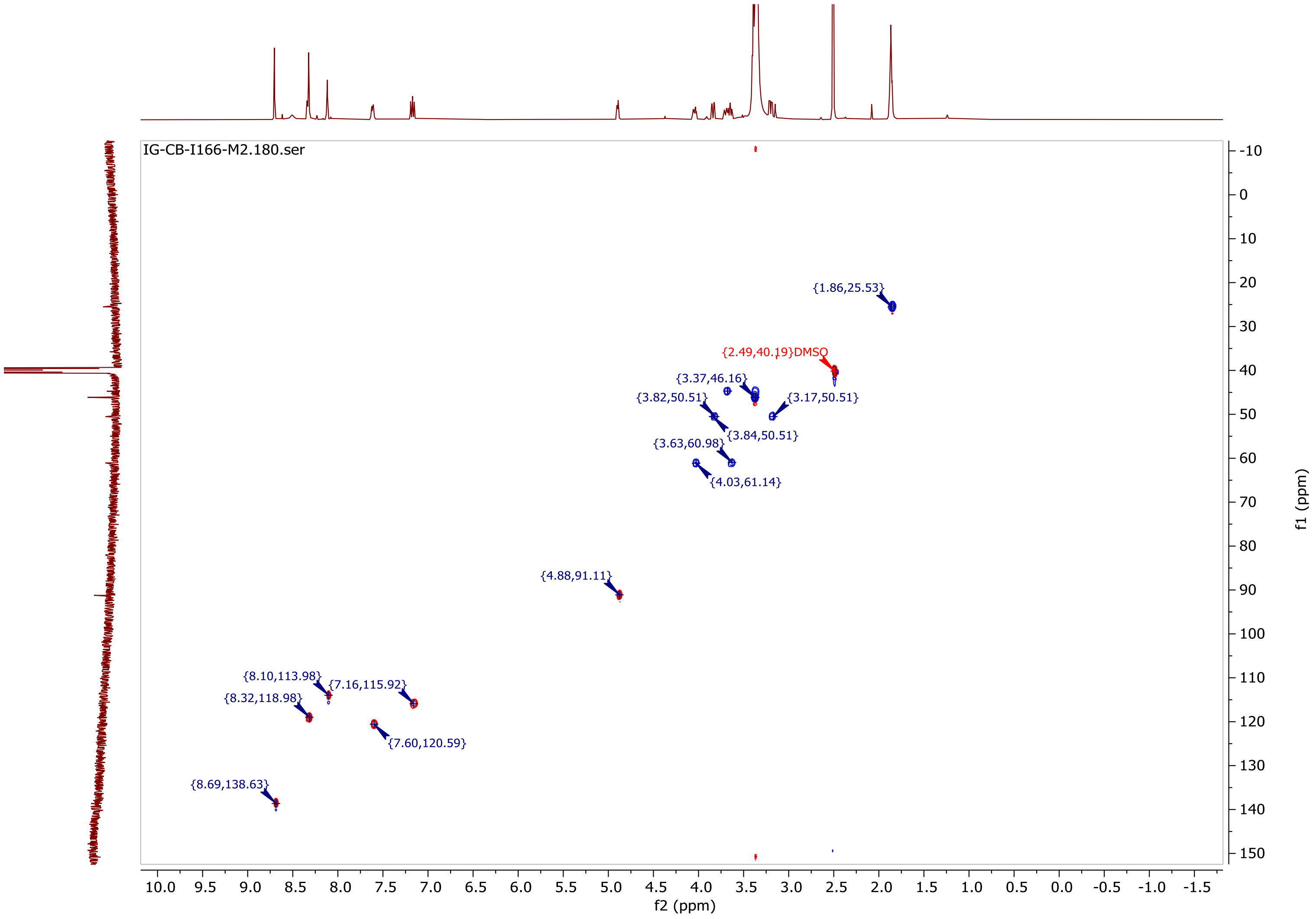
IG-CB-I166-M2.180.ser
{1.86,25.53}
{2.49,40.19}DMSO
{3.37,46.16}
{3.82,50.51}
{3.17,50.51}
{3.84,50.51}
{3.63,60.98}
{4.03,61.14}
{4.88,91.11}
{8.10,113.98}
{7.16,115.92}
{8.32,118.98}
{7.60,120.59}
{8.69,138.63}
f2 (ppm)
f1 (ppm)

HMBC

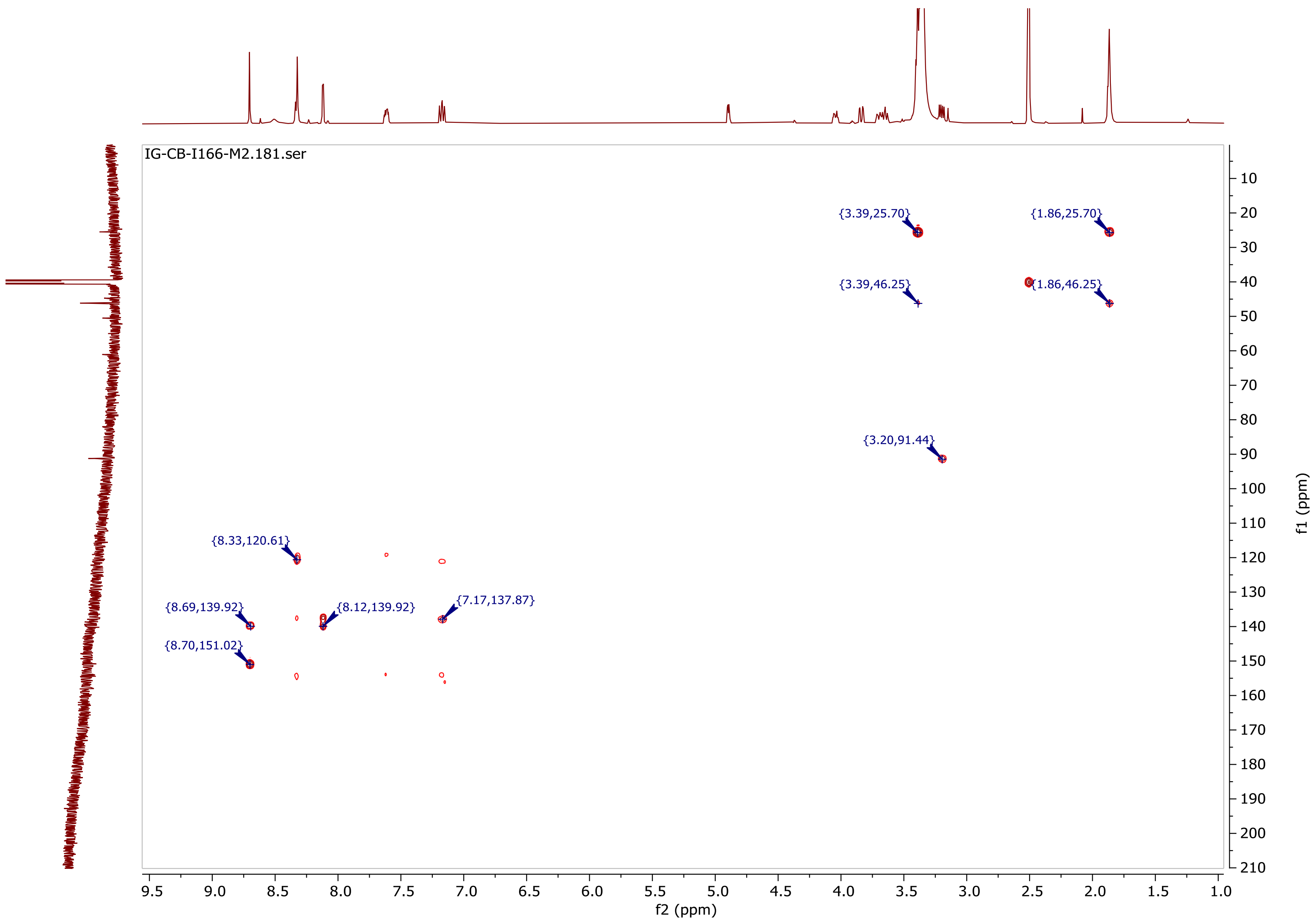

IG-CB-I166-M2.181.ser
{3.39,25.70}
{1.86,25.70}
{3.39,46.25}
{1.86,46.25}
{3.20,91.44}
{8.33,120.61}
{8.69,139.92}
{8.12,139.92}
{7.17,137.87}
{8.70,151.02}
f2 (ppm)
f1 (ppm)

NOESY

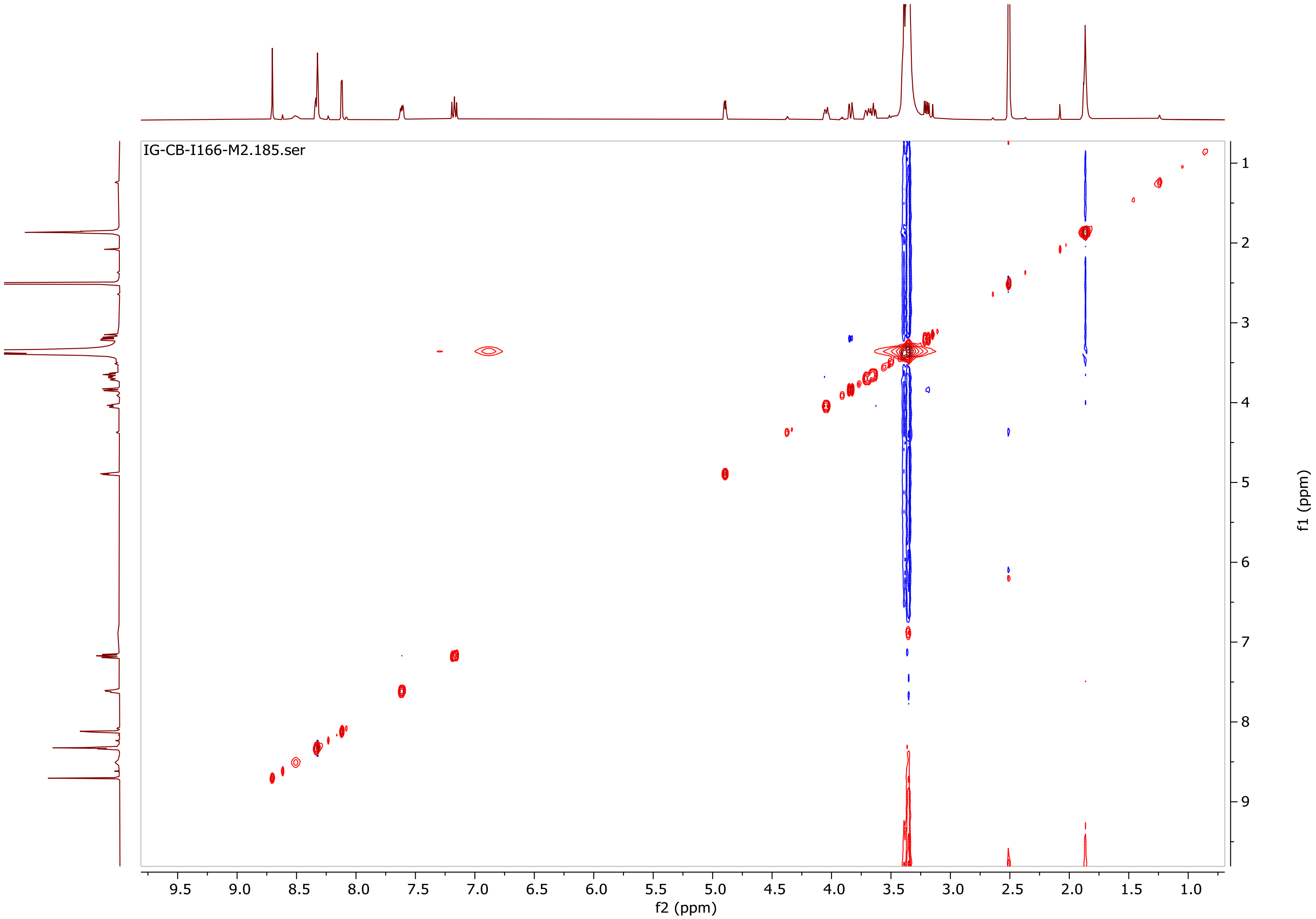

IG-CB-I166-M2.185.ser
f2 (ppm)
f1 (ppm)
9.5 9.0 8.5 8.0 7.5 7.0 6.5 6.0 5.5 5.0 4.5 4.0 3.5 3.0 2.5 2.0 1.5 1.0
1 2 3 4 5 6 7 8 9

DEPTqgpsp

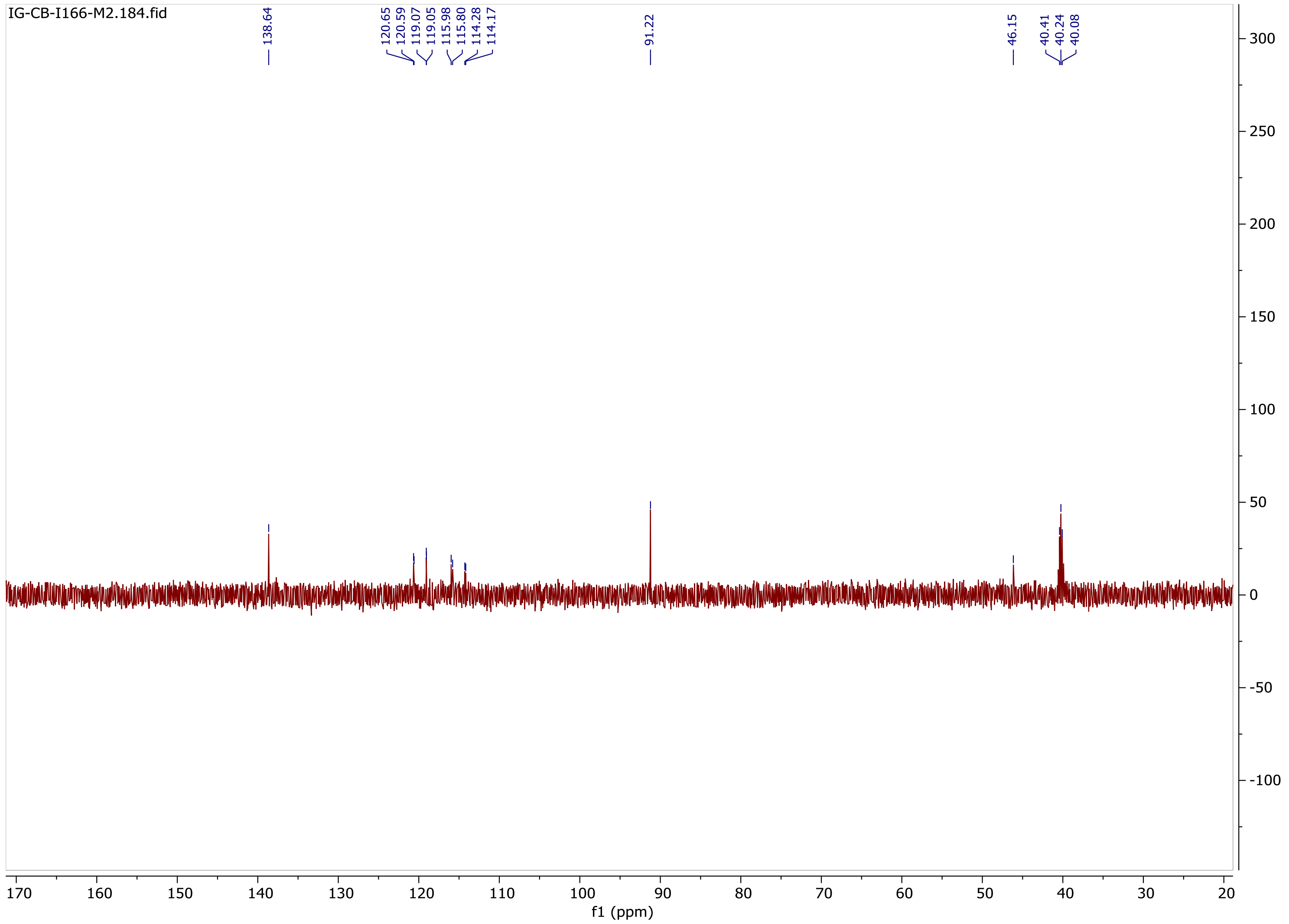

IG-CB-I166-M2.184.fid
138.64
120.65
120.59
119.07
119.05
115.98
115.80
114.28
114.17
91.22
46.15
40.41
40.24
40.08
300
250
200
150
100
50
0
-50
-100
170
160
150
140
130
120
110
100
90
80
70
60
50
40
30
20
f1 (ppm)

Zgpg30

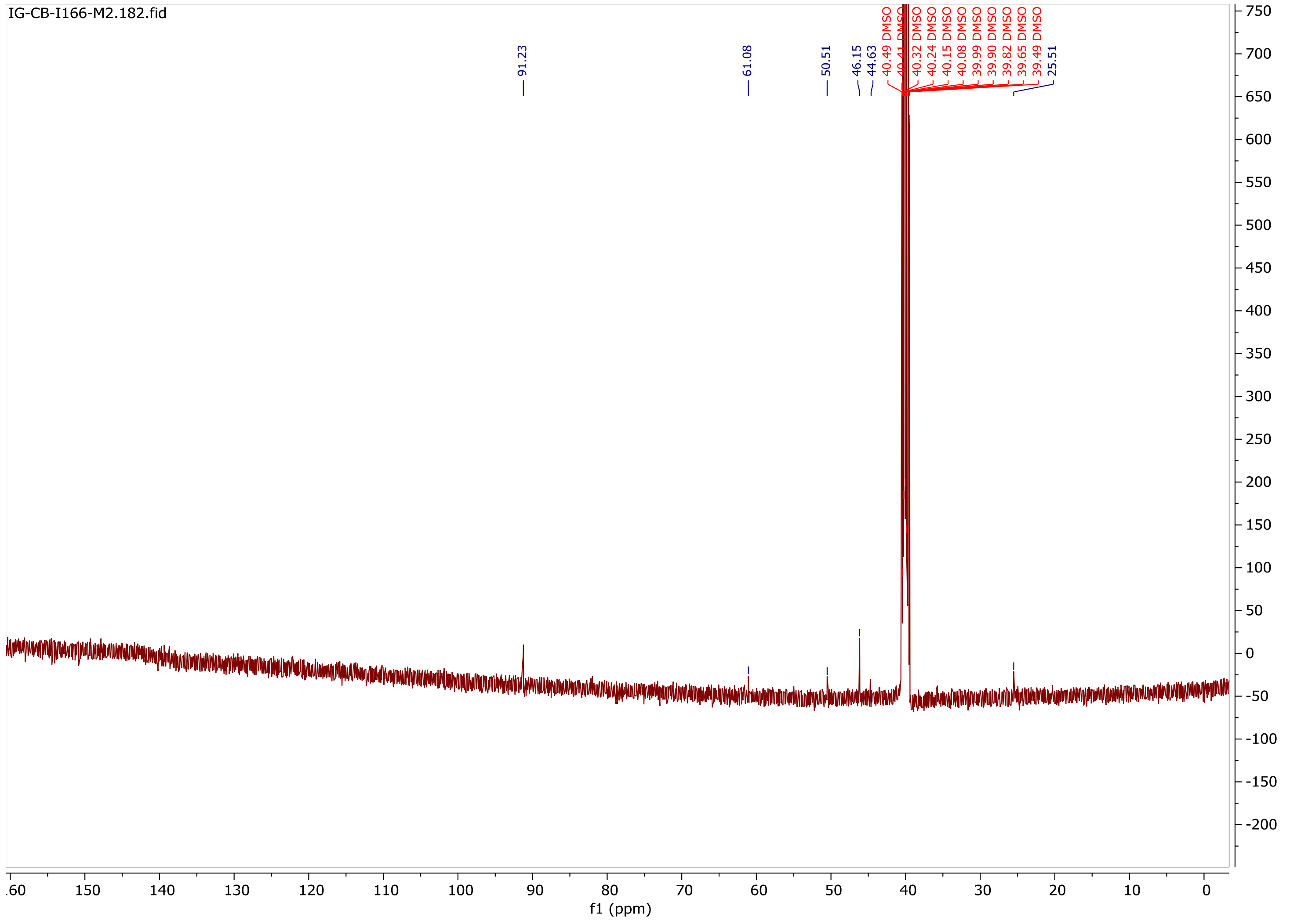
IG-CB-I166-M2.182.fid
91.23
61.08
50.51
46.15
44.63
40.49 DMSO
40.41 DMSO
40.32 DMSO
40.24 DMSO
40.15 DMSO
40.08 DMSO
39.99 DMSO
39.90 DMSO
39.82 DMSO
39.65 DMSO
39.49 DMSO
25.51
f1 (ppm)

DEPT-135

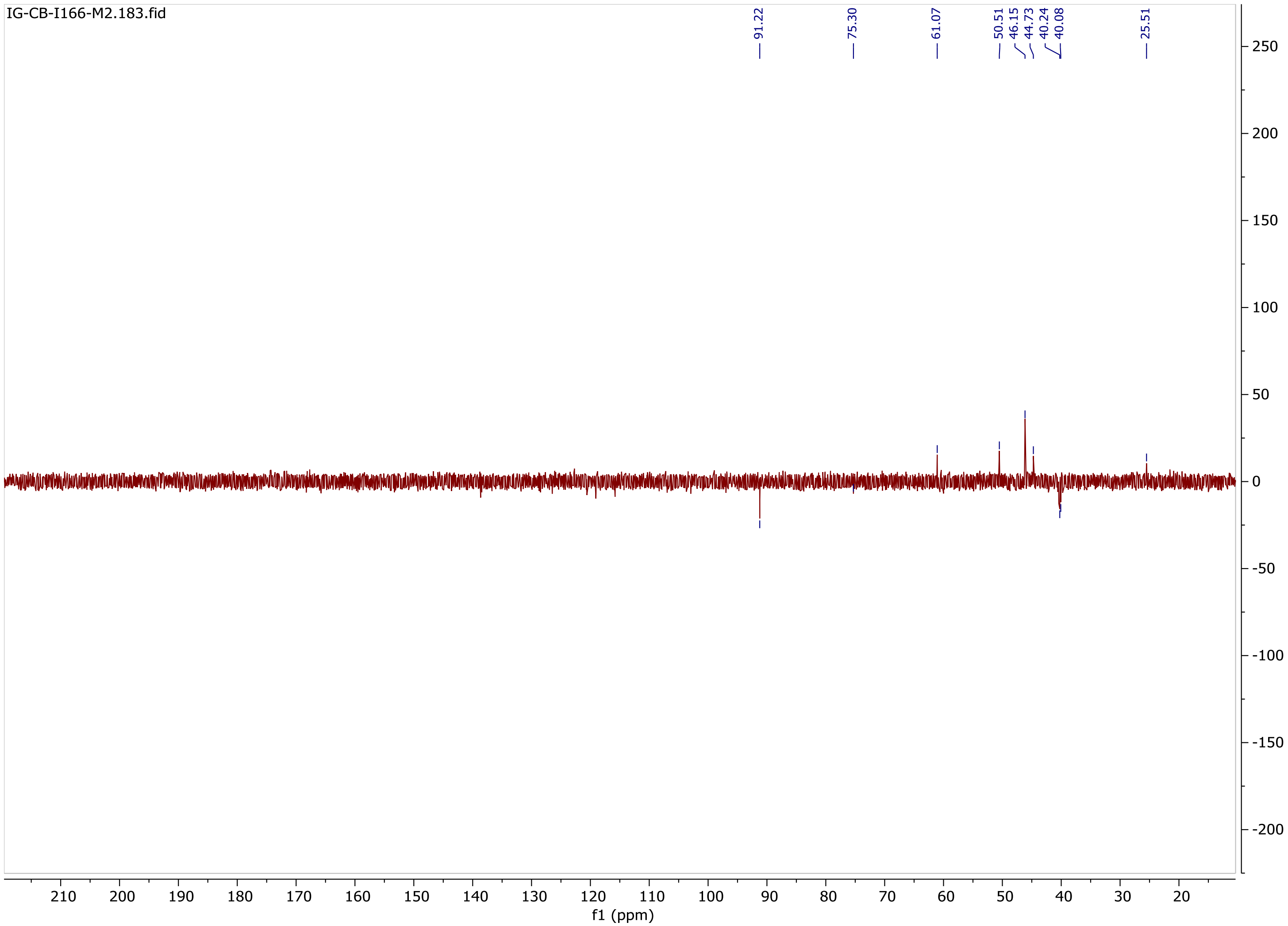
IG-CB-I166-M2.183.fid
91.22
75.30
61.07
50.51
46.15
44.73
40.24
40.08
25.51
250
200
150
100
50
0
-50
-100
-150
-200
210 200 190 180 170 160 150 140 130 120 110 100 90 80 70 60 50 40 30 20
f1 (ppm)

HRMS

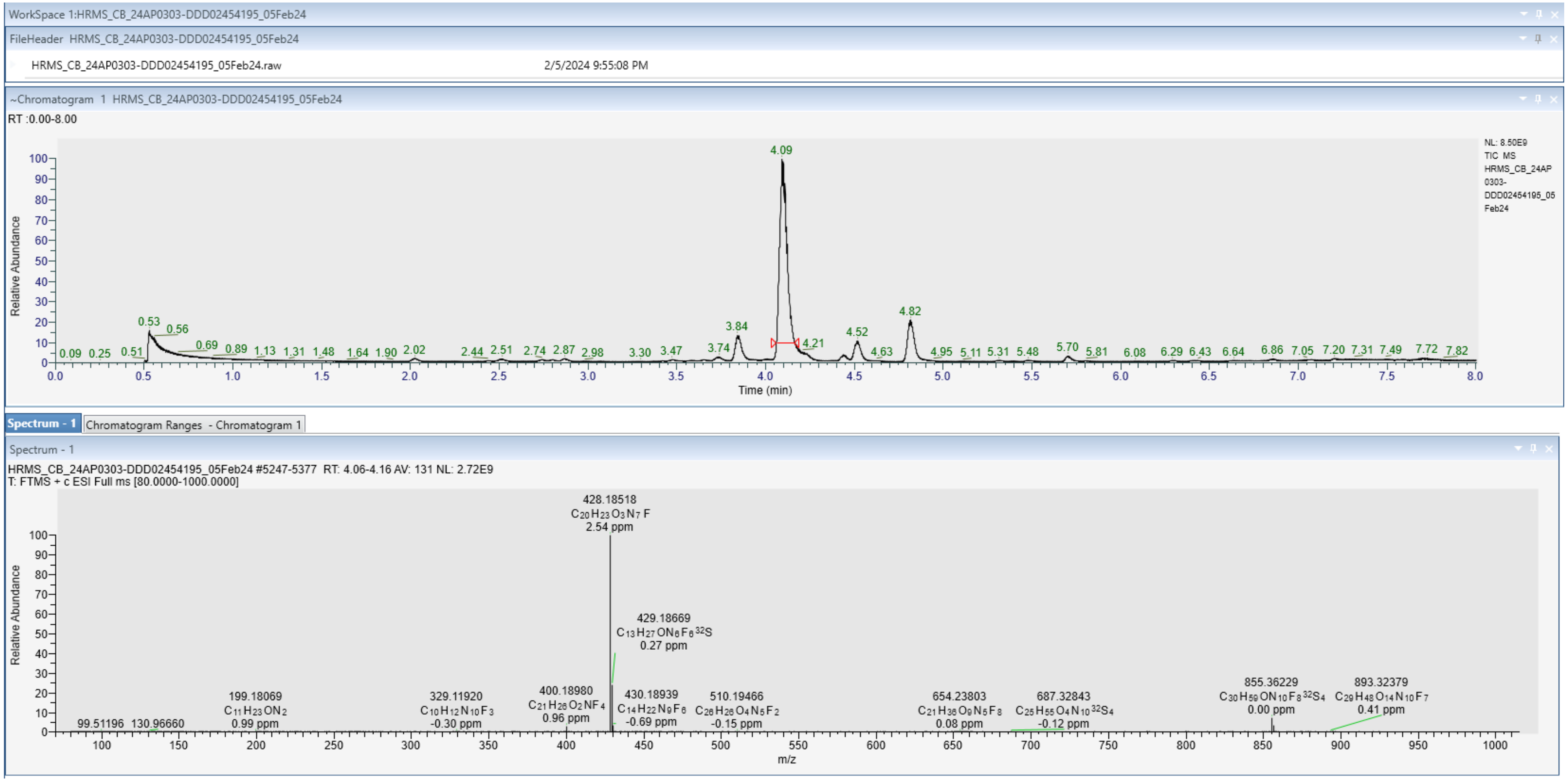

WorkSpace 1:HRMS_CB_24AP0303-DDD02454195_05Feb24
FileHeader HRMS_CB_24AP0303-DDD02454195_05Feb24
HRMS_CB_24AP0303-DDD02454195_05Feb24.raw
2/5/2024 9:55:08 PM
~Chromatogram 1 HRMS_CB_24AP0303-DDD02454195_05Feb24
RT :0.00-8.00
NL: 8.50E9
TIC MS
HRMS_CB_24AP0303-DDD02454195_05Feb24
Relative Abundance
Time (min)
4.09
4.82
4.52
3.84
0.53
Spectrum - 1
Chromatogram Ranges - Chromatogram 1
HRMS_CB_24AP0303-DDD02454195_05Feb24 #5247-5377 RT: 4.06-4.16 AV: 131 NL: 2.72E9
T: FTMS + c ESI Full ms [80.0000-1000.0000]
428.18518
C20 H23 O3 N7 F
2.54 ppm
429.18669
C13 H27 O N6 F6 32S
0.27 ppm
400.18980
C21 H26 O2 N F4
0.96 ppm
430.18939
C14 H22 N9 F6
-0.69 ppm
510.19466
C26 H26 O4 N5 F2
-0.15 ppm
199.18069
C11 H23 O N2
0.99 ppm
329.11920
C10 H12 N10 F3
-0.30 ppm
654.23803
C21 H36 O9 N5 F8
0.08 ppm
687.32843
C25 H55 O4 N10 32S4
-0.12 ppm
855.36229
C30 H59 O N10 F8 32S4
0.00 ppm
893.32379
C29 H48 O14 N10 F7
0.41 ppm
99.51196
130.96660
m/z

Compound **9c**

^{1}H NMR

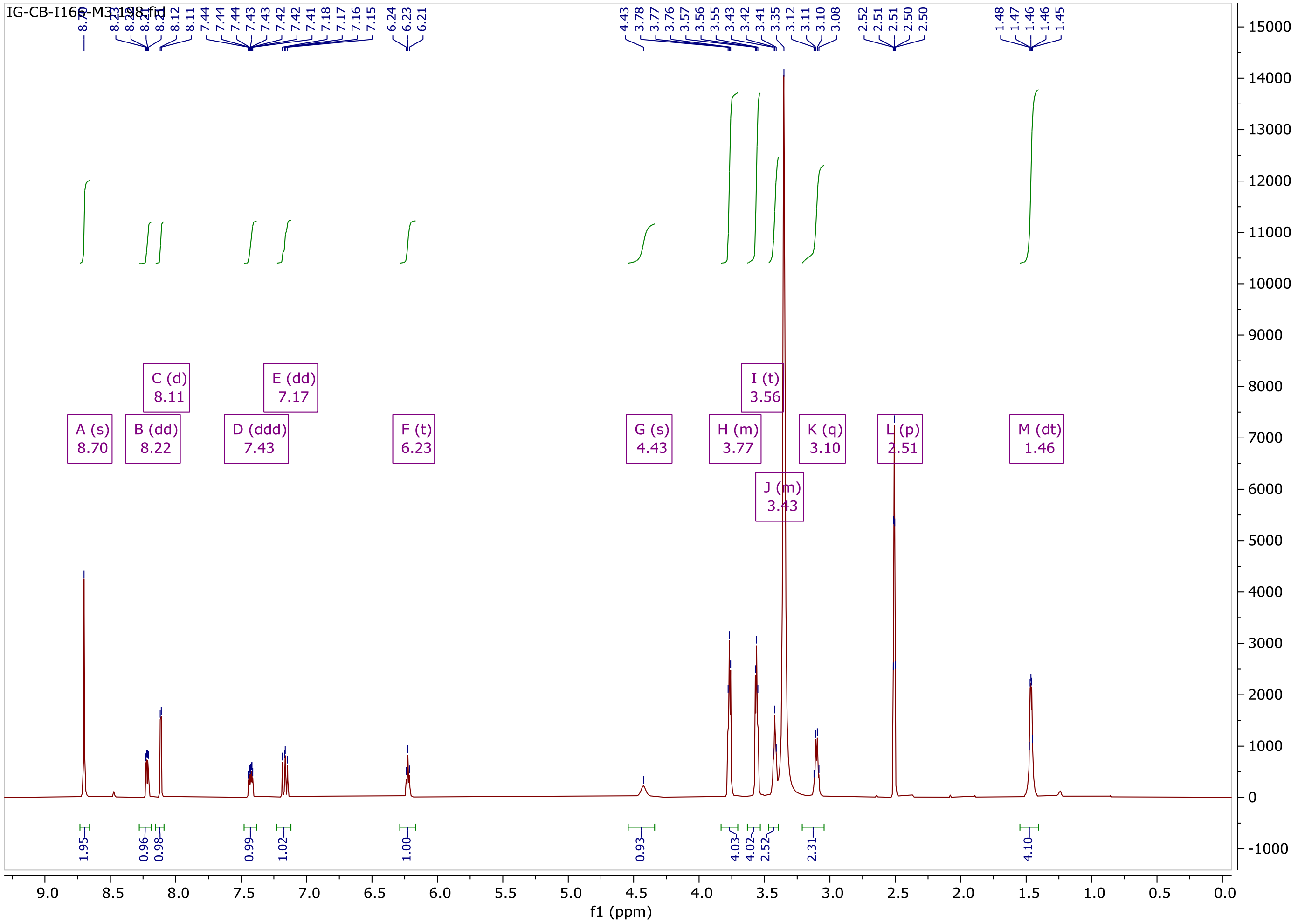

COSY

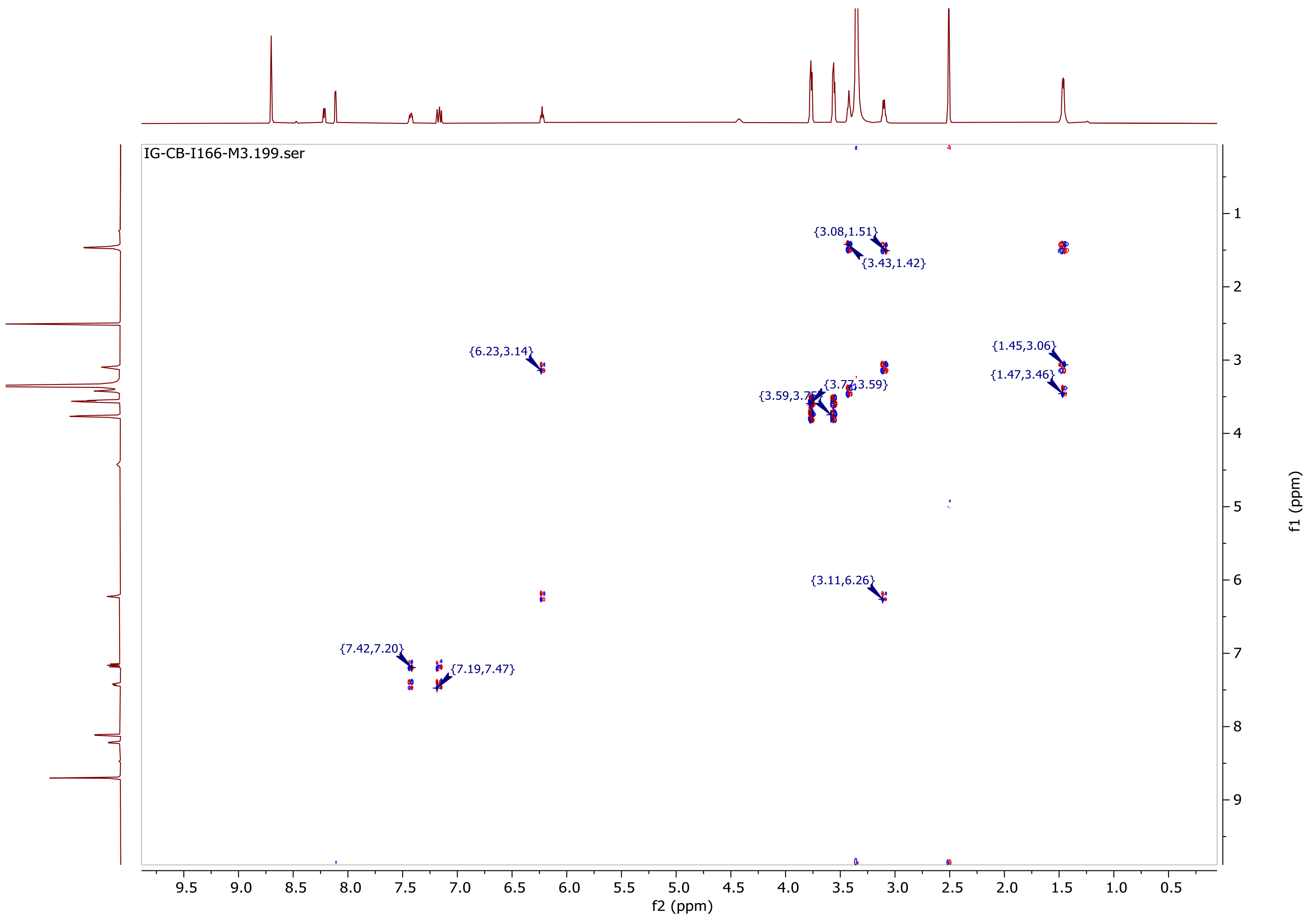
IG-CB-I166-M3.199.ser
{3.08,1.51}
{3.43,1.42}
{6.23,3.14}
{1.45,3.06}
{1.47,3.46}
{3.77,3.59}
{3.11,6.26}
{7.42,7.20}
{7.19,7.47}
f2 (ppm)
f1 (ppm)

HSQC

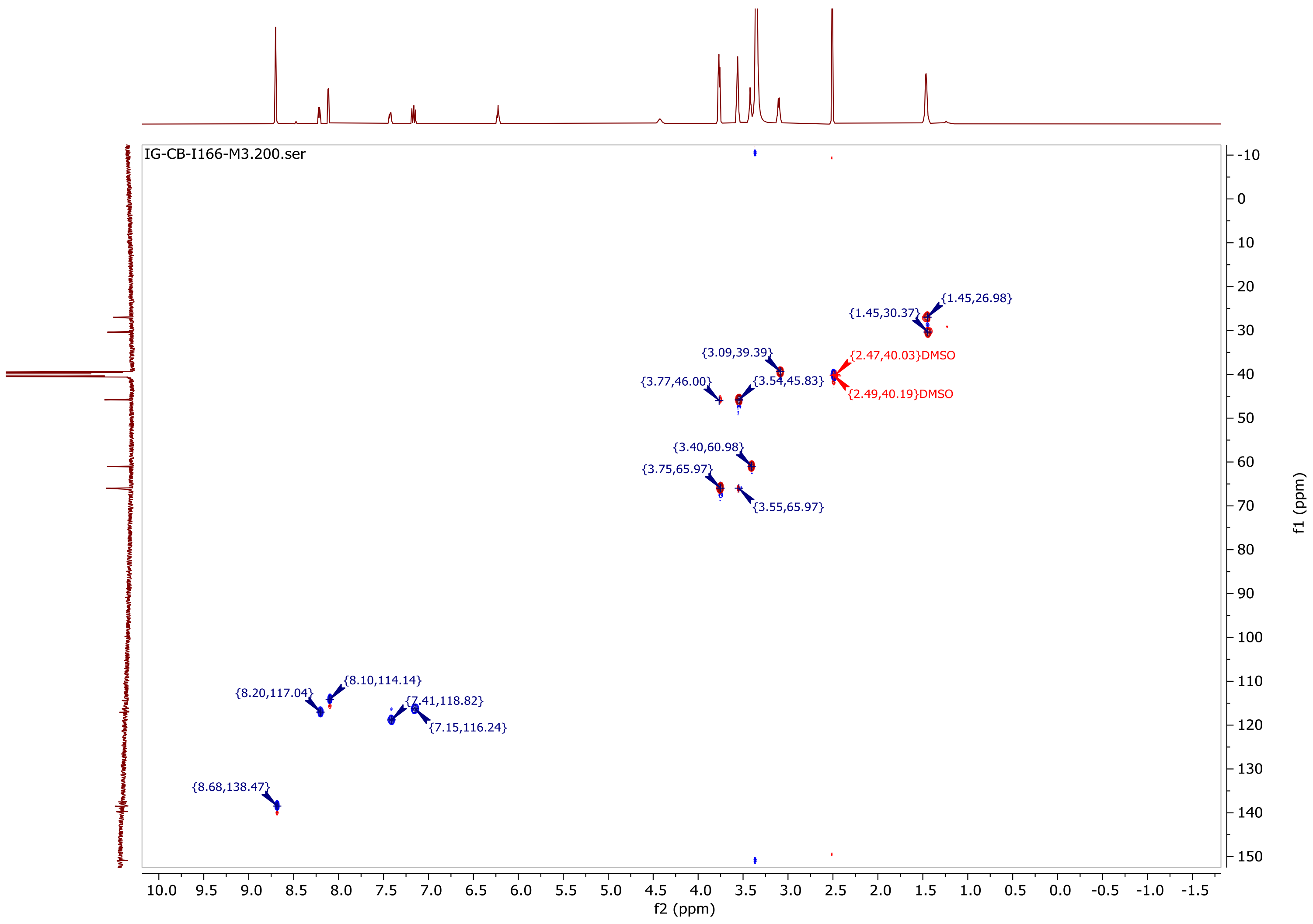
IG-CB-I166-M3.200.ser
{1.45,26.98}
{1.45,30.37}
{3.09,39.39}
{2.47,40.03}DMSO
{3.77,46.00}
{3.54,45.83}
{2.49,40.19}DMSO
{3.40,60.98}
{3.75,65.97}
{3.55,65.97}
{8.10,114.14}
{8.20,117.04}
{7.41,118.82}
{7.15,116.24}
{8.68,138.47}
f2 (ppm)
f1 (ppm)

HMBC

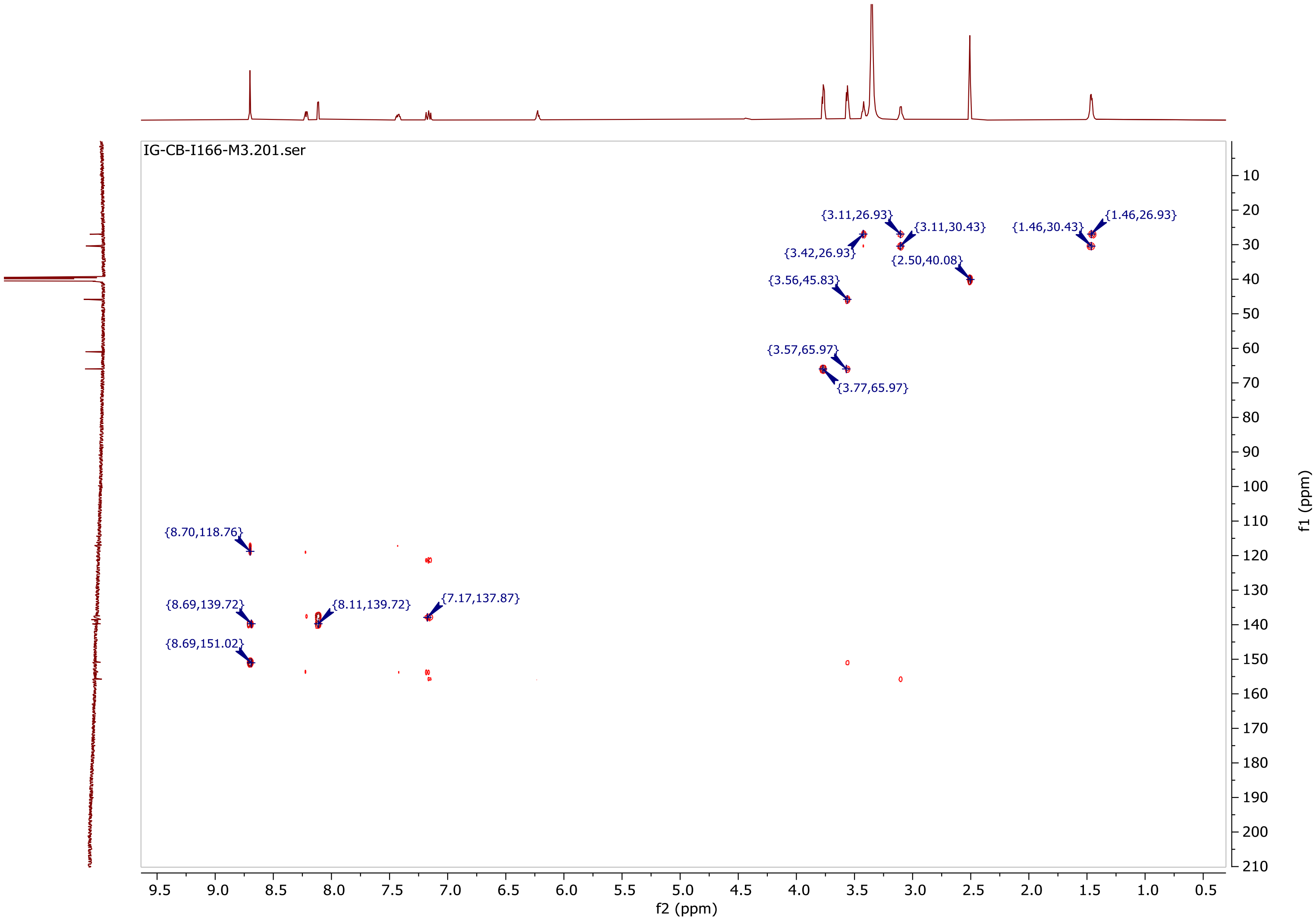
IG-CB-I166-M3.201.ser
{3.11,26.93}
{3.11,30.43}
{1.46,30.43}
{1.46,26.93}
{3.42,26.93}
{2.50,40.08}
{3.56,45.83}
{3.57,65.97}
{3.77,65.97}
{8.70,118.76}
{8.69,139.72}
{8.11,139.72}
{7.17,137.87}
{8.69,151.02}
f2 (ppm)
f1 (ppm)

NOESY

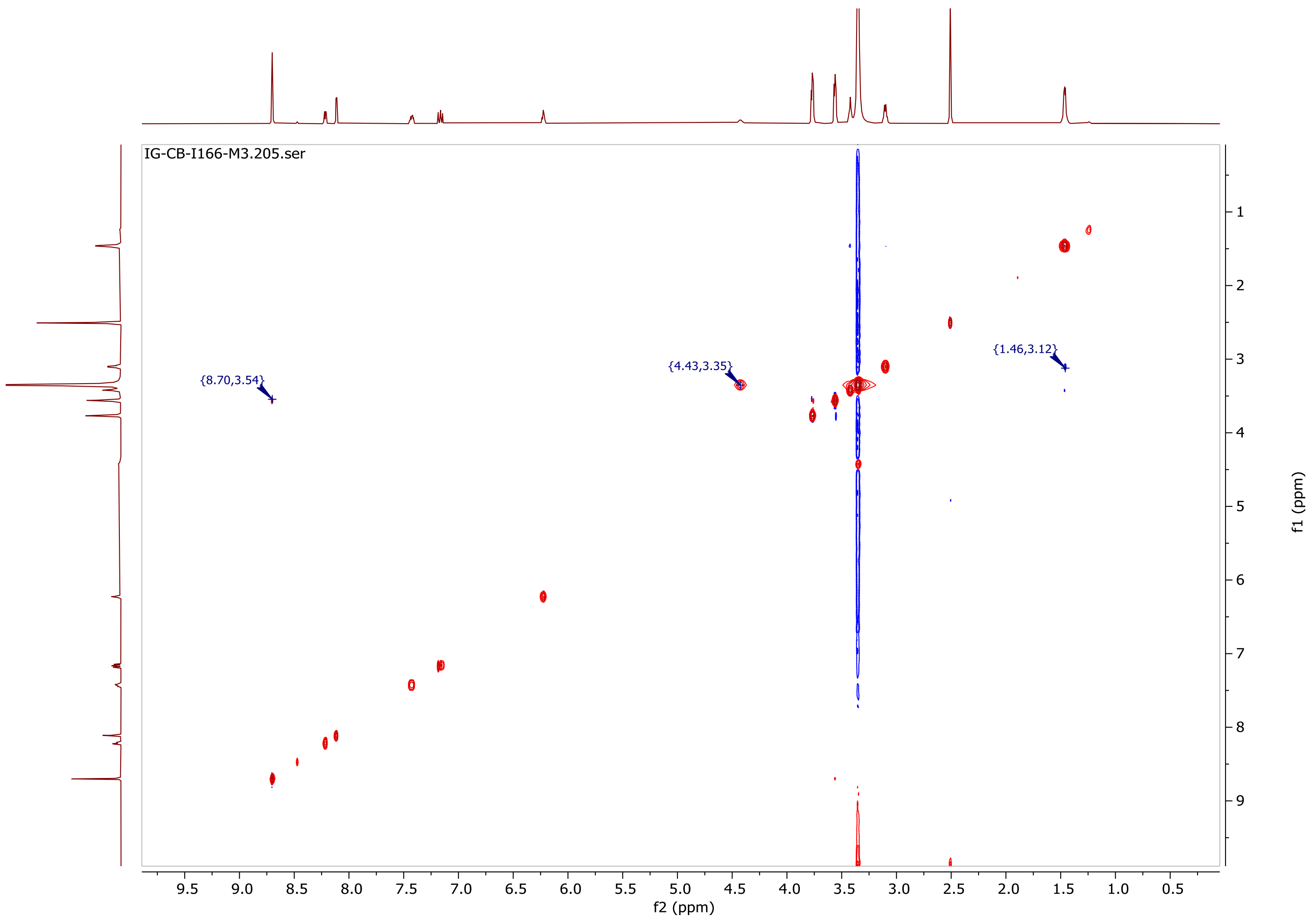

DEPTqgpsp

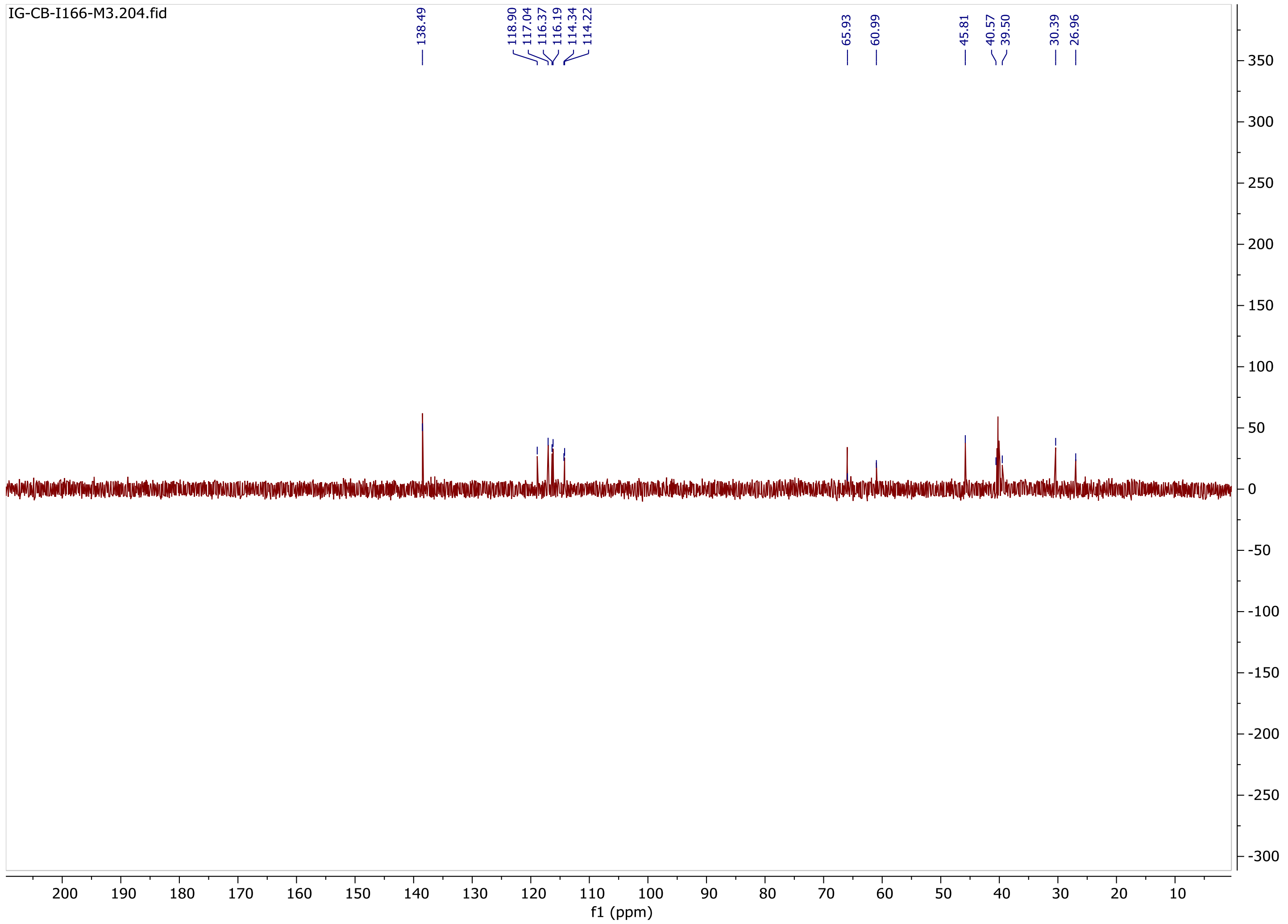
IG-CB-I166-M3.204.fid
138.49
118.90
117.04
116.37
116.19
114.34
114.22
65.93
60.99
45.81
40.57
39.50
30.39
26.96
350
300
250
200
150
100
50
0
-50
-100
-150
-200
-250
-300
200 190 180 170 160 150 140 130 120 110 100 90 80 70 60 50 40 30 20 10
f1 (ppm)

Zgpg30

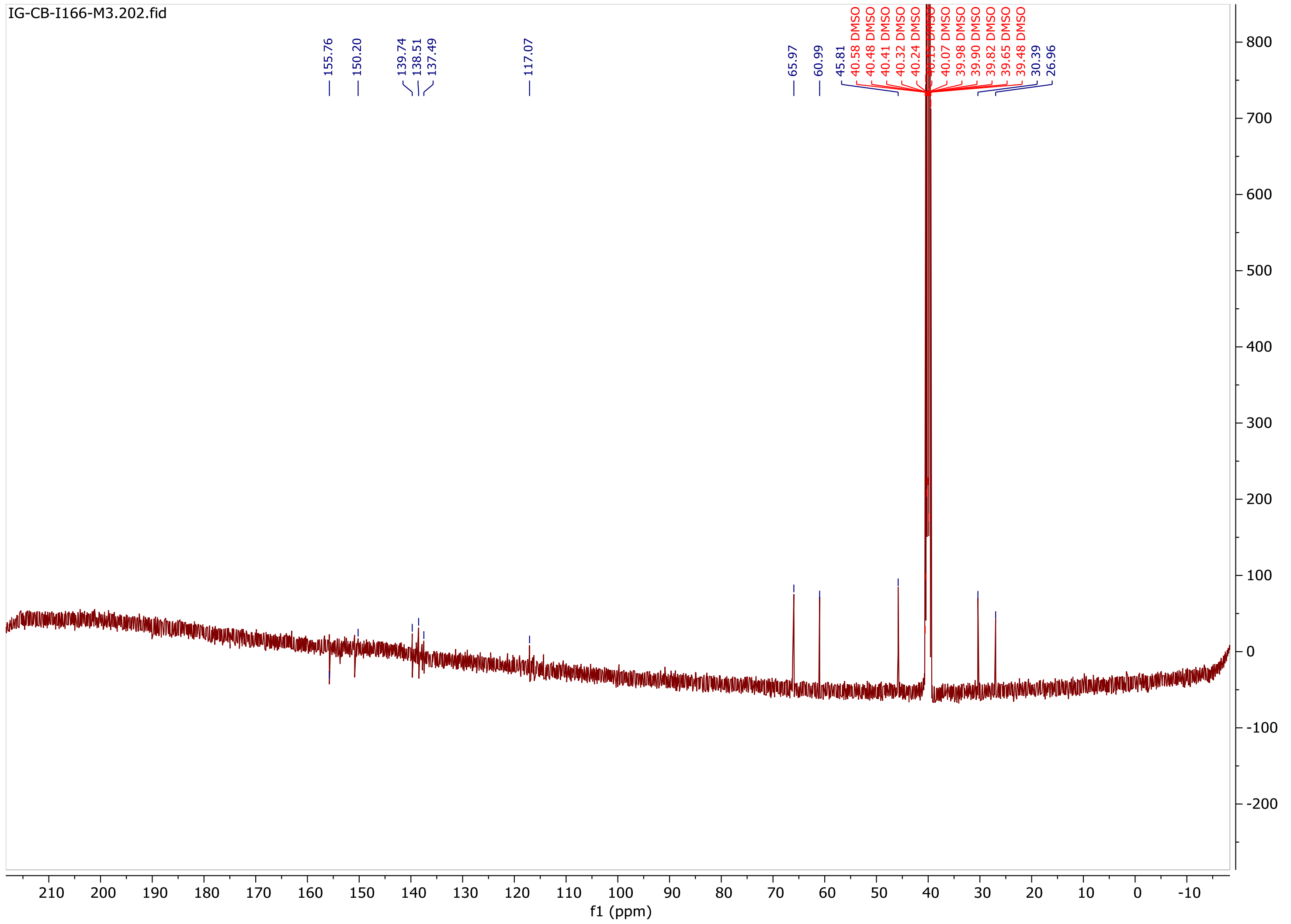

IG-CB-I166-M3.202.fid
155.76
150.20
139.74
138.51
137.49
117.07
65.97
60.99
45.81
40.58 DMSO
40.48 DMSO
40.41 DMSO
40.32 DMSO
40.24 DMSO
40.15 DMSO
40.07 DMSO
39.98 DMSO
39.90 DMSO
39.82 DMSO
39.65 DMSO
39.48 DMSO
30.39
26.96
f1 (ppm)

DEPT-135

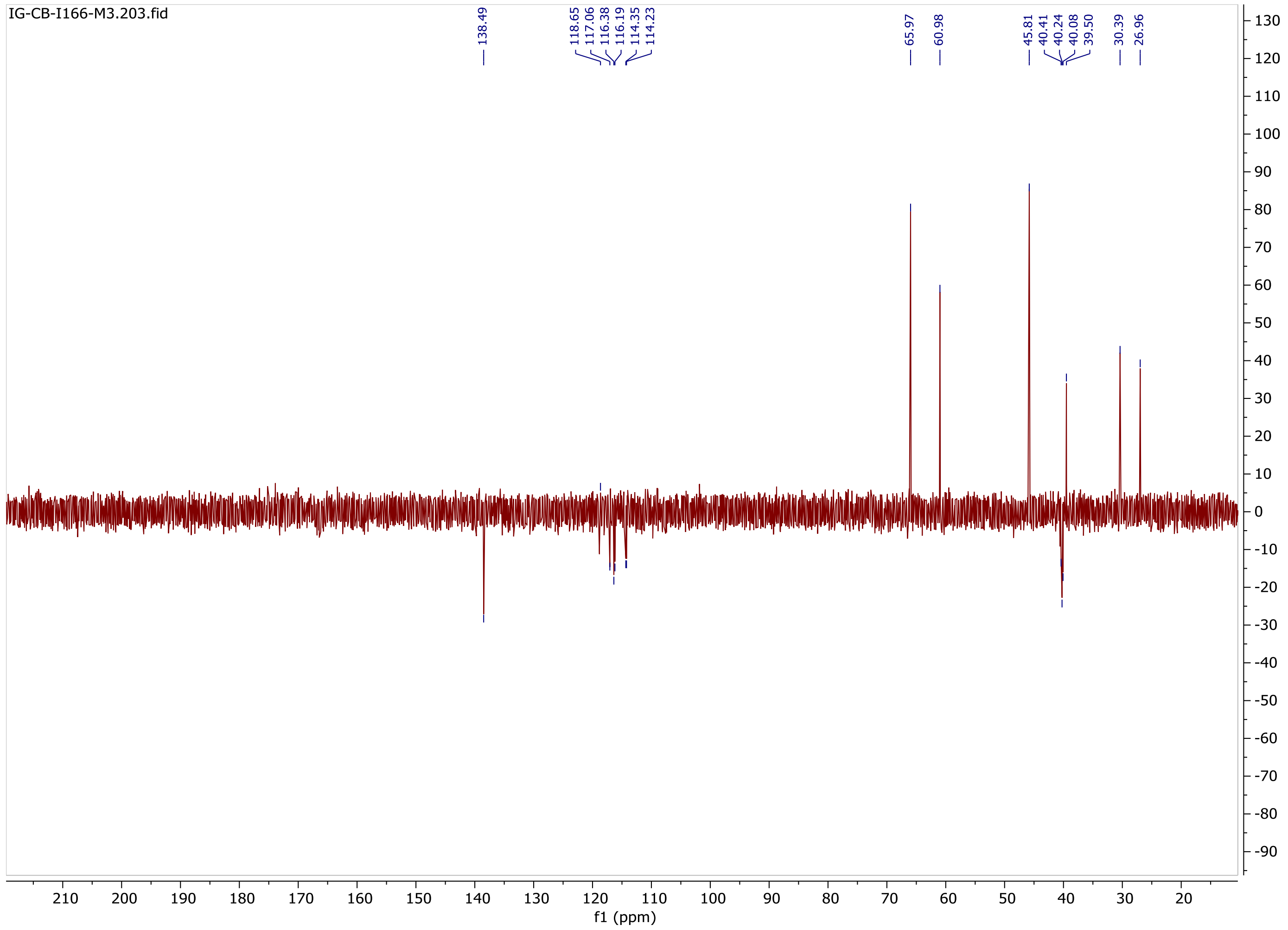

IG-CB-I166-M3.203.fid
138.49
118.65
117.06
116.38
116.19
114.35
114.23
65.97
60.98
45.81
40.41
40.24
40.08
39.50
30.39
26.96
f1 (ppm)

HRMS

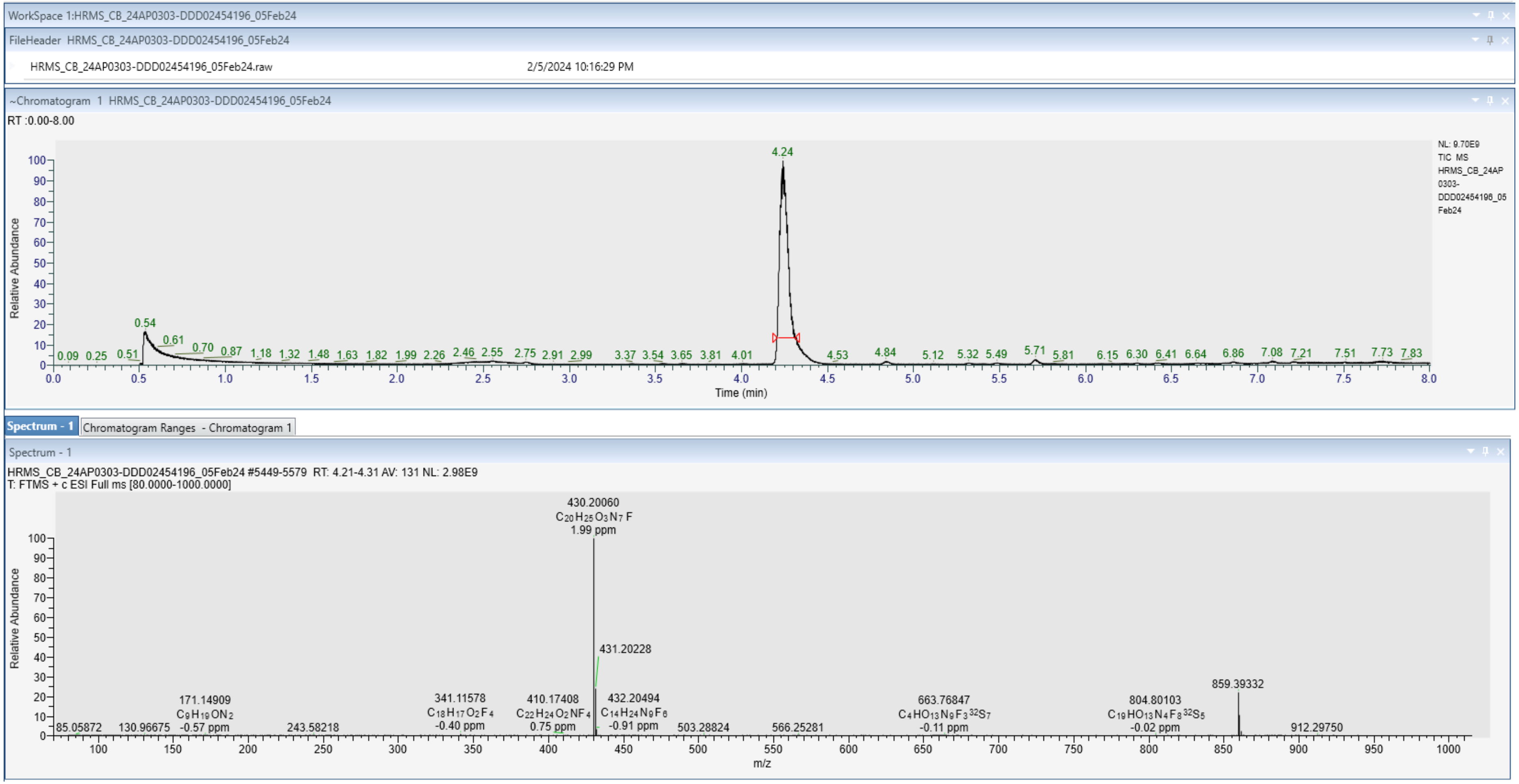
WorkSpace 1:HRMS_CB_24AP0303-DDD02454196_05Feb24
FileHeader HRMS_CB_24AP0303-DDD02454196_05Feb24
HRMS_CB_24AP0303-DDD02454196_05Feb24.raw
2/5/2024 10:16:29 PM
~Chromatogram 1 HRMS_CB_24AP0303-DDD02454196_05Feb24
RT :0.00-8.00
NL: 9.70E9
TIC MS
HRMS_CB_24AP0303-DDD02454196_05Feb24
4.24
Relative Abundance
Time (min)
Spectrum - 1
Chromatogram Ranges - Chromatogram 1
HRMS_CB_24AP0303-DDD02454196_05Feb24 #5449-5579 RT: 4.21-4.31 AV: 131 NL: 2.98E9
T: FTMS + c ESI Full ms [80.0000-1000.0000]
430.20060
C20 H25 O3 N7 F
1.99 ppm
431.20228
432.20494
C14 H24 N9 F6
-0.91 ppm
410.17408
C22 H24 O2 N F4
0.75 ppm
341.11578
C18 H17 O2 F4
-0.40 ppm
171.14909
C9 H19 O N2
-0.57 ppm
85.05872
130.96675
243.58218
503.28824
566.25281
663.76847
C4 H O13 N9 F3 32S7
-0.11 ppm
804.80103
C19 H O13 N4 F8 32S5
-0.02 ppm
859.39332
912.29750
m/z

Compound **10a**

^{1}H NMR

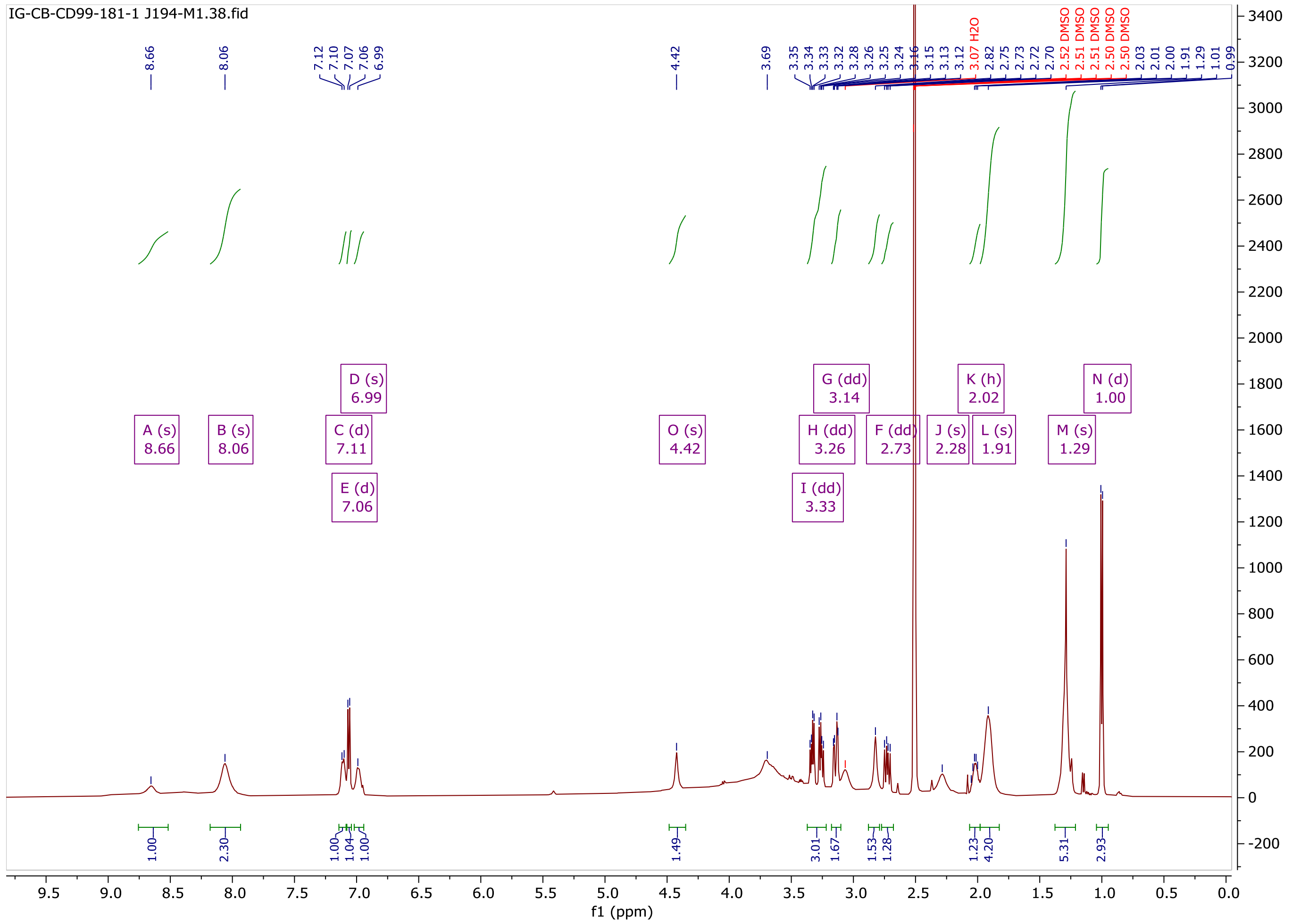

COSY

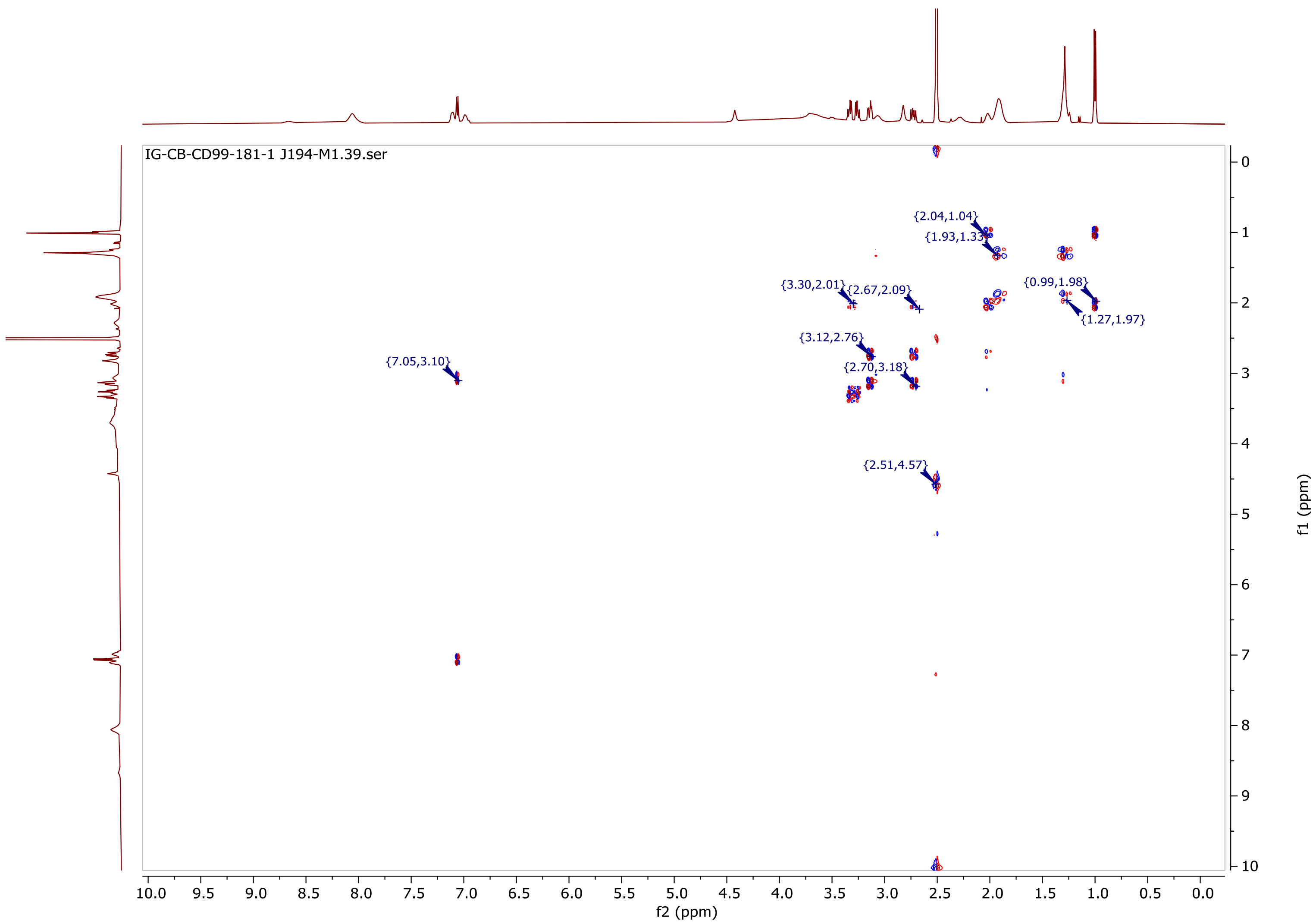
IG-CB-CD99-181-1 J194-M1.39.ser
{2.04,1.04}
{1.93,1.33}
{3.30,2.01}
{2.67,2.09}
{0.99,1.98}
{1.27,1.97}
{3.12,2.76}
{7.05,3.10}
{2.70,3.18}
{2.51,4.57}
f2 (ppm)
f1 (ppm)

HSQC

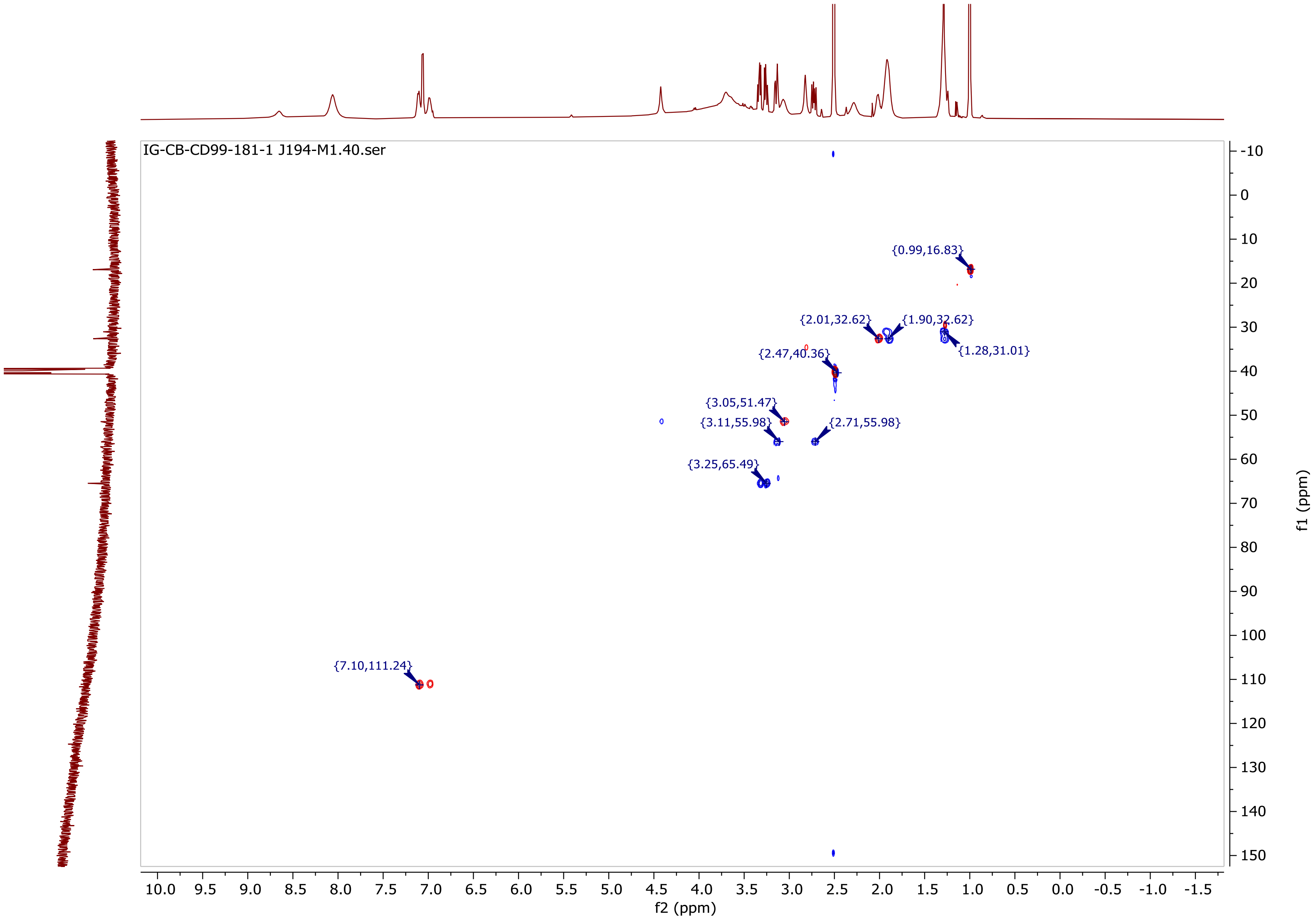

IG-CB-CD99-181-1 J194-M1.40.ser
{0.99,16.83}
{2.01,32.62}
{1.90,32.62}
{1.28,31.01}
{2.47,40.36}
{3.05,51.47}
{3.11,55.98}
{2.71,55.98}
{3.25,65.49}
{7.10,111.24}
f2 (ppm)
f1 (ppm)

NOESY

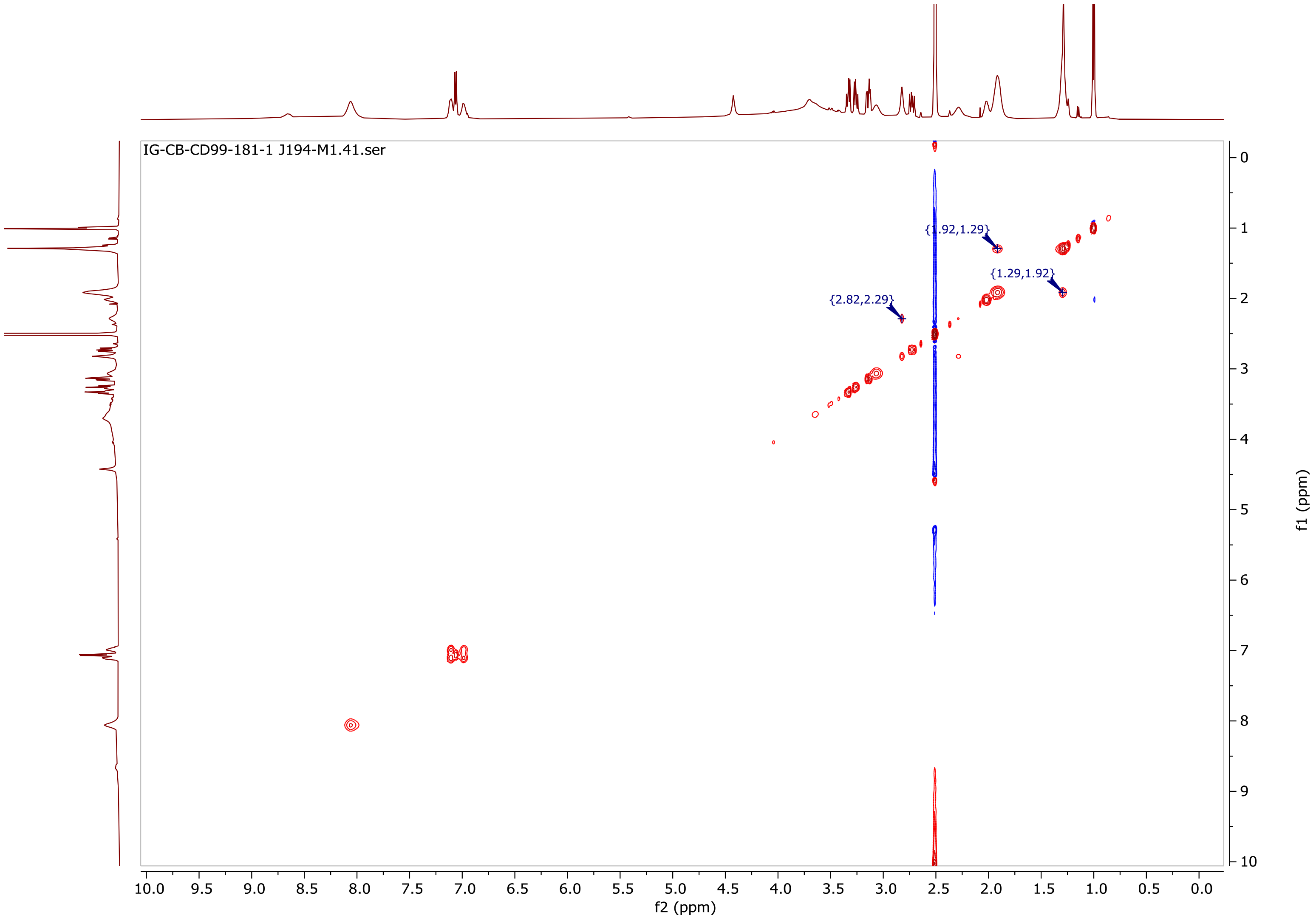
IG-CB-CD99-181-1 J194-M1.41.ser
{2.82,2.29}
{1.29,1.92}
f2 (ppm)
f1 (ppm)
10.0 9.5 9.0 8.5 8.0 7.5 7.0 6.5 6.0 5.5 5.0 4.5 4.0 3.5 3.0 2.5 2.0 1.5 1.0 0.5 0.0
0 1 2 3 4 5 6 7 8 9 10

DEPTqgpsp

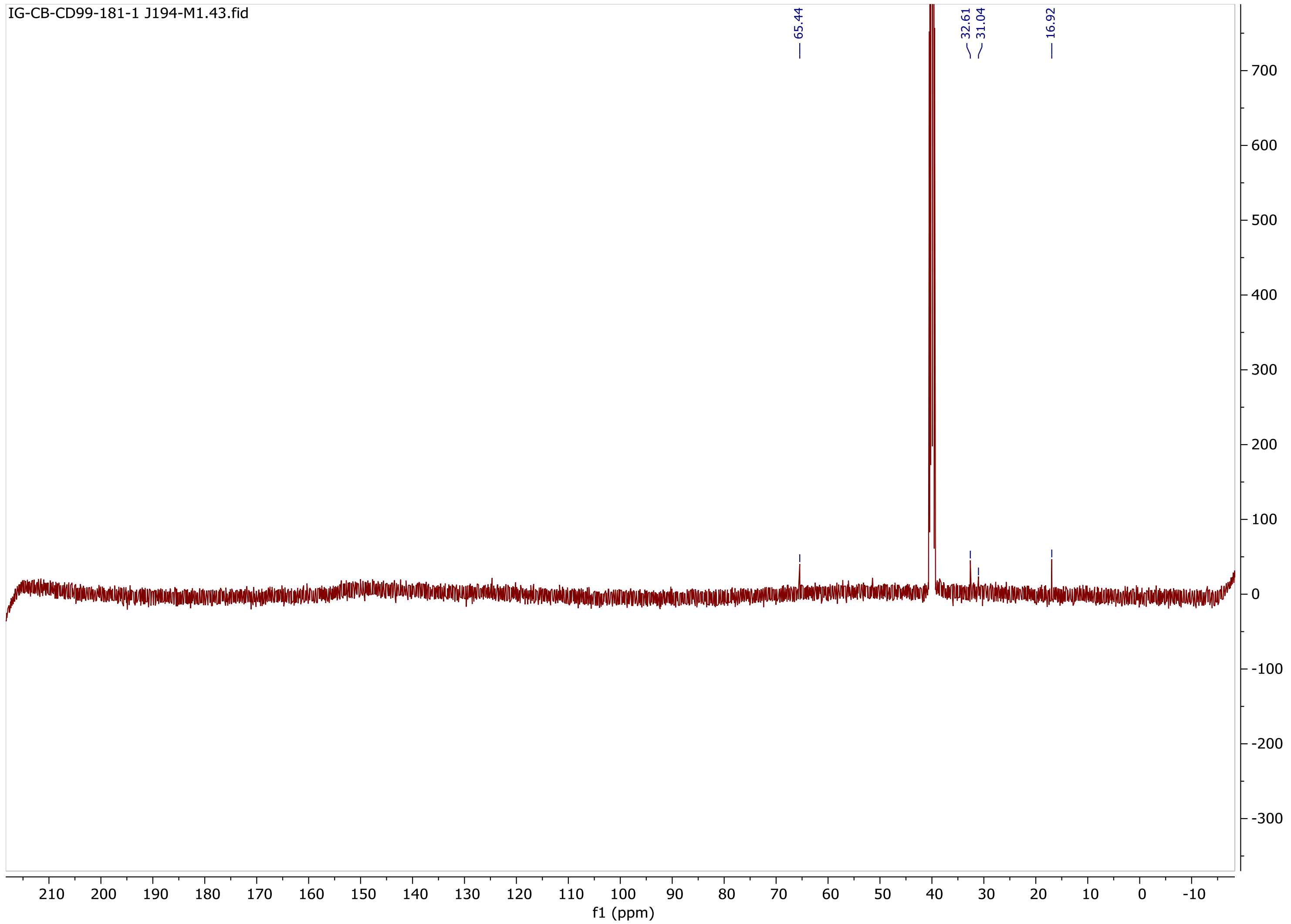
IG-CB-CD99-181-1 J194-M1.43.fid
65.44
32.61
31.04
16.92
700
600
500
400
300
200
100
0
-100
-200
-300
210
200
190
180
170
160
150
140
130
120
110
100
90
80
70
60
50
40
30
20
10
0
-10
f1 (ppm)

DEPT-135

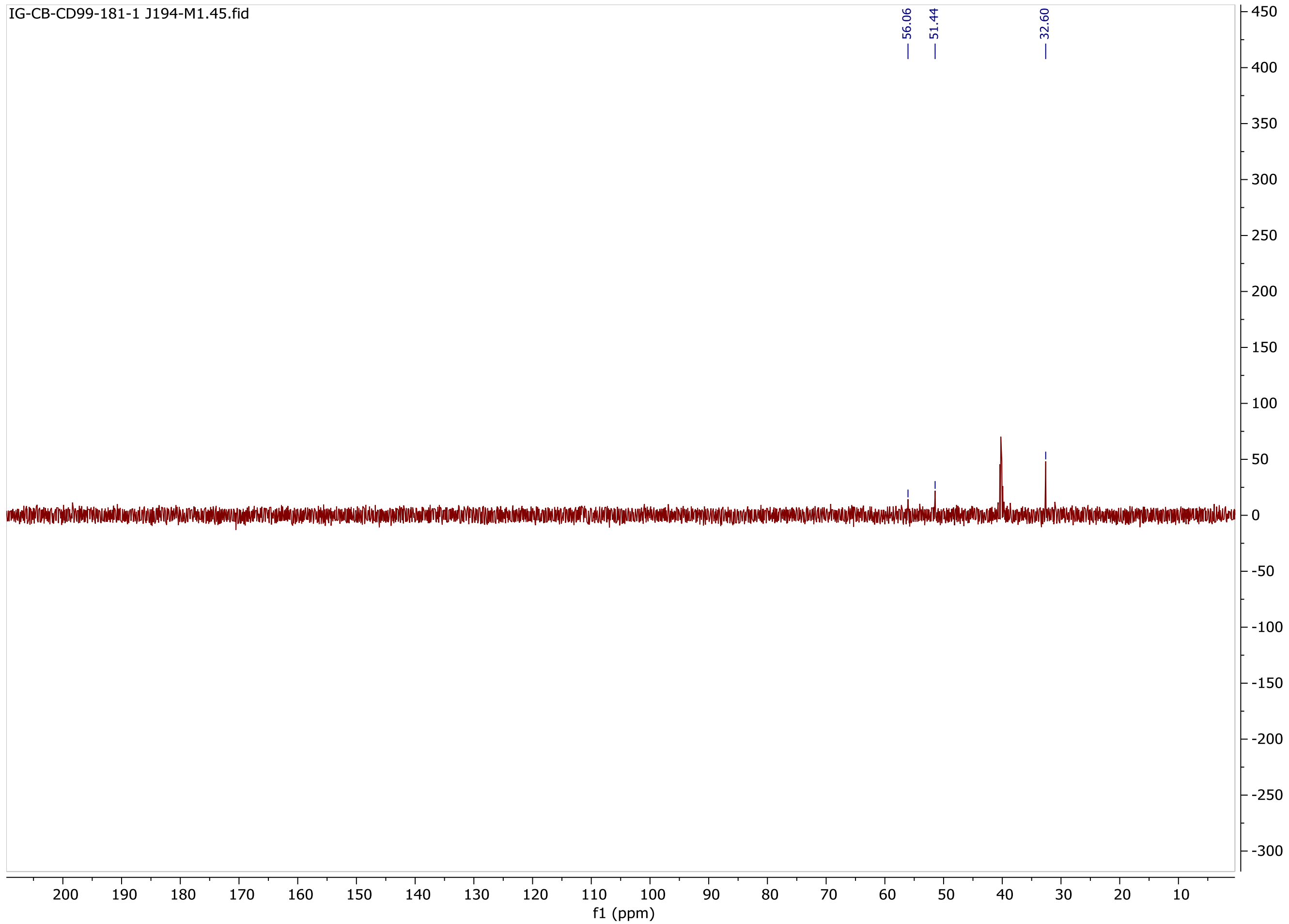

IG-CB-CD99-181-1 J194-M1.45.fid
56.06
51.44
32.60
450
400
350
300
250
200
150
100
50
0
-50
-100
-150
-200
-250
-300
200 190 180 170 160 150 140 130 120 110 100 90 80 70 60 50 40 30 20 10
f1 (ppm)

DEPT-90

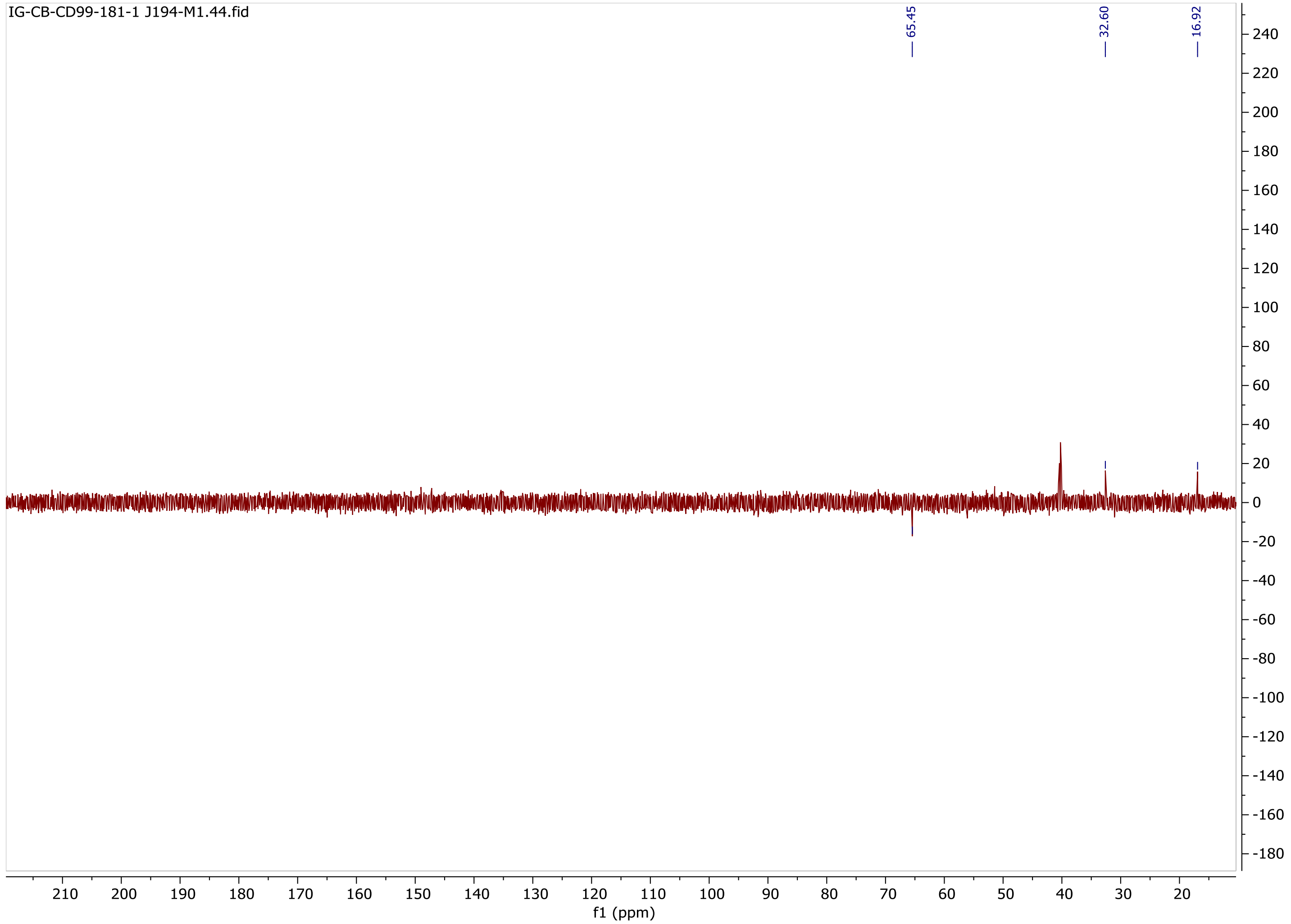

IG-CB-CD99-181-1 J194-M1.44.fid
65.45
32.60
16.92
240
220
200
180
160
140
120
100
80
60
40
20
0
-20
-40
-60
-80
-100
-120
-140
-160
-180
210
200
190
180
170
160
150
140
130
120
110
100
90
80
70
60
50
40
30
20
f1 (ppm)

HRMS

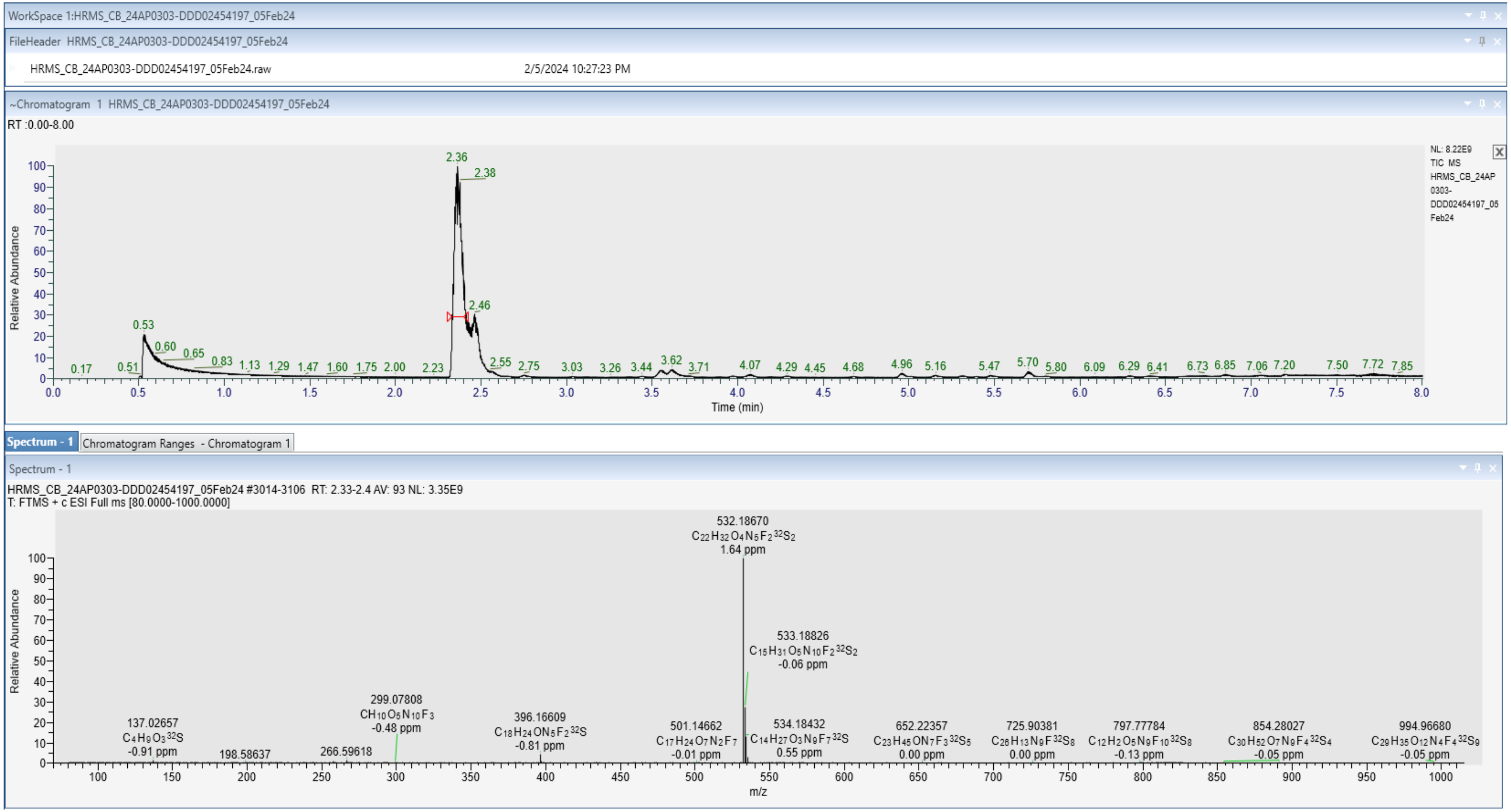
WorkSpace 1:HRMS_CB_24AP0303-DDD02454197_05Feb24
FileHeader HRMS_CB_24AP0303-DDD02454197_05Feb24
HRMS_CB_24AP0303-DDD02454197_05Feb24.raw
2/5/2024 10:27:23 PM
~Chromatogram 1 HRMS_CB_24AP0303-DDD02454197_05Feb24
RT :0.00-8.00
NL: 8.22E9
TIC MS
HRMS_CB_24AP0303-DDD02454197_05Feb24
Relative Abundance
Time (min)
Spectrum - 1
Chromatogram Ranges - Chromatogram 1
HRMS_CB_24AP0303-DDD02454197_05Feb24 #3014-3106 RT: 2.33-2.4 AV: 93 NL: 3.35E9
T: FTMS + c ESI Full ms [80.0000-1000.0000]
532.18670
C22 H32 O4 N6 F2 32S2
1.64 ppm
533.18826
C15 H31 O5 N10 F2 32S2
-0.06 ppm
299.07808
CH10 O5 N10 F3
-0.48 ppm
137.02657
C4 H9 O3 32S
-0.91 ppm
396.16609
C18 H24 ON5 F2 32S
-0.81 ppm
501.14662
C17 H24 O7 N2 F7
-0.01 ppm
534.18432
C14 H27 O3 N9 F7 32S
0.55 ppm
652.22357
C23 H45 ON7 F3 32S5
0.00 ppm
725.90381
C26 H13 N9 F 32S8
0.00 ppm
797.77784
C12 H2 O5 N9 F10 32S8
-0.13 ppm
854.28027
C30 H52 O7 N9 F4 32S4
-0.05 ppm
994.96680
C29 H35 O12 N4 F4 32S9
-0.05 ppm
198.58637
266.59618
m/z

Compound **10b**

^{1}H NMR

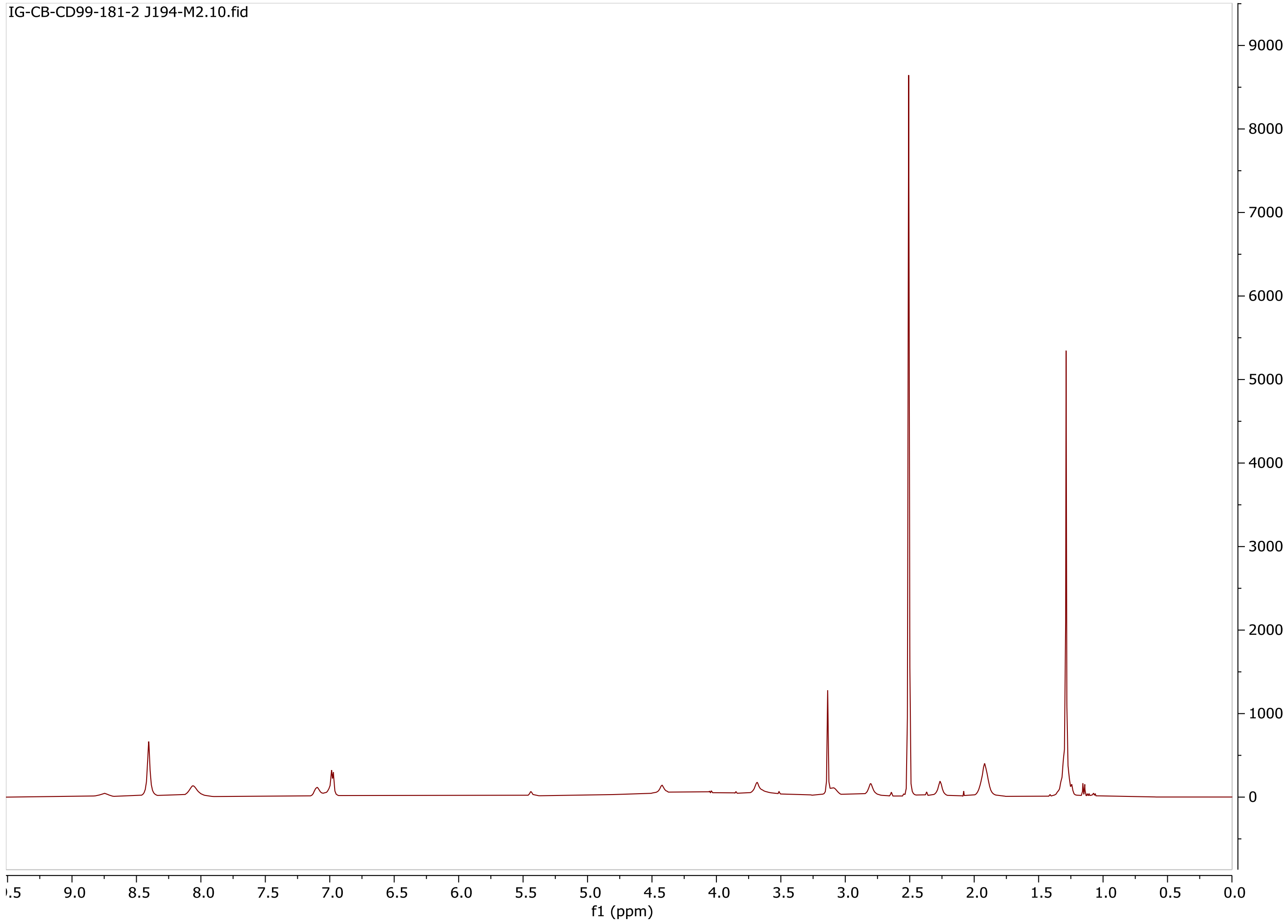

COSY

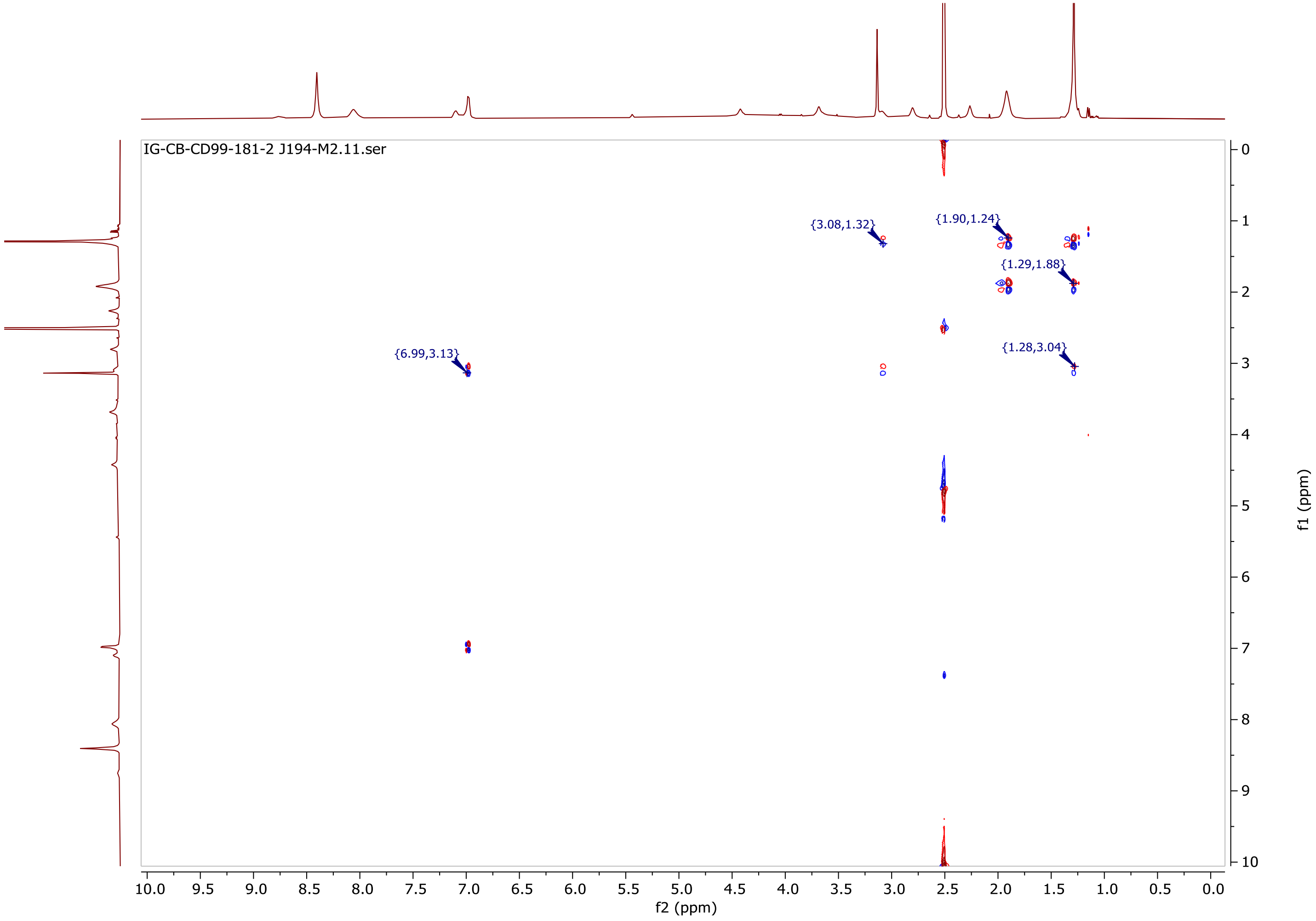
IG-CB-CD99-181-2 J194-M2.11.ser
{3.08,1.32}
{1.90,1.24}
{1.29,1.88}
{6.99,3.13}
{1.28,3.04}
10.0 9.5 9.0 8.5 8.0 7.5 7.0 6.5 6.0 5.5 5.0 4.5 4.0 3.5 3.0 2.5 2.0 1.5 1.0 0.5 0.0
f2 (ppm)
0 1 2 3 4 5 6 7 8 9 10
f1 (ppm)

HSQC

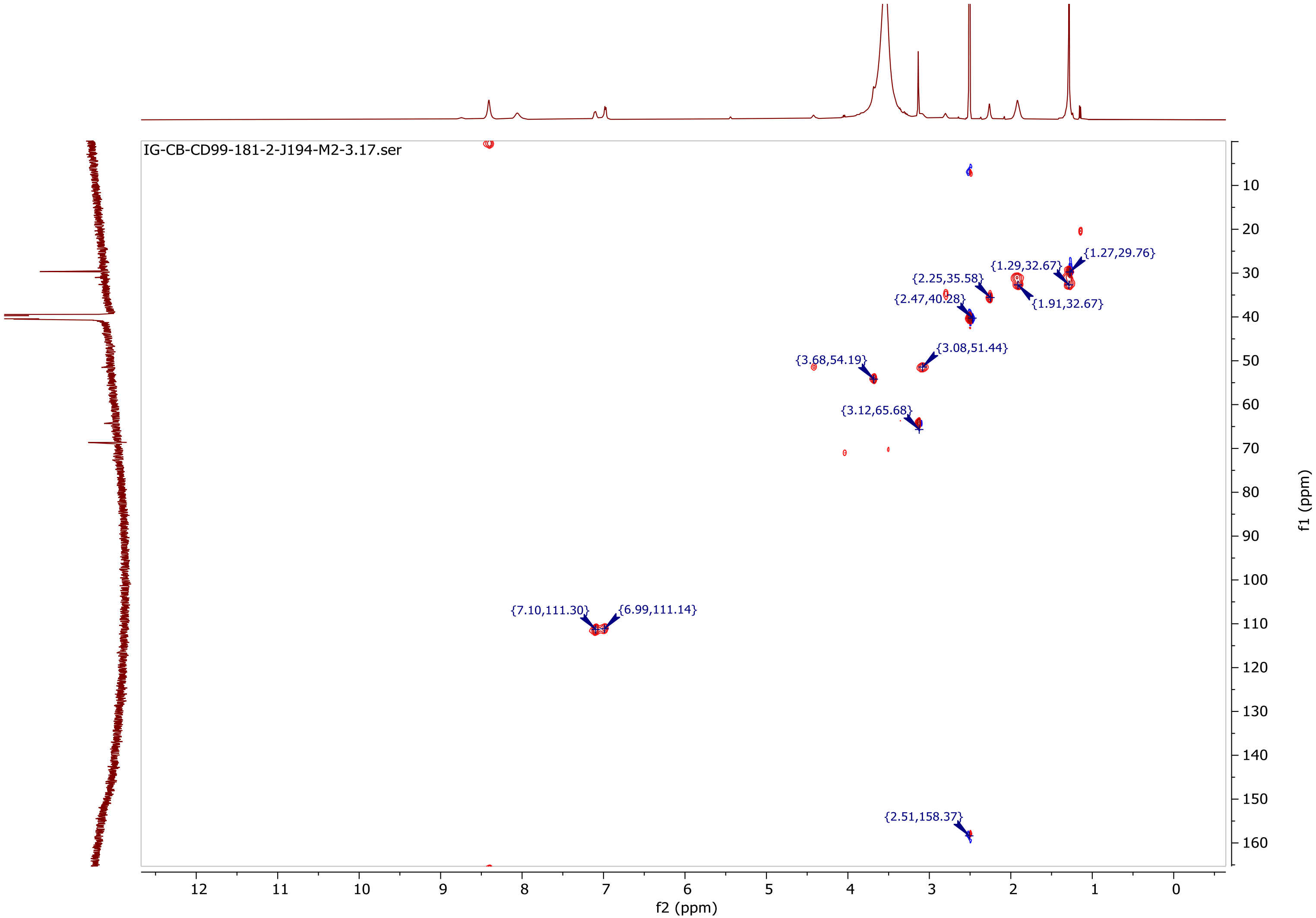

IG-CB-CD99-181-2-J194-M2-3.17.ser
{1.27,29.76}
{1.29,32.67}
{2.25,35.58}
{2.47,40.28}
{1.91,32.67}
{3.08,51.44}
{3.68,54.19}
{3.12,65.68}
{7.10,111.30}
{6.99,111.14}
{2.51,158.37}
f2 (ppm)
f1 (ppm)

HMBC

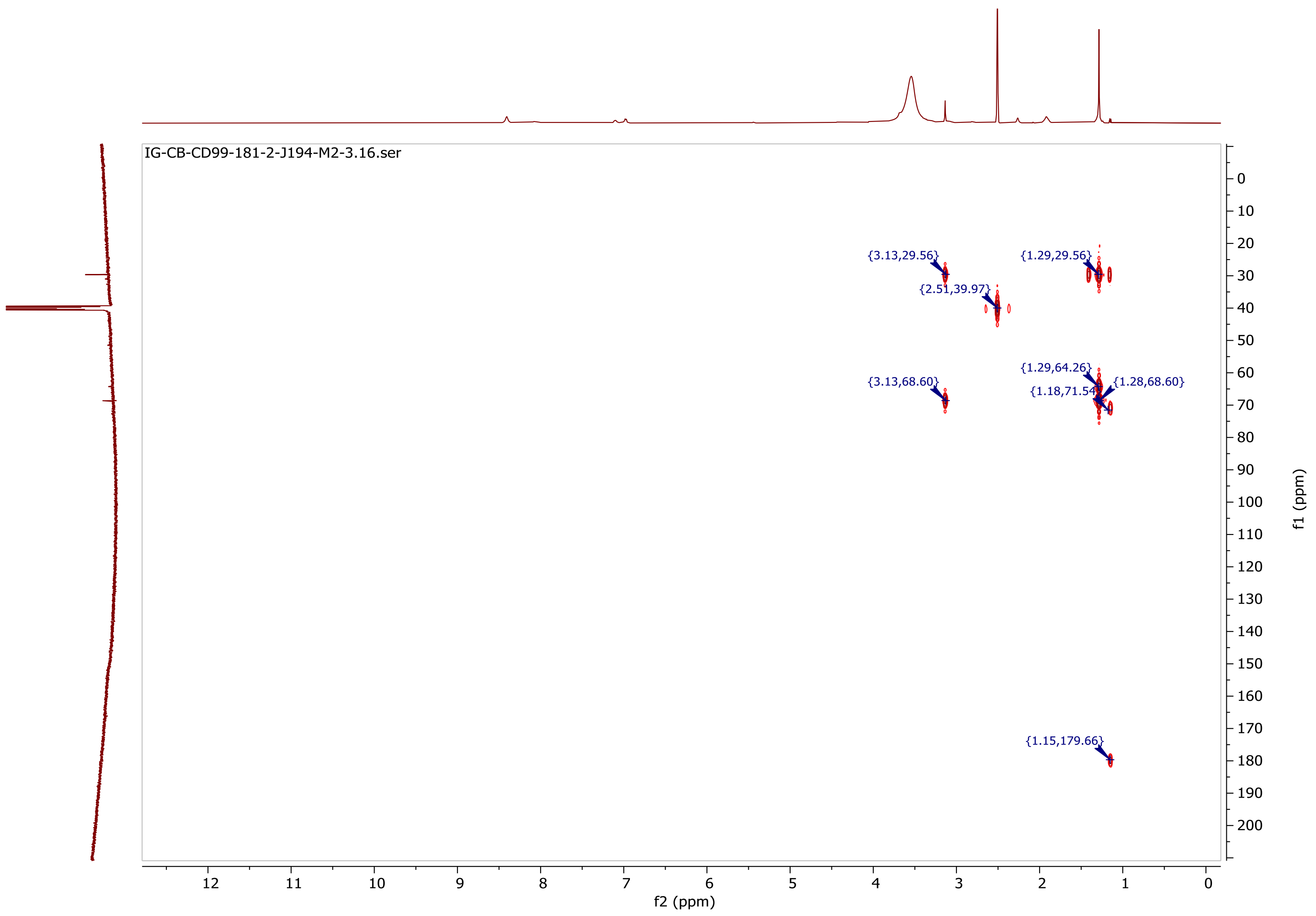

IG-CB-CD99-181-2-J194-M2-3.16.ser
{3.13,29.56}
{1.29,29.56}
{2.51,39.97}
{1.29,64.26}
{3.13,68.60}
{1.28,68.60}
{1.18,71.54}
{1.15,179.66}
f2 (ppm)
f1 (ppm)

NOESY

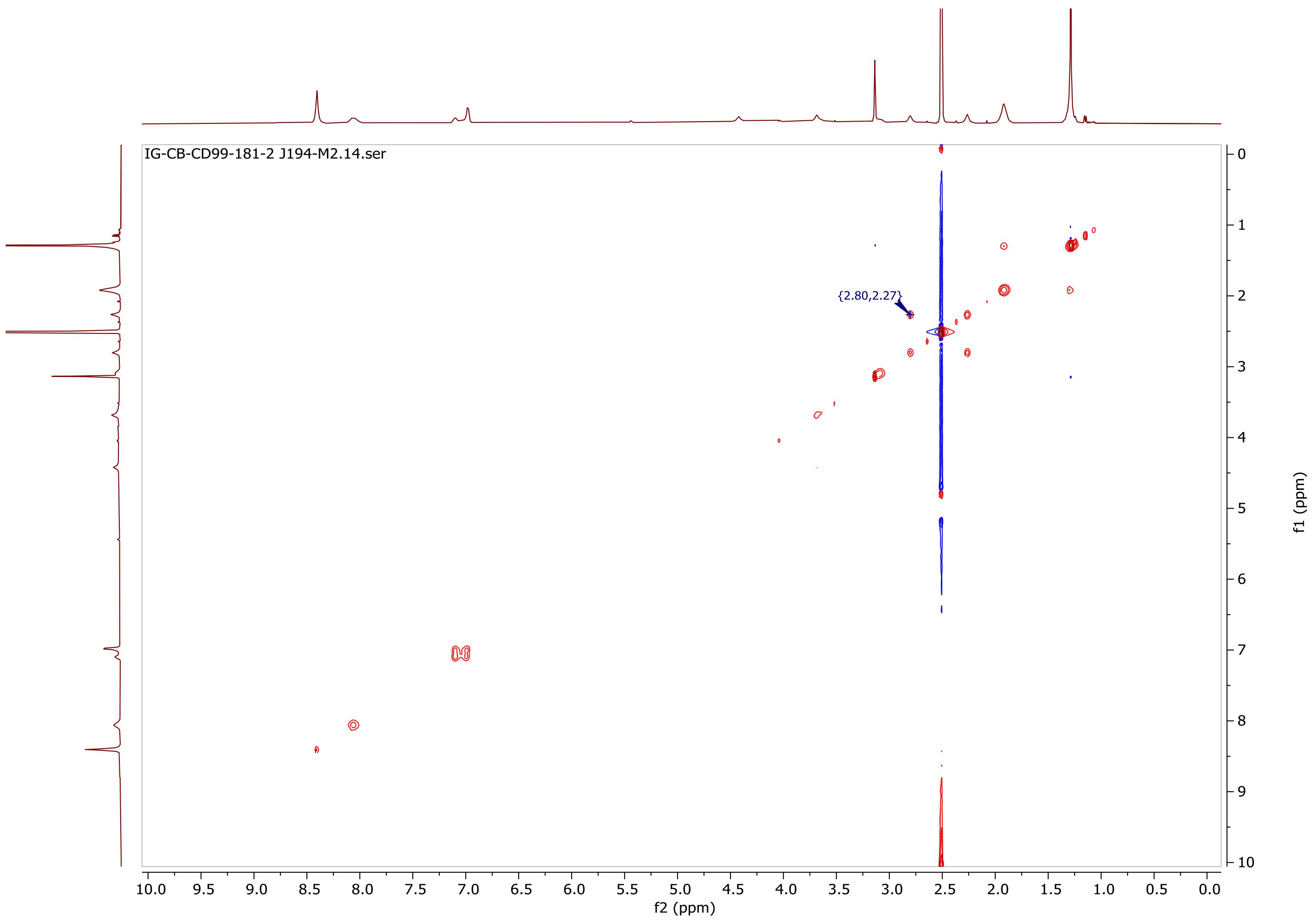
IG-CB-CD99-181-2 J194-M2.14.ser
{2.80,2.27}
f2 (ppm)
f1 (ppm)
10.0 9.5 9.0 8.5 8.0 7.5 7.0 6.5 6.0 5.5 5.0 4.5 4.0 3.5 3.0 2.5 2.0 1.5 1.0 0.5 0.0
0 1 2 3 4 5 6 7 8 9 10

DEPTqgpsp

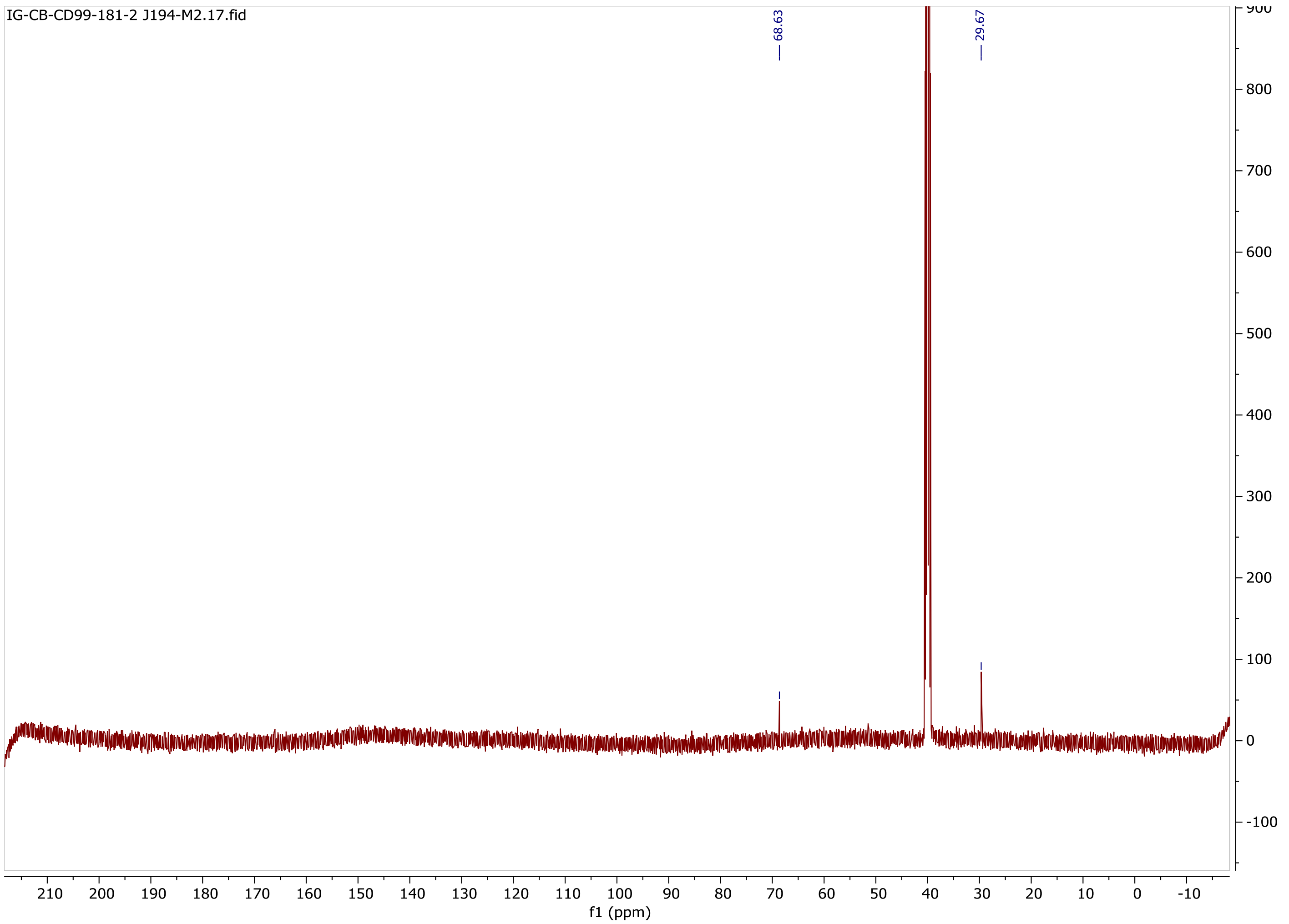
IG-CB-CD99-181-2 J194-M2.17.fid
68.63
29.67
900
800
700
600
500
400
300
200
100
0
-100
210
200
190
180
170
160
150
140
130
120
110
100
90
80
70
60
50
40
30
20
10
0
-10
f1 (ppm)

zgpg30

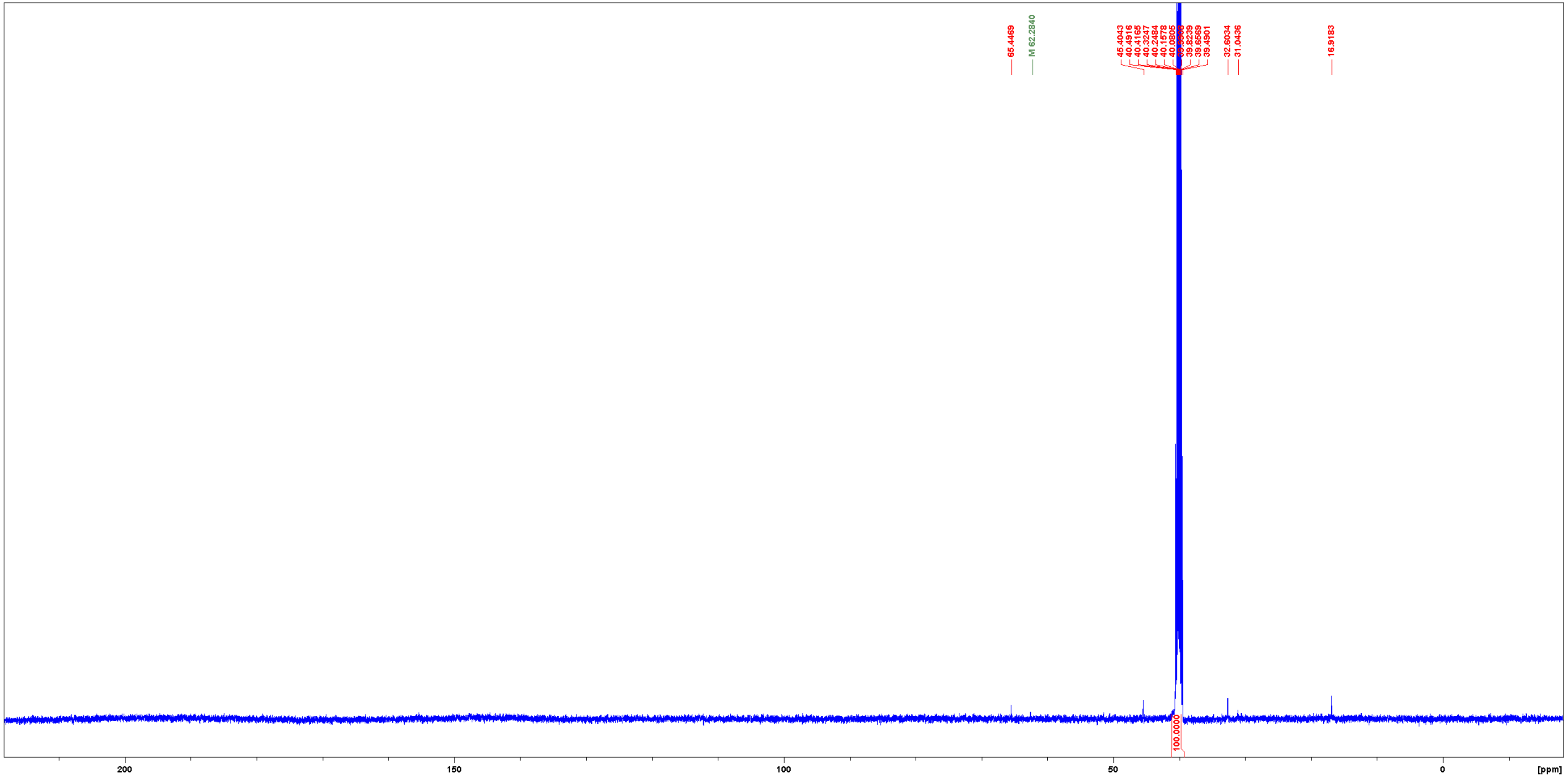
65.4469
M 62.2840
45.4043
40.4916
40.4165
40.3247
40.2484
40.1578
40.0805
39.9908
39.8239
39.6569
39.4901
32.6034
31.0436
16.9183
100.0000
200
150
100
50
0
[ppm]

DEPT-135

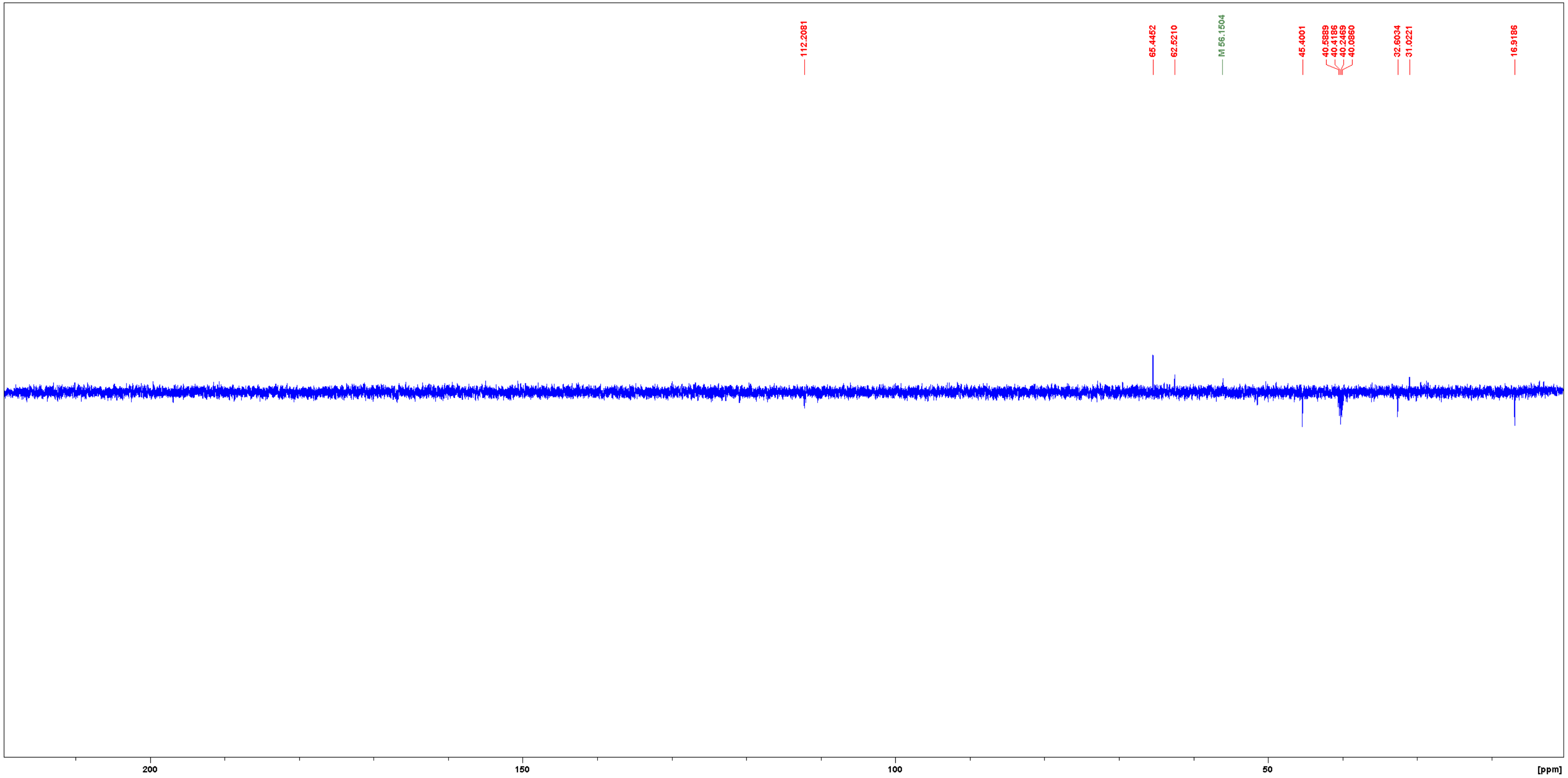

112.2081
65.4452
62.5210
M 56.1504
45.4001
40.5889
40.4186
40.2469
40.0860
32.6034
31.0221
16.9186
200
150
100
50
[ppm]

HRMS

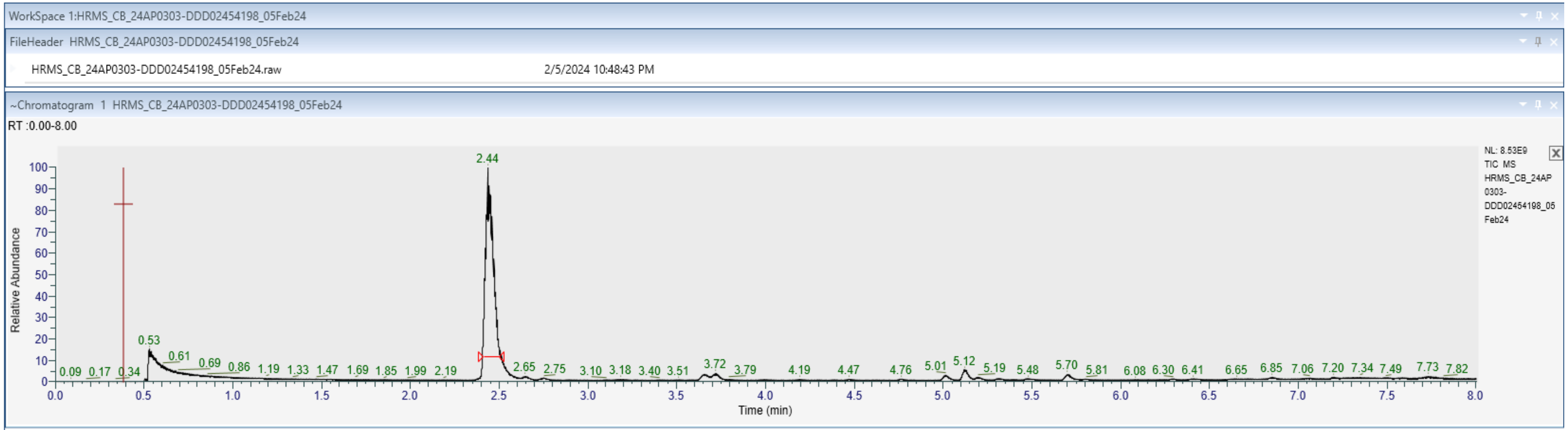

WorkSpace 1:HRMS_CB_24AP0303-DDD02454198_05Feb24
FileHeader HRMS_CB_24AP0303-DDD02454198_05Feb24
HRMS_CB_24AP0303-DDD02454198_05Feb24.raw
2/5/2024 10:48:43 PM
~Chromatogram 1 HRMS_CB_24AP0303-DDD02454198_05Feb24
RT :0.00-8.00
NL: 8.53E9 TIC MS HRMS_CB_24AP0303-DDD02454198_05Feb24
Relative Abundance
Time (min)
2.44
0.53

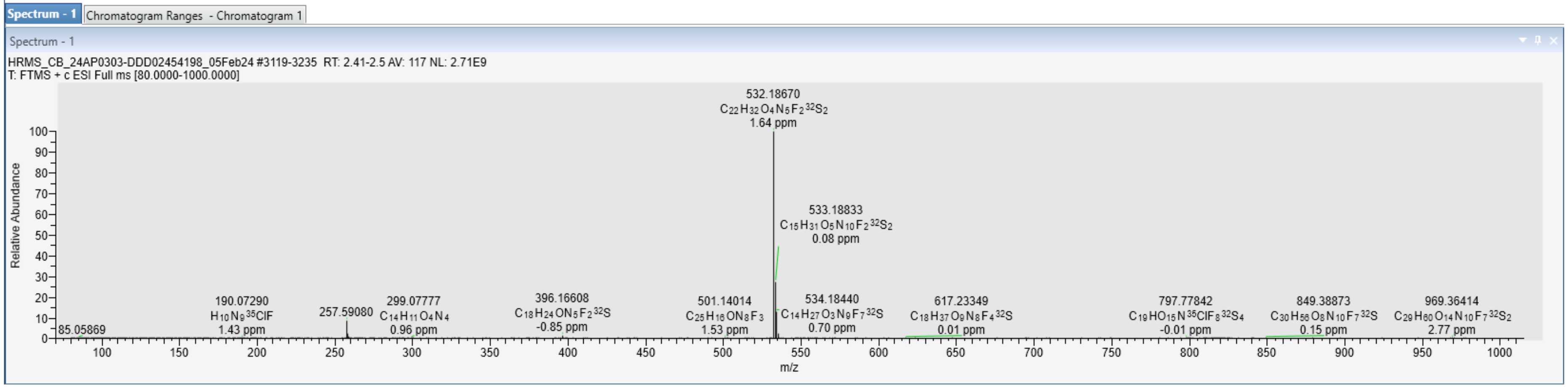

Spectrum - 1
Chromatogram Ranges - Chromatogram 1
HRMS_CB_24AP0303-DDD02454198_05Feb24 #3119-3235 RT: 2.41-2.5 AV: 117 NL: 2.71E9
T: FTMS + c ESI Full ms [80.0000-1000.0000]
532.18670
C22 H32 O4 N5 F2 32S2
1.64 ppm
533.18833
C15 H31 O5 N10 F2 32S2
0.08 ppm
534.18440
C14 H27 O3 N9 F7 32S
0.70 ppm
501.14014
C25 H16 ON8 F3
1.53 ppm
396.16608
C18 H24 ON5 F2 32S
-0.85 ppm
299.07777
C14 H11 O4 N4
0.96 ppm
257.59080
190.07290
H10 N9 35ClF
1.43 ppm
85.05869
617.23349
C18 H37 O9 N8 F4 32S
0.01 ppm
797.77842
C19 HO15 N 35ClF8 32S4
-0.01 ppm
849.38873
C30 H56 O8 N10 F7 32S
0.15 ppm
969.36414
C29 H60 O14 N10 F7 32S2
2.77 ppm
Relative Abundance
m/z

Compound **11a**

^{1}H NMR

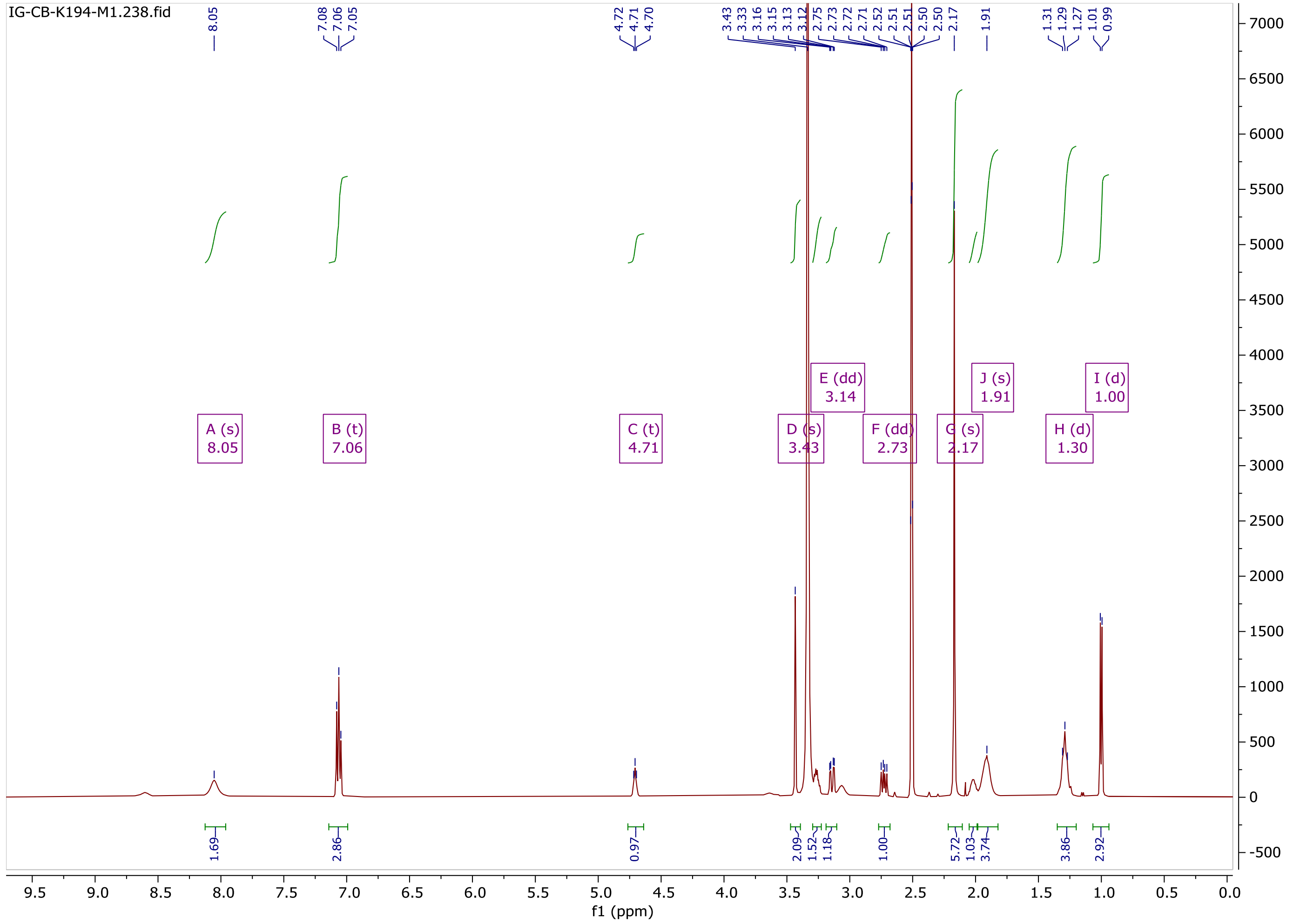

COSY

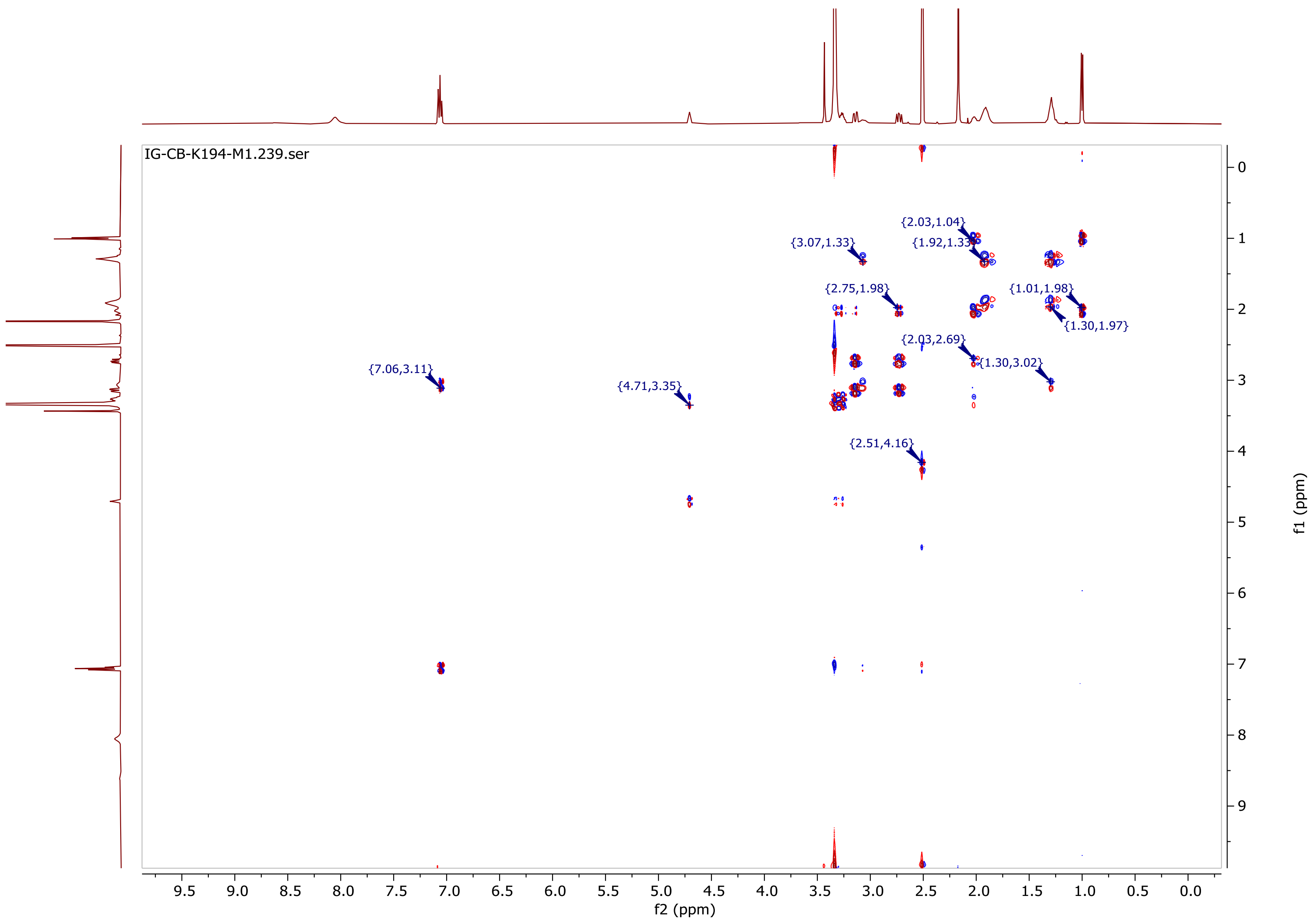
IG-CB-K194-M1.239.ser
{2.03,1.04}
{3.07,1.33}
{1.92,1.33}
{2.75,1.98}
{1.01,1.98}
{1.30,1.97}
{2.03,2.69}
{1.30,3.02}
{7.06,3.11}
{4.71,3.35}
{2.51,4.16}
f2 (ppm)
f1 (ppm)

HSQC

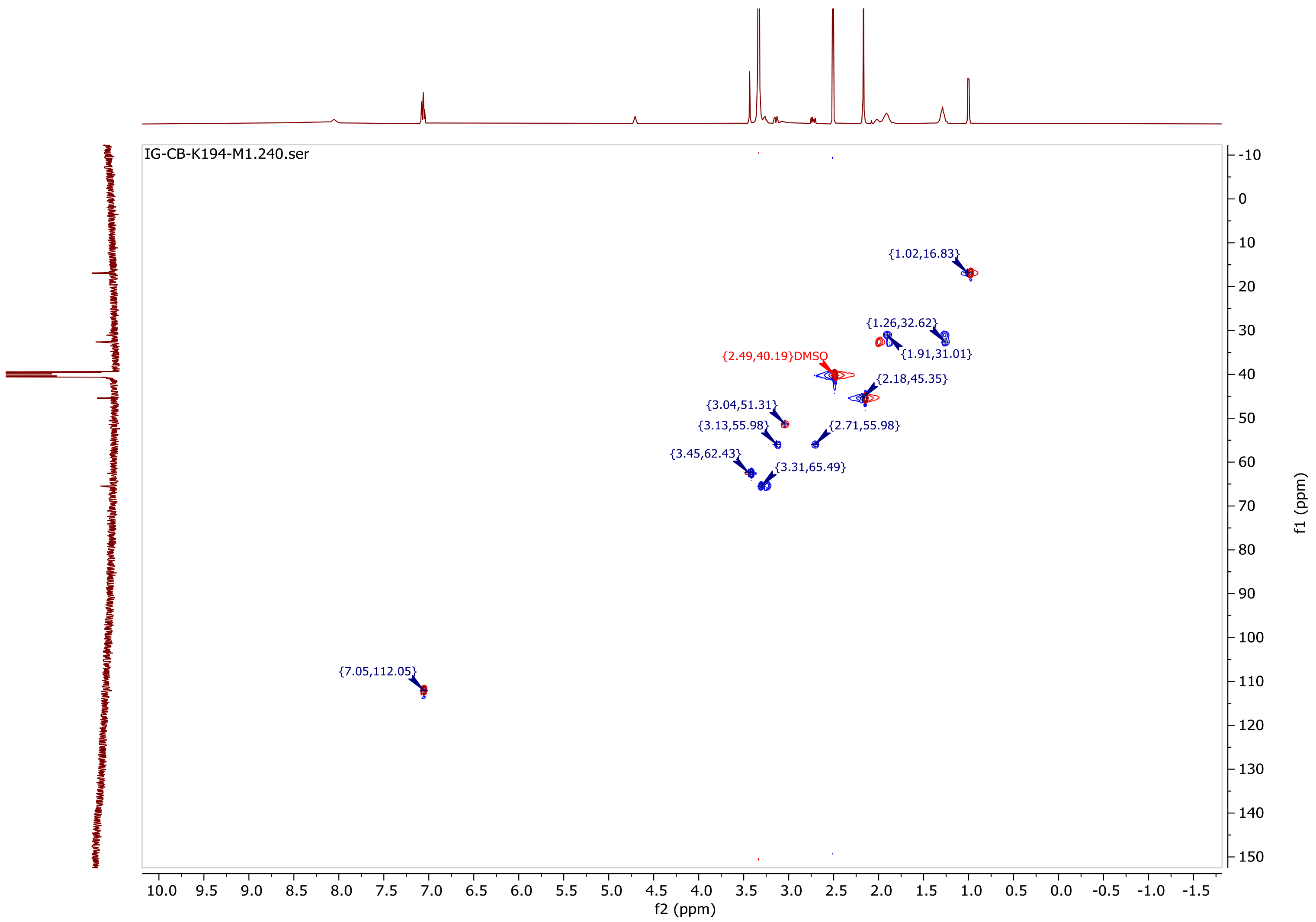
IG-CB-K194-M1.240.ser
{1.02,16.83}
{1.26,32.62}
{1.91,31.01}
{2.49,40.19}DMSO
{2.18,45.35}
{3.04,51.31}
{3.13,55.98}
{2.71,55.98}
{3.45,62.43}
{3.31,65.49}
{7.05,112.05}
f2 (ppm)
f1 (ppm)

HMBC

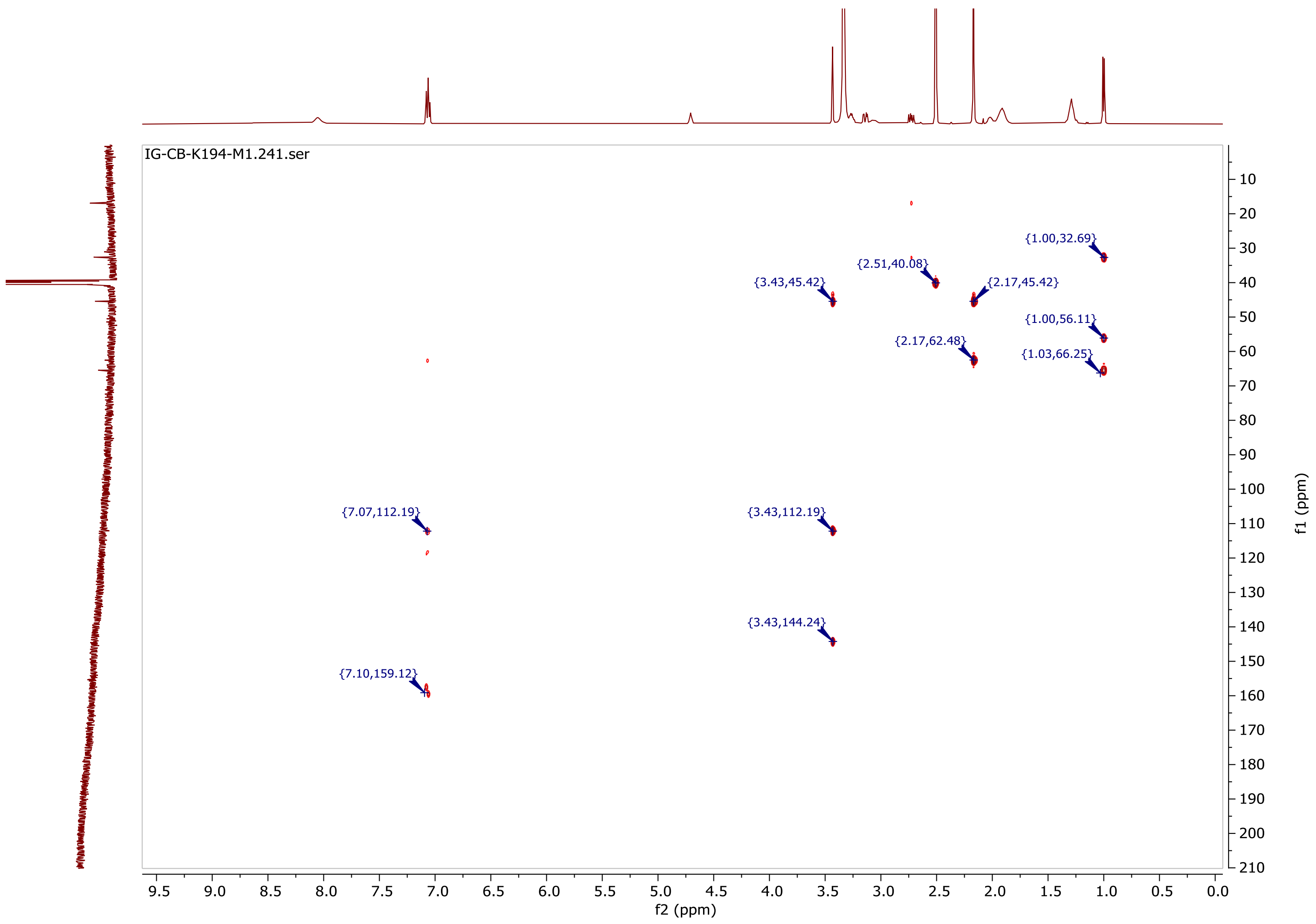

IG-CB-K194-M1.241.ser
{1.00,32.69}
{2.51,40.08}
{3.43,45.42}
{2.17,45.42}
{1.00,56.11}
{2.17,62.48}
{1.03,66.25}
{7.07,112.19}
{3.43,112.19}
{3.43,144.24}
{7.10,159.12}
f2 (ppm)
f1 (ppm)

DEPTqgpsp

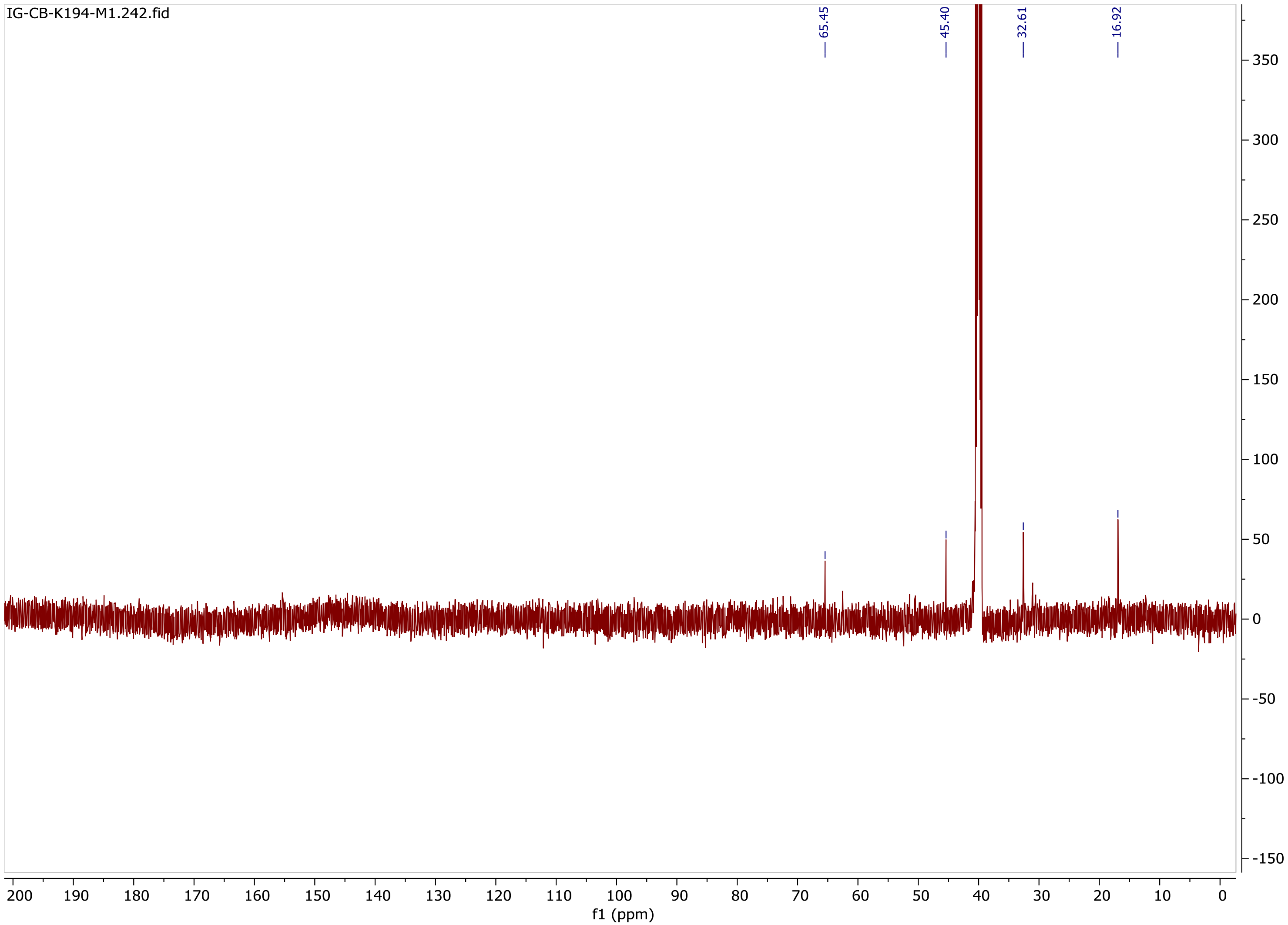

IG-CB-K194-M1.242.fid
65.45
45.40
32.61
16.92
350
300
250
200
150
100
50
0
-50
-100
-150
200
190
180
170
160
150
140
130
120
110
100
90
80
70
60
50
40
30
20
10
0
f1 (ppm)

DEPT-90

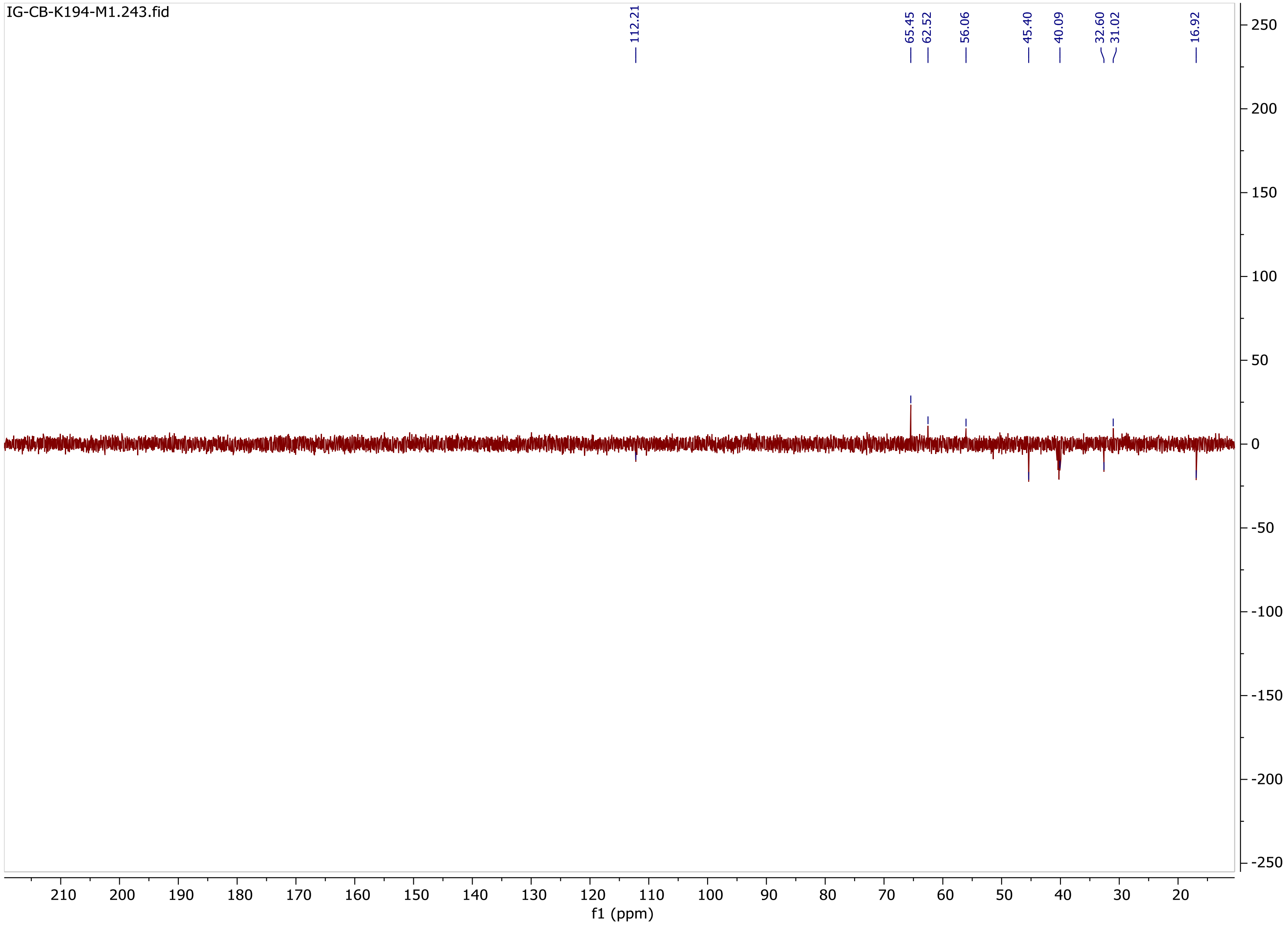

IG-CB-K194-M1.243.fid
112.21
65.45
62.52
56.06
45.40
40.09
32.60
31.02
16.92
250
200
150
100
50
0
-50
-100
-150
-200
-250
210
200
190
180
170
160
150
140
130
120
110
100
90
80
70
60
50
40
30
20
f1 (ppm)

DEPT-135

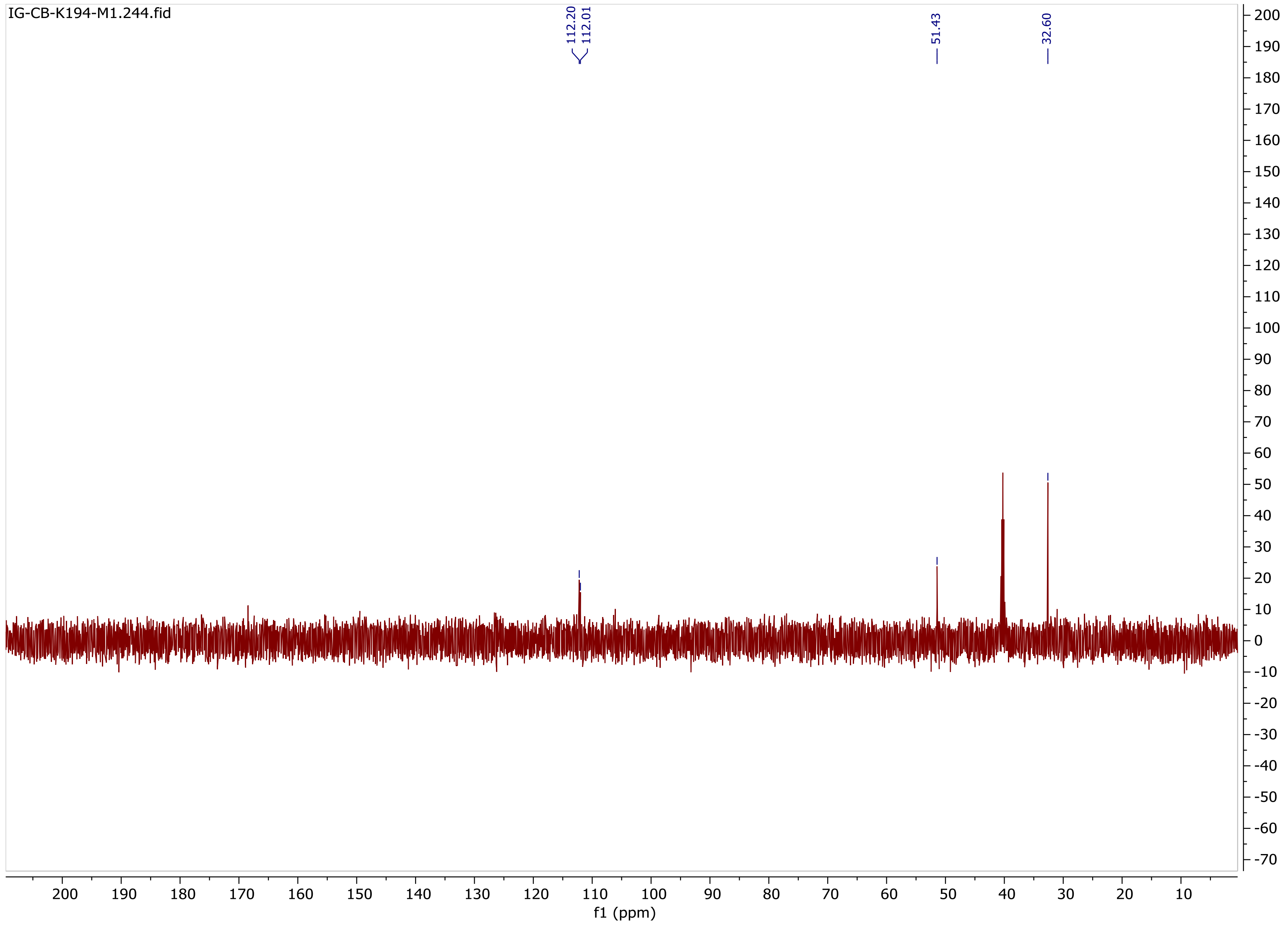

HRMS

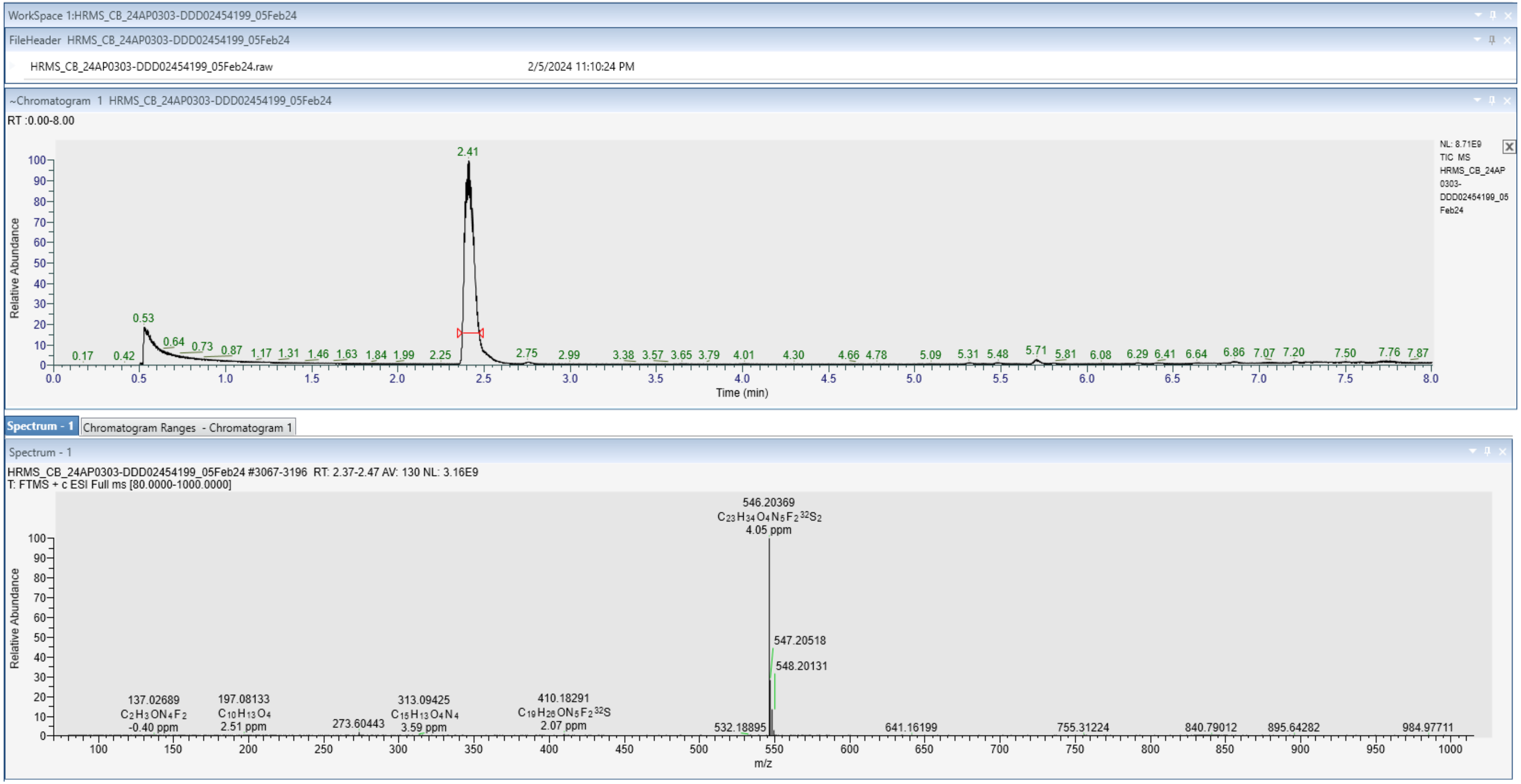
WorkSpace 1:HRMS_CB_24AP0303-DDD02454199_05Feb24
FileHeader HRMS_CB_24AP0303-DDD02454199_05Feb24
HRMS_CB_24AP0303-DDD02454199_05Feb24.raw
2/5/2024 11:10:24 PM
~Chromatogram 1 HRMS_CB_24AP0303-DDD02454199_05Feb24
RT :0.00-8.00
NL: 8.71E9
TIC MS
HRMS_CB_24AP0303-DDD02454199_05Feb24
2.41
0.53
Relative Abundance
Time (min)
Spectrum - 1
Chromatogram Ranges - Chromatogram 1
HRMS_CB_24AP0303-DDD02454199_05Feb24 #3067-3196 RT: 2.37-2.47 AV: 130 NL: 3.16E9
T: FTMS + c ESI Full ms [80.0000-1000.0000]
546.20369
C23H34O4N5F2 32S2
4.05 ppm
547.20518
548.20131
137.02689
C2H3ON4F2
-0.40 ppm
197.08133
C10H13O4
2.51 ppm
273.60443
313.09425
C15H13O4N4
3.59 ppm
410.18291
C19H26ON5F2 32S
2.07 ppm
532.18895
641.16199
755.31224
840.79012
895.64282
984.97711
m/z

Compound **11b**

^{1}H NMR

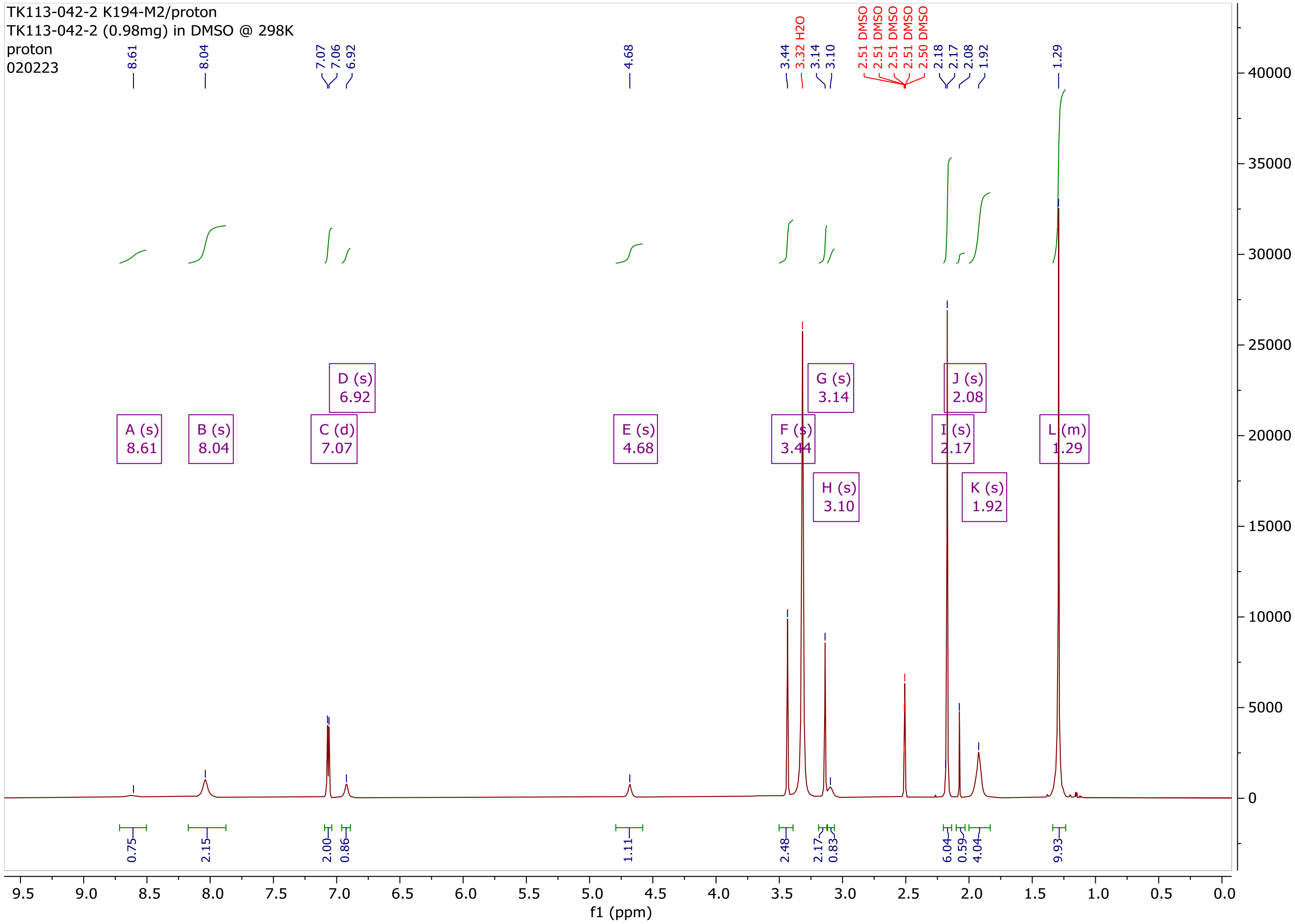

COSY

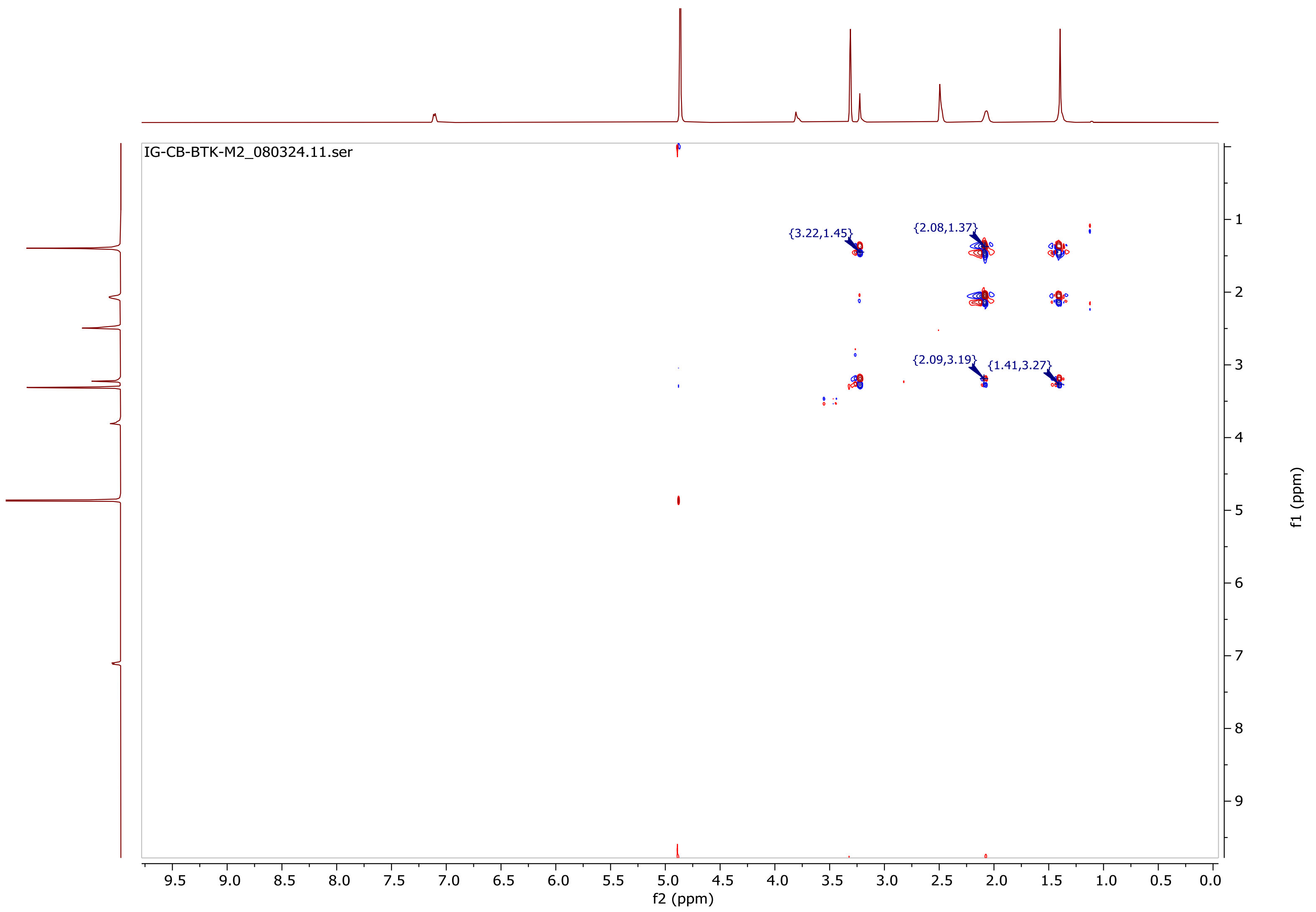

IG-CB-BTK-M2_080324.11.ser
{3.22,1.45}
{2.08,1.37}
{2.09,3.19}
{1.41,3.27}
f2 (ppm)
f1 (ppm)

HSQC

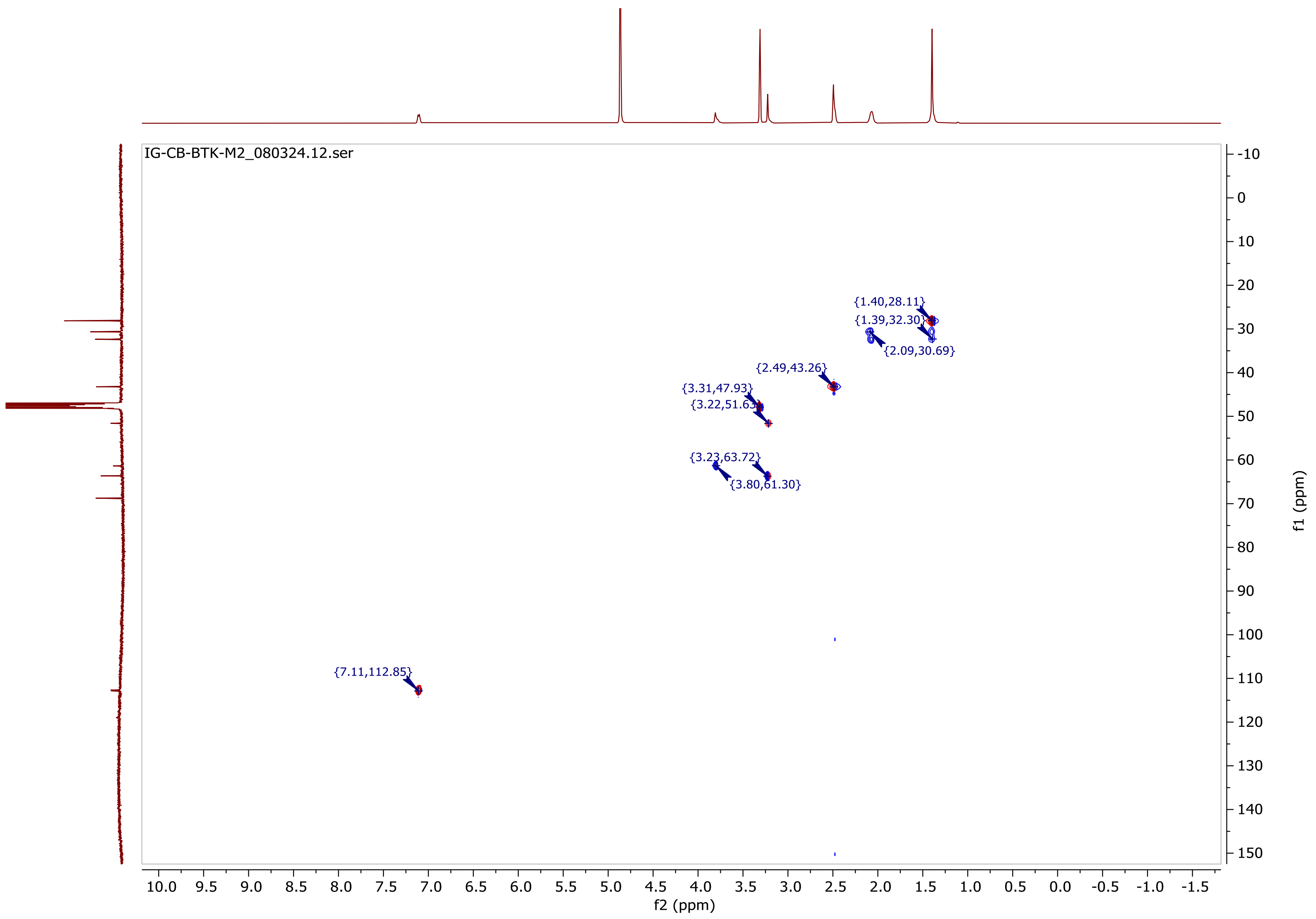
IG-CB-BTK-M2_080324.12.ser
{1.40,28.11}
{1.39,32.30}
{2.09,30.69}
{2.49,43.26}
{3.31,47.93}
{3.22,51.63}
{3.23,63.72}
{3.80,61.30}
{7.11,112.85}
f2 (ppm)
f1 (ppm)

HMBC

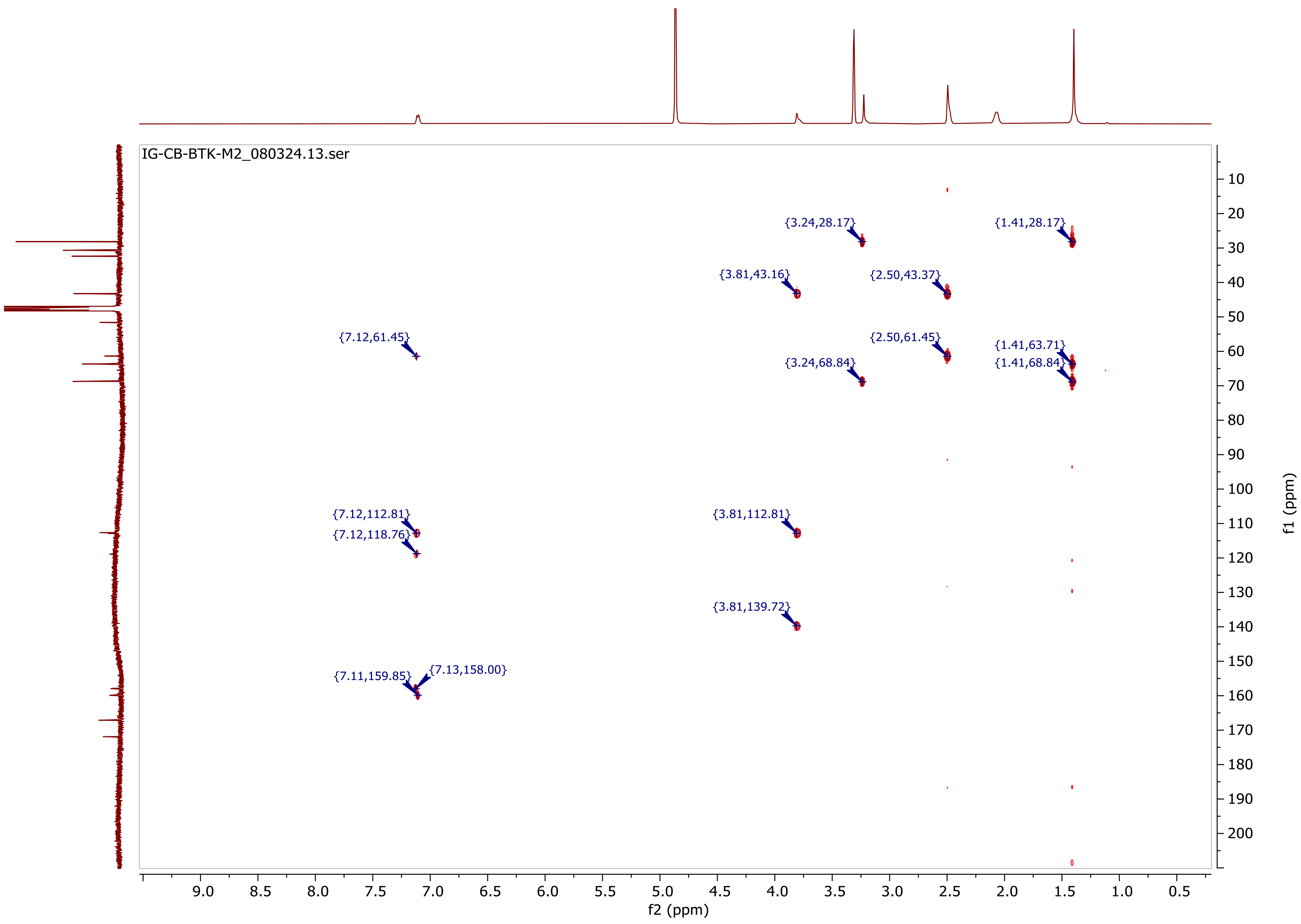
IG-CB-BTK-M2_080324.13.ser
{3.24,28.17}
{1.41,28.17}
{3.81,43.16}
{2.50,43.37}
{7.12,61.45}
{2.50,61.45}
{1.41,63.71}
{3.24,68.84}
{1.41,68.84}
{7.12,112.81}
{3.81,112.81}
{7.12,118.76}
{3.81,139.72}
{7.11,159.85}
{7.13,158.00}
f2 (ppm)
f1 (ppm)

13C

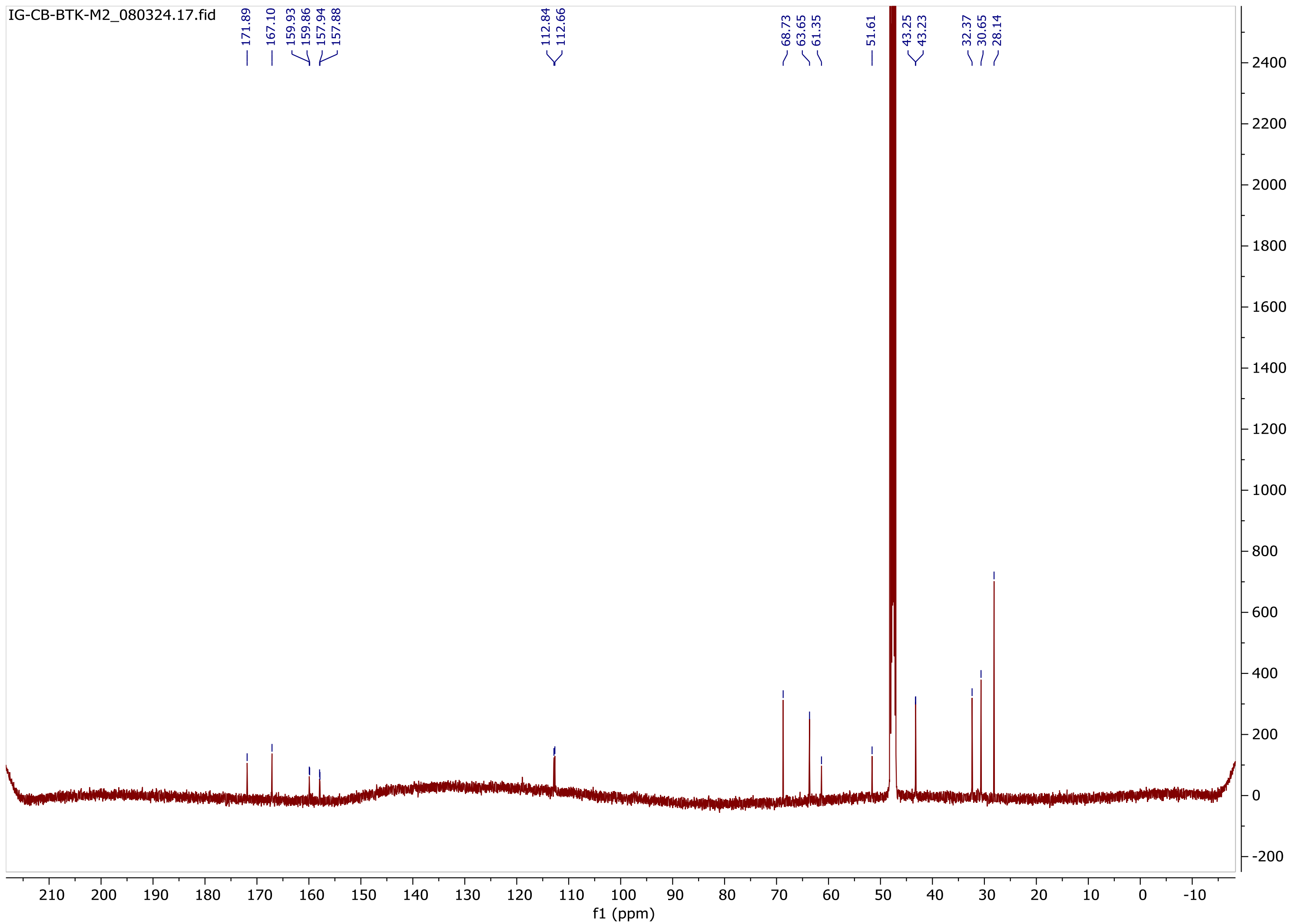
IG-CB-BTK-M2_080324.17.fid
171.89
167.10
159.93
159.86
157.94
157.88
112.84
112.66
68.73
63.65
61.35
51.61
43.25
43.23
32.37
30.65
28.14
f1 (ppm)

DEPTqgpsp

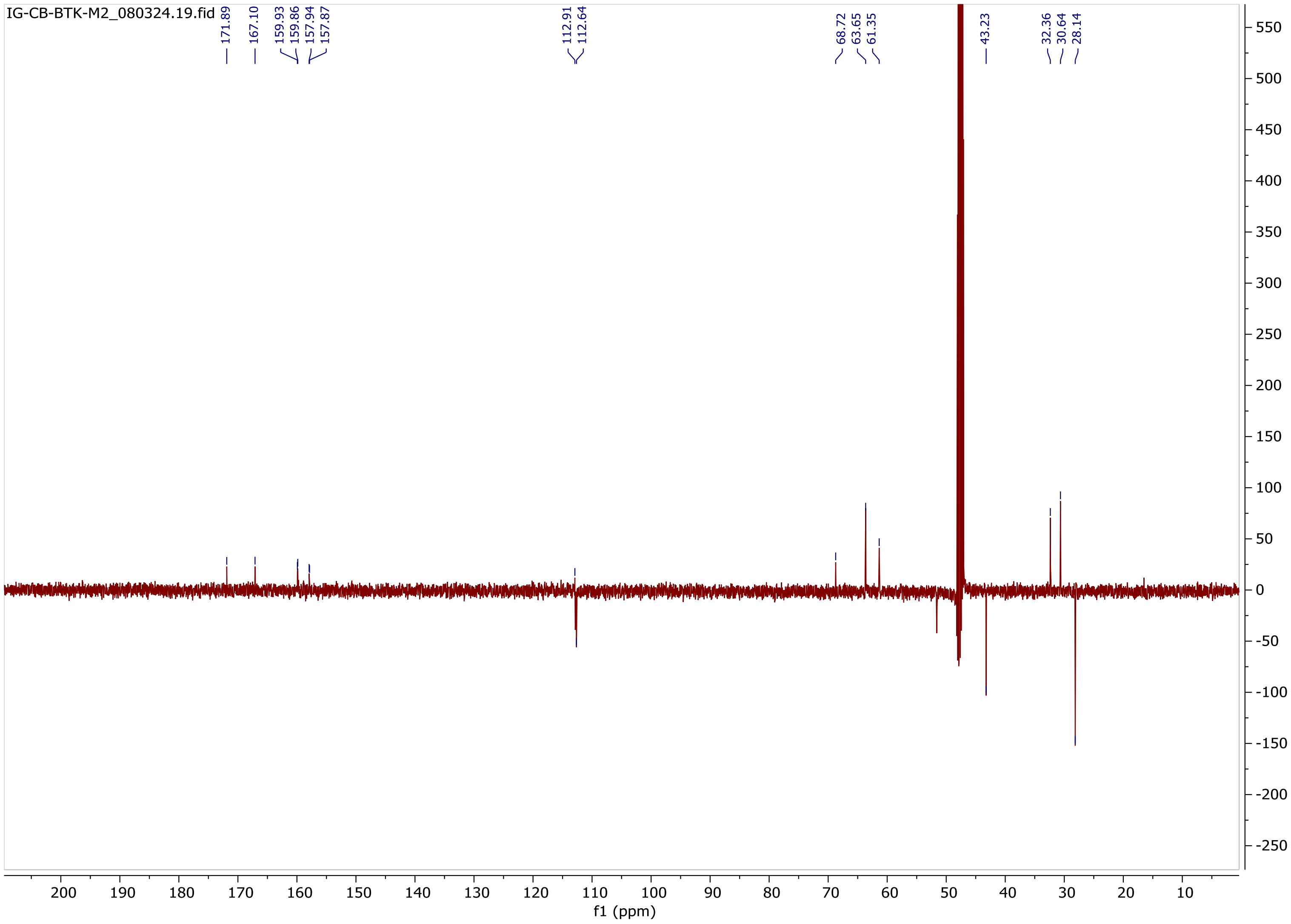
IG-CB-BTK-M2_080324.19.fid
171.89
167.10
159.93
159.86
157.94
157.87
112.91
112.64
68.72
63.65
61.35
43.23
32.36
30.64
28.14
f1 (ppm)

DEPT-90

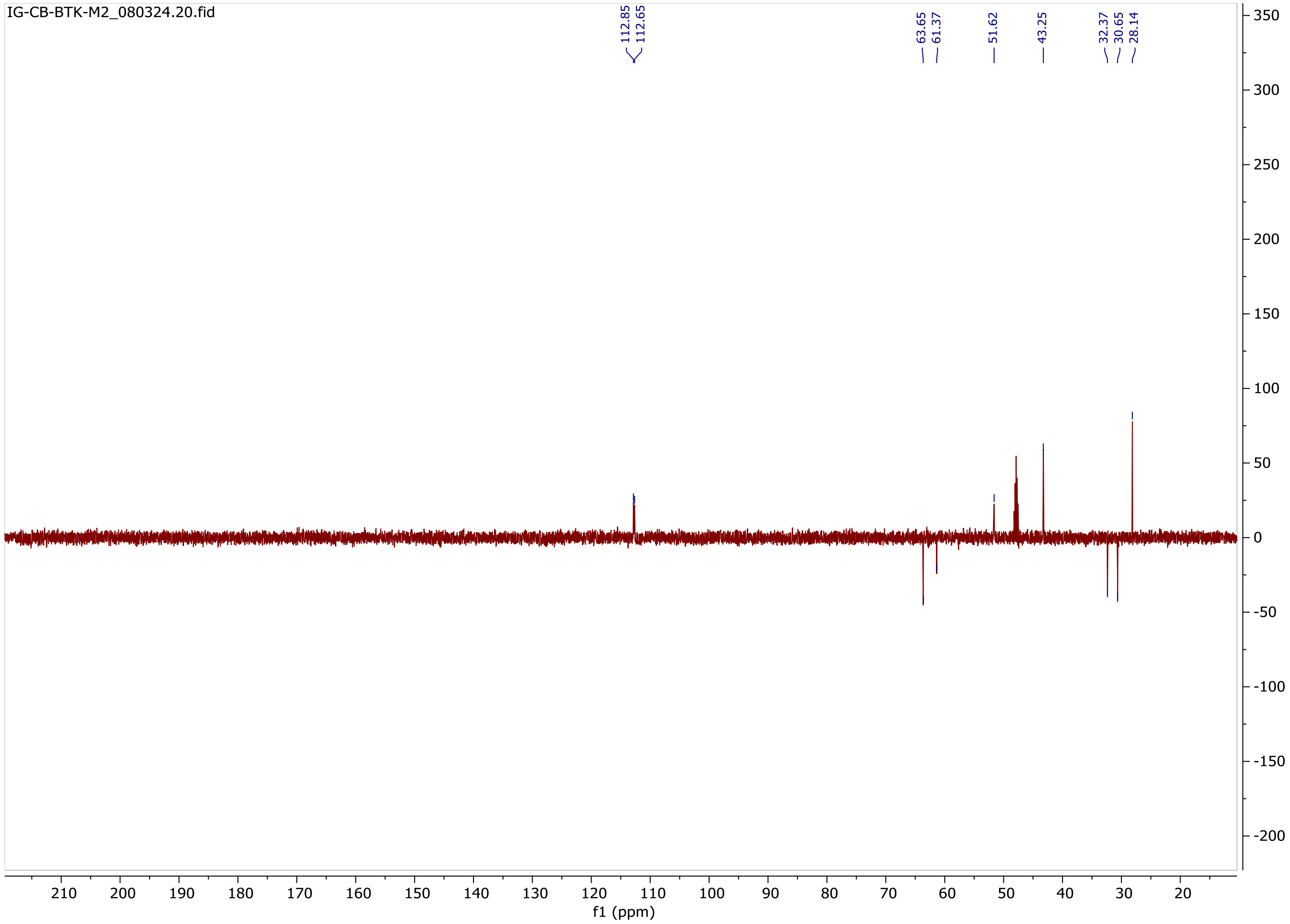

DEPT-135

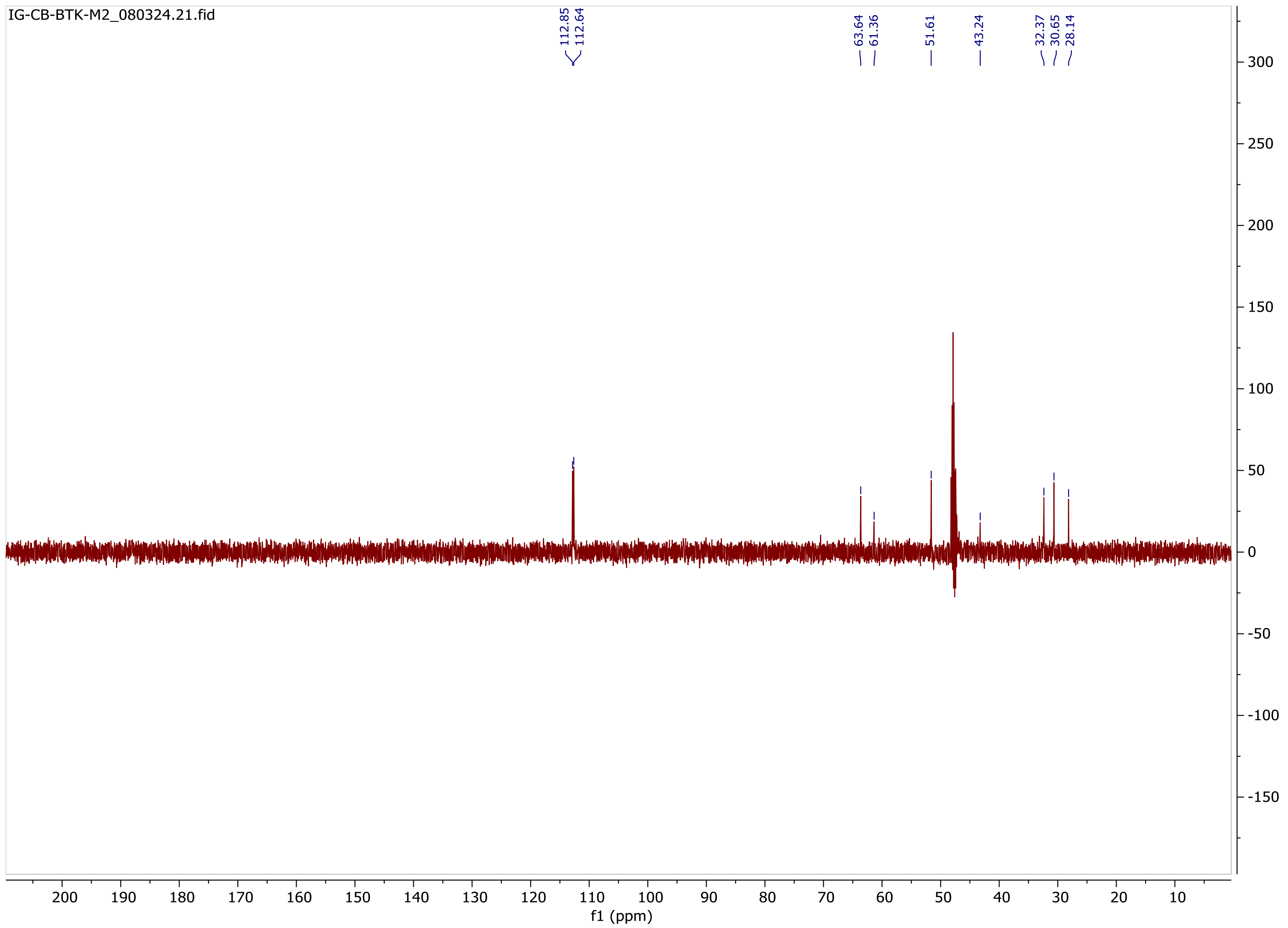

IG-CB-BTK-M2_080324.21.fid
112.85
112.64
63.64
61.36
51.61
43.24
32.37
30.65
28.14
f1 (ppm)

HRMS

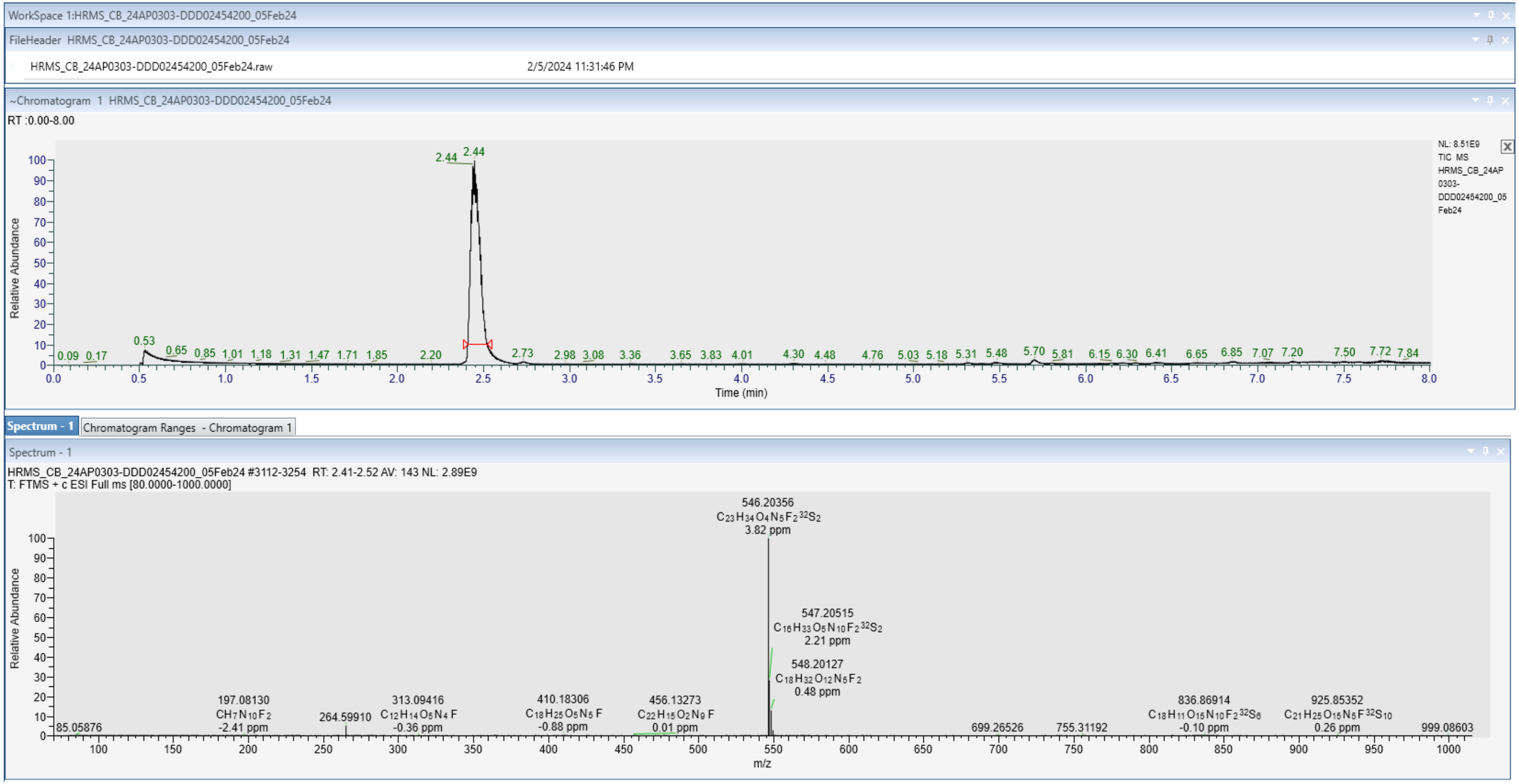
WorkSpace 1:HRMS_CB_24AP0303-DDD02454200_05Feb24
FileHeader HRMS_CB_24AP0303-DDD02454200_05Feb24
HRMS_CB_24AP0303-DDD02454200_05Feb24.raw
2/5/2024 11:31:46 PM
~Chromatogram 1 HRMS_CB_24AP0303-DDD02454200_05Feb24
RT :0.00-8.00
NL: 8.51E9
TIC MS
HRMS_CB_24AP0303-DDD02454200_05Feb24
Relative Abundance
Time (min)
2.44
0.53
Spectrum - 1
Chromatogram Ranges - Chromatogram 1
HRMS_CB_24AP0303-DDD02454200_05Feb24 #3112-3254 RT: 2.41-2.52 AV: 143 NL: 2.89E9
T: FTMS + c ESI Full ms [80.0000-1000.0000]
546.20356
C23 H34 O4 N5 F2 32S2
3.82 ppm
547.20515
C16 H33 O5 N10 F2 32S2
2.21 ppm
548.20127
C18 H32 O12 N5 F2
0.48 ppm
85.05876
197.08130
CH7 N10 F2
-2.41 ppm
264.59910
313.09416
C12 H14 O5 N4 F
-0.36 ppm
410.18306
C18 H25 O5 N5 F
-0.88 ppm
456.13273
C22 H15 O2 N9 F
0.01 ppm
699.26526
755.31192
836.86914
C18 H11 O15 N10 F2 32S6
-0.10 ppm
925.85352
C21 H25 O15 N5 F 32S10
0.26 ppm
999.08603
m/z

Compound **12a**

^{1}H NMR

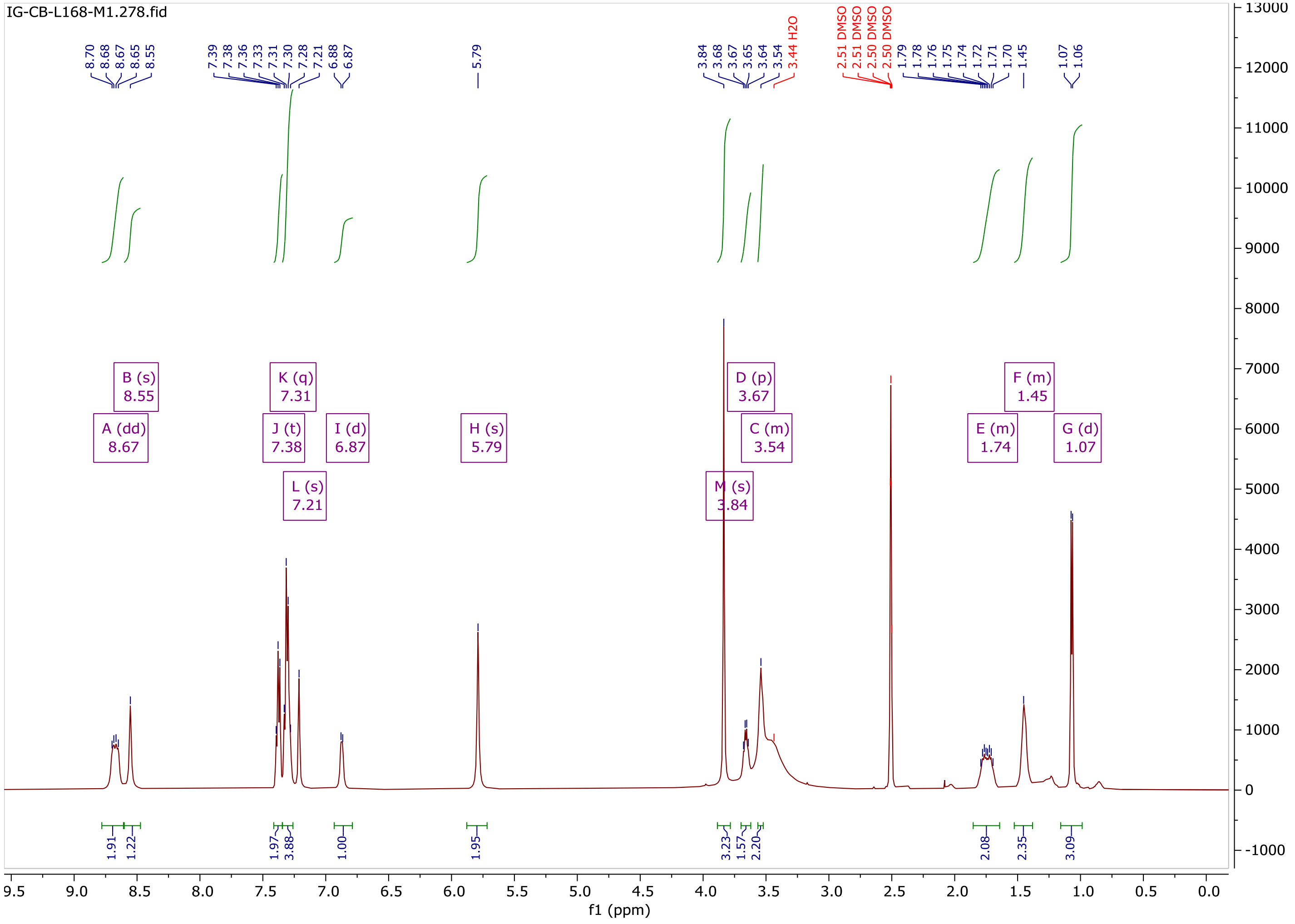

COSY

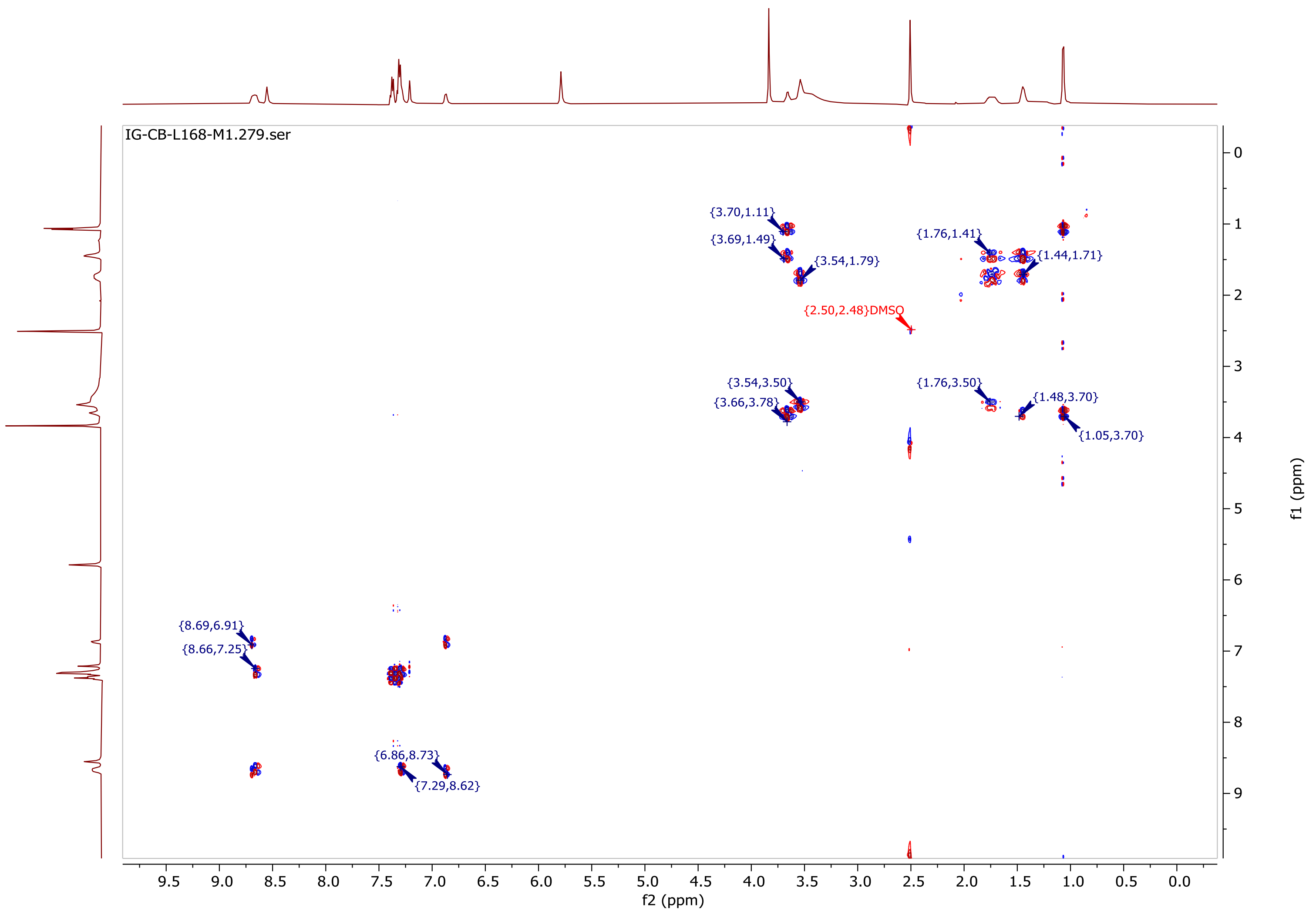
IG-CB-L168-M1.279.ser
{3.70,1.11}
{3.69,1.49}
{3.54,1.79}
{1.76,1.41}
{1.44,1.71}
{2.50,2.48}DMSO
{3.54,3.50}
{3.66,3.78}
{1.76,3.50}
{1.48,3.70}
{1.05,3.70}
{8.69,6.91}
{8.66,7.25}
{6.86,8.73}
{7.29,8.62}
f2 (ppm)
f1 (ppm)

HSQC

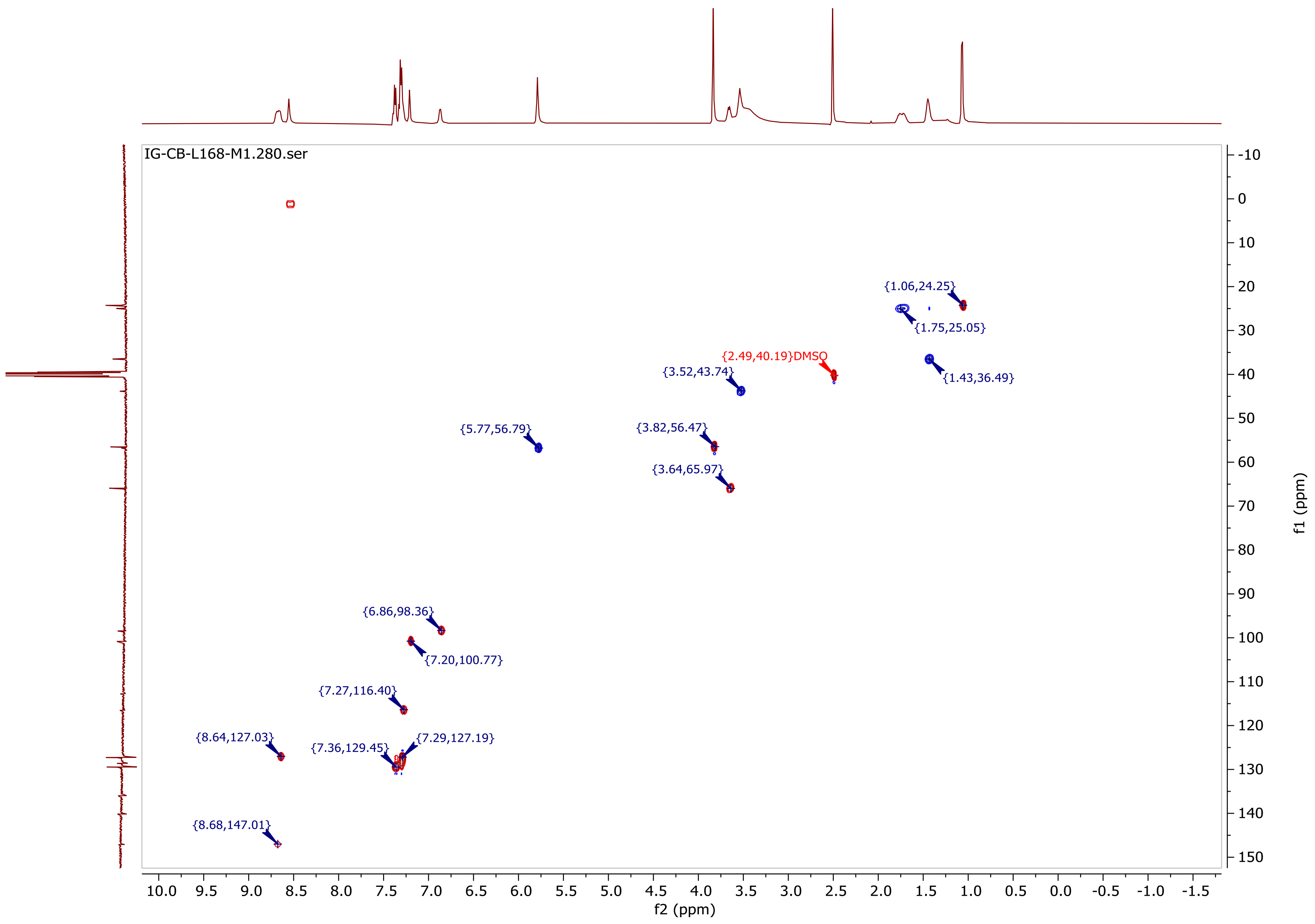
IG-CB-L168-M1.280.ser
{1.06,24.25}
{1.75,25.05}
{2.49,40.19}DMSO
{3.52,43.74}
{1.43,36.49}
{5.77,56.79}
{3.82,56.47}
{3.64,65.97}
{6.86,98.36}
{7.20,100.77}
{7.27,116.40}
{8.64,127.03}
{7.36,129.45}
{7.29,127.19}
{8.68,147.01}
f2 (ppm)
f1 (ppm)

HMBC

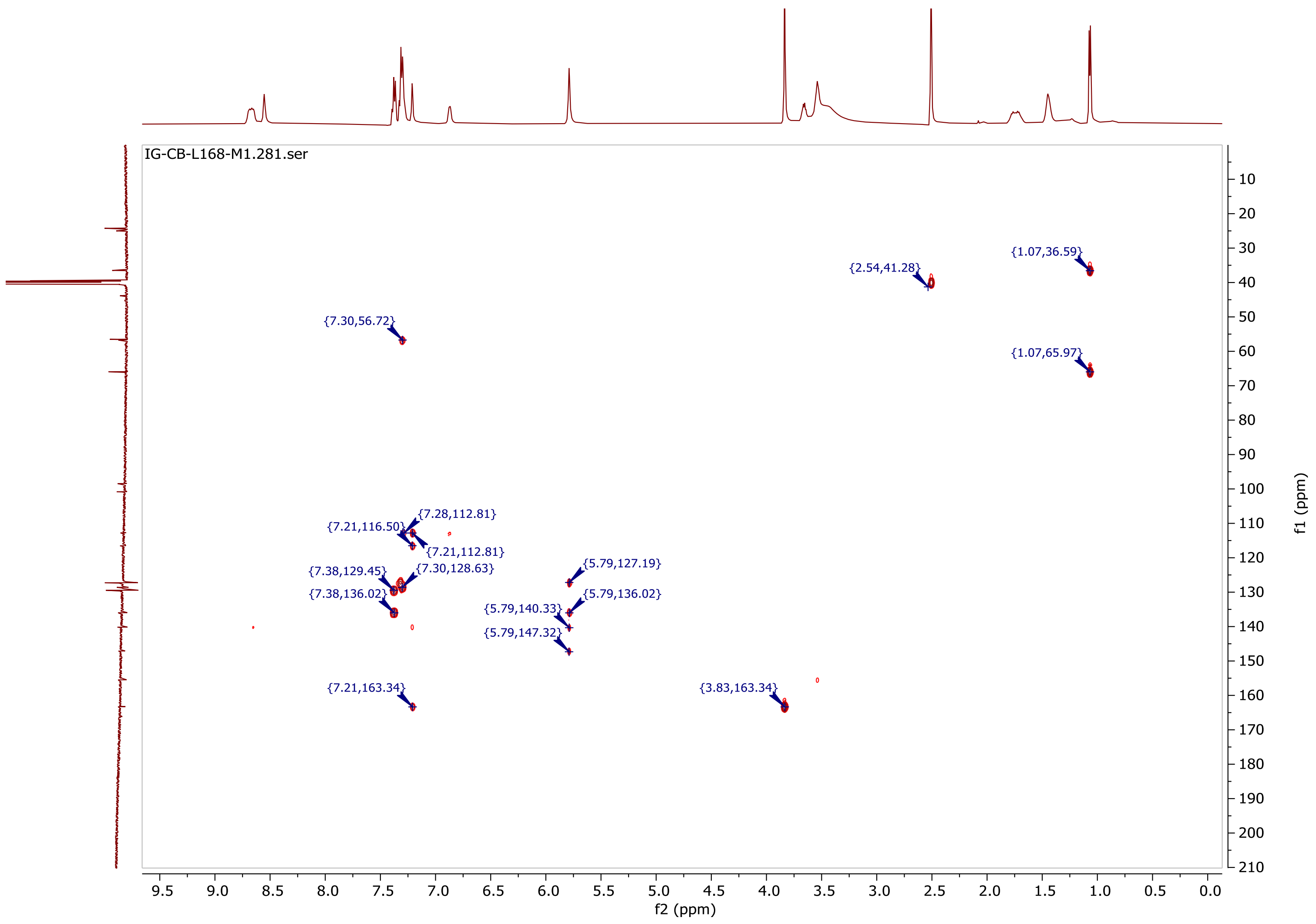

IG-CB-L168-M1.281.ser
{2.54,41.28}
{1.07,36.59}
{7.30,56.72}
{1.07,65.97}
{7.28,112.81}
{7.21,116.50}
{7.21,112.81}
{7.38,129.45}
{7.30,128.63}
{5.79,127.19}
{7.38,136.02}
{5.79,136.02}
{5.79,140.33}
{5.79,147.32}
{7.21,163.34}
{3.83,163.34}
f2 (ppm)
f1 (ppm)

DEPTqgpsp

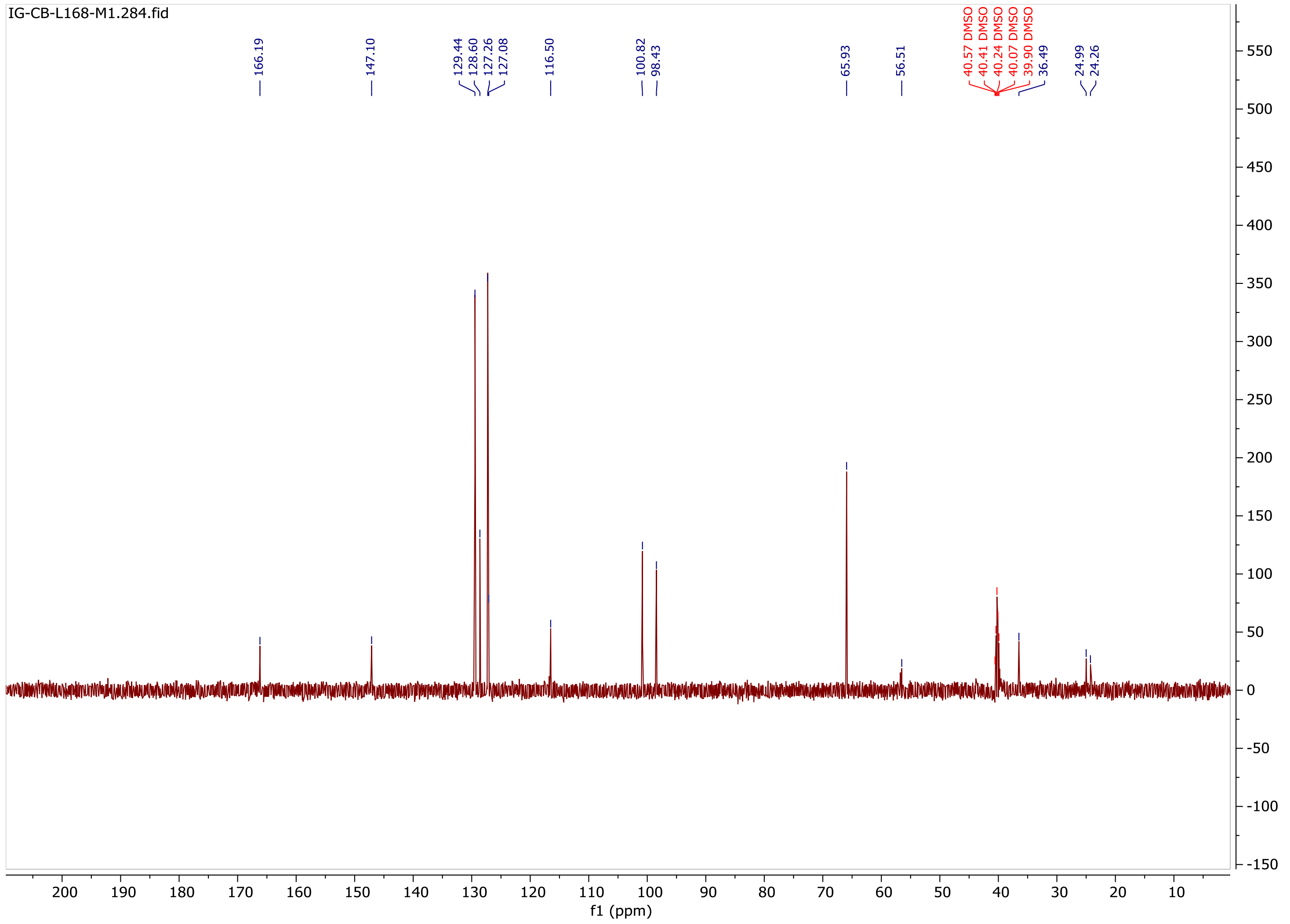

IG-CB-L168-M1.284.fid
166.19
147.10
129.44
128.60
127.26
127.08
116.50
100.82
98.43
65.93
56.51
40.57 DMSO
40.41 DMSO
40.24 DMSO
40.07 DMSO
39.90 DMSO
36.49
24.99
24.26
f1 (ppm)

Zgpg30

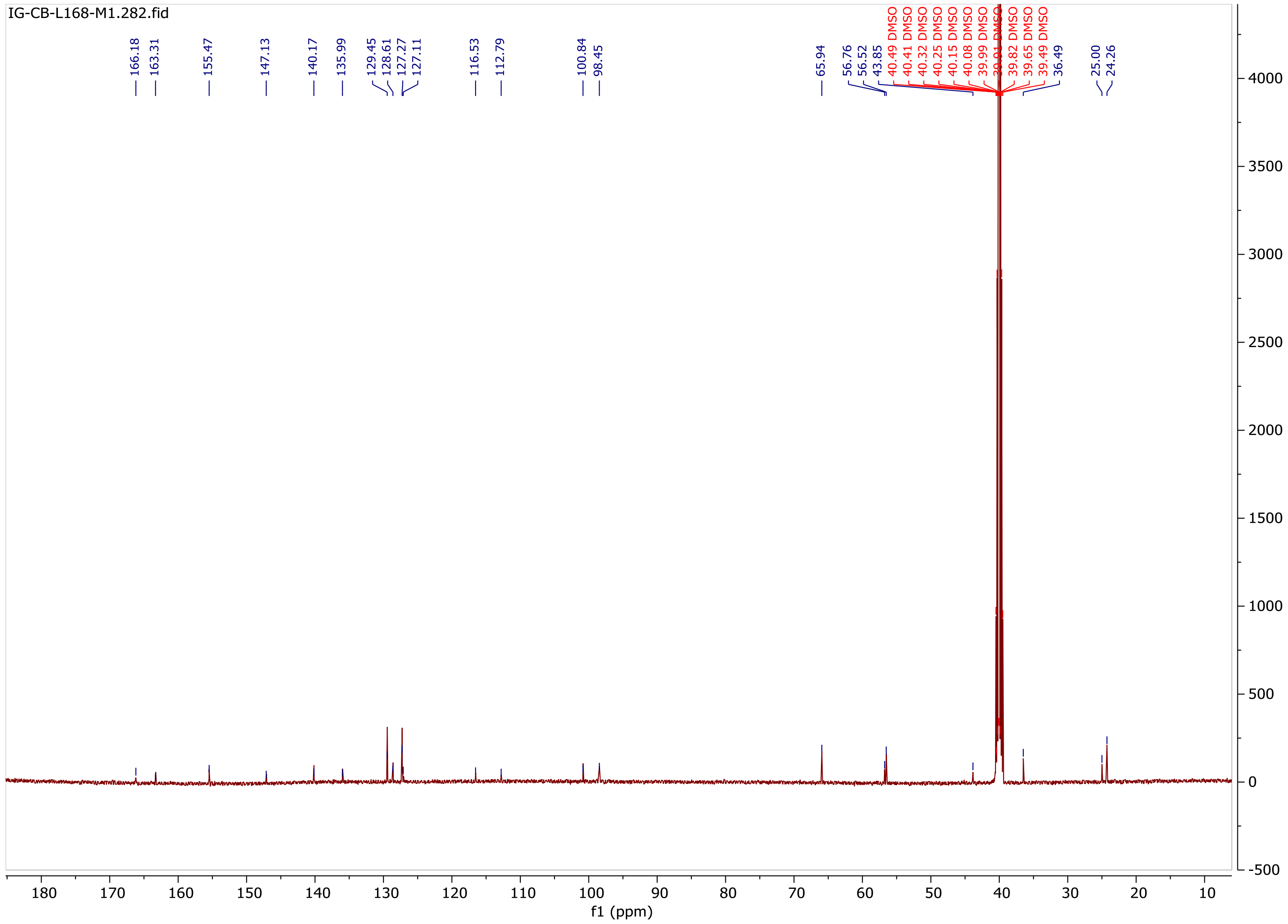

IG-CB-L168-M1.282.fid
166.18
163.31
155.47
147.13
140.17
135.99
129.45
128.61
127.27
127.11
116.53
112.79
100.84
98.45
65.94
56.76
56.52
43.85
40.49 DMSO
40.41 DMSO
40.32 DMSO
40.25 DMSO
40.15 DMSO
40.08 DMSO
39.99 DMSO
39.91 DMSO
39.82 DMSO
39.65 DMSO
39.49 DMSO
36.49
25.00
24.26
f1 (ppm)

DEPT-135

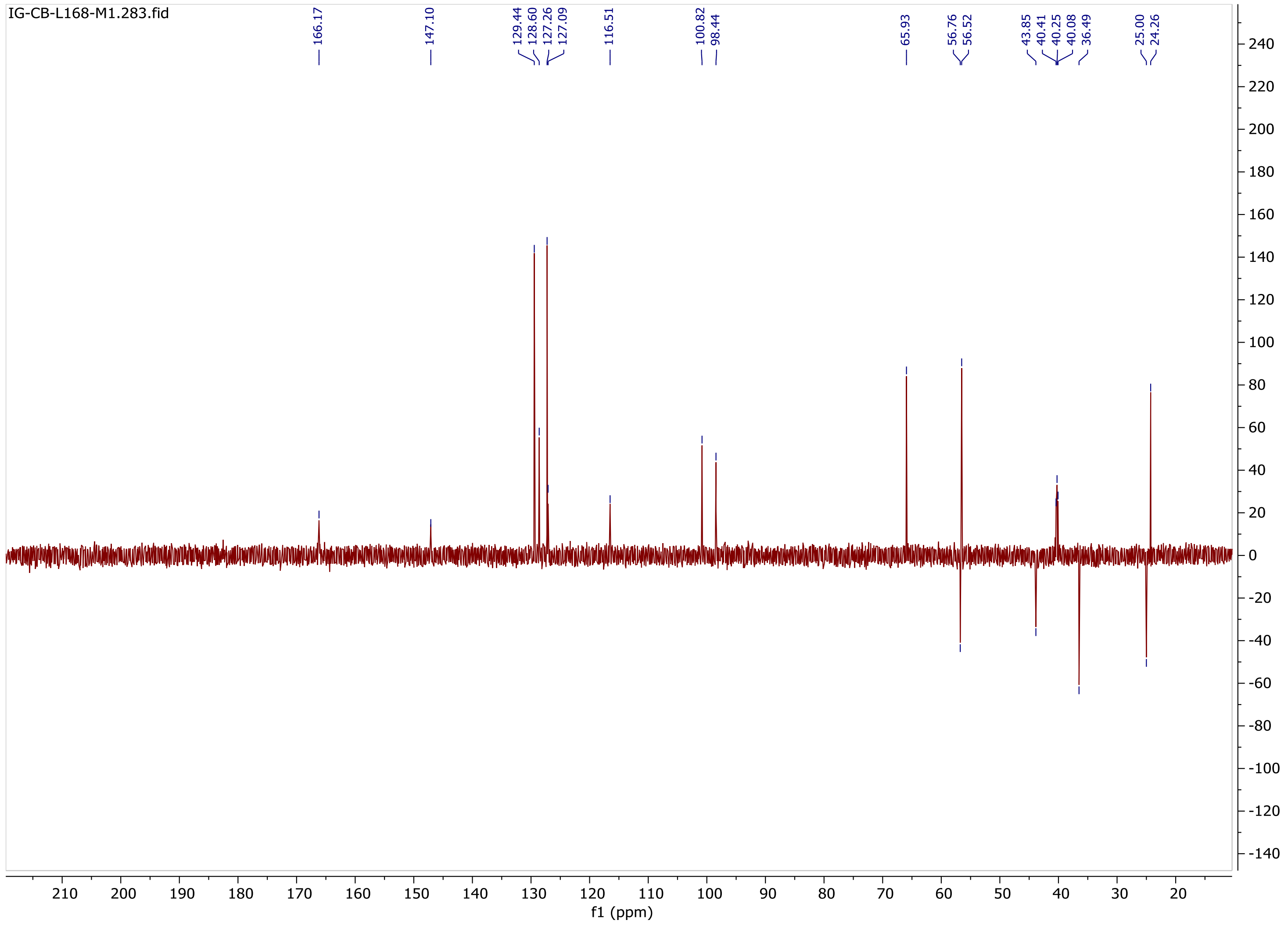

IG-CB-L168-M1.283.fid
166.17
147.10
129.44
128.60
127.26
127.09
116.51
100.82
98.44
65.93
56.76
56.52
43.85
40.41
40.25
40.08
36.49
25.00
24.26
f1 (ppm)

HRMS

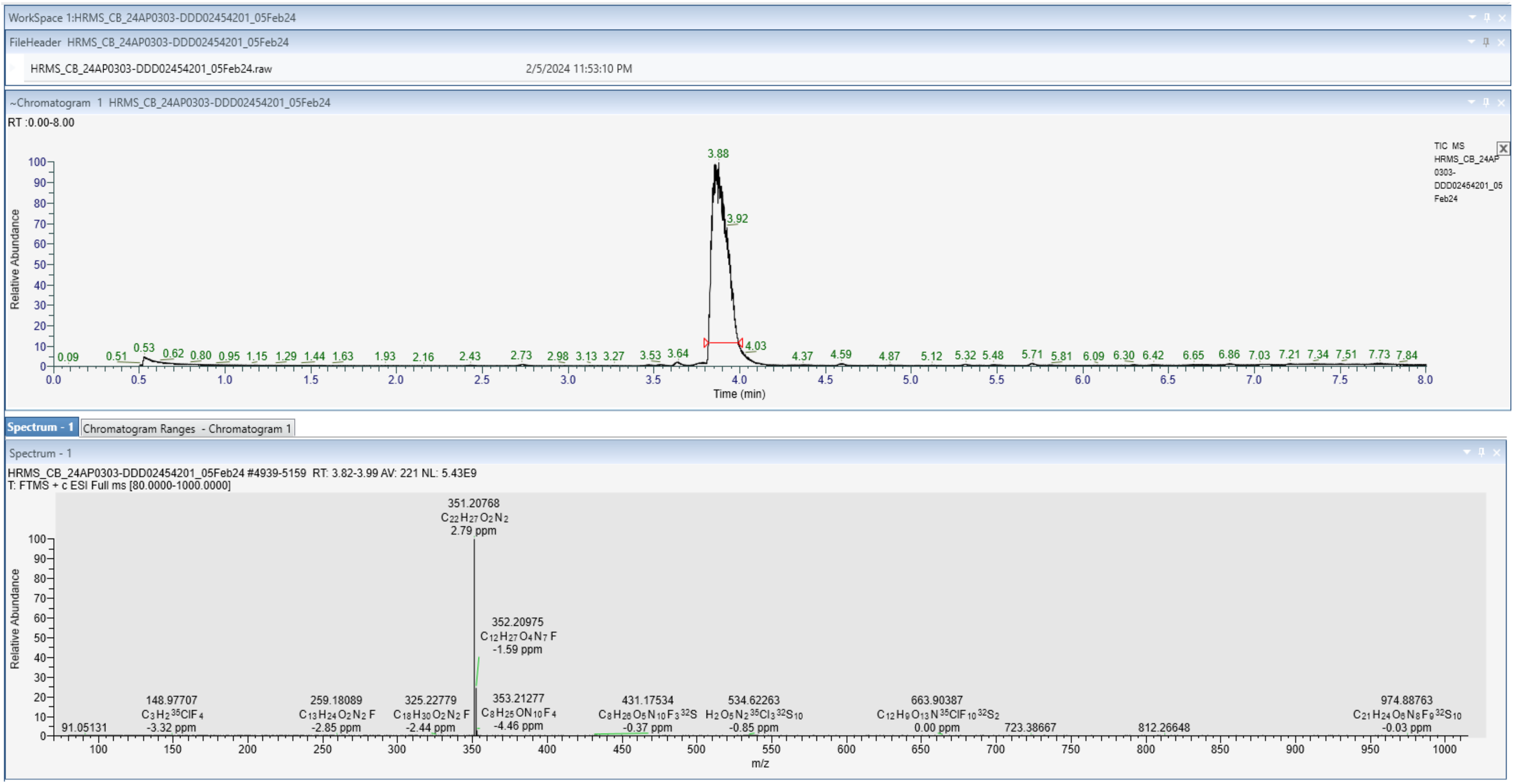
WorkSpace 1:HRMS_CB_24AP0303-DDD02454201_05Feb24
FileHeader HRMS_CB_24AP0303-DDD02454201_05Feb24
HRMS_CB_24AP0303-DDD02454201_05Feb24.raw
2/5/2024 11:53:10 PM
~Chromatogram 1 HRMS_CB_24AP0303-DDD02454201_05Feb24
RT :0.00-8.00
3.88
3.92
4.03
TIC MS HRMS_CB_24AP0303-DDD02454201_05Feb24
Relative Abundance
Time (min)
Spectrum - 1
Chromatogram Ranges - Chromatogram 1
HRMS_CB_24AP0303-DDD02454201_05Feb24 #4939-5159 RT: 3.82-3.99 AV: 221 NL: 5.43E9
T: FTMS + c ESI Full ms [80.0000-1000.0000]
351.20768
C22 H27 O2 N2
2.79 ppm
352.20975
C12 H27 O4 N7 F
-1.59 ppm
353.21277
C8 H25 ON10 F4
-4.46 ppm
91.05131
148.97707
C3 H2 35ClF4
-3.32 ppm
259.18089
C13 H24 O2 N2 F
-2.85 ppm
325.22779
C18 H30 O2 N2 F
-2.44 ppm
431.17534
C8 H26 O5 N10 F3 32S
-0.37 ppm
534.62263
H2 O5 N2 35Cl3 32S10
-0.85 ppm
663.90387
C12 H9 O13 N 35ClF10 32S2
0.00 ppm
723.38667
812.26648
974.88763
C21 H24 O8 N8 F9 32S10
-0.03 ppm
m/z

Compound **12b**

^{1}H NMR

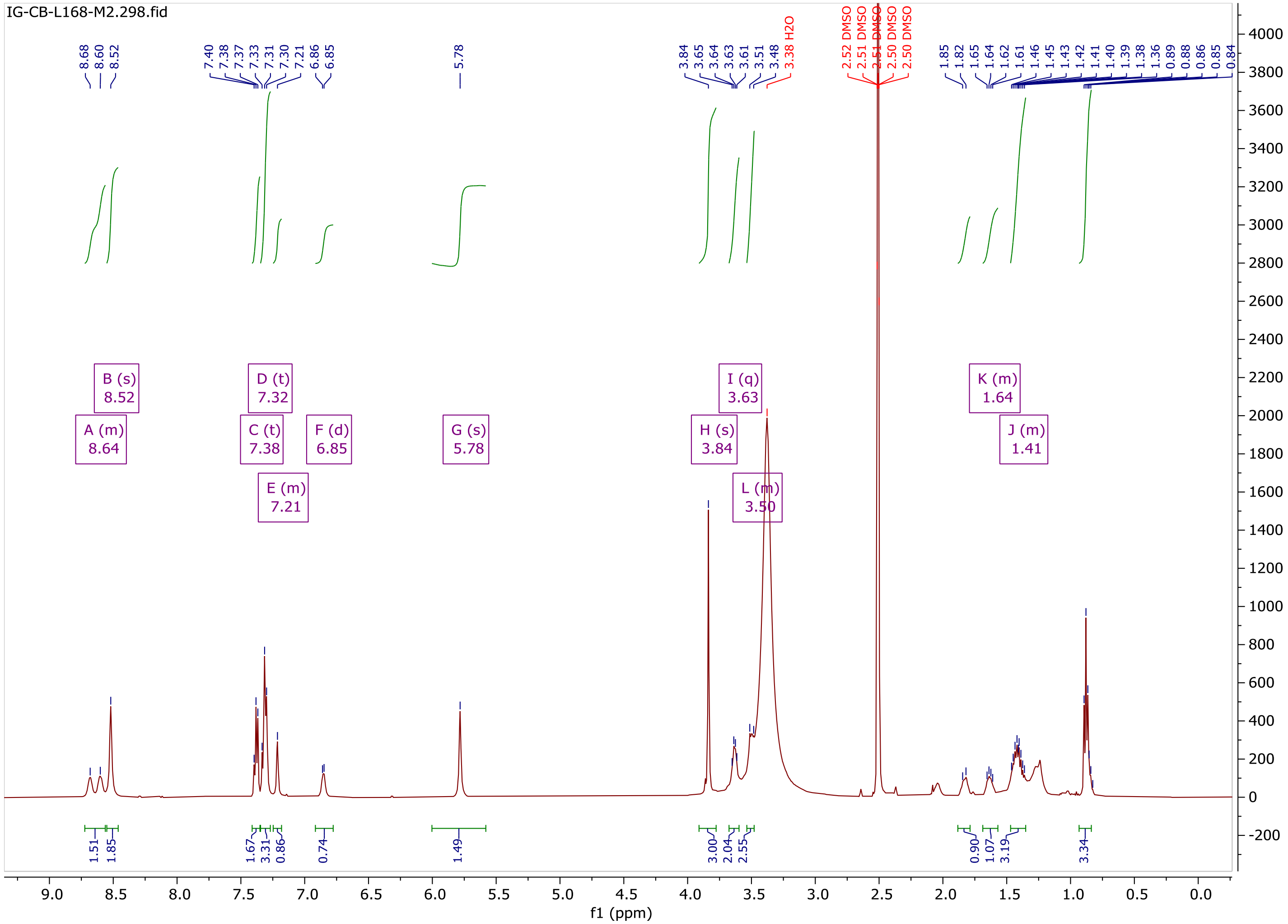

COSY

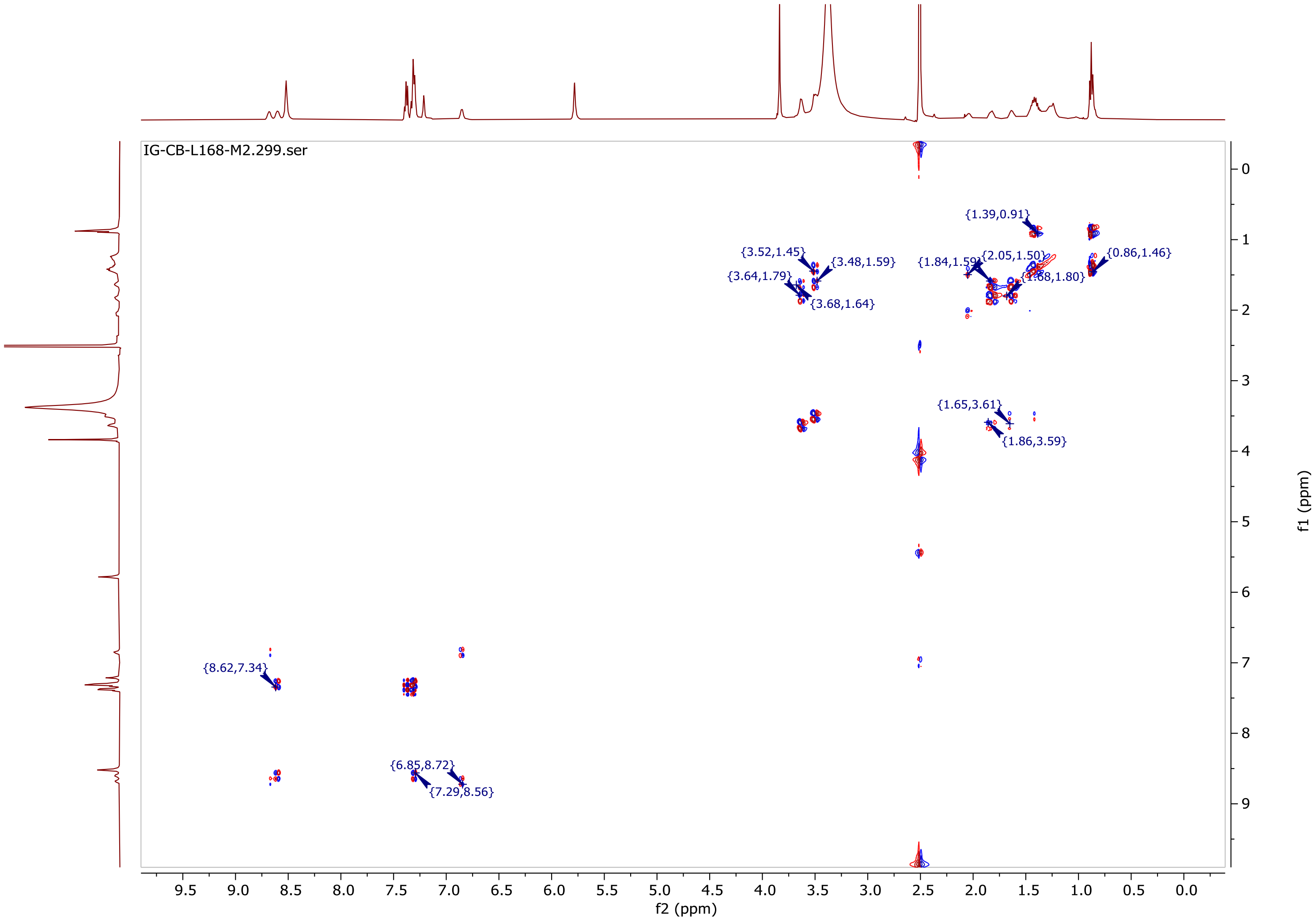
IG-CB-L168-M2.299.ser
{1.39,0.91}
{3.52,1.45}
{3.48,1.59}
{3.64,1.79}
{3.68,1.64}
{1.84,1.59}
{2.05,1.50}
{1.68,1.80}
{0.86,1.46}
{1.65,3.61}
{1.86,3.59}
{8.62,7.34}
{6.85,8.72}
{7.29,8.56}
f2 (ppm)
f1 (ppm)

HSQC

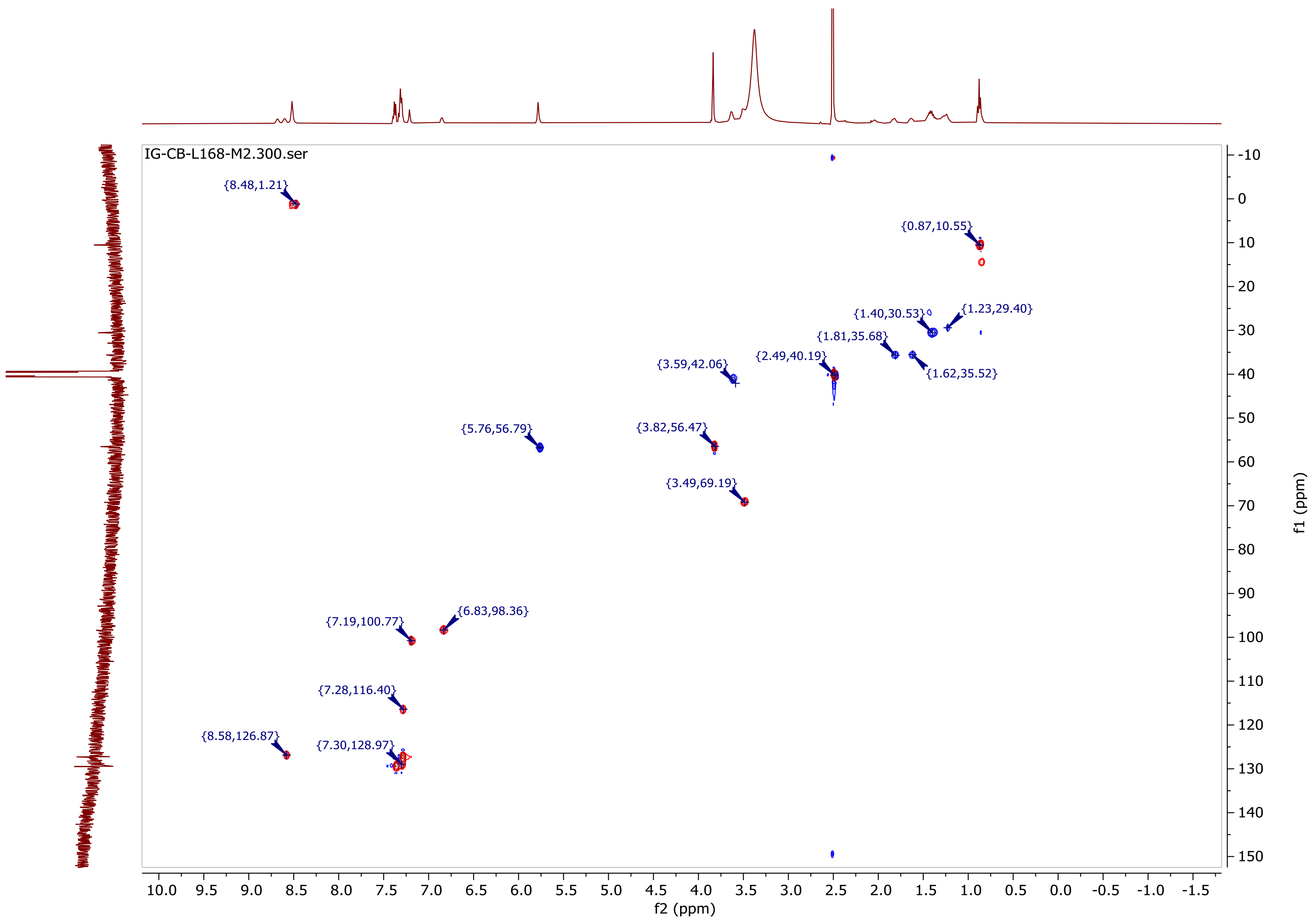
IG-CB-L168-M2.300.ser
{8.48,1.21}
{0.87,10.55}
{1.40,30.53}
{1.23,29.40}
{1.81,35.68}
{1.62,35.52}
{2.49,40.19}
{3.59,42.06}
{5.76,56.79}
{3.82,56.47}
{3.49,69.19}
{6.83,98.36}
{7.19,100.77}
{7.28,116.40}
{8.58,126.87}
{7.30,128.97}
f2 (ppm)
f1 (ppm)

HMBC

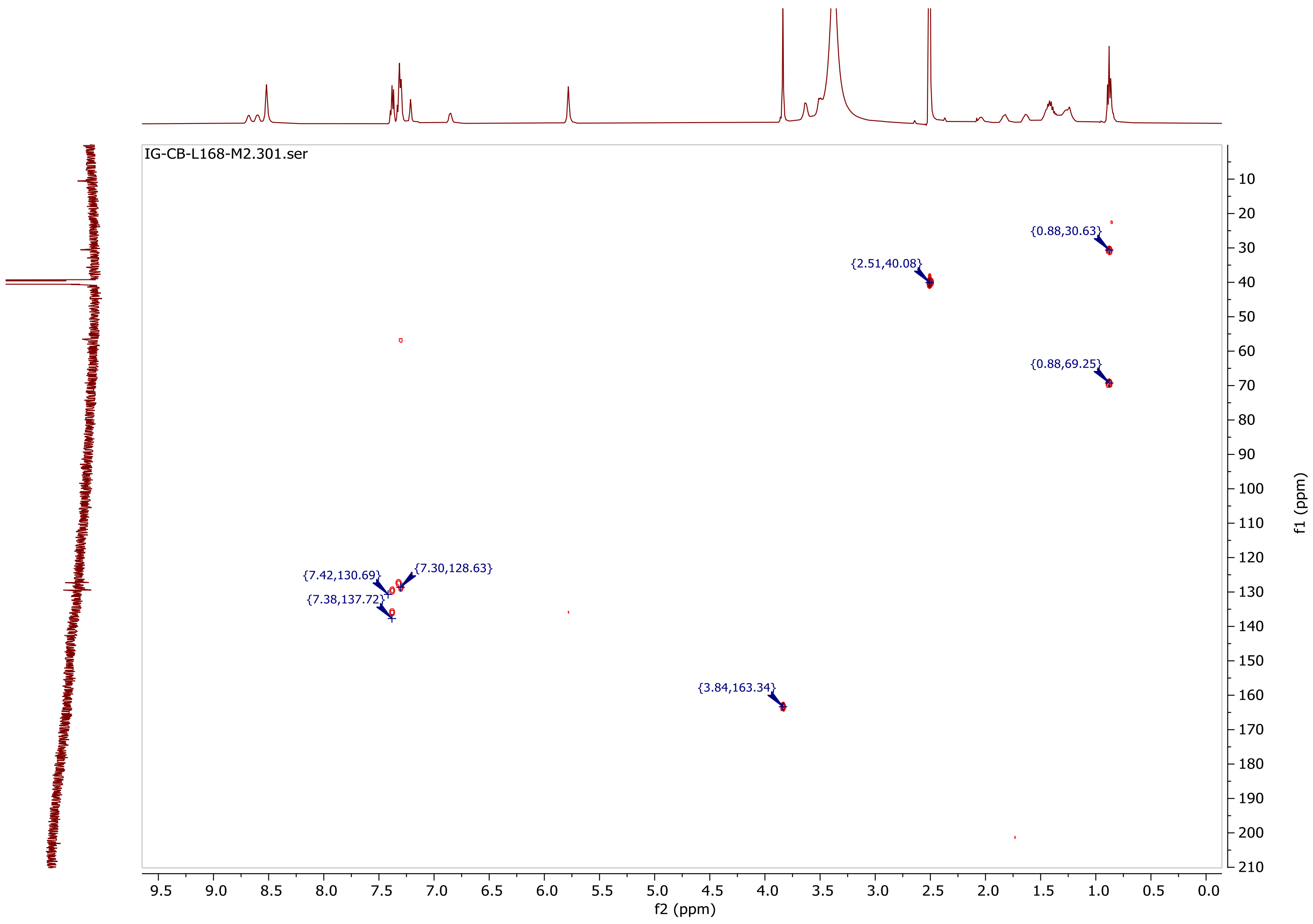

IG-CB-L168-M2.301.ser
{0.88,30.63}
{2.51,40.08}
{0.88,69.25}
{7.42,130.69}
{7.30,128.63}
{7.38,137.72}
{3.84,163.34}
f2 (ppm)
f1 (ppm)

DEPTqgpsp

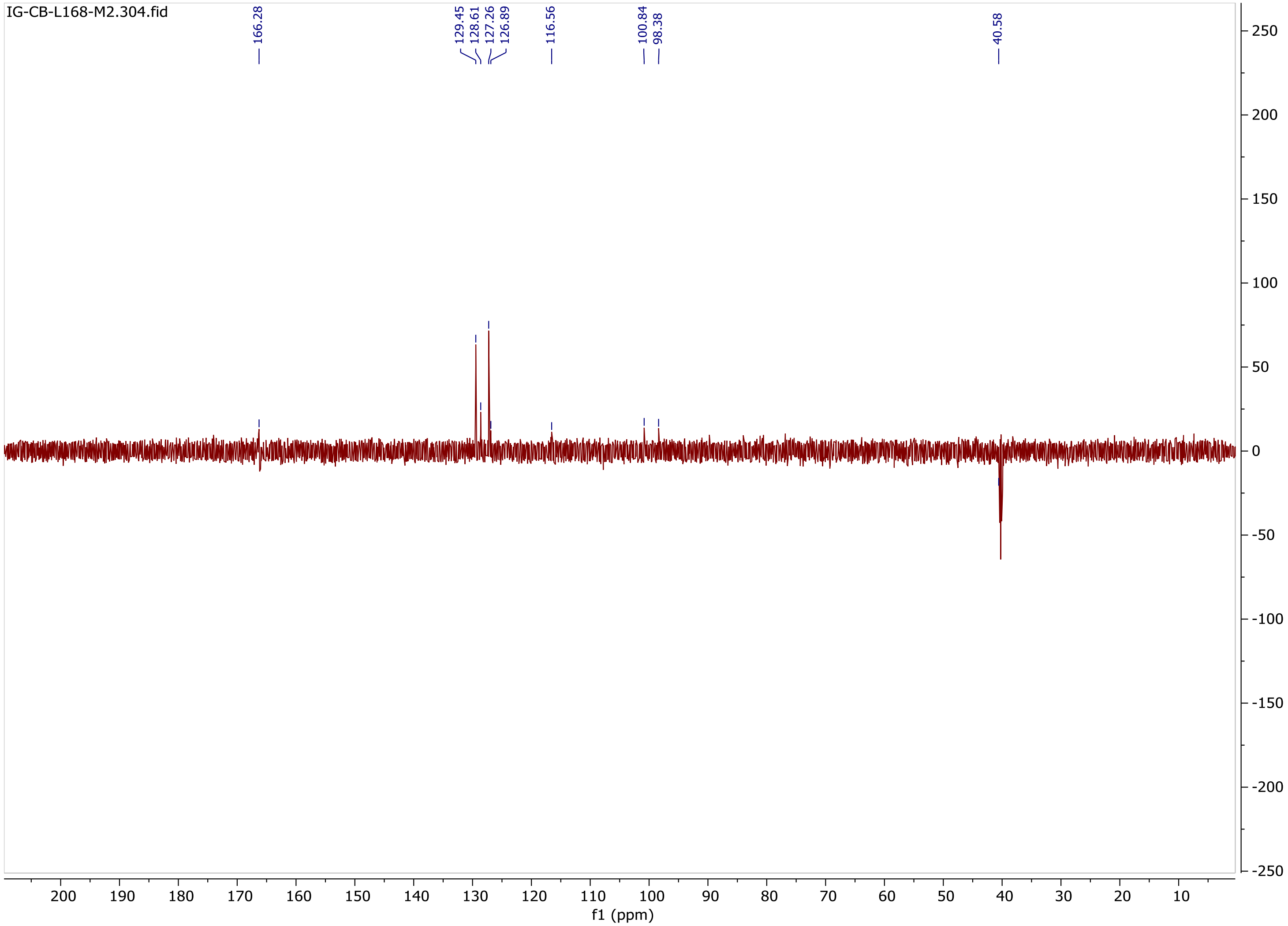
IG-CB-L168-M2.304.fid
166.28
129.45
128.61
127.26
126.89
116.56
100.84
98.38
40.58
250
200
150
100
50
0
-50
-100
-150
-200
-250
200
190
180
170
160
150
140
130
120
110
100
90
80
70
60
50
40
30
20
10
f1 (ppm)

Zgpg30

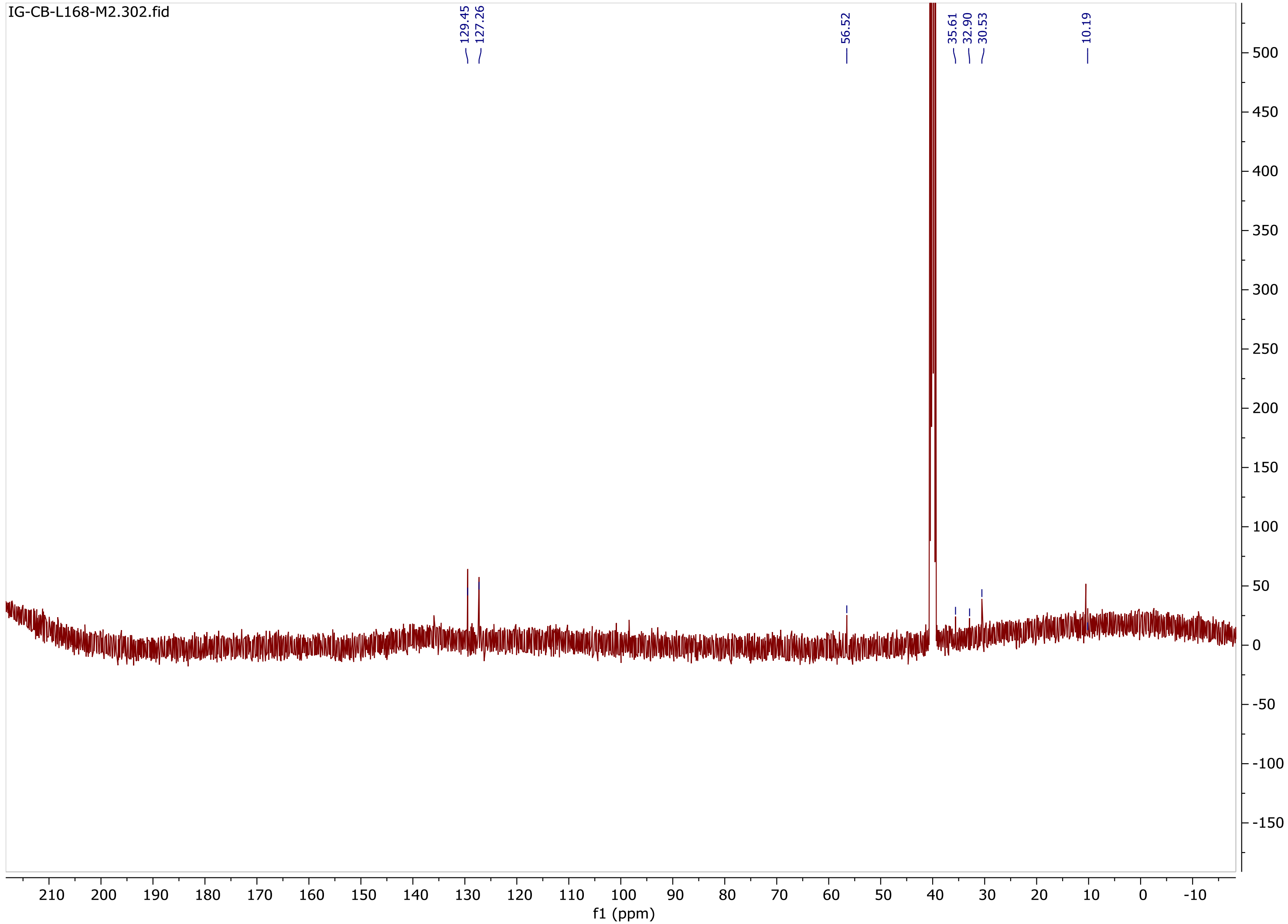
IG-CB-L168-M2.302.fid
129.45
127.26
56.52
35.61
32.90
30.53
10.19
500
450
400
350
300
250
200
150
100
50
0
-50
-100
-150
210 200 190 180 170 160 150 140 130 120 110 100 90 80 70 60 50 40 30 20 10 0 -10
f1 (ppm)

DEPT-135

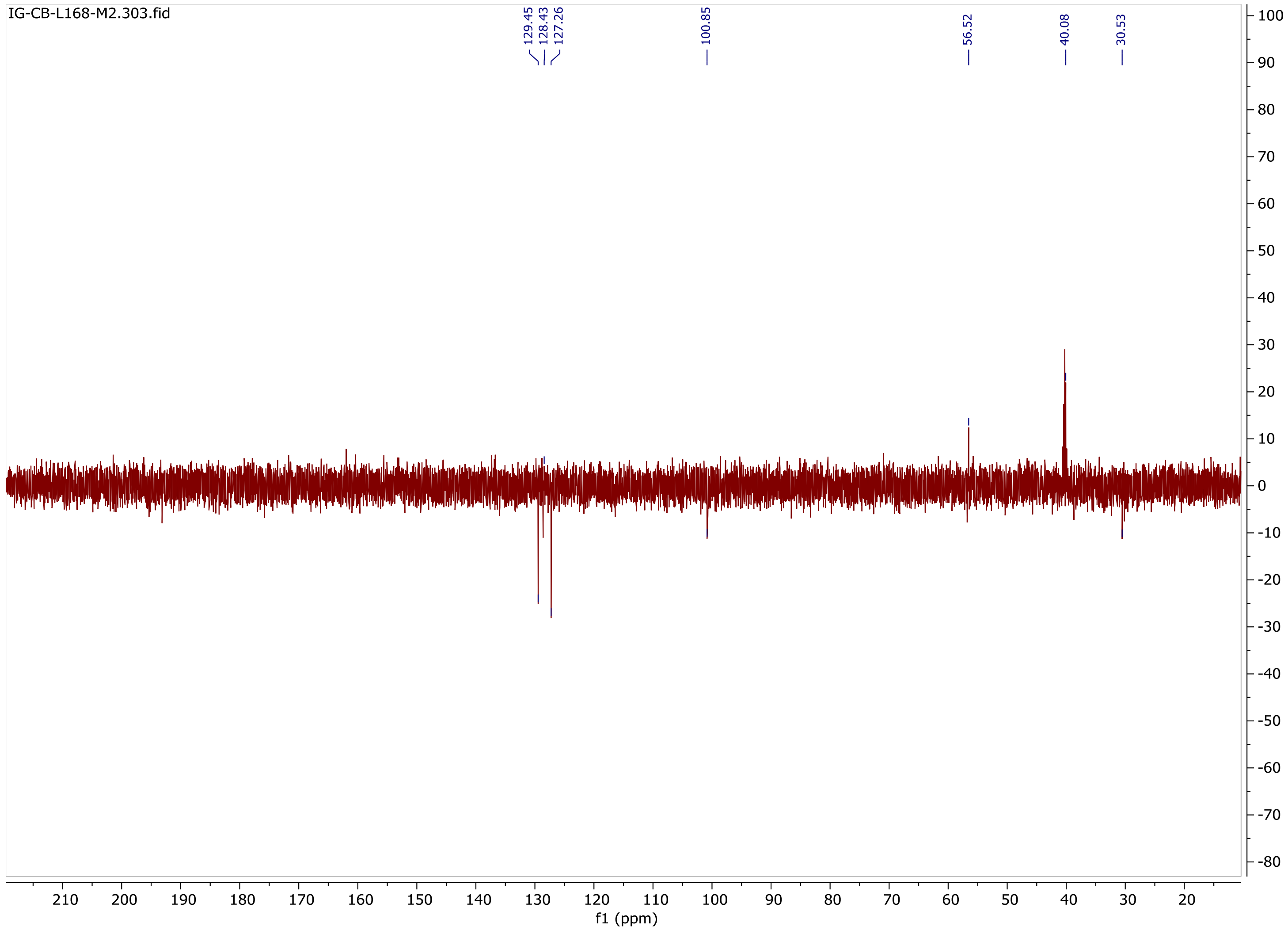
IG-CB-L168-M2.303.fid
129.45
128.43
127.26
100.85
56.52
40.08
30.53
f1 (ppm)

HRMS

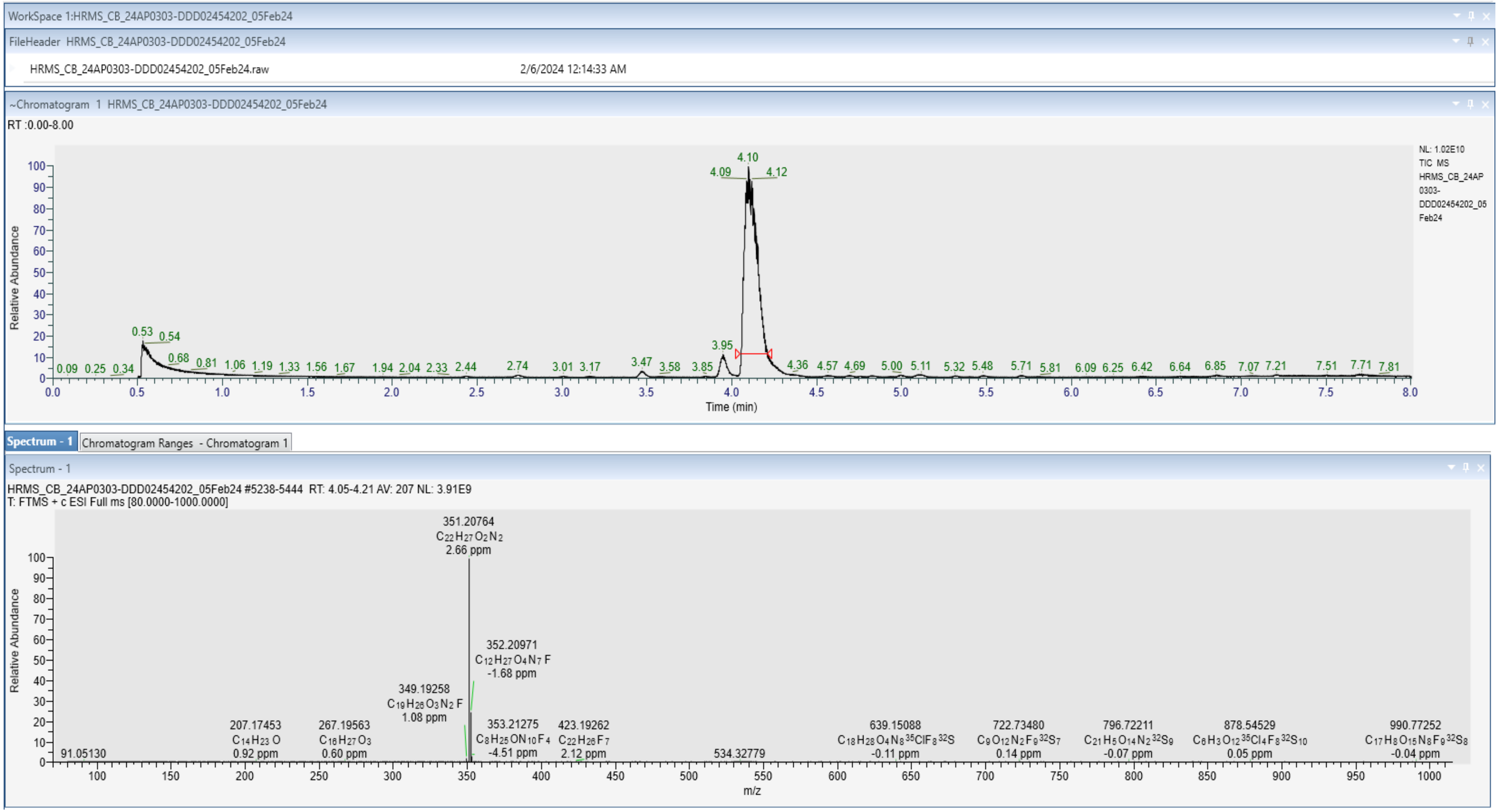
WorkSpace 1:HRMS_CB_24AP0303-DDD02454202_05Feb24
FileHeader HRMS_CB_24AP0303-DDD02454202_05Feb24
HRMS_CB_24AP0303-DDD02454202_05Feb24.raw
2/6/2024 12:14:33 AM
~Chromatogram 1 HRMS_CB_24AP0303-DDD02454202_05Feb24
RT :0.00-8.00
NL: 1.02E10 TIC MS HRMS_CB_24AP0303-DDD02454202_05Feb24
Relative Abundance
Time (min)
Spectrum - 1
Chromatogram Ranges - Chromatogram 1
HRMS_CB_24AP0303-DDD02454202_05Feb24 #5238-5444 RT: 4.05-4.21 AV: 207 NL: 3.91E9
T: FTMS + c ESI Full ms [80.0000-1000.0000]
351.20764
C22 H27 O2 N2
2.66 ppm
352.20971
C12 H27 O4 N7 F
-1.68 ppm
349.19258
C19 H26 O3 N2 F
1.08 ppm
m/z

Compound **12c**

^{1}H NMR

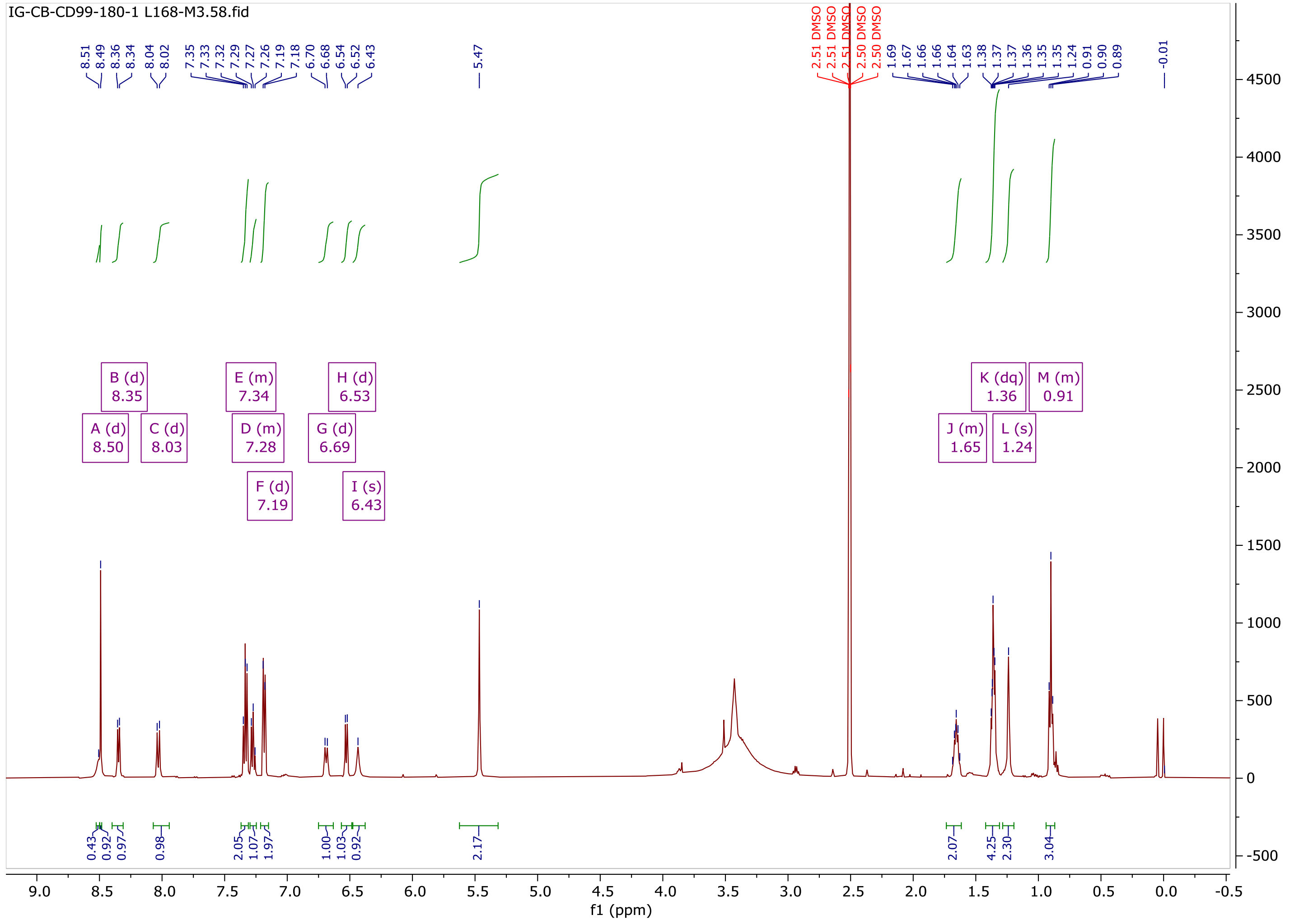

COSY

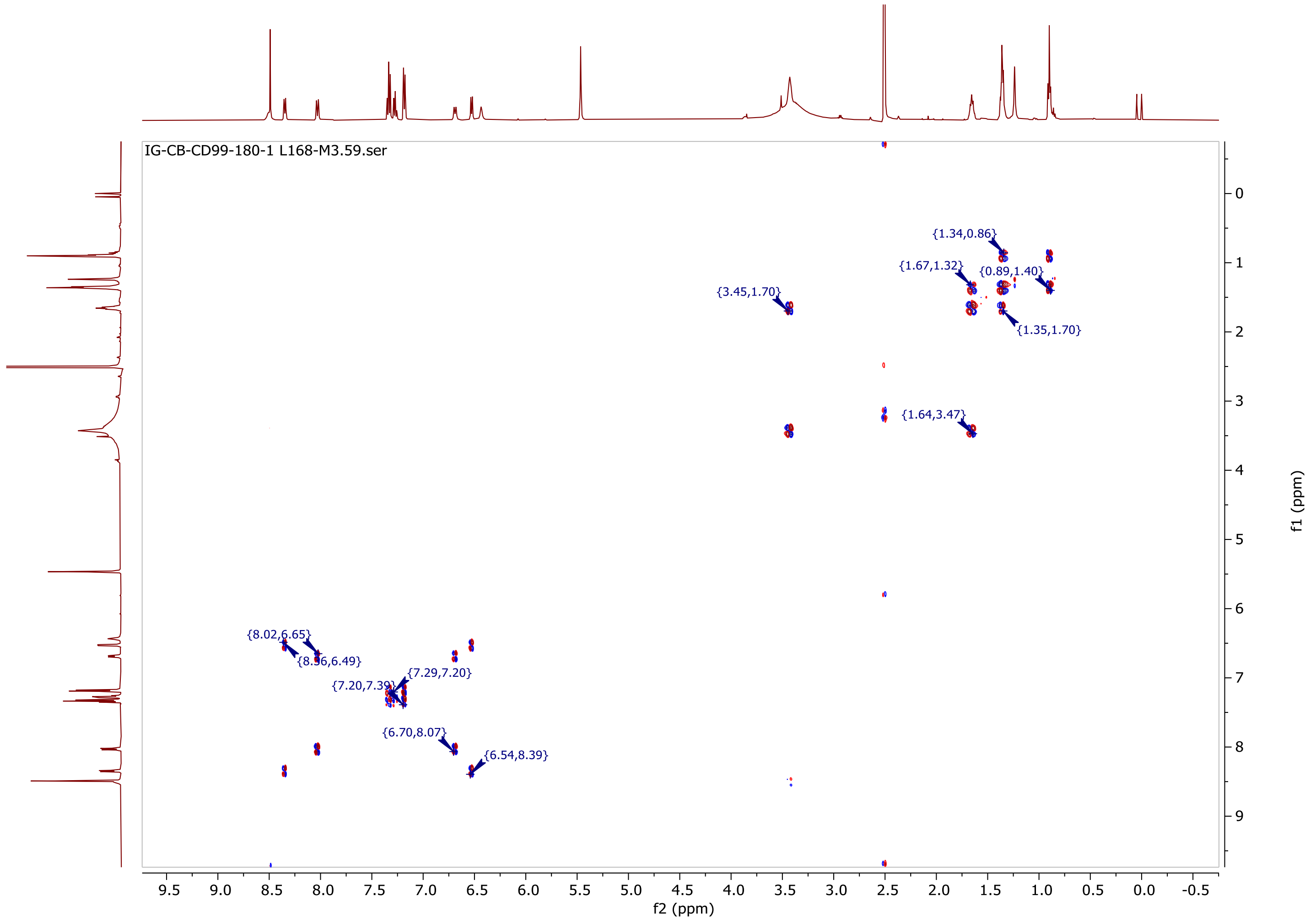
IG-CB-CD99-180-1 L168-M3.59.ser
{1.34,0.86}
{1.67,1.32}
{0.89,1.40}
{3.45,1.70}
{1.35,1.70}
{1.64,3.47}
{8.02,6.65}
{8.36,6.49}
{7.29,7.20}
{7.20,7.39}
{6.70,8.07}
{6.54,8.39}
f2 (ppm)
f1 (ppm)

HSQC

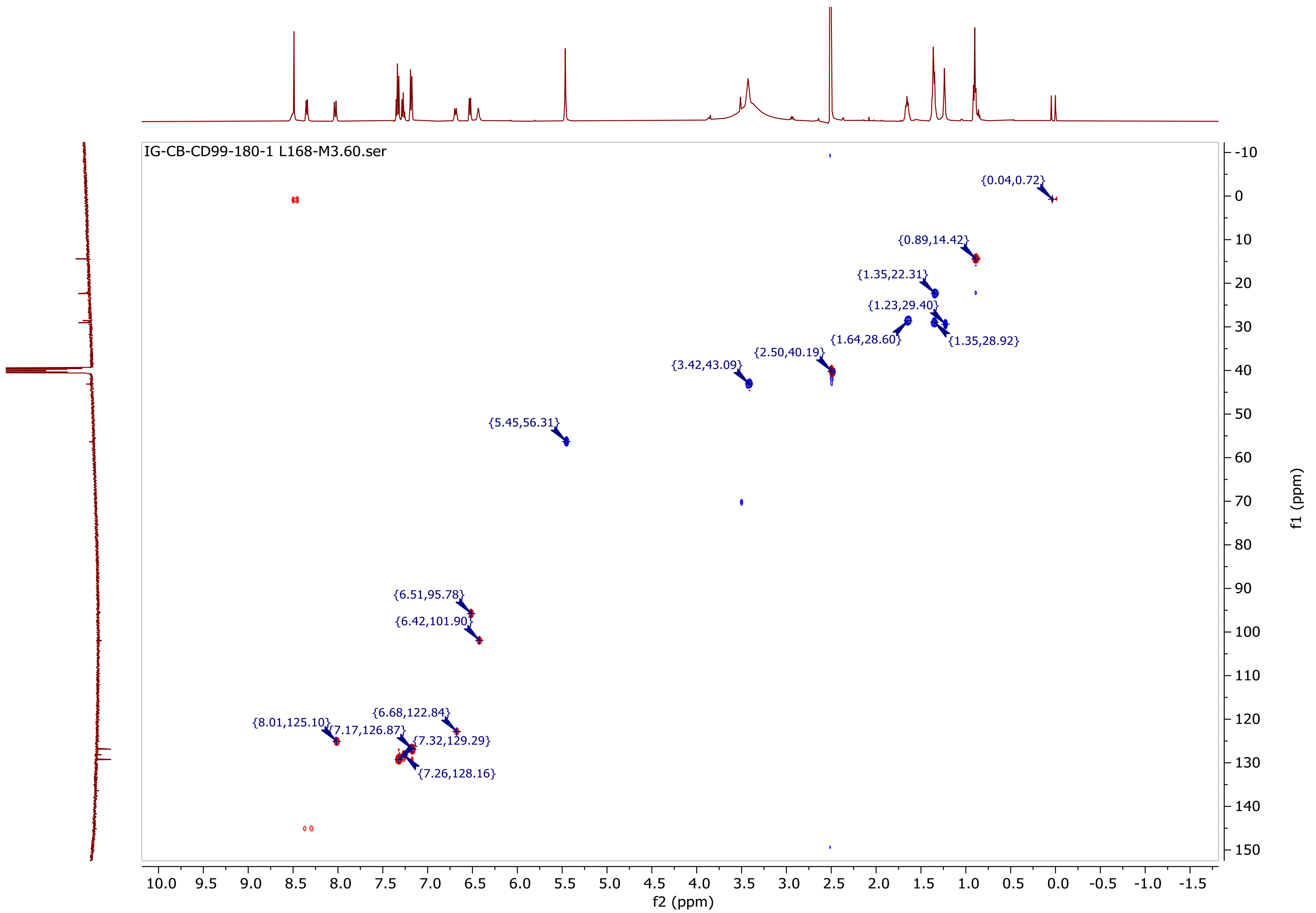
IG-CB-CD99-180-1 L168-M3.60.ser
{0.04,0.72}
{0.89,14.42}
{1.35,22.31}
{1.23,29.40}
{1.64,28.60}
{1.35,28.92}
{2.50,40.19}
{3.42,43.09}
{5.45,56.31}
{6.51,95.78}
{6.42,101.90}
{6.68,122.84}
{8.01,125.10}
{7.17,126.87}
{7.32,129.29}
{7.26,128.16}
f2 (ppm)
f1 (ppm)

HMBC

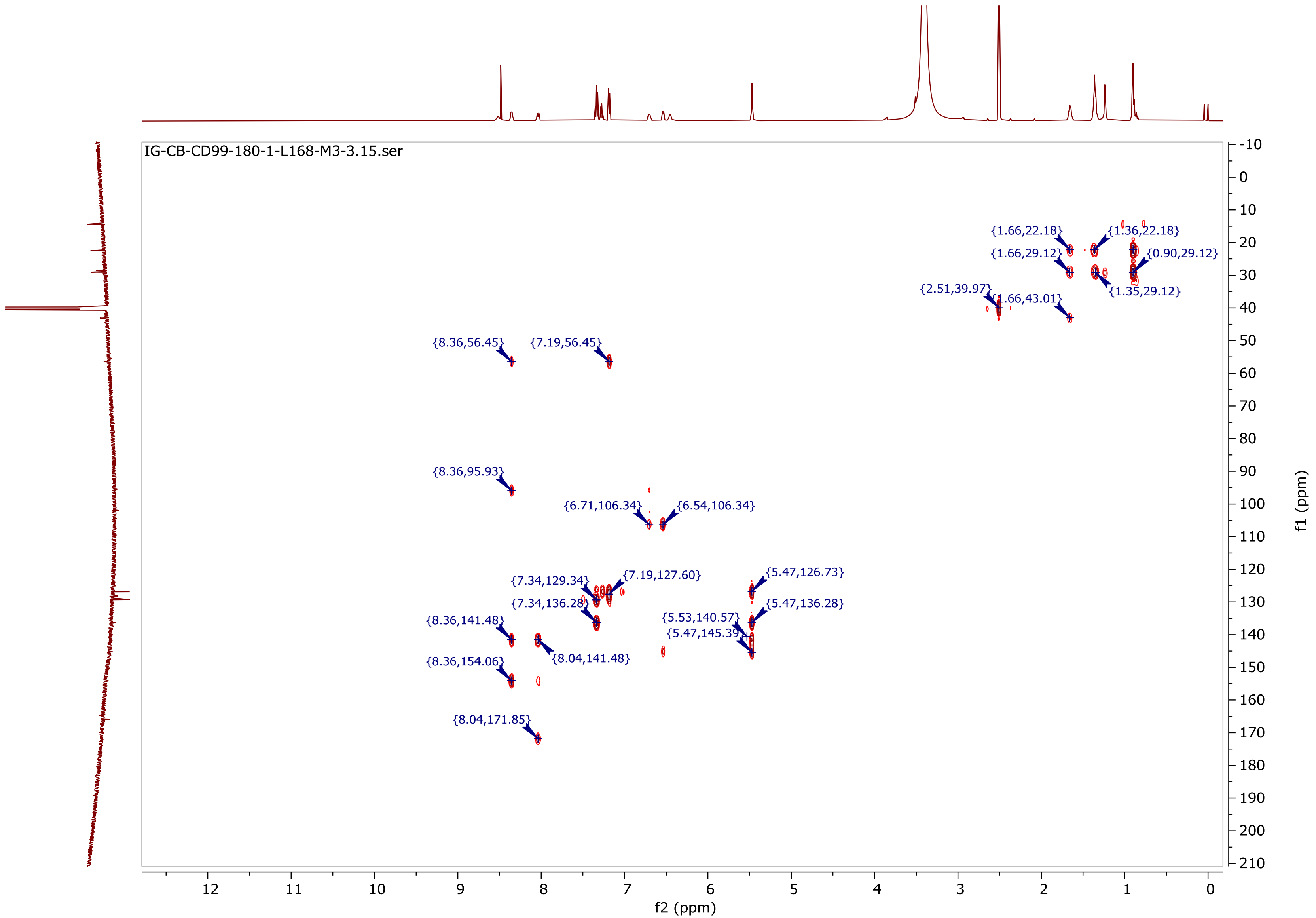

IG-CB-CD99-180-1-L168-M3-3.15.ser
{1.66,22.18}
{1.36,22.18}
{1.66,29.12}
{0.90,29.12}
{1.35,29.12}
{2.51,39.97}
{1.66,43.01}
{8.36,56.45}
{7.19,56.45}
{8.36,95.93}
{6.71,106.34}
{6.54,106.34}
{7.19,127.60}
{7.34,129.34}
{5.47,126.73}
{7.34,136.28}
{5.47,136.28}
{8.36,141.48}
{5.53,140.57}
{5.47,145.39}
{8.04,141.48}
{8.36,154.06}
{8.04,171.85}
f1 (ppm)
f2 (ppm)

S203

DEPTqgpsp

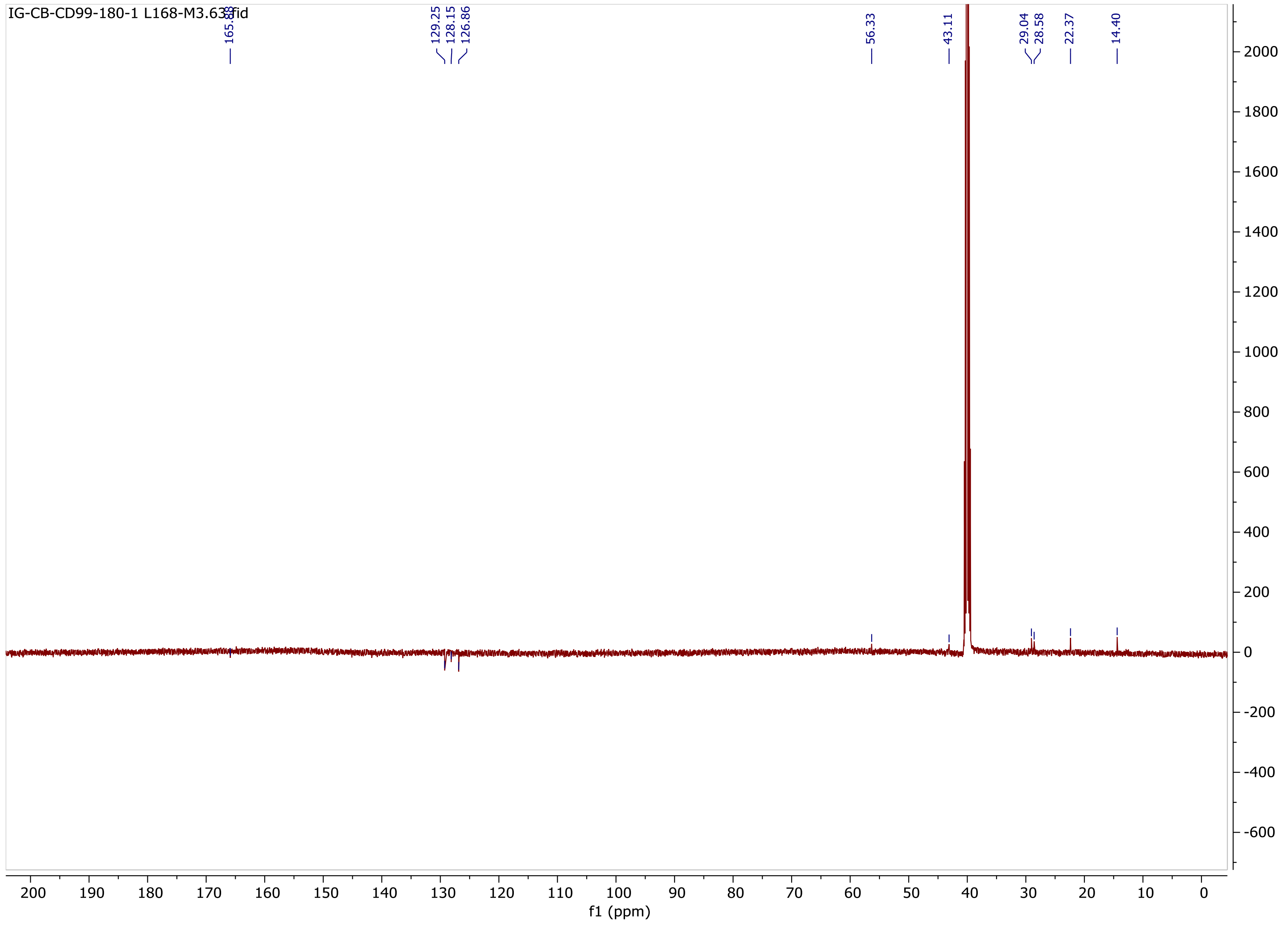
IG-CB-CD99-180-1 L168-M3.63.fid
165.93
129.25
128.15
126.86
56.33
43.11
29.04
28.58
22.37
14.40
2000
1800
1600
1400
1200
1000
800
600
400
200
0
-200
-400
-600
200
190
180
170
160
150
140
130
120
110
100
90
80
70
60
50
40
30
20
10
0
f1 (ppm)

DEPT-90

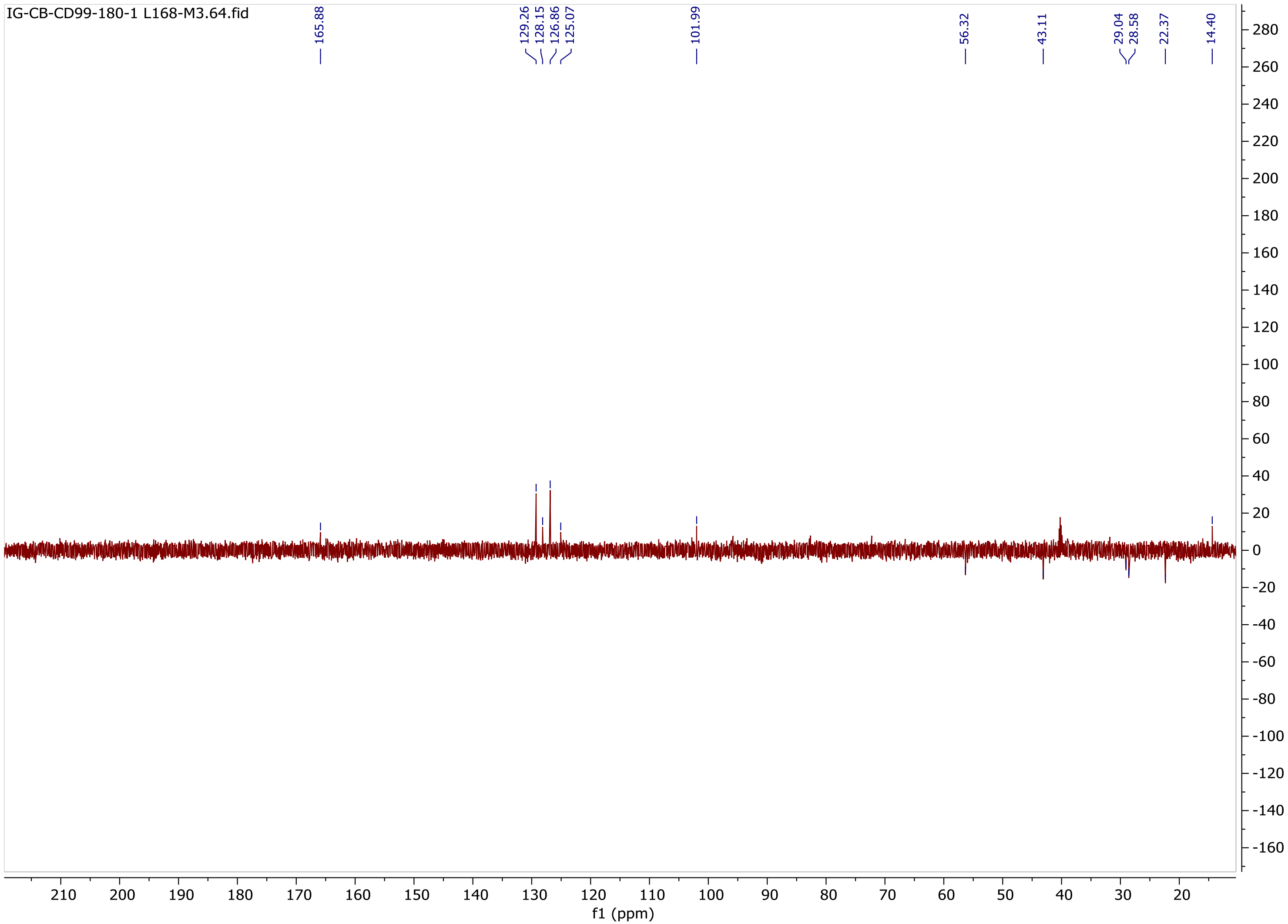
IG-CB-CD99-180-1 L168-M3.64.fid
165.88
129.26
128.15
126.86
125.07
101.99
56.32
43.11
29.04
28.58
22.37
14.40
f1 (ppm)

DEPT-135

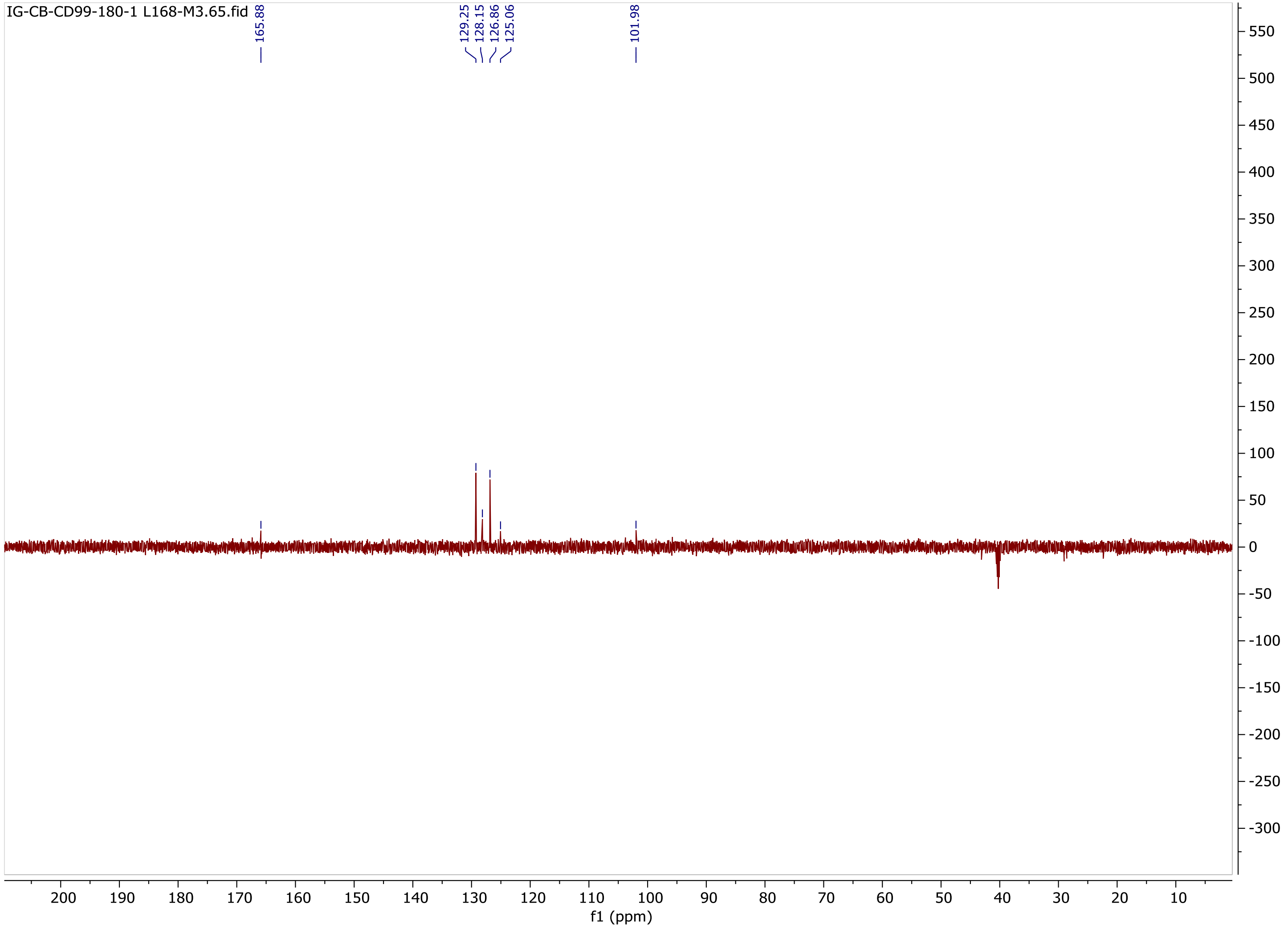

IG-CB-CD99-180-1 L168-M3.65.fid
165.88
129.25
128.15
126.86
125.06
101.98
550
500
450
400
350
300
250
200
150
100
50
0
-50
-100
-150
-200
-250
-300
200
190
180
170
160
150
140
130
120
110
100
90
80
70
60
50
40
30
20
10
f1 (ppm)

HRMS

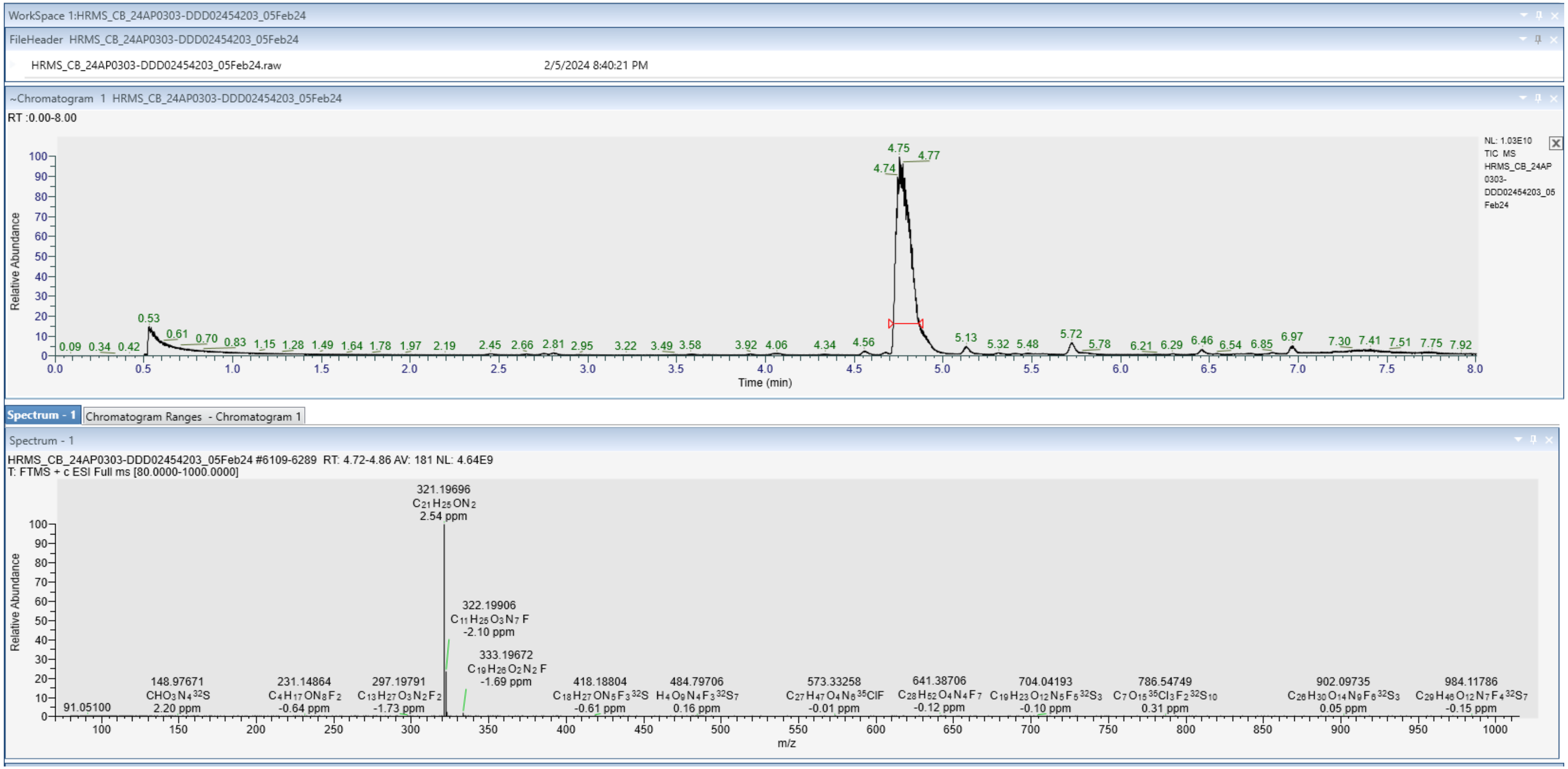
WorkSpace 1:HRMS_CB_24AP0303-DDD02454203_05Feb24
FileHeader HRMS_CB_24AP0303-DDD02454203_05Feb24
HRMS_CB_24AP0303-DDD02454203_05Feb24.raw
2/5/2024 8:40:21 PM
~Chromatogram 1 HRMS_CB_24AP0303-DDD02454203_05Feb24
RT :0.00-8.00
NL: 1.03E10 TIC MS HRMS_CB_24AP0303-DDD02454203_05Feb24
Relative Abundance
Time (min)
Spectrum - 1
Chromatogram Ranges - Chromatogram 1
HRMS_CB_24AP0303-DDD02454203_05Feb24 #6109-6289 RT: 4.72-4.86 AV: 181 NL: 4.64E9
T: FTMS + c ESI Full ms [80.0000-1000.0000]
321.19696
C21 H25 ON2
2.54 ppm
322.19906
C11 H25 O3 N7 F
-2.10 ppm
333.19672
C19 H26 O2 N2 F
-1.69 ppm
m/z

References

1) Geyer KK, Niazi UH, Duval D, Cosseau C, Tomlinson C, Chalmers IW, et al. (2017) The Biomphalaria glabrata DNA methylation machinery displays spatial tissue expression, is differentially active in distinct snail populations and is modulated by interactions with Schistosoma mansoni. PLoS Negl Trop Dis 11(5): e0005246. https://doi.org/10.1371/journal.pntd.0005246

2) Whatley KCL, Padalino G, Whiteland H, Geyer KK, Hulme BJ, Chalmers IW, et al. (2019) The repositioning of epigenetic probes/inhibitors identifies new anti-schistosomal lead compounds and chemotherapeutic targets. PLoS Negl Trop Dis 13(11): e0007693. https://doi.org/10.1371/journal. pntd.0007693

3) : Paveley RA, Mansour NR, Hallyburton I, Bleicher LS, Benn AE, et al. (2012) Whole Organism High-Content Screening by Label-Free, Image-Based Bayesian Classification for Parasitic Diseases. PLoS Negl Trop Dis 6(7): e1762. doi:10.1371/journal.pntd.0001762

4) Paradela LS, Wall RJ, Carvalho S, Chemi G, Corpas-Lopez V, Moynihan E, Bello D, Patterson S, Güther MLS, Fairlamb AH, Ferguson MAJ, Zuccotto F, Martin J, Gilbert IH, Wyllie S. Multiple unbiased approaches identify oxidosqualene cyclase as the molecular target of a promising anti-leishmanial. Cell Chem Biol. 2021 May 20;28(5):711-721.e8. doi: 10.1016/j.chembiol.2021.02.008. Epub 2021 Mar 9. PMID: 33691122; PMCID: PMC8153249.PMID: 33691122

5) L. M. MacLean, J. Thomas, M. D. Lewis, I. Cotillo, D. W. Gray, M. De Rycker, Development of Trypanosoma cruzi in vitro assays to identify compounds suitable for progression in Chagas' disease drug discovery. PLOSNeglected Tropical Diseases 12, e0006612 (2018).

6) M. De Rycker, J. Thomas, J. Riley, S. J. Brough, T. J. Miles, D. W. Gray, Identification of Trypanocidal Activity for Known Clinical Compounds Using a New Trypanosoma cruzi Hit-Discovery Screening Cascade. PLOSNeglected Tropical Diseases 10, e0004584 (2016).

7) De Rycker M, Hallyburton I, Thomas J, Campbell L, Wyllie S, Joshi D, Cameron S, Gilbert IH, Wyatt PG, Frearson JA, Fairlamb AH, Gray DW. Comparison of a high-throughput high-content intracellular Leishmania donovani assay with an axenic amastigote assay. Antimicrobial Agents Chemotherapy 57(7):2913-22 (2013).

8) Jones DC, Hallyburton I, Stojanovski L, Read KD, Frearson JA, Fairlamb AH. Identification of a κ-opioid agonist as a potent and selective lead for drug development against human African trypanosomiasis. Biochem Pharmacol. 2010 Nov 15;80(10):1478-86. doi: 10.1016/j.bcp.2010.07.038. Epub 2010 Aug 7. PMID: 20696141; PMCID: PMC3025325. PMID: 20696141
